# Integrated Systems Biology Approach Identifies Novel Maternal and Placental Pathways of Preeclampsia

**DOI:** 10.3389/fimmu.2018.01661

**Published:** 2018-08-08

**Authors:** Nandor Gabor Than, Roberto Romero, Adi Laurentiu Tarca, Katalin Adrienna Kekesi, Yi Xu, Zhonghui Xu, Kata Juhasz, Gaurav Bhatti, Ron Joshua Leavitt, Zsolt Gelencser, Janos Palhalmi, Tzu Hung Chung, Balazs Andras Gyorffy, Laszlo Orosz, Amanda Demeter, Anett Szecsi, Eva Hunyadi-Gulyas, Zsuzsanna Darula, Attila Simor, Katalin Eder, Szilvia Szabo, Vanessa Topping, Haidy El-Azzamy, Christopher LaJeunesse, Andrea Balogh, Gabor Szalai, Susan Land, Olga Torok, Zhong Dong, Ilona Kovalszky, Andras Falus, Hamutal Meiri, Sorin Draghici, Sonia S. Hassan, Tinnakorn Chaiworapongsa, Manuel Krispin, Martin Knöfler, Offer Erez, Graham J. Burton, Chong Jai Kim, Gabor Juhasz, Zoltan Papp

**Affiliations:** ^1^Perinatology Research Branch, Eunice Kennedy Shriver National Institute of Child Health and Human Development, National Institutes of Health, United States Department of Health and Human Services, Bethesda, MD, United States; ^2^Perinatology Research Branch, Eunice Kennedy Shriver National Institute of Child Health and Human Development, National Institutes of Health, United States Department of Health and Human Services, Detroit, MI, United States; ^3^Department of Obstetrics and Gynecology, Wayne State University School of Medicine, Detroit, MI, United States; ^4^Systems Biology of Reproduction Lendulet Research Group, Institute of Enzymology, Research Centre for Natural Sciences, Hungarian Academy of Sciences, Budapest, Hungary; ^5^Maternity Private Department, Kutvolgyi Clinical Block, Semmelweis University, Budapest, Hungary; ^6^First Department of Pathology and Experimental Cancer Research, Semmelweis University, Budapest, Hungary; ^7^Department of Obstetrics and Gynecology, University of Michigan, Ann Arbor, MI, United States; ^8^Department of Epidemiology and Biostatistics, Michigan State University, East Lansing, MI, United States; ^9^Center for Molecular Medicine and Genetics, Wayne State University, Detroit, MI, United States; ^10^Department of Computer Science, College of Engineering, Wayne State University, Detroit, MI, United States; ^11^Laboratory of Proteomics, Department of Physiology and Neurobiology, ELTE Eotvos Lorand University, Budapest, Hungary; ^12^Channing Division of Network Medicine, Brigham and Women’s Hospital, Harvard University, Boston, MA, United States; ^13^Zymo Research Corporation, Irvine, CA, United States; ^14^Department of Obstetrics and Gynaecology, University of Debrecen, Debrecen, Hungary; ^15^Institute of Biochemistry, Biological Research Centre, Hungarian Academy of Sciences, Szeged, Hungary; ^16^Department of Genetics, Cell and Immunobiology, Semmelweis University, Budapest, Hungary; ^17^Department of Morphology and Physiology, Semmelweis University, Budapest, Hungary; ^18^TeleMarpe Ltd, Tel Aviv, Israel; ^19^Department of Clinical and Translational Science, Wayne State University, Detroit, MI, United States; ^20^Department of Physiology, Wayne State University School of Medicine, Detroit, MI, United States; ^21^Department of Obstetrics and Gynecology, Medical University of Vienna, Vienna, Austria; ^22^Department of Obstetrics and Gynecology, Soroka University Medical Center School of Medicine, Faculty of Health Sciences, Ben-Gurion University of the Negev, Beer Sheva, Israel; ^23^Centre for Trophoblast Research, Department of Physiology, Development and Neuroscience, University of Cambridge, Cambridge, United Kingdom; ^24^Department of Pathology, Wayne State University School of Medicine, Detroit, MI, United States; ^25^Department of Pathology, Asan Medical Center, University of Ulsan, Seoul, South Korea

**Keywords:** inflammation, ischemia, liquid biopsy, omics, placenta, pregnancy, systems biology, trophoblast invasion

## Abstract

Preeclampsia is a disease of the mother, fetus, and placenta, and the gaps in our understanding of the complex interactions among their respective disease pathways preclude successful treatment and prevention. The placenta has a key role in the pathogenesis of the terminal pathway characterized by exaggerated maternal systemic inflammation, generalized endothelial damage, hypertension, and proteinuria. This *sine qua non* of preeclampsia may be triggered by distinct underlying mechanisms that occur at early stages of pregnancy and induce different phenotypes. To gain insights into these molecular pathways, we employed a systems biology approach and integrated different “omics,” clinical, placental, and functional data from patients with distinct phenotypes of preeclampsia. First trimester maternal blood proteomics uncovered an altered abundance of proteins of the renin-angiotensin and immune systems, complement, and coagulation cascades in patients with term or preterm preeclampsia. Moreover, first trimester maternal blood from preterm preeclamptic patients *in vitro* dysregulated trophoblastic gene expression. Placental transcriptomics of women with preterm preeclampsia identified distinct gene modules associated with maternal or fetal disease. Placental “virtual” liquid biopsy showed that the dysregulation of these disease gene modules originates during the first trimester. *In vitro* experiments on hub transcription factors of these gene modules demonstrated that DNA hypermethylation in the regulatory region of *ZNF554* leads to gene down-regulation and impaired trophoblast invasion, while *BCL6* and *ARNT2* up-regulation sensitizes the trophoblast to ischemia, hallmarks of preterm preeclampsia. In summary, our data suggest that there are distinct maternal and placental disease pathways, and their interaction influences the clinical presentation of preeclampsia. The activation of maternal disease pathways can be detected in all phenotypes of preeclampsia earlier and upstream of placental dysfunction, not only downstream as described before, and distinct placental disease pathways are superimposed on these maternal pathways. This is a paradigm shift, which, in agreement with epidemiological studies, warrants for the central pathologic role of preexisting maternal diseases or perturbed maternal–fetal–placental immune interactions in preeclampsia. The description of these novel pathways in the “molecular phase” of preeclampsia and the identification of their hub molecules may enable timely molecular characterization of patients with distinct preeclampsia phenotypes.

## Introduction

Preeclampsia, one of the most severe obstetrical complications affecting 5–8% of pregnant women (1–5), is a leading cause of maternal (4–15) and perinatal morbidity and mortality (6, 16–18). In addition, pathologic changes in the affected mothers and fetuses lead to a higher risk of subsequent metabolic and cardiovascular diseases later in life (8, 9, 11, 13, 19–23), further increasing healthcare costs. In spite of the severity of the problem, there is yet no early diagnosis of all forms of preeclampsia, and the current therapy is still based on the delivery of the placenta (2, 24), given the complexity of the disease and the lack of insight into the early perturbed molecular pathways.

**Figure 1:**
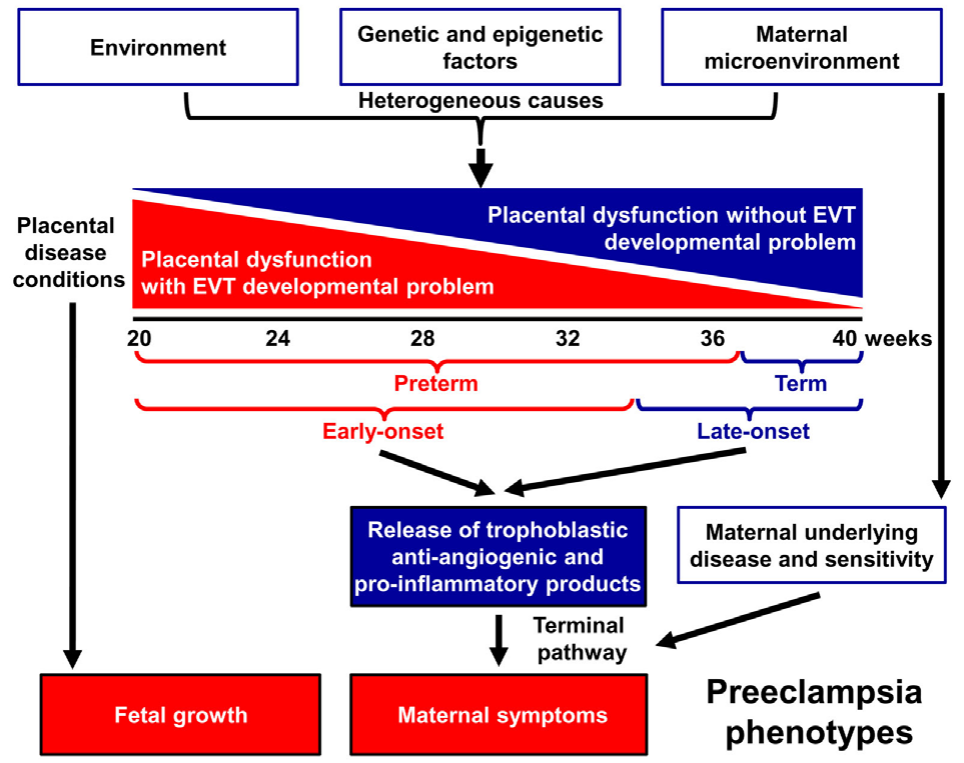
Pathogenesis of preeclampsia. Preeclampsia is a syndrome with heterogeneous etiology and a spectrum of phenotypes. It may appear at varying gestational ages with different degrees of severity and involvement of the fetus. Preterm, especially early-onset preeclampsia generally has a more severe clinical presentation in the mother and is more often associated with the delivery of a growth-restricted neonate than term or late-onset preeclampsia. It is a multi-stage disease with the maldevelopment and/or dysfunction of distinct trophoblast lineages of the placenta at the center of the disease. Villous and extravillous trophoblast (EVT) development and/or function may be impaired in the preclinical stage, most extensively in preterm preeclampsia associated with fetal growth restriction. The resulting abnormal maternal spiral artery remodeling, fluctuating blood-flow, and ischemic stress lead to placental histological changes and the release of harmful substances from the placenta. As a consequence, the terminal pathway of preeclampsia, an exaggerated maternal systemic inflammatory and anti-angiogenic condition, occurs. The frequency and severity of placental developmental problems continuously decrease with advancing gestational age. In term forms, other stressors than maternal vascular malperfusion and placental ischemia may trigger placental stress, trophoblastic dysfunction, and the induction of the terminal pathway. Alternatively, the maternal endothelium may have an exaggerated sensitivity to factors released from a relatively normal placenta.

Indeed, preeclampsia is a syndrome with heterogeneous etiology and a spectrum of phenotypes (Figure 1). It may affect women at varying gestational ages with different degrees of severity and consequences for the fetus (2, 25–31). The current classifications of preeclampsia are based upon its severity and the timing of clinical presentation, mostly dividing preeclampsia into preterm (<37 weeks) or term (≥37 weeks) and early-onset (<34 weeks) or late-onset (≥34 weeks) phenotypes (24, 26, 32–36). Preterm preeclampsia has a more severe clinical presentation and is often accompanied by fetal growth restriction compared to term preeclampsia (2, 25, 26, 29). However, severe maternal disease and fetal growth restriction may be observed in both term and preterm preeclampsia, and their presentation may not be associated with each other, suggesting that the clinical phenotype is the result of an interplay between various factors and disease pathways, also supported by observations in animal models (37).

A growing body of evidence offers support for the conclusion that the maldevelopment and/or dysfunction of distinct trophoblast lineages of the placenta have a central role in the pathogenesis of preeclampsia, and that the severity of the placental disease is subsequently reflected in the clinical phenotype of this syndrome. In the preclinical stage, extravillous trophoblast (EVT) development may be impaired, leading to EVT dysfunction, shallow trophoblast invasion, failure of the physiological transformation of the maternal spiral arteries, abnormal blood-flow to the placenta, and histological changes consistent with maternal vascular malperfusion (28, 30, 38–43). The frequency and severity of these lesions decrease from the preterm toward the term phenotype of preeclampsia (28, 33, 44–46) (Figure 1), mirrored by the decreasing prevalence of fetal growth restriction or the delivery of small-for-gestational age (SGA) neonates (47–53). Thus, EVT development is less frequently and extensively affected in late-onset preeclampsia, which constitutes about 90% of all cases (50, 54, 55).

In preterm preeclampsia, failure of the remodeling of the spiral arteries may lead to abnormal blood flow to the placenta and subsequently to placental structural damage and ischemic stress, villous trophoblast (VT) dysfunction and the release of detrimental placental substances (e.g. anti-angiogenic factors, pro-inflammatory cytokines, and syncytiotrophoblast debris) into the maternal circulation (40, 41, 43, 56–66). As a consequence, the terminal pathway of preeclampsia, featuring an anti-angiogenic state and exaggerated maternal systemic inflammation, occurs in most cases, and its intensity correlates with the severity of preeclampsia, which may be coupled with damage to the maternal endothelium and to the kidneys, liver, and central nervous system during the clinical phase (21, 42, 43, 64, 67–72). VT development can also be impaired in preeclampsia (73–75), especially in the preterm form associated with SGA, where VT turnover is affected together with morphometric features (73, 76).

Term preeclampsia is characterized by a lesser magnitude of maternal systemic inflammatory and anti-angiogenic states (30, 37, 55–57, 61, 62, 77–92). This phenotype may result from different stressors other than maternal vascular malperfusion and ischemia of the placenta, which include various preexisting maternal disorders, such as obesity, chronic hypertension, diabetes, and metabolic, kidney, and autoimmune diseases (25, 93, 94). These stressors may still trigger placental stress and VT dysfunction (31, 91) and induce a maternal pro-inflammatory milieu. Alternatively, maternal endothelial dysfunction may result from an exaggerated sensitivity to factors released from the placenta (21, 25, 31, 95), which increases the risk of preeclampsia upon maternal genetic predisposition for cardiovascular disease (96, 97). Indeed, preeclampsia has a genetic predisposition with high heritability of both phenotypes, and it shares common risk alleles with coronary artery disease (98–104).

In spite of extensive research efforts, our understanding of the early pathologic pathways of preeclampsia has been limited given several obstacles. First, the complexity of the disease pathways and the heterogeneity of the syndrome have not been investigated in an integrative manner in both maternal and placental compartments throughout pregnancy. Second, it has been impossible to investigate the early placental disease pathways because of the invasive nature of placental biopsy and the limited information on placental functions obtained non-invasively. Consequently, an increasing number of high-dimensional studies aiming to detect molecular signatures of preeclampsia either in the placenta or in maternal blood have mostly targeted later stages of pregnancy, at a more advanced stage of placental development and pathology (105–143). Third, animal models of preeclampsia fail to mimic early placental pathways of preeclampsia due to the anatomical and physiological uniqueness of deep placentation in humans (144–147). Fourth, *in vitro* studies on human placental development and trophoblast functions are hindered by the lack of self-replicating trophoblast stem cells with the ability to differentiate into both VTs and EVTs (148–150).

**Figure 2:**
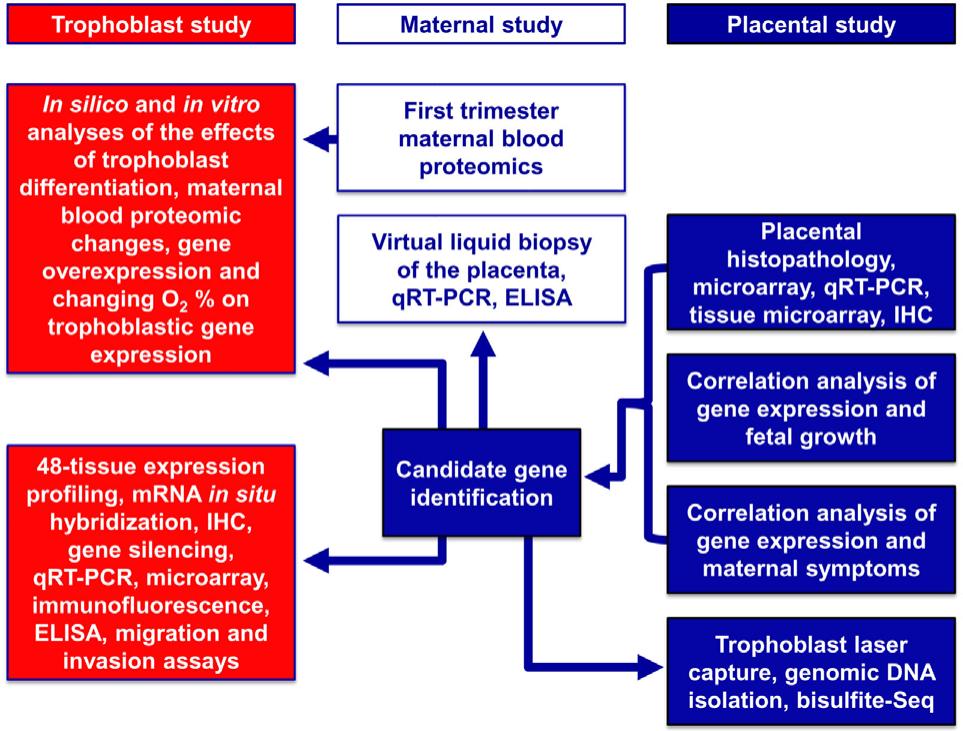
Flow-chart of experimental procedures. The placental study included extensive histologic, transcriptomic, epigenetic, and protein level investigations of the placenta to target the molecular pathways in the center of disease. Molecular changes were correlated with disease outcomes in both mothers and babies to uncover placental pathways affecting either maternal or fetal wellbeing. The maternal study included first trimester maternal blood proteomics and “virtual” liquid biopsy of the placenta to reveal blood factors of maternal or placental origin that can reflect disease conditions in early pregnancy. In the trophoblast study, hub transcription factors in placental gene modules separately associated with maternal or placental/fetal disease were investigated with various *in vitro* methods to model *in vivo* disease pathways.

Here, we used a systems biology approach that integrated various omics and targeted methods to investigate both placental and maternal compartments and most aspects of preeclampsia, including placental disease; fetal and maternal outcomes; environmental, maternal, microenvironmental, and epigenetic factors; and trophoblastic functions. In the first placental study, we performed extensive investigations of the placenta at histologic, transcriptomic, epigenetic, and protein levels to target molecular pathways at the center of the disease. Molecular changes were correlated with maternal and neonatal morbidities associated with preeclampsia to uncover placental pathways affecting either maternal or fetal wellbeing. In the second, maternal study, we employed maternal blood proteomics and “virtual” liquid biopsy of the placenta to reveal blood factors of maternal or placental origin that can reflect disease conditions in early pregnancy. In the third, trophoblast study, we utilized *in vitro* functional assays on the trophoblast to investigate hub transcription factors at the center of placental disease gene modules and to model their *in vivo* involvement in placental pathways associated with maternal or placental/fetal disease (Figure 2).

## Results

### Placental Study

#### Alterations in the Placental Transcriptome in Preterm Preeclampsia

**Figure 3:**
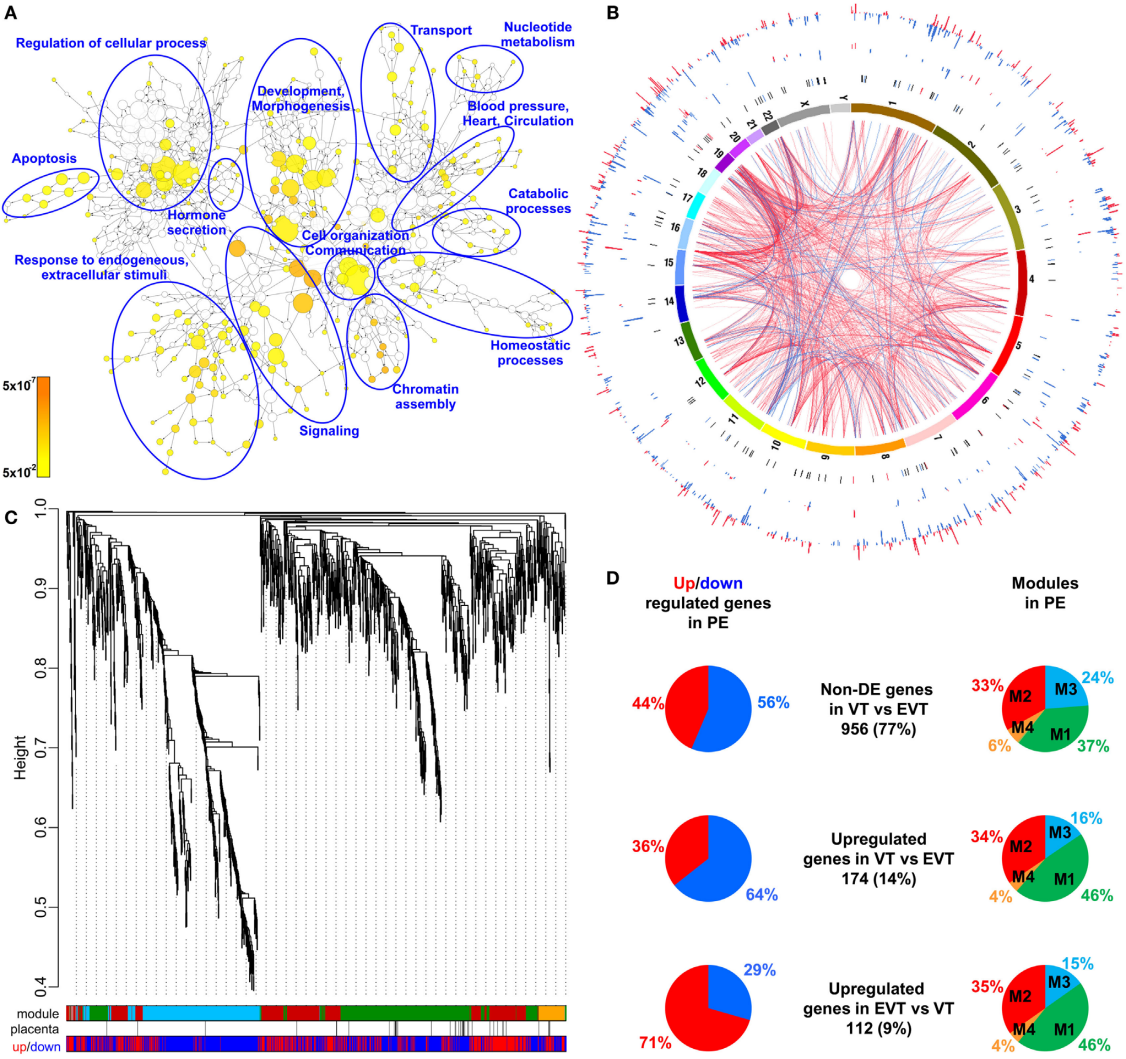
Placental transcriptomic changes in preterm preeclampsia. **(A)** The network of biological processes enriched among differentially expressed (DE) genes. Circle sizes relate to the number of genes involved in the biological processes; colors refer to *p*-values according to the color code. Groups of the most enriched biological processes were circled and labeled. **(B)** Circos visualization of DE genes in preterm preeclampsia (PE). The inner circle shows chromosomes; curved lines represent correlation (red: positive; blue: negative) between transcription regulatory genes and their targets; the second circle shows genomic location of predominantly placenta-expressed genes (black lines: non-DE; red or blue lines: up- or down-regulated); third and fourth circles show the locations of DE transcription regulatory genes and their targets, respectively with blue (down-regulated) and red (up-regulated) bars. The height of the bars represents the magnitude of expression changes. **(C)** Dysregulated placental gene expression was characterized by four major disease gene modules within DE genes, marked with different colors. The height plotted on the *y*-axis represents distance metric. Of 38 predominantly placenta-expressed genes (black vertical lines), 33 belonged to the M1 (green, *n* = 22) and M2 (red, *n* = 11) modules. These modules were enriched in predominantly placenta-expressed genes and enriched in up-regulated (M2) or down-regulated (M1) genes marked under the modules with red or blue lines, respectively. **(D)** Pie charts depict the distribution of three gene sets among dysregulated genes or gene modules. Genes with similar expression in villous (VT) and extravillous trophoblast (EVT) had similar distribution among up- or down-regulated genes and in modules M1 and M2. Genes with up-regulation in VT compared to EVT were predominantly down-regulated in preeclampsia and positioned in module M1. Genes with up-regulation in EVT compared to VT were predominantly up-regulated in preeclampsia and positioned also in module M1.

Given that the pathogenesis of preeclampsia has been implicated to originate from the placenta, we first aimed to investigate placental transcriptomic changes leading to placental dysfunction as well as regulatory networks involved in the pathologic pathways. The combined analysis of preterm preeclampsia cases and gestational age-matched controls (*n* = 17) in a Hungarian patient population (Table 1) in our placental microarray data (132) revealed 1,409 differentially expressed (DE) genes (Data S1 in Supplementary Material), which are involved in fundamental cellular processes, including blood pressure (BP) regulation, apoptosis, development, hormone secretion, metabolism, homeostasis, and signaling (Figure 3A). DE genes included 137 transcription regulatory genes and 38 predominantly placenta-expressed genes. This latter set of genes (*n* = 164, Data S2 in Supplementary Material), which was defined by BioGPS microarray data (*n* = 153) or expression data from separate studies in the lack of BioGPS data (*n* = 11) (151–153), was enriched among DE genes in preeclampsia [odds ratio (OR) = 3.4, *p* = 6.9 × 10^−9^]. This suggests that genes predominantly expressed by the placenta have pathologic and diagnostic significance in preeclampsia, a phenomenon indicated earlier by our targeted studies (153–155).

**Table 1:** Demographics of Hungarian women included in the placental microarray study.

Groups	Controls for preterm PE (*n* = 5)	Preterm PE (*n* = 12)
Maternal age (years)^b^	31.6 (31.5–34.3)	30.3 (26.1–35)
Primiparity^a^	40	66.7
Gestational age (weeks)^b^	31.0 (30.9–34.0)	31.2 (29.3–33.2)
Race^a^		
Caucasian	100	100
African-American	0	0
Other	0	0
Systolic BP (mmHg)^b^	120 (120–120)	163 (160–170)^c^
Diastolic BP (mmHg)^b^	80 (70–80)	100 (100–101)^c^
Proteinuria^a^	0	100^c^
Birthweight (g)^b^	1,990 (1,640–2,210)	1,065 (990–1,420)
Cesarean delivery^a^	100	100

*BP, blood pressure; PE, preeclampsia*.

*^a^Percentage*.

*^b^Median (interquartile range)*.

*^c^p < 0.01*.

Subsequently, we investigated the genomic links among DE genes by searching for genomic regions associated with the observed placental transcriptomic changes. We found that Chr6 (OR = 1.54, *q* = 1.6 × 10^−3^) and Chr7 (OR = 1.42, *q* = 0.02) were particularly affected by these gene expression changes (Data S3 in Supplementary Material), while Chr19 (OR = 2.6, *q* = 0.02) was enriched in dysregulated transcription regulatory genes (Data S4 in Supplementary Material). Of interest, predominantly placenta-expressed genes were also enriched on Chr19 (OR = 2.5, *q* = 0.002) (Data S5 in Supplementary Material). Figure 3B shows the non-random genomic localization of DE genes in preterm preeclampsia and the pronounced gene dysregulation associated with Chr19. These results are consistent with the fact that Chr19 harbors large transcription regulatory gene families (156), and probably reflect its regulatory role in placental/trophoblastic gene expression and their dysregulation in preeclampsia.

#### Alterations in Biological Processes and Regulatory Networks in Preterm Preeclampsia

Next, we aimed to identify functional links among DE genes by identifying gene co-expression network modules and hub transcription regulatory genes driving differential expression in the modules. Weighted co-expression network analysis (WGCNA) was conducted among DE genes resulting in the assignment of these into four major modules, labeled as M1 (green, *n* = 506), M2 (red, *n* = 442), M3 (blue, *n* = 381), and M4 (orange, *n* = 74) (Figure 3C; Data S1 in Supplementary Material). Most predominantly placenta-expressed genes belonged to modules M1 (*n* = 22 genes) and M2 (*n* = 12). Module M1 was enriched in down-regulated genes (OR = 1.88, *p* = 2.59 × 10^−8^), while module M2 was enriched in up-regulated genes (OR = 6.47, *p* = 2.2 × 10^−16^), suggesting the presence of distinct dysregulated gene-networks. Genes with predominant VT expression (14%) were mainly down-regulated, while genes with predominant EVT expression (9%) were mainly up-regulated even though both sets had the most members in module M1 (Figure 3D). These data suggested that the functions of both VT and EVT are strongly impacted in preterm preeclampsia, albeit in different ways.

Predominantly placenta-expressed genes, down-regulated in module M1, are regulators of fetal growth (*CSH1, HSD11B2*) (157, 158), metabolism (*ESRRG*) (159), estrogen synthesis (*HSD17B1*) (160), and immune functions (*LGALS14*) (153), some of which were reported to be down-regulated in preeclampsia (30, 91, 130, 155, 161, 162). Within this module, *ESRRG, POU5F1*, and *ZNF554* transcription regulatory genes had the highest number of significant correlations with predominantly placenta-expressed genes and, hence, deemed as hub factors (Figure S1A in Supplementary Material). Of note, these transcription factors have been implicated in the regulation of stemness and differentiation (163, 164), pointing to the possible involvement of module M1 in the dysregulation of trophoblast differentiation in preterm preeclampsia. We selected *ZNF554* for functional studies, since it belongs to the KRAB zinc finger family, crucial for early embryonic development and differentiation (165), and it may regulate genes in its co-expression network involved in biological processes affected by preeclampsia, such as development, chromatin assembly, signaling, adhesion, migration, and metabolism (Figure S1B in Supplementary Material).

In module M2, *FLT1*, which expresses sFlt-1, the main driver of BP elevation in the terminal pathway of preeclampsia (56, 90), was up-regulated. Moreover, module M2 genes were strongly overrepresented (OR = 29.9, *p* = 6.54 × 10^−95^) among genes that had correlated expression with mean arterial pressure (MAP) (Data S6 in Supplementary Material), suggesting a key role for this module in promoting hypertension. Within this module, *BCL6, BHLHE40*, and *ARNT2* had the highest correlation in gene expression with predominantly placenta-expressed genes, including *FLT1* (Figure S1A in Supplementary Material). Of note, these transcription regulatory hub genes are involved in hypoxia response. ARNT2 heterodimerizes with hypoxia-response regulator HIF-1α that is involved in trophoblast invasion and the pathogenesis of preeclampsia (166–169), and ARNT2 is a key regulator for adaption to hypoxic conditions at high altitudes, where the incidence of preeclampsia is much higher (16%) than at low altitude (170, 171). *BHLHE40* links immune and hypoxia-induced pathways (172). *BCL6*, a gene previously found to be up-regulated in the placenta in preterm preeclampsia, represses trophoblast differentiation and is regulated by stress-activated protein kinase signaling pathways (135, 173, 174). Our co-expression analysis revealed an enrichment of biological processes, such as differentiation, apoptosis, metabolism, signaling, and responses to stimuli including oxygen among genes co-expressed with *BCL6* (Figure S1C in Supplementary Material). Since *BCL6* is a key player in inflammation and oxygen-driven regulation of cell fate, we selected this gene for functional studies along with *ARNT2*.

#### The Association of Gene Modules With Clinical Parameters

Next, to validate the microarray results on a larger patient population (*n* = 100) comprised mostly of subjects of African-American origin presenting various phenotypes of preeclampsia (Table 2; Figure S2 in Supplementary Material), we selected 47 genes for qRT-PCR profiling of 100 placentas (Table 3), provided these genes were: (1) dysregulated genes with predominant placental and syncytiotrophoblastic expression, potentially encoding biomarkers; (2) dysregulated hub transcription regulatory genes that had a high co-expression with dysregulated, predominantly placenta-expressed genes in modules M1 and M2; and (3) non-dysregulated genes with roles in trophoblast differentiation, trophoblast-specific gene expression, or the pathogenesis of preeclampsia.

**Table 2:** Demographics of American women included in the placental validation study.

Groups	Controls for preterm PE (*n* = 20)	Preterm PE (*n* = 20)	Preterm PE with SGA (*n* = 20)
Maternal age (years)^b^	22 (20–28.5)	23.5 (21–27)	22.5 (19.5–30)
Primiparity^a^	20	40	25
Gestational age (weeks)^b^	32.3 (28.2–34.9)	31.4 (29.6–33.6)	31.8 (29.7–34.4)
Race^a^			
Caucasian	5	10	10
African-American	95	90	90
Other	0	0	0
Systolic BP (mmHg)^b^	116 (110–125)	177 (166–187)^c^	171 (164–189)^c^
Diastolic BP (mmHg)^b^	65 (59–71)	105 (103–111)^c^	108 (94–118)^c^
Proteinuria^b^	0	3 (2–3)^c^	3 (3–3)^c^
Birthweight (g)^b^	1,635 (1,075–2,715)	1,488 (1,050–1,908)	1,173 (908-1,650)^d^
Birthweight percentile^b^	40.5 (31.9–53.4)	22.7 (18.3–32.9)^d^	6.7 (1–8.6)^c^
Cesarean delivery^a^	45	80^d^	75

**Groups**	**Controls for term PE (*n* = 20)**	**Term PE (*n* = 10)**	**Term PE with SGA (*n* = 10)**

Maternal age (years)^b^	22 (21–32)	19 (19–35)	26.5 (19–31)
Primiparity^a^	15	40	10
Gestational age (weeks)^b^	38.6 (38–39.1)	39.1 (38.6–39.6)	38.4 (37.3–38.9)
Race^a^			
Caucasian	15	0	0
African-American	80	100	100
Other	5	0	0
Systolic BP (mmHg)^b^	121 (111–134)	173 (165–178)^c^	169 (164–190)^c^
Diastolic BP (mmHg)^b^	70 (64–73)	106 (102–110)^c^	102 (97–104)^c^
Proteinuria^b^	0	3 (1–3)^c^	3 (1–3)^c^
Birthweight (g)^b^	3,215 (3,110–3,335)	3,123 (2,990–3,200)	2,405 (2,205–2,555)^c^
Birthweight percentile^b^	46 (37.2–63)	37.1 (28.5–48.8)	1.1 (1–3.5)^c^
Cesarean delivery^a^	55	40	20

*BP, blood pressure; PE, preeclampsia; SGA, small-for-gestational age*.

*^a^Percentage*.

*^b^Median (interquartile range)*.

*^c^p < 0.001*.

*^d^p < 0.05*.

**Table 3:** Genes included in the placental qRT-PCR validation study.

Gene symbol	DE genes in preterm PE (microarray)	Gene modules (microarray)	Hub TR genes in modules (microarray)	TR genes	PPE genes	DE genes in preterm PE (qRT-PCR)	DE genes in term PE (qRT-PCR)
*ARNT2*	Up	M2	M2	Yes	–	**Up**	–
*BCL3*	Up	M1	–	Yes	–	–	**Up**
*BCL6*	Up	M2	M2	Yes	–	**Up**	–
*BTG2*	Down	M2	–	Yes	–	**Down**	**Down**
*CDKN1A*	Down	M1	–	–	–	–	Up
*CGB*	Up	M4	–	–	Yes	–	**Up**
*CLC*	Up	M2	–	–	–	**Up**	**Up**
*CLDN1*	Down	M1	–	–	–	**Down**	**Down**
*CRH*	Up	M2	–	–	Yes	**Up**	–
*CSH1*	–	M1	–	–	Yes	Down	–
*CYP19A1*	–	–	–	Yes	Yes	–	–
*DUSP1*	Up	M2	–	–	–	**Up**	–
*ENG*	–	–	–	–	–	Up	Up
*ERVFRDE1*	–	–	–	–	Yes	–	–
*ERVWE1*	–	–	–	–	Yes	–	–
*ESRRG*	Down	M1	M1	Yes	Yes	**Down**	–
*FBLN1*	Down	M1	–	–	Yes	–	–
*FLT1*	Up	M2	–	–	–	**Up**	**Up**
*GATA2*	–	–	–	Yes	–	–	–
*GCM1*	–	–	–	Yes	Yes	Down	–
*GH2*	–	–	–	–	Yes	–	–
*HLF*	Down	M1	–	Yes	–	**Down**	–
*HSD11B2*	Down	M1	–	–	Yes	–	–
*HSD17B1*	Down	M1	–	–	Yes	**Down**	**Down**
*IKBKB*	Up	M1	–	–	–	–	–
*INSL4*	Up	M4	–	–	Yes	–	–
*JUNB*	Up	M2	–	Yes	–	**Up**	–
*KIT*	Up	M2	–	–	–	**Up**	–
*LEP*	Up	M2	–	–	Yes	**Up**	**Up**
*LGALS13*	–	–	–	–	Yes	Down	Down
*LGALS14*	Down	M1	–	–	Yes	**Down**	**Down**
*LGALS16*	–	–	–	–	Yes	–	–
*LGALS17A*	–	–	–	–	–	–	–
*MAPK13*	–	–	–	–	–	–	Up
*NANOG*	–	–	–	Yes	–	–	–
*PAPPA*	–	–	–	–	Yes	–	–
*PAPPA2*	Up	M2	–	–	Yes	**Up**	**Up**
*PGF*	–	–	–	–	Yes	–	–
*PLAC1*	Down	M1	–	–	Yes	**Down**	–
*POU5F1*	Up	M1	M1	Yes	–	–	–
*SIGLEC6*	Up	M2	–	–	Yes	**Up**	**Up**
*TEAD3*	–	–	–	Yes	Yes	–	Up
*TFAM*	Down	M3	–	Yes	–	–	Up
*TFAP2A*	–	–	–	Yes	Yes	–	–
*TPBG*	Up	M2	–	–	–	**Up**	**Up**
*VDR*	Up	M2	–	Yes	–	–	–
*ZNF554*	Down	M1	M1	Yes	–	**Down**	**Down**

*PE, preeclampsia; DE, differentially expressed; TR, transcription regulatory; PPE, predominantly placenta-expressed*.

*Identical dysregulation in the validation cohort as in the microarray cohort is depicted in bold font*.

**Figure 4:**
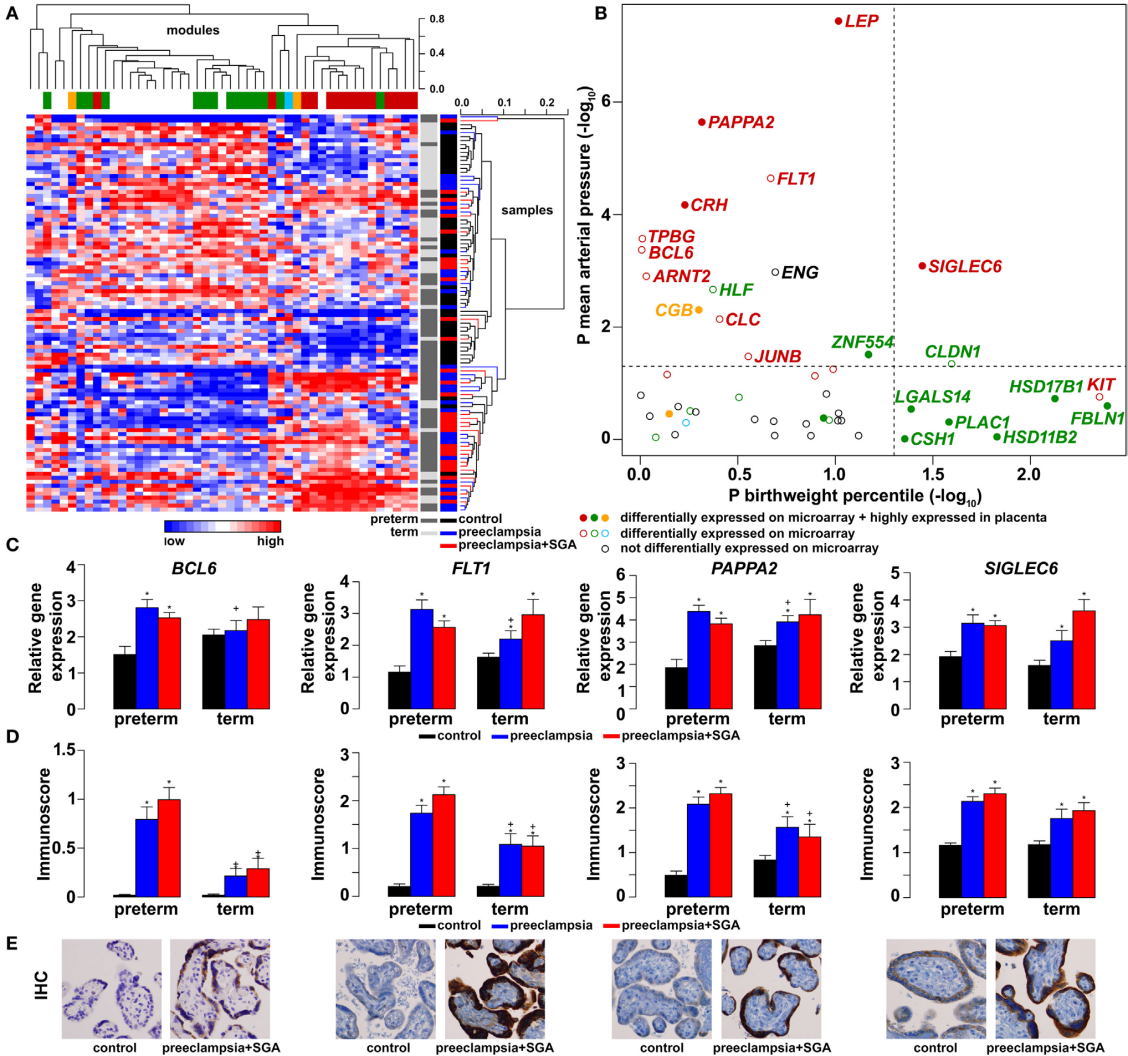
Gene modules associated with blood pressure (BP) and birthweight (BW). **(A)** Hierarchical clustering of qRT-PCR data obtained with 100 samples and 47 genes. Pearson correlation was used for similarity analysis and average method for linkage calculation. Samples were colored according to patient groups and maturity status. M1 (green) and M2 (red) module genes and 34 of 60 samples from women with preeclampsia clustered together. **(B)** Association of gene expression with BP and BW. The significance *p*-values for these coefficients were plotted for all genes, colored according to module classification (black: not changed on the microarray). Filled circles represent predominantly placenta-expressed genes and dashed lines the significance threshold at *p* = 0.05. Seven of 9 genes related to BW belong to module M1, while 10 of 15 genes related to BP are from module M2. Gene expression **(C)** and protein immunostaining **(D)** of selected four genes in module M2 show similar patterns in the sub-groups of preeclampsia, and semi-quantitative immunoscorings validate gene expression data. Significant differences in preterm or term preeclampsia samples compared to gestational-age matched controls are shown with “*”. When the change with preeclampsia in preterm samples was different from that in term samples, a “+” marks such an interaction. Comparisons for all genes are available in Figure S3 in Supplementary Material. **(E)** Representative images of the same placenta from a preterm control (left, 29 weeks) and a patient with preterm preeclampsia associated with SGA (right, 31 weeks) are shown for the immunostainings (hematoxylin counterstaining, 40× magnification).

As depicted in the heatmap representing qRT-PCR data for the 100 placentas and 47 genes (Figure 4A), validation data supported microarray experiments in terms of (1) differential expression of selected genes, (2) high correlation of genes within modules M1 and M2, and (3) separate dysregulation of modules M1 and M2 from each other. Comparison between preterm preeclampsia and gestational age-matched controls revealed that qRT-PCR data validated microarray results (differential or non-differential gene expression) for 33 of 47 genes (70%), and further confirmed the differential expression of four genes that were non-dysregulated in the microarray cohort (Table 3). As expected, the extent of changes in gene expression was less pronounced in term preeclampsia (Table 3; Figure 4C; Figure S3 in Supplementary Material). As a further confirmation, there was a strong correlation between qRT-PCR data as well as tissue microarray (TMA) and immunostaining results for selected proteins in module M2 (Figures 4C–E). The extent of dysregulation was larger in preterm phenotypes of preeclampsia than in term phenotypes, in agreement with the more severe placental pathology (44). Among transcription regulatory genes of module M1, *HLF* had strong down-regulation only in preterm preeclampsia while *ZNF554* was expressed at a lower level in all preeclampsia phenotypes (Figure S3 in Supplementary Material). Among the transcription regulatory genes in module M2, *ARNT2, BCL6*, and *JUNB* were highly expressed in preterm but not in term preeclampsia, suggesting that these might play a role only in the pathology of preterm cases (Figure 4C; Figure S3 in Supplementary Material). Overall, these data reflect the heterogeneous placental pathology and the more severely affected pathways in preterm preeclampsia.

Because of the possible involvement of these two gene modules in distinct pathologic pathways, we correlated qRT-PCR data with maternal and fetal clinico-pathological indicators to further investigate their association (Figure 4B). This analysis showed that 7 of 9 genes related to the birthweight (BW) percentile were from module M1, while 10 of 15 genes related to BP were from module M2, confirming our observations with microarray data. Therefore, we decided to also refer to these as “M1-BW” and “M2-BP” modules. Of interest, the expression of most (9/14) M1 genes was negatively associated with the “maternal vascular malperfusion” score of the placenta, while the expression of most (10/14) M2 genes was positively correlated with this parameter (Data S7 in Supplementary Material). This observation fits well with the accepted concept that maternal vascular malperfusion of the placenta leads to oxidative stress (40, 41, 63, 175) and the increased placental expression of *FLT1* (63).

### Maternal Study

#### Maternal Blood Reflects Gene Module Dysregulation in Preeclampsia

Next, we investigated whether placental gene module dysregulation can be detected in maternal blood and whether liquid biopsy can be used to determine when the module dysregulation occurs. To study the relationship between placental gene expression and maternal biomarker concentrations, we selected predominantly placenta-expressed genes with the most differential expression in modules M1 (*CSH1*) or M2 (*LEP*), and correlated their placental expression levels with concentrations of their secreted protein products (hPL and leptin) in maternal blood collected in both first (*n* = 12) and third trimesters (*n* = 19) in a Hungarian patient population. Positive correlations were found between placental gene expression and maternal blood protein concentrations for both biomarkers in both trimesters (Figure S4 in Supplementary Material), suggesting that certain proteins in the maternal circulation reflect the placental expression of their encoding genes throughout pregnancy.

**Figure 5:**
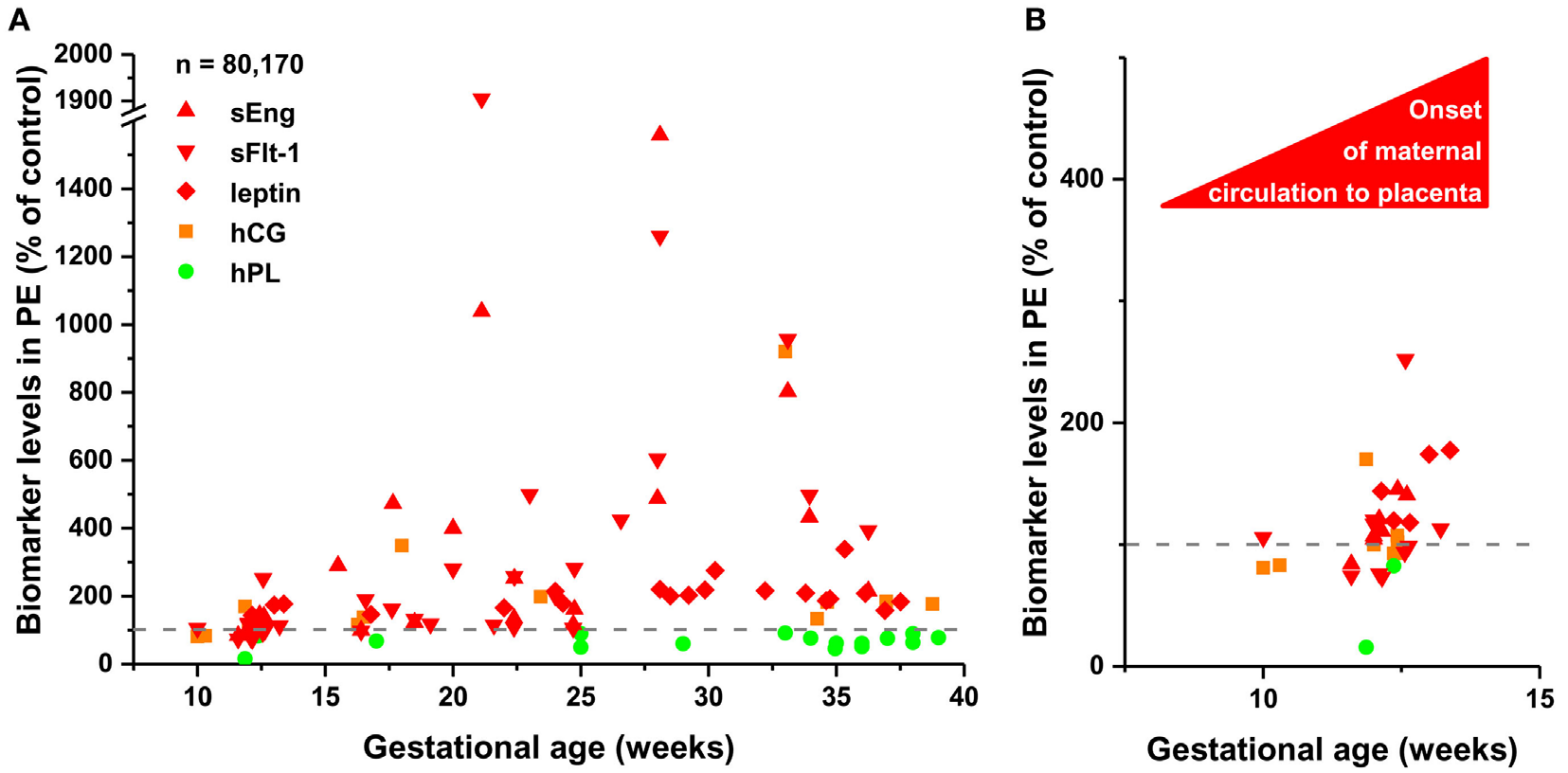
The timing of gene module dysregulation in preterm preeclampsia. **(A)** A database of 80,170 measurements published in 61 reports (35, 61, 82, 88, 126, 178–233) was built for “virtual” liquid biopsy of the placenta in preterm preeclampsia with maternal blood levels of proteins with the highest expression in the placenta among all tissues [i.e. hCG, human placental lactogen (hPL), sEng, sFlt-1, and leptin]. Levels of these biomarkers in preterm preeclampsia were expressed as the percentage of control levels (dashed line) and were represented in scatter plots by different colors reflecting gene module classification. Based on qRT-PCR data, sEng belongs to module M2 (red). hPL (M1, green module) levels in preeclampsia were constantly below control levels during gestation, while the levels of module M2 biomarkers in preeclampsia had continuous elevation compared to control levels as a function of gestational age. **(B)** Analysis of first trimester data revealed the lower expression of all biomarkers in preeclampsia than in controls before the onset of maternal circulation, and the increasing expression of module M2 biomarkers in preeclampsia compared to controls after 12 weeks of gestation.

To detect disease-associated protein signatures of placental dysfunction in maternal blood, similar to plasma DNA tissue mapping for noninvasive prenatal assessments (176, 177), we performed “virtual” liquid biopsy of the placenta in preterm preeclampsia (Figure S5 in Supplementary Material; Figure 5). We identified five genes with predominant placental expression, which have products extensively investigated in maternal blood in preterm preeclampsia or in all cases of preeclampsia during all trimesters. From data of 61 reports (35, 61, 82, 88, 126, 178–233), we built a database of 80,170 measurements in which preeclampsia data were expressed as a percentage of average levels in controls. Regarding biomarkers in module M1, we found hPL levels in preterm preeclampsia to be consistently below control levels throughout pregnancy. This is substantiated by the down-regulation of another M1 biomarker, HSD17B1, in first trimester maternal blood (160), confirming a generalized down-regulation of M1 biomarkers in early pregnancy. By contrast, the levels of biomarkers in module M2 had constant elevation in preterm preeclampsia compared to controls during gestation. When analyzing only data collected in the first trimester, prior to the onset of maternal circulation to the placenta, levels of M1 and M2 biomarkers were lower in women with preeclampsia compared to controls, while after 12 weeks of gestation, following the opening of the intervillous spaces to maternal blood flow, patients had increasing levels of M2 biomarkers in maternal blood compared to controls. These findings offer support for the conclusion that placental transcriptomic changes typical for preeclampsia in the third trimester are rooted in the first trimester.

#### Altered Maternal Serum Proteome in Preeclampsia

**Figure 6:**
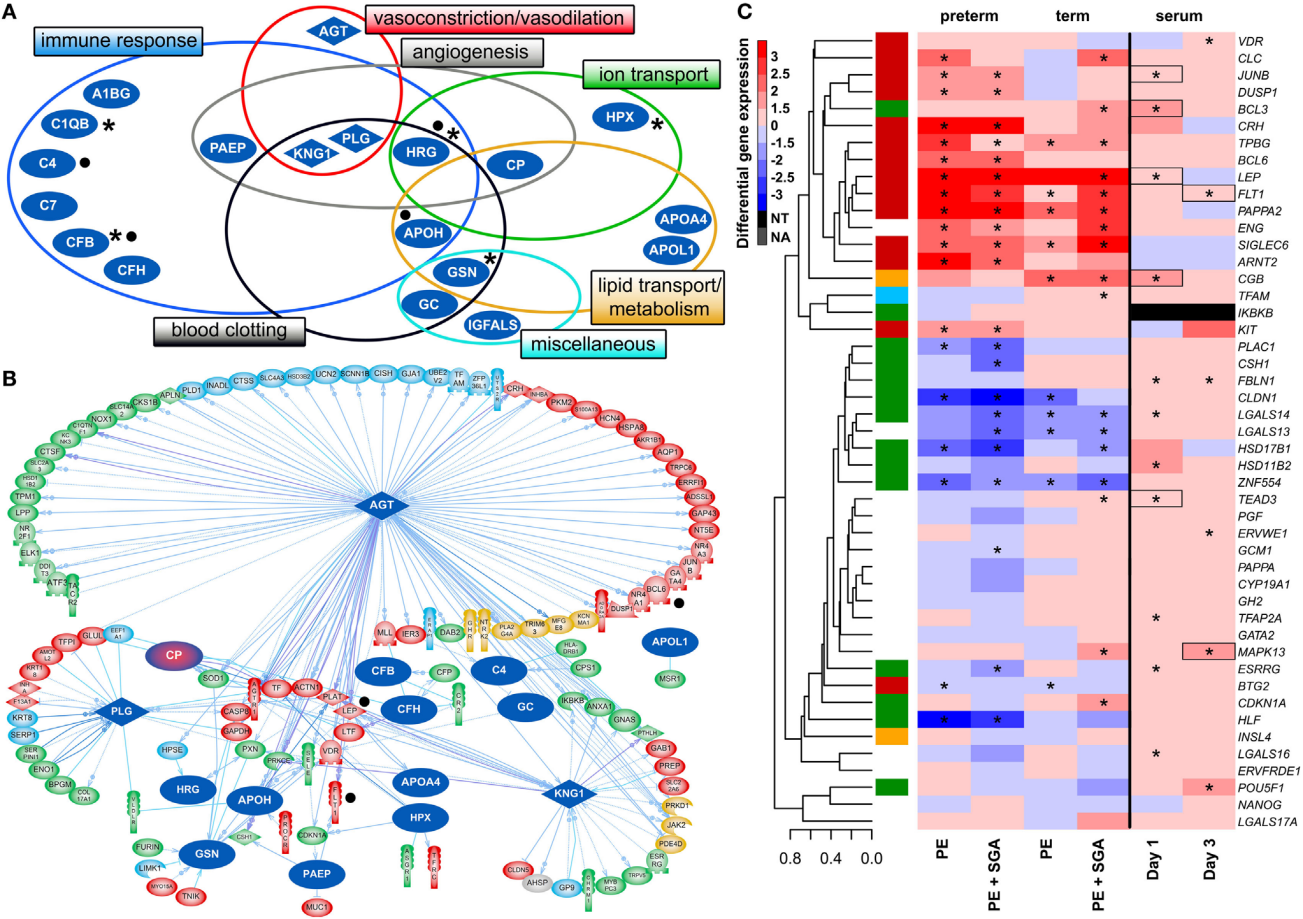
Maternal blood proteomic changes and their effect on differentially expressed (DE) genes in preterm preeclampsia. **(A)** The 19 DE maternal serum proteins in preterm preeclampsia identified by two-dimensional differential in-gel electrophoresis could be classified into functional groups relevant for preeclampsia pathophysiology. Asterisks denote proteins reported by others to have the same direction differential abundance in the first trimester in patients developing preterm preeclampsia (126, 143, 239–241). Dots denote immune proteins that multiple reaction monitoring assays identified to have differential abundance in preterm preeclampsia in the same direction. **(B)** The 19 DE serum proteins have connections to 121 DE placental genes, among which 48 belong to module M2. Angiotensinogen has more connections than other proteins (OR = 1.9, *p* = 4.9 × 10^−5^), and the most connections to module M2 genes (*n* = 35); 77 of 86 connections of angiotensinogen have a directional effect toward the gene. Dots denote genes in connection to angiotensinogen found up-regulated in villous trophoblast (VT) upon treatment with first trimester serum from preeclamptic women. **(C)** Hierarchical clustering tree and heatmap representing differential placental gene expression in preeclampsia as in Figure 4A. The expression of the same genes (except *IKBKB*) was determined in primary VTs upon treatment with first trimester maternal serum. Serum from preeclamptic compared to control women induced the up-regulation of seven placental dysregulated genes (depicted with boxes) on the first and third days of VT differentiation, among which six were up-regulated in term and three in preterm preeclampsia. Stars depict significant changes, color bar encodes signed (up or down)-fold changes. Abbreviations: NA, not expressed; NT, not examined.

Next, we investigated the maternal serum proteome in early pregnancy in distinct phenotypes of preeclampsia and the potential effects of proteomic changes on the placental transcriptome. Comparing samples from women with preterm preeclampsia associated with SGA and their respective controls from an Israeli patient population (*n* = 10, Table 4), 19 DE protein spots were identified and investigated by mass spectrometry. According to public data, many of these proteins have a role in immune response, complement and coagulation cascades, lipid transport and metabolism, angiogenesis, BP regulation, and ion transport (Figure 6A, Data S8 in Supplementary Material). Comparing samples from women with term preeclampsia and their respective controls from this Israeli patient population (*n* = 10, Table 4), 14 DE protein spots could be identified (Figure S6A; Data S8 in Supplementary Material). Many of the proteins found in these spots are the same as those found in preterm preeclampsia or function in the same pathways. Of note, these pathways and the 26 differentially abundant proteins identified in term and preterm preeclampsia had mostly been implicated in a later stage of preeclampsia (43, 101, 120, 234–238). We concluded that there is a common dysregulation of the maternal serum proteome in term and preterm preeclampsia; however, the extent of changes is larger in the latter, in agreement with the more fulminant and early pathogenesis.

**Table 4:** Demographics of Israeli women included in the maternal blood two-dimensional differential in-gel electrophoresis proteomics study.

Groups	Controls for preterm PE with SGA (*n* = 5)	Preterm PE with SGA (*n* = 5)	Controls for term PE (*n* = 5)	Term PE (*n* = 5)
Maternal age (years)^b^	30 (24–31)	29 (27–37)	29 (27–34)	26 (23–32)
Primiparity^a^	40	20	80	40
Gestational age at blood draw (weeks)^b^	10 (9–11)	8 (8–9)	8 (8–9)	9 (8–10)
Gestational age at delivery (weeks)^b^	39.7 (38.6–40.0)	34.9 (29.3–35.3)^d^	38.7 (38.6–41.0)	38.1 (38.0–38.1)
Race^a^				
Caucasian	100	100	100	100
African-American	0	0	0	0
Other	0	0	0	0
Systolic BP (mmHg)^b^	105 (104–110)	160 (150–165)^c^	110 (110–118)	150 (140–160)^c^
Diastolic BP (mmHg)^b^	60 (60–70)	100 (100–100)^c^	67 (63–68)	100 (90–100)^c^
Proteinuria^b^	0	4 (3–4)^c^	0	3 (3–4)^c^
Birthweight (g)^b^	2,955 (2,900–3,100)	1,720 (975–1,800)^c^	3,390 (3,270–3,600)	3,200 (3,150–3,210)
Cesarean delivery^a^	0	60^c^	60	40

*BP, blood pressure; PE, preeclampsia; SGA, small-for-gestational age*.

*^a^Percentage*.

*^b^Median (interquartile range)*.

*^c^p < 0.05 when compared to corresponding controls*.

*^d^p < 0.001 when compared to corresponding controls*.

Supporting our findings, five studies (126, 143, 239–241) found proteins with differential abundance in preterm preeclampsia in the same direction as our two-dimensional differential in-gel electrophoresis (2D-DIGE) study (Figure 6A). We also collected maternal blood specimens in the first trimester from a Hungarian patient population (*n* = 15, Table 5), and measured the concentrations of 10 immune proteins in patients with preterm preeclampsia associated with SGA and matched controls using liquid chromatography–mass spectrometry multiple reaction monitoring (MRM). In spite of the difference between the methods and ethnic background, MRM identified 4 of these 10 proteins as having differential abundance in the same direction as in the 2D-DIGE study, supporting the early pro-inflammatory changes in the maternal proteome in preterm preeclampsia (Figure 6A; Data S9 in Supplementary Material). MRM and proteomic evidence published to date supported 37% (7/19) of the proteomic changes detected by 2D-DIGE.

**Table 5:** Demographics of Hungarian women included in the maternal blood multiple reaction monitoring proteomics study.

Groups	Controls for preterm PE with SGA (*n* = 10)	Preterm PE with SGA (*n* = 5)
Maternal age (years)^b^	29.7 (28.9–31.5)	34.7 (28.6–35.1)
Primiparity^a^	20	80
Gestational age at blood draw (weeks)^b^	12.6 (12.6–12.8)	12.4 (12.4–13.0)
Gestational age at delivery (weeks)^b^	40.3 (39.9–40.8)	32.4 (26.4–34.0)^c^
Race^a^		
Caucasian	100	100
African-American	0	0
Other	0	0
Systolic BP (mmHg)^b^	124 (118–126)	170 (170–170)^c^
Diastolic BP (mmHg)^b^	75 (68–79)	100 (100–110)^c^
Proteinuria (g)^b^	0	3 (1–6)^c^
Birthweight (g)^b^	3,460 (3,308–3,623)	1,380 (790–1,450)^c^
Cesarean delivery^a^	30	100^c^

*BP, blood pressure; PE, preeclampsia; SGA, small-for-gestational age*.

*^a^Percentage*.

*^b^Median (interquartile range)*.

*^c^p < 0.05*.

#### The *In Silico* Effects of Altered Maternal Serum Proteome on the Placenta

To reveal whether early proteomic changes in maternal blood may affect the placental transcriptome, placental DE genes with documented connections to DE proteins in maternal serum were identified by Pathway Studio. The 121 DE placental genes with connections to DE serum proteins in preterm preeclampsia were marginally over-represented by those from module M2 (48/121, OR = 1.4, *p* = 0.057). Angiotensinogen had the largest number of connections to DE genes including *FLT1* and *LEP* (*n* = 86, OR = 1.9, *p* = 4.9 × 10^−5^). This protein also had the largest number of connections to module M2 genes (*n* = 35), followed by plasminogen (*n* = 11) and kininogen-1 (*n* = 9), all involved in BP regulation (Figure 6B). These data were supported by the “renin-angiotensin signaling” as being a top pathway (*p* = 1.28 × 10^−4^) among the 35 angiotensinogen-connected DE genes (Data S10 in Supplementary Material). Similar results were obtained when analyzing connections between 116 DE placental genes and DE serum proteins in term preeclampsia (Figure S6B in Supplementary Material).

### Trophoblast Study

#### The *In Vitro* Effects of an Altered Maternal Serum Proteome on the Trophoblast

Next, we aimed to study various factors implicated in the pathogenesis of preeclampsia in trophoblast models to determine whether these may drive the observed placental transcriptomic changes. Since the results noted above suggested that maternal serum proteins can influence the placental transcriptome, we first measured the effects of maternal blood from early pregnancy on trophoblastic gene expression. We treated primary VTs during differentiation with first trimester sera from women with preterm preeclampsia or normal pregnancy, and analyzed the expression of genes included in the placental validation study. Serum from preeclamptic women compared to controls induced up-regulation of seven placental DE genes on the first and third days of trophoblast differentiation, including *FLT1* and *LEP* (Figure 6C). Among these, six genes were up-regulated in the placenta in term and three in preterm preeclampsia. These results support *in silico* findings and suggest that maternal serum factors can up-regulate *FLT1* and may induce the terminal pathway.

#### The Effects of Altered VT Differentiation

**Figure 7:**
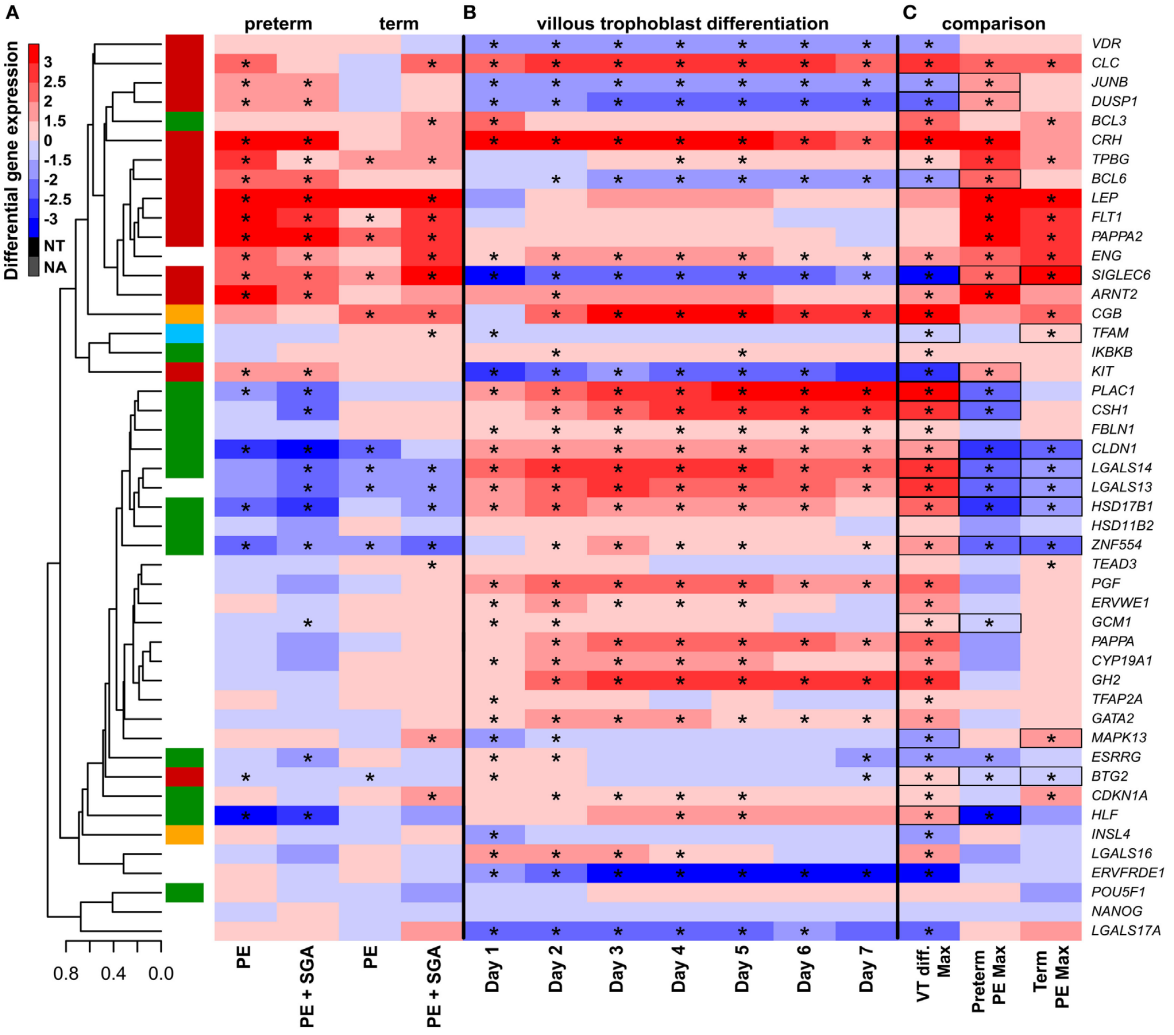
The effect of trophoblast differentiation on differentially expressed genes in preeclampsia. **(A)** Hierarchical clustering tree of expression data for 47 genes in 100 placental specimens was augmented with a heatmap representing differential gene expression in term or preterm subgroups of preeclampsia. **(B)** Primary villous trophoblast (VT) differentiation time series expression data for 47 genes were depicted with a heatmap representing differential gene expression in each time point (days 1–7) compared to day 0. **(C)** Maximum expression values in the VT differentiation time series were presented alongside with maximum expression values in the placenta in preterm or term preeclampsia. Comparative visualization revealed the opposite-direction differential regulation of 17 genes in preeclampsia compared to VT differentiation as depicted with black boxes. Among these genes, 15 had this behavior in preterm preeclampsia and 9 in term preeclampsia (*p* = 0.057). In **(A–C)**, stars depict significant changes, color bar encodes signed (up or down)-fold changes. Abbreviations: NA, not expressed; NT, not examined.

Next, we examined whether disturbance in VT differentiation may be reflected in the placental gene expression signature in women with different preeclampsia phenotypes. Since there were no (*n* = 46) or subtle (*n* = 2) gestational age-dependent differences in the expression of selected target or housekeeping genes in third trimester control placentas in our microarray study (132), the comparison of placentas and trophoblasts from various gestational ages was deemed to be valid. Thus, we performed qRT-PCR profiling of primary VTs during 7 days of differentiation to reveal the dynamics in the expression of genes (Figure 7B), which we similarly profiled in the placenta (Figure 7A). We determined whether maximum expression change of selected genes during VT differentiation compared to day 0 inversely correlated with their maximum expression change in different preeclampsia phenotypes. We found that this was the case for 17 genes (Figure 7C), suggesting the delay or inhibition of VT differentiation-related expression change of these genes in preeclampsia. Among these 17 genes, 15 showed this behavior in preterm and 9 in term preeclampsia, suggesting that VT differentiation problems are more pronounced in preterm than in term preeclampsia (*p* = 0.057).

#### The Impact of Hypoxia, Ischemia, and Overexpression of *BCL6* and *ARNT2* on the Trophoblast

**Figure 8:**
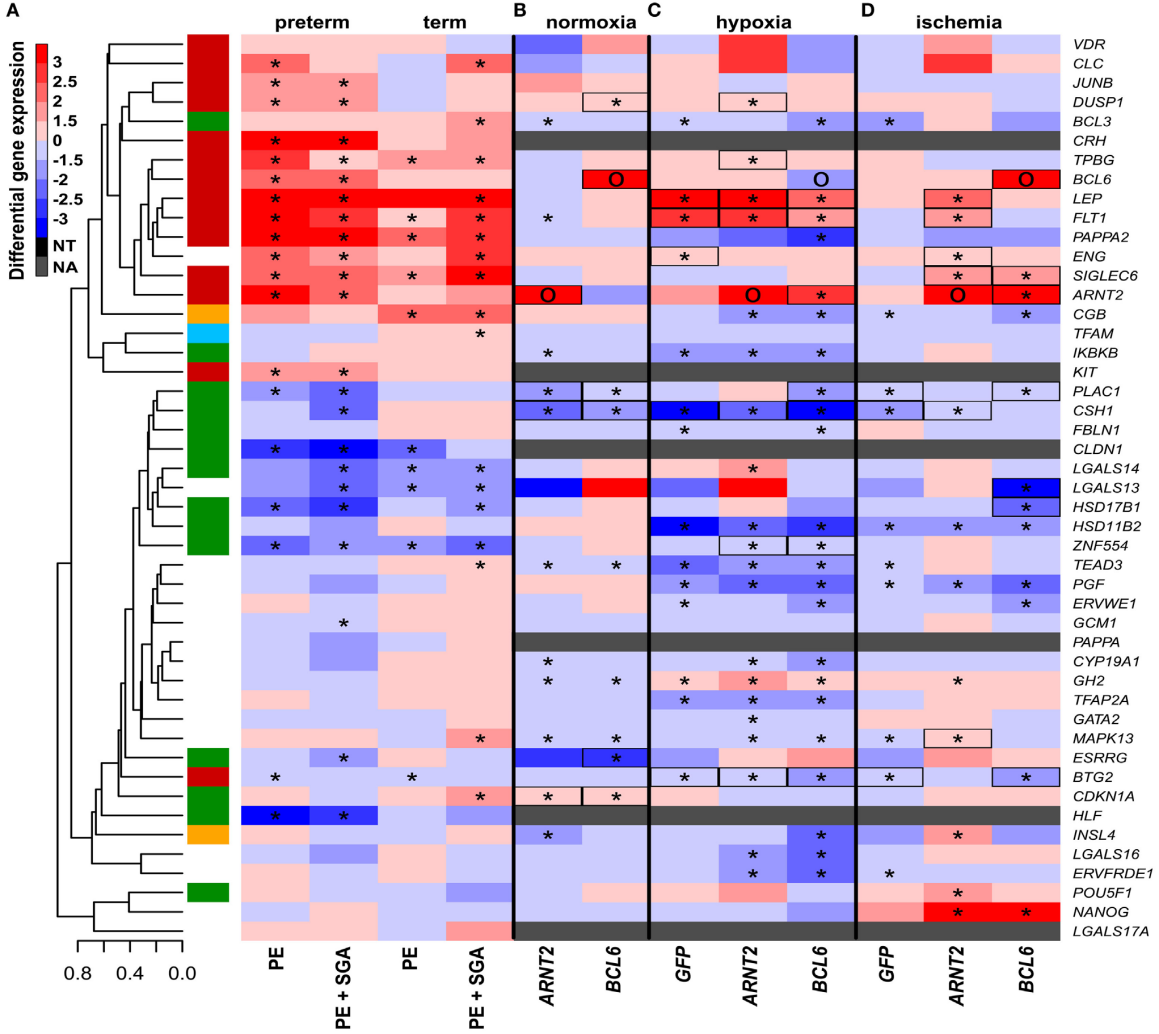
The effect of O_2_ levels, *BCL6* and *ARNT2* overexpression on differentially expressed genes in preeclampsia. **(A)** Hierarchical clustering tree and heatmap representing differential placental gene expression in preeclampsia. **(B)** The overexpression of *ARNT2* or *BCL6* in normoxic BeWo cells induced the dysregulation of three or five genes dysregulated in preeclampsia, respectively (boxed). **(C)** Hypoxia induced the dysregulation of five genes in BeWo cells also altered in preeclampsia. Hypoxia combined with *ARNT2* or *BCL6* overexpression led to the dysregulation of a large number of genes. **(D)** Ischemia induced the dysregulation of three genes in BeWo cells similar to preeclampsia. Ischemia combined with *ARNT2* or *BCL6* overexpression led to the dysregulation of 11 genes similar to preeclampsia. **(C,D)** Represents comparisons of gene expressions between hypoxia/ischemia vs. normoxia. In **(A–D)**, stars depict significant changes, “O” depicts “overexpressed,” color bar encodes signed (up or down)-fold changes. Black boxes depict genes with similar expression changes *in vitro* as in the placenta in preeclampsia. Abbreviations: NA, not expressed; NT, not examined.

Subsequently, we tested how other factors implicated in preeclampsia pathogenesis, namely physiologic hypoxia (2% O_2_) (167) or alternating hypoxic (1% O_2_) and normoxic (20% O_2_) conditions (ischemia) (40, 41, 242), in combination with the overexpression of hub transcription regulatory factors, may affect trophoblastic gene expression in a widely used BeWo cell trophoblast model. Two percent O_2_ induced the dysregulation of only five genes, including *LEP* and *FLT1*, from the set of genes investigated in the placenta in preterm preeclampsia (Figure 8C), while alternating O_2_ concentrations induced the dysregulation of only three genes (Figure 8D). Since hypoxia or ischemia alone did not induce similar transcriptomic changes in BeWo cells as seen in the placenta in preeclampsia, we examined how the overexpression of hub transcription regulatory genes in module M2 may modify the effects of these two conditions. Of note, 2% O_2_ combined with *ARNT2* or *BCL6* overexpression led to the dysregulation of a large number of genes (Figure 8C). There were 9 genes (6 in the M2 and 3 in the M1 modules) dysregulated in BeWo cells, including *FLT1, ARNT2*, and *ZNF554*, similar to preeclampsia. Alternating O_2_ concentrations combined with *ARNT2* or *BCL6* overexpression led to the dysregulation of 11 genes (5 in the M2 and 3 in the M1 modules), including *LEP, FLT1*, and *ENG*, similar to preeclampsia (Figure 8D). However, *ARNT2* or *BCL6* overexpression at normoxic conditions did not lead to substantial gene expression changes (Figure 8B), underlining the importance of gene–environment interactions. A permutation test showed that *BCL6* overexpression in ischemia mimicked overall expression changes of module M1 and M2 genes in preterm preeclampsia, while *ARNT2* overexpression in ischemia (and also in hypoxia) mimicked the up-regulation of module M2 genes in term and preterm preeclampsia (Data S11 in Supplementary Material). Since *BCL6* overexpression up-regulated *ARNT2* both in ischemia and hypoxia but not *vice versa*, we propose that *BCL6* is upstream of *ARNT2*. The up-regulation of these two transcription regulatory genes sensitize the trophoblast to ischemia, leading to the early dysregulation of modules M1 and M2, thus promoting preterm preeclampsia (Figure S7 in Supplementary Material).

Since none of the investigated conditions could up-regulate *BCL6*, we wondered whether *BCL6* overexpression might have an epigenetic background. Treating BeWo cells with 5-azacitidine down-regulated *BCL6*, supporting that its expression is regulated by DNA methylation in the trophoblast (Figure S8 in Supplementary Material). Of note, a recent study described the first intron of *BCL6* to be key in its overexpression in Burkitt lymphoma *via* altered DNA methylation (243). The Human Reference Epigenome Mapping Project revealed a differentially methylated region (DMR) in this intron in H1 embryonic stem cells as well as trophoblastic and neuronal cells derived from H1 cells, suggesting that this DMR may be differentially methylated in the trophoblast compared to other cells. To address whether this intronic region may be affected in the trophoblast in preeclampsia, we investigated DNA methylation in this region in primary VTs compared to cord blood cells collected from the same normal pregnancies. Bisulfite sequencing showed that Chr3:187,458,083-187,458,651 and Chr3:187,460,304-187,460,374 regions contain 12 hypermethylated CpGs in the trophoblasts compared to cord blood cells (Figures S8 and S9 in Supplementary Material). Further, we tested DNA methylation in this region in 100 placentas with qRT-PCR data including patients with preeclampsia after laser capturing VTs. Three CpGs (Chr3:187,458,095, Chr3:187,458,163, and Chr3:187,458,327) were differentially methylated in preeclampsia (Figure S10 in Supplementary Material), of which CpG Chr3:187,458,163 was differentially methylated in preterm preeclampsia, suggesting that this CpG may have a role in *BCL6* dysregulation in preeclampsia.

#### The Effects of *ZNF554* Down-Regulation in VTs

**Figure 9:**
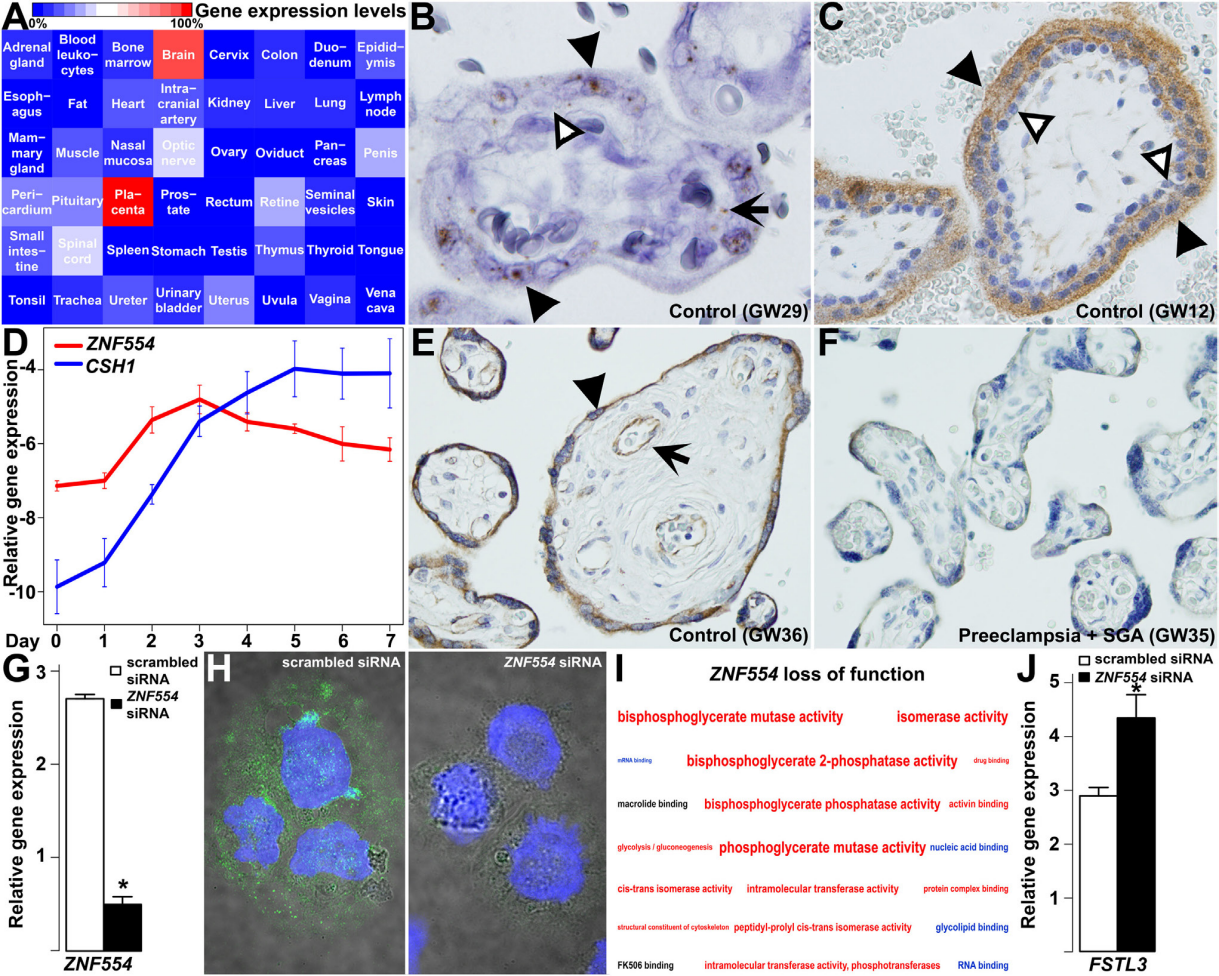
*ZNF554* down-regulation in the villous trophoblast (VT) in preeclampsia. **(A)** Tissue qRT-PCR array revealed the highest *ZNF554* expression in the placenta among 48 human tissues. Color code depicts gene expression levels relative to that of the placenta (100%). **(B)** *In situ* hybridization of a third trimester control placenta (GW29) and **(C)** immunohistochemistry of a first trimester placenta (GW12) shows mainly syncytiotrophoblastic *ZNF554* expression (hematoxylin counterstaining, 1,400 and 400× magnifications, respectively). Black or white arrowheads depict syncytiotrophoblast or cytotrophoblast, while black arrow depicts fetal endothelium, respectively. **(D)** qRT-PCR revealed that *ZNF554* expression is up-regulated during VT differentiation in parallel with *CSH1*. **(E,F)** ZNF554 immunopositivity was faint in the syncytiotrophoblast in preeclampsia [**(F)**, GW35] compared to gestational-age matched controls [**(E)**, GW36] (hematoxylin counterstaining, 400× magnifications). Arrow and arrowhead depict syncytiotrophoblast and villous endothelium, respectively. **(G)** *ZNF554* mRNA expression was 74% lower in *ZNF554*-silenced BeWo cells compared to controls used for the microarrays (*p* = 5.24 × 10^−6^). **(H)** Nuclear and cytoplasmic ZNF554 immunofluorescence decreased in BeWo cells treated with *ZNF554* siRNA compared to control cells (3,500× magnifications). **(I)** Molecular functions and one Kyoto Encyclopedia of Genes and Genomes (KEGG) pathway (glycolysis/gluconeogenesis) affected in *ZNF554*-silenced BeWo cells. Colors denote the proportions of up- or down-regulated genes (red: >0.5 up-regulated; blue:>0.5 down-regulated; black: 0.5–0.5 up- and down-regulated). Letter sizes represent the minus log_10_ of *p*-values of the given functions or pathway. **(J)** qRT-PCR validated *FSTL3* up-regulation (2.7-fold, *p* < 0.001) in BeWo cells upon *ZNF554* knock-down.

Next, we were interested in how the dysregulation of module M1 genes may play a role in preeclampsia pathology. Among hub genes of this module, *ZNF554* was of most interest due to the biological processes enriched in its co-expression network (Figure S1B in Supplementary Material), and also to its potential placenta- and preeclampsia-related regulation by transposable elements. This hypothesis was based on the fact that insertion of transposable elements into regulatory regions can lead to transcriptional changes, especially in the placenta (155, 244–247), and that the 5′ flanking region of *ZNF554* contains many LTR10A copies which had top enrichment among module M1 genes (OR = 17.4, *p* = 1.27 × 10^−7^; Data S12 in Supplementary Material). Of note, LTR10A drives placenta-specific expression of *NOS3* (248), and it may also have a similar effect on *ZNF554*. Indeed, *ZNF554* had the highest expression in the placenta in comparison to 47 other human tissues, which mostly had negligible *ZNF554* expression (Figure 9A).

Subsequent *in situ* hybridization (Figure 9B) and immunostaining (Figure 9C) of first and third trimester placentas of women with a normal pregnancy showed dominant *ZNF554* expression in the syncytiotrophoblast but not in the villous cytotrophoblast. Thus, we investigated *ZNF554* expression during VT differentiation, in which it was up-regulated similar to *CSH1* (Figure 9D), supporting that *ZNF554* expression is developmentally regulated in VTs. Of interest, ZNF554 immunostaining was faint in the syncytiotrophoblast in preeclampsia compared to controls (Figures 9E,F). To characterize the loss of syncytiotrophoblastic ZNF554 function, we silenced *ZNF554* in BeWo cells. At 74% *ZNF554* knock-down (*p* = 5.24 × 10^−6^) (Figure 9G), decreased nuclear and cytoplasmic ZNF554 immunostaining was found (Figure 9H). Microarray analyses of *ZNF554*-silenced cells revealed 123 DE genes (Data S13 in Supplementary Material) including 9 DE placental genes in preeclampsia, and the dysregulation of the “glycolysis/gluconeogenesis” pathway (OR = 7.8, *q* = 0.06) as well as 18 molecular functions including “RNA binding” (down) and “activin binding” (up) (Figure 9I; Data S14 in Supplementary Material). The up-regulation of *FSTL3* was confirmed by qRT-PCR (2.7-fold, *p* < 0.001) (Figure 9J). *FSTL3* encodes a secreted glycoprotein that inactivates activin and other TGFβ ligands (249). It is involved in the regulation of EVT invasion (250, 251) and its placental up-regulation is associated with low BW in preeclampsia (252). This finding confirms that the dysregulation of *ZNF554* may have key downstream effects on the pathogenesis of preeclampsia.

#### The Effects of *ZNF554* Down-Regulation in EVTs

**Figure 10:**
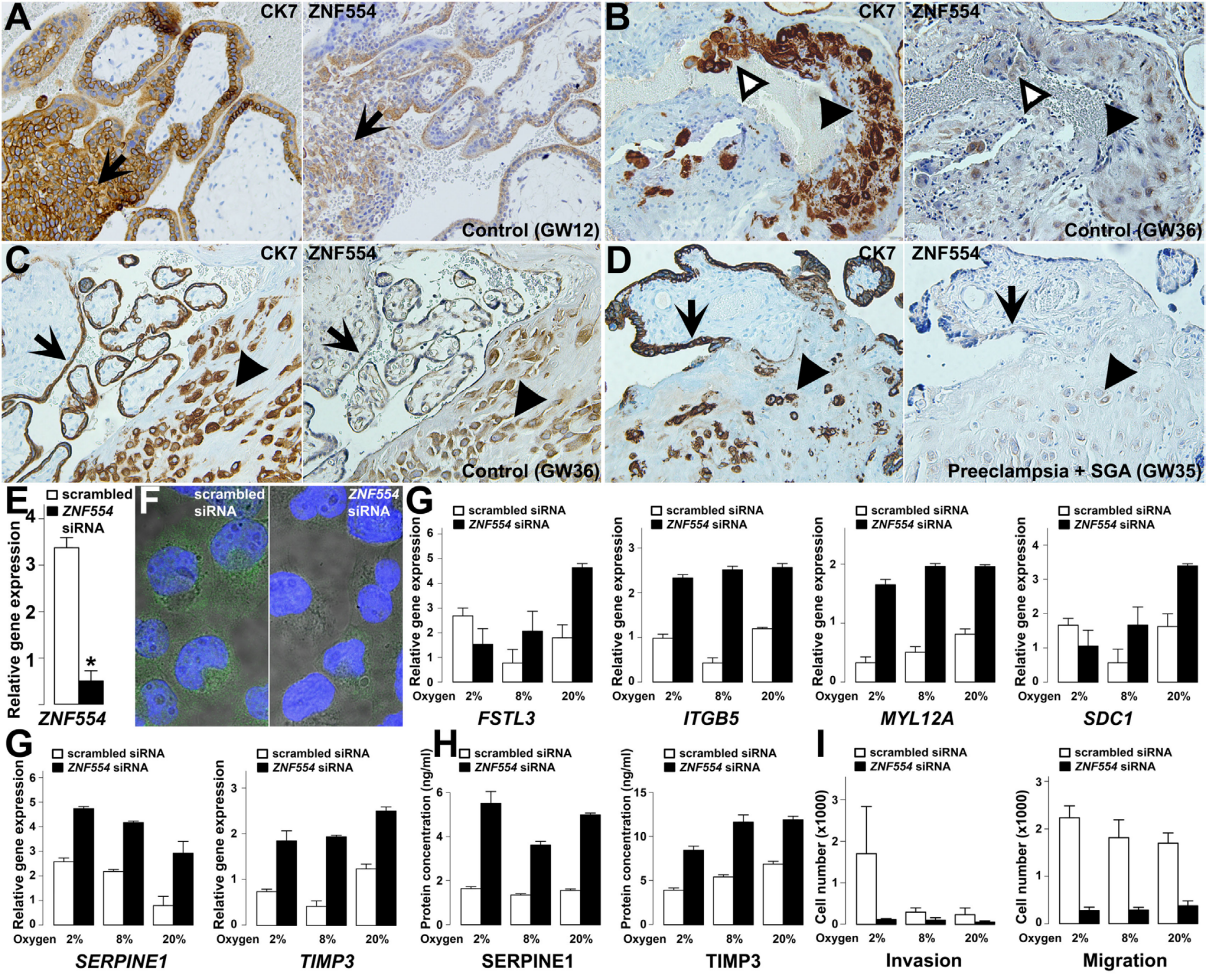
*ZNF554* down-regulation in extravillous trophoblasts (EVTs) in preeclampsia. **(A)** EVTs in trophoblastic columns and **(B)** endovascular and intraluminal trophoblasts in the myometrium were immunostained for cytokeratin-7 (left) and ZNF554 (right). Arrows depict the direction of trophoblast invasion, black and white arrowheads point to EVTs in the wall and lumen of the spiral artery, respectively (serial sections, hematoxylin counterstaining, 200× magnifications). ZNF554 immunostaining in EVTs (arrowheads) and syncytiotrophoblast (arrows) was weaker in **(D)** preeclampsia than in **(C)** controls (serial sections, hematoxylin counterstaining, 200× magnifications). **(E)** *ZNF554* mRNA expression decreased to 13% upon knock-down (*p* < 0.001), **(F)** ZNF554 immunofluorescence was weaker in the nucleus and cytoplasm of *ZNF554*-silenced (left) than of control (right) HTR8/SVneo cells (3,500× magnifications). **(G)** The dysregulation of selected genes upon *ZNF554* knock-down was confirmed by qRT-PCR. **(H)** Plasminogen activator inhibitor-1 (PAI-1 / SERPINE1) and TIMP-3 proteins were increasingly secreted from *ZNF554*-silenced cells. **(I)** *ZNF554*-silenced cells had remarkably decreased invasive (left) and migratory (right) characteristics. O_2_ concentrations are shown below the bars.

We supposed that *ZNF554* may also affect EVTs, since its expression in EVTs was detected in first and third trimester maternal decidua (Figures 10A–D), and ZNF554-positive intraluminal and endovascular trophoblasts were found in the wall of transformed spiral arteries (Figure 10B). In preterm preeclampsia, ZNF554 immunostaining of EVTs was weaker than in controls (Figures 10C,D). To characterize the loss of ZNF554 function in EVTs, we silenced *ZNF554* in trophoblastic HTR8/SVneo cells (253). At 87% knock-down (*p* < 0.001) (Figure 10E), we observed decreased nuclear and cytoplasmic ZNF554 immunostaining (Figure 10F). Microarray analysis of *ZNF554*-silenced cells showed 185 DE genes (Data S15 in Supplementary Material) including 18 DE placental genes in preeclampsia. Gene ontology (GO) analysis revealed 16 molecular functions dysregulated, including “cyclin-dependent protein kinase regulator activity,” “metalloendopeptidase inhibitor activity,” and “insulin-like growth factor binding.” The 67 enriched biological processes included “regulation of growth,” “smooth muscle cell migration,” “smooth muscle cell-matrix adhesion,” and “response to oxygen levels,” all relevant to trophoblast invasion and placental pathology of preeclampsia (Data S16 in Supplementary Material).

qRT-PCR confirmed the dysregulation of eight DE genes. Two genes (*CDKN1A, STK40*) are involved in the regulation of cell proliferation and differentiation (254, 255), and proliferation assays showed that *ZNF554* knock-down decreased cell proliferation in HTR8/SVneo cells slightly after 48 h (*−*14%, *p* = 0.02) (Figure S11 in Supplementary Material). Six genes (*FSTL3, ITGB5, MYL12A, SDC1, SERPINE1*, and *TIMP3*) encode proteins involved in cell adhesion, migration, invasion, and angiogenesis (Figure 10G). Since EVTs move through an environment with changing O_2_ levels, we used O_2_ concentrations for conditions relevant for endovascular (8%) and interstitial (2%) trophoblast invasion besides standard cell cultures (20%). The effect of *ZNF554* knock-down was significant regardless of O_2_ levels on four genes (*ITGB5, MYL12A, SERPINE1*, and *TIMP3*), while there was an interaction between O_2_ levels and *ZNF554* silencing on two genes (*FSTL3* and *SDC1*) (Figure 10G).

The up-regulation of SERPINE1 (PAI-1) and tissue inhibitor of metalloproteinases-3 (TIMP-3) was also confirmed at the protein level in supernatants of *ZNF554*-silenced cells (Figure 10H). Both proteins have an inhibitory function on trophoblast migration and invasion (250, 256, 257), and TIMP-3 is the major tissue metalloproteinase inhibitor at the maternal–fetal interface, which is up-regulated in preeclampsia (106, 258, 259). These results suggested that *ZNF554*-silenced cells have reduced migratory and invasive functions. Indeed, functional assays revealed that *ZNF554* silencing had a strong inhibitory effect on trophoblast migration (*p* = 1.9 × 10^−10^) regardless of the O_2_ concentration, and also on invasion (*p* < 0.001), especially at 2% O_2_ concentration (Figure 10I). These data corroborated that *ZNF554* supports trophoblast invasion *via* modulating a set of key genes that are involved in this process.

#### DNA Methylation-Mediated Trophoblastic *ZNF554* Down-Regulation in Preeclampsia

We wondered whether placental *ZNF554* down-regulation might have an epigenetic background, given that the *ZNF554* flanking region contains several transposable elements, including several Alus, which are generally hypomethylated in germ cells and the placenta (260, 261), while their hypermethylation leading to altered gene expression may be detected in preeclampsia (262, 263). Thus, the down-regulation of *ZNF554* expression in the placenta of patients with preeclampsia may also be reflected in the DNA methylation of the transposable elements in its 5′ flanking region.

**Figure 11:**
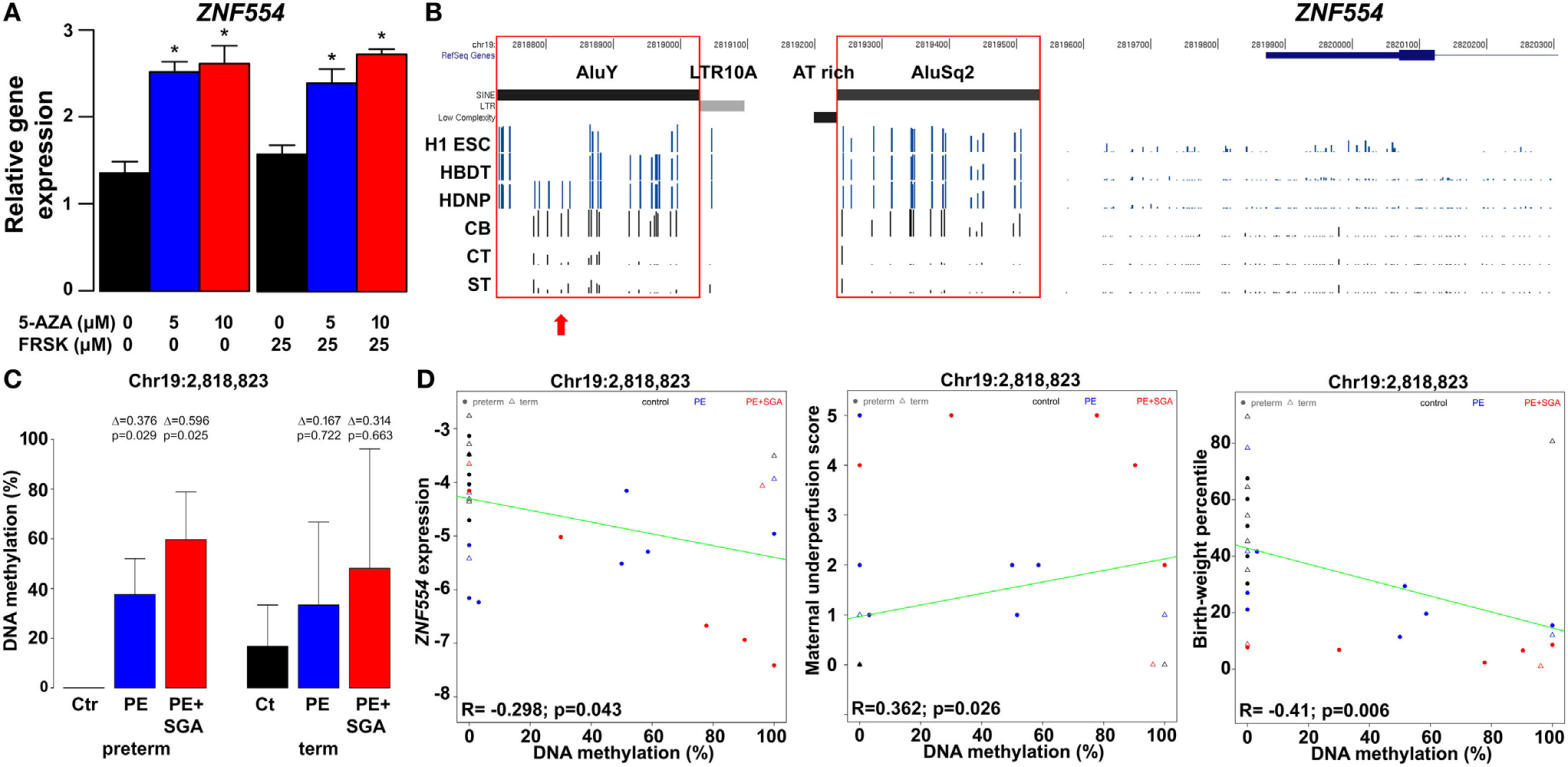
The effect of trophoblastic DNA methylation on *ZNF554* expression and clinical parameters in preeclampsia. **(A)** Increased *ZNF554* expression in BeWo cells upon treatment with 5-azacitidine (5-AZA) irrespective of forskolin (FRSK) co-treatment. **(B)** *Upper three lanes*: whole genome bisulfite sequencing data of *ZNF554* 5′ flanking region from the Human Reference Epigenome Mapping Project. H1 ESC, H1 embryonic stem cell; HBDT, H1 BMP4-derived trophoblast; and HDNP, H1-derived neuronal progenitor. *Lower three lanes*: bisulfite sequencing data from this study. Abbreviations: CB, cord blood cell; CT, cytotrophoblast; ST, syncytiotrophoblast. *Red boxes*: AluY and AluSq2 are heavily methylated in cord blood cells and hypomethylated in trophoblasts. *Red arrow*: CpG Chr19:2,818,823. **(C)** CpG Chr19:2,818,823 was differentially methylated in trophoblasts in preterm preeclampsia ± SGA. **(D)** Correlation was observed between Chr19:2,818,823 CpG methylation and *ZNF554* expression, maternal vascular malperfusion score of the placenta, and birthweight percentile.

The treatment of BeWo cells with 5-azacitidine increased *ZNF554* expression, showing the role of DNA methylation in trophoblastic *ZNF554* regulation (Figure 11A). The subsequent search in the Human Reference Epigenome Mapping Project data revealed a DMR located in the AluY, which was hypomethylated in H1 embryonic stem cells and H1-derived trophoblasts compared to H1-derived neuronal cells (Figure 11B). This was of interest, since AluY is a retrotransposon evolved recently in primates, and its differential DNA methylation supports the expression of other gene transcripts in the placenta compared to somatic tissues (264). These data prompted us to investigate the DNA methylation in this genomic region in primary VTs and cord blood cells collected from the same normal pregnancies. In fact, bisulfite sequencing showed that the AluY, similarly to the AluSq2, is heavily methylated in cord blood cells compared to the hypomethylated trophoblast, suggesting its importance in the developmental regulation of *ZNF554* expression (Figure 11B; Figure S12 in Supplementary Material).

Further, we tested DNA methylation in this region in 100 placentas with qRT-PCR data, including patients with preeclampsia after laser capture of the VTs. Bisulfite sequencing of the trophoblastic DNA revealed four CpGs on AluY (Chr19:2,818,823, Chr19:2,818,864, Chr19:2,818,868, and Chr19:2,818,876) hypomethylated in controls but hypermethylated in preterm preeclampsia, with highest methylation in cases associated with SGA (Figure 11C; Figure S13 in Supplementary Material). Importantly, we found correlations between Chr19:2,818,823 CpG methylation and *ZNF554* expression (*R* = −0.30, *p* = 0.04), maternal vascular underperfusion score of the placenta (*R* = 0.36, *p* = 0.03), and BW percentile (*R* = −0.41, *p* < 0.01) (Figure 11D). These data collectively provide evidence that the hypermethylation of certain CpGs in AluY in the trophoblast may result in low *ZNF554* expression, impaired trophoblast invasion, preeclampsia, and fetal growth restriction.

## Discussion

The placenta has a key role in the pathogenesis of the terminal pathway of preeclampsia, which may be triggered by discrete disease pathways at early stages of pregnancy, leading to the development of different preeclampsia phenotypes. In this study, an integrated systems biology approach was employed to gain insights into these complex pathways, given that this strategy offered the ultimate analytic solution to investigate and understand the complex disease pathways of the syndrome of preeclampsia (265–268). We incorporated “omics,” clinical, placental, and functional data from patients with distinct phenotypes of preeclampsia. We employed molecular network-based approaches to identify networks and modules of genes or proteins that are perturbed in the placenta and the maternal circulation of women with preeclampsia.

**Figure 12:**
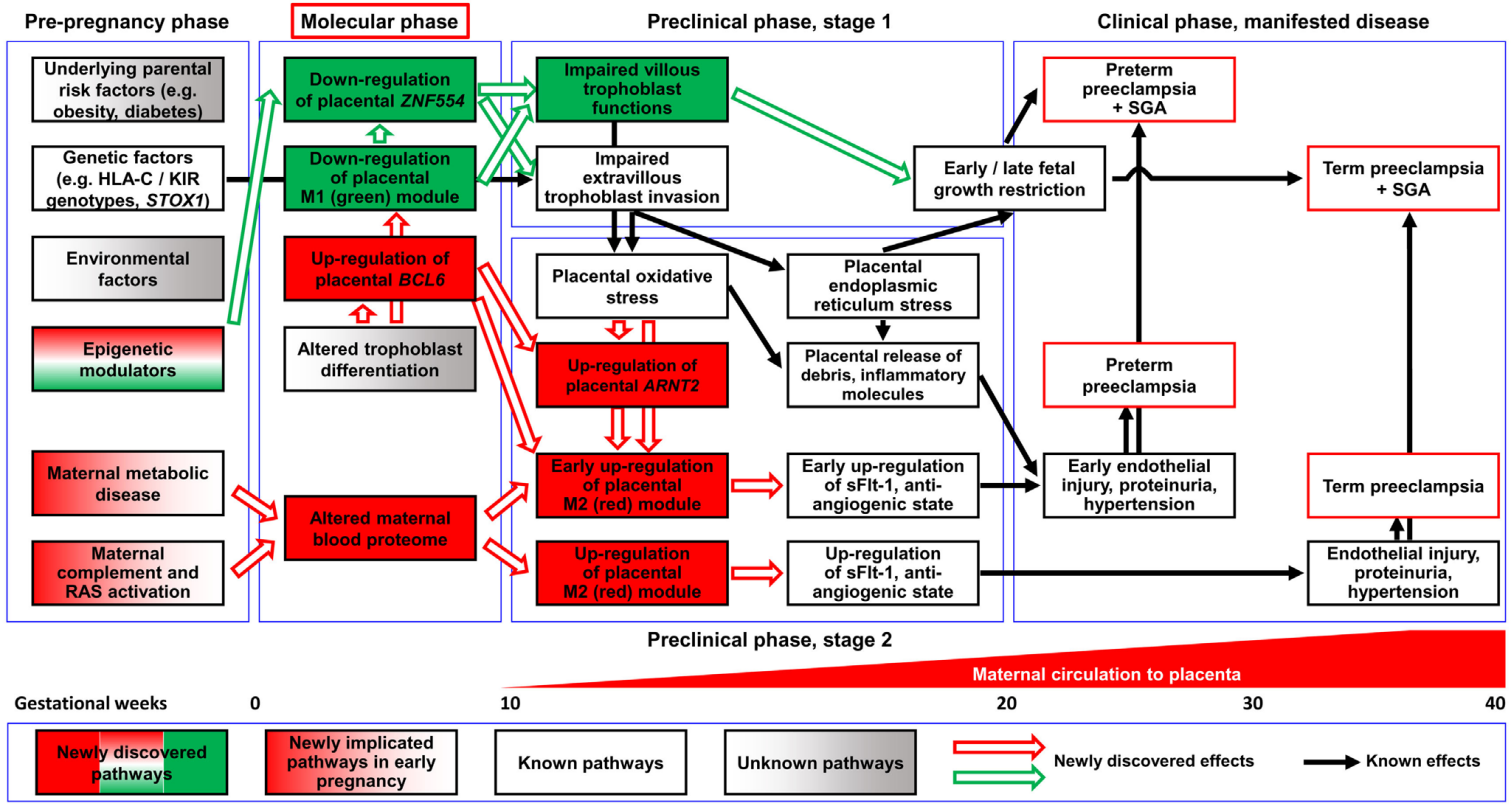
Pathologic pathways in preeclampsia. *Maternal pathways*: alterations in the maternal blood proteome, including systemic inflammatory changes, can be observed in both preterm and term preeclampsia before the maternal circulation of the placenta has been established, supporting the observation that maternal factors have a key role in triggering early disease pathways. Later, these alterations can induce trophoblastic functional changes leading to the up-regulation of module M2 genes, the overproduction of sFlt-1, and an anti-angiogenic state through a trajectory that does not necessarily affect fetal growth. *Placental pathways*: altered differentiation of the trophoblast leads to the dysregulation of module M1 genes and hub factors in module M2. The down-regulation of *ZNF554* and module M1 genes involved in the regulation of fetal growth and metabolism imply impaired villous trophoblast (VT) functions besides abnormal extravillous trophoblast (EVT) invasion. The up-regulation of the *BCL6-ARNT2* pathway sensitizes the trophoblast to ischemia and increases *FLT1* expression after the maternal circulation to the placenta has been established. These changes are observed only in preterm preeclampsia, suggesting that this placental pathway promotes the early development of preeclampsia. The interplay of these molecular pathways leads to the complex pathogenesis of preeclampsia. Abbreviations: RAS, renin-angiotensin system; SGA, small-for-gestational age.

Our placental transcriptomics study identified 1,409 DE genes involved in many biological processes (e.g. BP regulation, apoptosis, development, hormone secretion, metabolism, and signaling) that were previously implicated in the pathogenesis of preterm preeclampsia by other placental transcriptomics studies (105, 110, 111, 113, 114, 116, 117, 122, 123, 125, 127, 128, 130, 131, 134, 137, 269–272). Despite the differences in patient populations, design, or methodologies between these studies, many DE genes on our list have also been found by other groups. Indeed, from the 40-gene meta-signature that characterized the significant intersection of DE genes from independent placental gene signatures in preeclampsia in the meta-analysis of Kleinrouweler et al. (135), our microarray and qRT-PCR studies found 26 (65%) to be DE in preterm preeclampsia. Of note, 16 of these 26 genes belong to module M2, while only six to the M1 module. This supports our observation that the dysregulation of module M2 is associated with BP elevation, the maternal disease condition required for patient inclusion into these studies. The weaker involvement of module M1 genes in the meta-signature may reflect the heterogeneity of preeclampsia transcriptomics studies regarding fetal (growth restriction) and placental disease conditions. Our microarray study was homogeneous for preterm preeclampsia cases with low BW and placental disease, while only a couple of other studies had this rigor. To overcome the inconsistency of smaller placental transcriptomics studies, Leavey et al. (140, 142) employed advanced bioinformatics methods to aggregate microarray datasets across multiple platforms to generate large datasets of patient samples. Unsupervised clustering of these datasets revealed three distinct molecular subclasses of preeclampsia. Among these, the “canonical” subclass, which is associated with the differential expression of our module M2 genes, was characteristic for preterm preeclampsia and consistent with stress response to poor oxygenation, further supporting our findings. However, our discovery on the two major dysregulated placental disease gene modules and their hub transcription regulatory genes, separately associated with maternal or fetal disease pathways, are novel (Figure 12).

Functional assays on hub transcription factors of these two disease gene modules demonstrated that *ZNF554* (M1) down-regulation leads to impaired trophoblast invasion, while *BCL6* and *ARNT2* (M2) overexpression sensitizes the trophoblast to ischemia, which are hallmarks in the pathogenesis of preterm preeclampsia. In the “ZNF554” pathway, hypermethylation of AluY in the *ZNF554 5*′ flanking region inhibits gene expression, leading to impaired trophoblast invasion, placental vascular malperfusion, and low BW. In the “BCL6-ARNT2” pathway, which is activated only in preterm preeclampsia, ischemic stress of the trophoblast coupled with *BCL6* and *ARNT2* overexpression increases *FLT1* expression. This then eventually promotes the anti-angiogenic state, hypertension (37, 56, 57, 61, 62, 83), and the early onset of this syndrome (Figure S7 in Supplementary Material).

The perturbed placental disease gene modules in preterm preeclampsia can be detected by liquid biopsy in maternal blood in the early stages of pregnancy. Indeed, we detected the down-regulation of M1 and M2 disease gene module biomarkers in these patients during the first trimester. Of interest, the up-regulation of module M2 biomarkers can be detected after the establishment of maternal circulation in the intervillous space. These findings support the observation that placental transcriptomic changes, typical for preterm preeclampsia observed in the third trimester, are rooted in the first trimester. The positive correlation of gene expression in module M1 with BW and in module M2 with BP suggests that M1 genes may be biomarkers for placental and fetal growth and development while M2 genes can serve as biomarkers for placental stress.

First trimester maternal blood proteomics uncovered the altered abundance of proteins of the renin–angiotensin and immune systems as well as complement and coagulation cascades in patients who subsequently developed both preterm and term preeclampsia. The same proteins and pathways were found to be dysregulated in maternal blood in later stages of preeclampsia by other proteomics studies (108, 115, 118–120, 126, 129, 133, 136, 138, 139, 143); however, ours is the first revealing their dysregulation at an earlier stage of pregnancy when there is no or minimal direct connection between the placenta and the maternal circulation (273).

From these dysregulated maternal serum proteins, *in silico* analysis pointed to candidates, which may drive trophoblastic transcriptomic changes, and corroborated earlier findings on angiotensinogen/angiotensin II in driving hypertension indirectly through *FLT1* up-regulation in addition to its direct effects (91, 274). Moreover, *in vitro* functional assays revealed that altered maternal serum proteome in the first trimester can affect the trophoblastic transcriptome and up-regulate *FLT1*. This is in agreement with reports indicating that maternal blood factors in preeclampsia can induce trophoblastic soluble endoglin overexpression and the development of preeclampsia-like symptoms in mice (85, 275).

Remarkably, most of the dysregulated maternal serum proteins in the first trimester in both preterm (11 of 19) and term (7 of 14) preeclampsia are implicated in immune functions (Figure 6; Figure S6 in Supplementary Material), suggesting a critical role for immune pathways and inflammation in the early pathogenesis of both phenotypes of preeclampsia. This is consistent with clinical, epidemiological, and immunological evidence showing that: (1) preeclampsia has multiple risk factors (e.g. dyslipidemia, hypertension, insulin resistance, and obesity) characterized by heightened inflammation (29, 276); (2) the combination of inhibitory decidual NK (dNK) cell killer immunoglobulin-like receptor and the fetal HLA-C2 genotype increases the susceptibility to preeclampsia due to the loss of activating interactions between trophoblasts and dNK cells at early stages of placentation (168, 277–283); (3) an altered local immune regulation and a shift toward the pro-inflammatory macrophage phenotype promotes a pro-inflammatory milieu in the maternal decidua in preeclampsia (284, 285); (4) an imbalance between Th1/Th2/Th17/Treg cells in preeclampsia leads to failure of maternal–fetal tolerance mechanisms (286–289); and (5) complement system activation in preeclampsia leads to the activation of innate immune cells and placental damage (101, 234, 236, 237, 290–292). The role of inflammation in early preeclampsia disease pathways is also supported by *in vivo* studies showing that bacterial endotoxin administration to pregnant rats induces placentation defects and symptoms consistent with preeclampsia (293–298).

Overall, our data show that there are distinct maternal and placental disease pathways, and their interaction influences the clinical presentation of preeclampsia. The activation of maternal disease pathways can be detected in both preterm and term preeclampsia earlier and upstream of placental dysfunction, not only downstream as described before (43), and distinct placental disease pathways are superimposed on these maternal pathways. This is a paradigm shift in our understanding of preeclampsia, which in agreement with epidemiological studies (25, 31) warrants for the central pathologic role of preexisting maternal diseases or perturbed maternal–fetal–placental immune interactions in preeclampsia.

The superimposed placental disease pathways differ between preterm and term preeclampsia. For preterm preeclampsia, our functional data suggest that placental disease pathways are partly originated from altered trophoblast differentiation, which is followed by trophoblastic stress, induced by perturbed maternal blood proteome factors and/or ischemia after the onset of maternal circulation to the placenta. Our data are consistent with recent views indicating that defects in trophoblast proliferation, differentiation, invasion, and plugging are associated with defective decidualization (299), decidual inflammation (300), and the disturbance in endometrial-trophoblast dialog during the peri-conception (301) period. Abnormal trophoblast invasion and plugging will subsequently lead to the aberrant onset of maternal circulation (273) and malperfusion, causing placental oxidative stress in preterm preeclampsia (175, 273, 302). On the other hand, maternal disease pathways induce mainly placental dysfunction without maldevelopment in term preeclampsia. This is substantiated by the differences observed in the maternal proteome, placental transcriptome, and trophoblastic DNA methylation between term and preterm preeclampsia in our study. Moreover, this is also consistent with the major differences between these two preeclampsia phenotypes in etiology (31), placental histopathology (28, 33, 44–46), and stress levels (303) as well as clinical presentation (2, 25, 26, 29, 304).

Our findings are very timely in the light of recent clinical research showing that the administration of aspirin before 16 weeks of gestation to pregnant women at risk for preeclampsia prevents the preterm phenotype of this syndrome (24, 305–309). Thus, the anti-inflammatory and anti-platelet actions of aspirin (308–311) may ameliorate the early pro-inflammatory disease pathways leading to placental maldevelopment in preterm preeclampsia. Based on our discovery of these novel disease pathways and their hub molecules, we propose a “molecular phase” of preeclampsia (Figure 12), where early pathologic events can already be detected by maternal blood biomarkers, offering non-invasive diagnostics of maternal and placental disease pathways. Biomarkers of these disease pathways may open new venues for the molecular characterization of patients destined to develop preeclampsia, using multi-biomarker profiles that support preventive approaches for patients with distinct preeclampsia phenotypes.

## Materials and Methods

### Placental Study

#### Placental Microarray Study

##### Study Groups and Clinical Definitions

Placental tissue and maternal blood samples were collected from Caucasian women at the First Department of Obstetrics and Gynaecology, Semmelweis University in Budapest, Hungary as described previously (132). Pregnancies were dated according to ultrasound scans between 8 and 12 weeks of gestation. Patients with multiple pregnancies or fetuses having congenital or chromosomal abnormalities were excluded. The collection and investigation of human clinical samples were approved by the Health Science Board of Hungary. Written informed consent was obtained from women prior to sample collection, and the experiments conformed to the principles set out in the World Medical Association Declaration of Helsinki and the Department of Health and Human Services Belmont Report. Specimens and data were stored anonymously.

Women were enrolled in the following groups: (1) preterm severe preeclampsia, with or without HELLP (hemolysis, elevated liver enzymes, low platelets) syndrome (*n* = 12) and (2) preterm controls (n = 5) (Table 1). Preeclampsia was defined according to the criteria set by the American College of Obstetricians and Gynecologists (1), and subdivided into preterm (<37 weeks) or term (≥37 weeks) groups. Severe preeclampsia was defined according to Sibai et al. (2). Preterm controls had no medical complications, clinical or histological signs of chorioamnionitis, and delivered neonates with a BW appropriate-for-gestational age (AGA) (312). SGA was defined as neonatal BW below the 10th percentile for gestational age. Cesarean delivery was performed in all cases due to severe symptoms as well as in all controls due to previous Cesarean delivery or malpresentation.

##### Placental Tissue and Maternal Blood Collection

Placental tissue specimens were processed immediately after delivery as described previously (132). For the microarray study, 1 × 1 cm villous tissue samples were excised from central cotyledons close to the umbilical cord to reduce the possible bias due to regional differences in gene expression (313, 314). These tissue blocks were then dissected from the choriodecidua on dry ice, snap-frozen, and stored at −80^o^C. For histopathologic evaluations, five representative tissue blocks were taken from each placenta to include central and peripheral cotyledons and the maternal side of the placenta with the fetal membranes. These blocks were embedded in paraffin after fixation in 10% neutral-buffered formalin (FFPE). Maternal blood samples were obtained at the time of admission into the delivery room; aliquots of maternal sera and plasma were stored at −80^o^C.

##### Histopathologic Evaluation of the Placentas

Placental specimens were examined according to a standard protocol, describing the topography and size of macroscopic lesions. Four micrometer sections were cut from the five FFPE blocks and mounted on SuperFrost/Plus slides (Fisherbrand, UK). After deparaffinization, slides were rehydrated, stained with hematoxylin and eosin, and examined in 10 randomly chosen microscopic fields using bright-field light microscopy by a pathologist blinded to the clinical information. Macroscopic and microscopic lesions were defined according to published criteria (315–317).

##### Placental Total RNA Isolation and Microarray Experiments

Tissues were homogenized using a ThermoSavant FastPrep FP120 Homogenizer (Thermo Scientific, Wilmington, DE, USA) with Lysing MatrixD (MP Biomedicals, Illkirch, France). Total RNA was isolated using RNeasy Fibrous Tissue Mini Kit (QIAGEN GmbH, Hilden, Germany), quantified with NanoDrop 1000 (Thermo Scientific), and assessed by Agilent 2100 Bioanalyzer (Matriks AS, Oslo, Norway). Total RNAs were labeled, and Cy3-RNAs were fragmented and hybridized to the Whole Human Genome Oligo Microarray G4112A (Agilent Technologies, Santa Clara, CA, USA) on an Agilent scanner, and processed with Agilent Feature Extraction software v9.5 according to the manufacturer’s guidelines.

##### Data Analysis

Demographics data were compared by the Fisher’s exact test and Mann–Whitney test using SPSS version 12.0 (SPSS Inc., Chicago, IL, USA). Microarray data analysis was performed using the R statistical language and environment^1^ following the MIAME guidelines and methodologies described previously (318). Microarray expression intensities were background corrected using the “minimum” method in the “backgroundCorrect” function of the “limma” package (319). After log_2_ transformation, data were quantile-normalized. From the 41,093 probesets on the array, 93 were removed before differential expression analysis, lacking annotation in the array definition file (Agilent Technologies). Subsequently, an expression filter was applied to retain probesets with intensity greater than log_2_(50) in at least two samples, yielding a final matrix of 30,027 probesets (15,939 unique genes). Differential gene expression was assessed using linear models including adjustment for batch effects, with coefficient evaluation *via* moderated *t*-tests. *P*-values were adjusted using the false discovery rate (FDR) method and Benjamini–Hochberg correction to compute *q*-values. Since there were no differences among patient groups in maternal age or ethnic background, we did not adjust for these parameters. Target gene Entrez IDs for the probesets were determined using the R package “hgu4112a.db.” For probesets without annotation in the package, Entrez IDs were taken from the array definition file (Agilent Technologies). Probesets remaining un-annotated (without Entrez ID and/or gene symbol) were removed from further analysis. Probesets were defined as DE (*n* = 1,409) if they had a *q* ≤ 0.2 and a fold-change ≥1.5 (Data S1 in Supplementary Material).

From the DE genes in preeclampsia, those encoding for proteins with functions in transcription regulation (*n* = 137) were identified using the Metacore (GeneGo Inc., Saint Joseph, MI, USA) and GeneCards v3^2^ databases.

We downloaded the human U133A/GNF1H microarray data on 79 human tissues, cells, and cell lines from the Symatlas/BioGPS database (151) to identify human genes with predominant placental expression. A probeset was defined as having predominant placental expression if its placental expression was (1) ≥1,000 fluorescence units; (2) six times higher than the median value in 78 other tissue and cell sources; and (3) two times higher than its expression in the tissue with the second highest expression. The resulting 215 probesets corresponded to 153 unique genes. Eleven additional genes that were not present on the microarray platform used by Symatlas/BioGPS (Affymetrix, Santa Clara, CA, USA) were added to this list based on previously published evidence of placenta-specific expression (151–153) (Data S2 in Supplementary Material). Of the 164 predominantly placenta-expressed genes, 157 were present in our placental microarray data. These genes were tested for enrichment in DE genes compared to all genes on the array (1,409 of 15,939) using the Fisher’s exact test.

The expression levels of DE genes in EVT compared to VT lineages were analyzed by retrieving published microarray datasets (320–322) and reanalyzing expression data. Raw Affymetrix GeneChip Human Genome U133A 2.0 Array data from Bilban et al. (320) and Tilburgs et al. (322) was downloaded from GEO (GSE9773) and ArrayExpress (E-MATB-3217) respectively, and was processed with the *“affy”* (323) and *“limma”* (324) packages of Bioconductor.^3^ After RMA normalization, log-fold changes were calculated. Processed Illumina Human HT-12 V3 BeadArray data from Apps et al. (321) was downloaded from ArrayExpress (E-MATB-429), and then log-fold changes were calculated.

Chromosomal locations for all genes tested on the Agilent array were obtained from the R package “org.Hs.eg.db.” Of the 15,939 unique and 1,409 DE genes on the array, 15,935 and 1,408 could be assigned to chromosomes, respectively. Mapping the microarray probesets on the Affymetrix human U133A/GNF1H chips to ENTREZ identifiers was performed using the Bioconductor “hgu133a.db” and “hgfocus.db” packages (325, 326). Chromosomal locations of the resulting list of genes were obtained from the package “org.Hs.eg.db” and from the National Center for Biotechnology Information for the 11 additional genes (327). Enrichment analyses for chromosomes among predominantly placenta-expressed genes, DE genes and DE genes encoding for transcriptional regulators (Data S3–S5 in Supplementary Material) were tested by the Fisher’s exact test. Chromosomal locations of these genes were visualized by Circos (328).

Weighted gene co-expression network analysis (329, 330) was applied on the 1,409 DE genes across 17 samples to identify distinct regulatory modules and prioritize candidate genes for qRT-PCR validation. A gene pair-wise similarity (absolute Pearson correlation) matrix was first computed, then soft-thresholded by raising it to the power of 10 (chosen based on the scale-free topology criterion) to obtain an adjacency matrix. The topology overlap matrix (TOM) was then derived from the adjacency matrix. The topology overlap (331) measures the node interconnectedness within a network and was generalized to WGCNA (329). This measure defines the similarity between the two genes based on both correlations within themselves and outside other genes. Gene distance matrix was defined as 1-TOM and used for average linkage hierarchical clustering. A hybrid dynamic tree-cutting method (332) was applied to obtain modules (tree clusters).

Gene modules identified with this approach were further tested for enrichment in DE genes using the Fisher’s exact test. Transcription regulatory genes expressed at high levels (average log_2_ intensity >9) and co-expressed (absolute Pearson coefficient >0.7) with the most genes among DE genes were treated as candidates for hub genes in the module. Hub genes then were selected based on the number and strength of their Pearson co-expression partners as well as their and their networks’ biological activities. The networks of biological processes enriched among genes co-expressed with hub factors modules were created by BINGO and visualized with Cytoscape.

Enrichment analysis of transposable elements present in the 10,000 bp upstream region of DE genes was performed separately for the M1 (green) and the M2 (red) modules in preeclampsia versus all genes present on the microarray using the Fisher’s exact test. The locations of transposable elements and their families and classes were obtained from the “RepeatMasker” table of the UCSC Table Browser.^4^ *P*-values <0.05 were considered significant.

#### Placental Validation Study

##### Study Groups and Clinical Definitions

Third trimester placentas (*n* = 100) collected predominantly from African-American women were retrieved from the Bank of Biological Specimens of the Perinatology Research Branch (Detroit, MI, USA). Pregnancies were dated according to ultrasound scans between 8 and 12 weeks. Patients with multiple pregnancies or fetuses having congenital or chromosomal abnormalities were excluded. The use of biological specimens and clinical data for research purposes was approved by the Wayne State University Human Investigation Committee and the Institutional Review Board of the *Eunice Kennedy Shriver* National Institute of Child Health and Human Development (NICHD, NIH, DHHS). Written informed consent was obtained from women prior to sample collection, and the experiments conformed to the principles set out in the World Medical Association Declaration of Helsinki and the Department of Health and Human Services Belmont Report. Specimens and data were stored anonymously.

The following homogenous patient groups were selected from a cohort: (1) preterm severe preeclampsia (*n* = 20); (2) preterm severe preeclampsia associated with SGA (*n* = 20); (3) preterm controls (*n* = 20); (4) term severe preeclampsia (*n* = 10); (5) term severe preeclampsia associated with SGA (*n* = 10); and (6) term controls (*n* = 20). Term controls, consisting of normal pregnant women with (*n* = 10) or without (*n* = 10) labor, and preterm controls with preterm labor and delivery had no medical complications or clinical or histological signs of chorioamnionitis, and delivered an AGA neonate (333) (Table 2; Figure S2 in Supplementary Material).

Labor was defined by the presence of regular uterine contractions at a frequency of at least two contractions every 10 min with cervical changes resulting in delivery (334). Preeclampsia was defined according to the criteria set by the American College of Obstetricians and Gynecologists (1) and was subdivided into preterm (<37 weeks) or term (≥37 weeks) groups. Severe preeclampsia was defined according to Sibai et al. (2). SGA was defined as neonatal BW below the 10th percentile for gestational age (333). Cesarean delivery was performed in cases due to severe symptoms and in controls due to previous Cesarean delivery or malpresentation.

##### Placental Tissue Collection

Third trimester placental tissue specimens (*n* = 100) were processed immediately after delivery. For histopathologic evaluations, representative tissue blocks were taken from each placenta to include central and peripheral cotyledons, maternal side of the placenta, fetal membranes, and the umbilical cord. These blocks were embedded in paraffin after fixation in 10% neutral-buffered formalin (FFPE). Villous tissue blocks from central cotyledons were selected for TMA. For gene expression studies, systematic random sampling (335) was used to obtain villous tissues to reduce the possible bias due to regional gene expression differences (313, 314). Excised tissue blocks were homogenized and mixed in TRIzol reagent (Life Technologies), snap-frozen with liquid N_2_, and stored at −80^o^C.

##### Histopathologic Evaluation of the Placentas

Placental specimens (*n* = 100) were examined according to a standard protocol, describing the topography and size of macroscopic lesions. Five µm sections were cut from the representative FFPE tissue blocks, stained with hematoxylin and eosin, and examined using bright-field light microscopy by two anatomic pathologists blinded to the clinical information. Histopathologic changes of the placenta were defined according to published criteria proposed by the Perinatal Section of the Society for Pediatric Pathology (336). “Maternal vascular malperfusion score” was calculated by summing the number of pathologic lesions consistent with this lesion category (46, 336) present in a given placenta.

##### Placental Total RNA Isolation and qRT-PCR

Total RNA was isolated from snap-frozen third trimester placental villous tissues (*n* = 100) with TRIzol reagent (Life Technologies) and RNeasy kit (QIAGEN, Valencia, CA, USA) according to the manufacturers’ recommendations. The 28S/18S ratios and the RNA integrity numbers were assessed using an Agilent Bioanalyzer 2100, and RNA concentrations were measured with NanoDrop 1000. Total RNA (500 ng) was reverse transcribed with the High Capacity cDNA Reverse Transcription Kit using random hexamers (Applied Biosystems, Foster City, CA, USA). TaqMan Assays (Applied Biosystems; Table 3, Data S17 in Supplementary Material) were used for high-throughput gene expression profiling on the Biomark qRT-PCR system (Fluidigm, San Francisco, CA, USA) according to the manufacturers’ instructions.

##### Placental TMA Construction, Immunohistochemistry, and Immunoscoring

Tissue microarrays were constructed from central tissue blocks of third trimester FFPE placentas (*n* = 100) as described earlier (337). Briefly, three 20 × 35 mm recipient blocks were made of Paraplast X-Tra tissue embedding media (Fisher Scientific, Pittsburgh, PA, USA). One mm diameter cores from tissue blocks were transferred in triplicate into recipient paraffin blocks using an automated tissue arrayer (Beecher Instruments, Inc., Silver Spring, MD, USA). Five µm sections cut from TMAs were placed on silanized slides and stained with antibodies and reagents (Data S18 in Supplementary Material) either on Ventana Discovery or Leica BOND-MAX autostainers.

Tissue microarray immunostainings were semi-quantitatively scored by three examiners blinded to the clinical information with an immunoreactive score modified from a previously published one (154). Immunostaining intensity was graded as follows: 0 = negative, 1 = weak, 2 = intermediate, and 3 = strong. All villi in a random field of each of the three cores were evaluated by all examiners, and scores within each core were averaged to represent the target protein quantity of that core. Thus, each placenta had three scores corresponding to the three cores examined.

##### Data Analysis

Demographics data were compared by the Fisher’s exact test and Mann–Whitney test using SPSS version 12.0 (SPSS). All other data were analyzed in the R statistical environment (see text footnote 1).

###### Placental qRT-PCR

Data were analyzed using the ΔΔCt method. Data were first normalized to the reference gene (*RPLP0*), and the batch effect was adjusted through calibrator samples. Log_2_ mRNA relative concentrations were obtained for each sample as −ΔCt_(gene)_ = Ct_(RPLPO)_ − Ct_(gene)_. The surrogate gene expression values (−ΔCt_gene_) were used to perform a hierarchical clustering with 1-Pearson correlation distance and average linkage. Between-group comparisons (in which groups were predefined based on the clinical characteristics of the patients) were performed by fitting a linear model on −ΔCt values, using the group variable indicator and the maturity status of the fetus (term vs. preterm) as covariates while allowing for an interaction effect (Figure 4; Figure S3 in Supplementary Material).

###### Histopathology

The association between qRT-PCR gene expression and the “maternal vascular malperfusion” score was tested using a linear model. *P*-values of <0.05 were considered significant.

###### TMA Immunoscoring

Group comparisons using immunoscores were conducted in the same way as for the qRT-PCR data (Figure 4).

#### Correlation of Clinical Parameters and Placental Gene Expression

##### Data Analysis

To reveal whether the expression of any gene on the microarray was correlated with MAP while controlling for BW, a linear model (*y*~MAP + BW + Batch) was fit for every gene on the array, in which *y* represents gene expression, and the dependent variables represent MAP, BW, and batch, respectively. A moderated *t*-test was used to obtain *p*-values, which then were adjusted using the FDR method and Benjamini–Hochberg correction for multiple testing. Significance was determined using a *q* ≤ 0.2. Gene modules were also tested for the enrichment in genes with their expression correlated with MAP using the Fisher’s exact test (Data S6 in Supplementary Material).

In the qRT-PCR validation study, we extended our analysis to include all 100 patients to test for the association between gene expression and mean arterial BP as well as BW percentile while adjusting for gestational age. All variables were treated as continuous. *P*-values of <0.05 were considered significant (Figure 4).

#### Genomic DNA Methylation Analysis of the Trophoblast

##### Laser Capture Microdissection

Fifteen µm sections were cut from snap-frozen placentas that were also used for qRT-PCR expression profiling (*n* = 100) on 2 µm Glass Foiled PEN slides (Leica Microsystems). The trophoblast layer of 300–350 villi in each specimen was laser captured by a Leica DM6000B microscope (Leica Microsystems) into 0.5 ml microcentrifuge tubes. The captured material was digested with Proteinase K (PicoPure DNA Extraction Kit, Applied Biosystems) at 56°C by overnight incubation. Digestions were stopped at 95°C, and samples were stored at −70°C until DNA isolation.

##### Genomic DNA Isolation

Genomic DNA was isolated from laser captured VTs (*n* = 100), from primary VTs (*n* = 3) collected for functional studies described below, and from respective umbilical cord blood leukocytes (*n* = 3) taken from the same pregnancies. The EZ1 Advanced Nucleic Acid Isolation System using EZ1 DNA Tissue and EZ1 DNA Blood Kits (QIAGEN) were utilized for DNA isolation, and DNA samples were quantified with Quantifiler Human DNA Quantification Kit (Applied Biosystems) according to the manufacturers’ instructions.

##### Primer Design and Validation

The methylation status of CpGs in human H1 embryonic stem cells, H1-derived trophoblast cultured cells, and H1-derived neuronal progenitor cultured cells obtained by whole-genome shotgun bisulfite sequencing (University of California, San Diego, USA; UCSD Human Reference Epigenome Mapping Project) were visualized by the Epigenome Browser^5^ and used for the selection of regions of interest. Primer design, targeted amplification, and sequencing were conducted using the targeted sequencing service protocol of Zymo Research Corporation. For targeted bisulfite sequencing, 30 primer pairs were designed and validated. Primers were synthesized by Integrated DNA Technologies, Inc., (Coralville, IA, USA) and underwent quality control, which included duplicate testing for specific amplification of 1 ng bisulfite DNA using bisulfite converted human DNA. Quality control criteria included robust and specific amplification (Cp values <40 cycles and CV <10% for duplicates) of the bisulfite primers on a LightCycler 480 real-time qRT-PCR instrument (Roche Diagnostics Corp. Indianapolis, IN, USA).

##### Bisulfite Conversion, Multiplex Amplification, Bar-Coding and Adapterization PCR, and Next-Generation Sequencing

Genomic DNA samples from laser captured VTs, primary VTs, and umbilical cord blood cells as well as control samples were subjected to sodium bisulfite treatment using the EZ DNA Methylation-Direct Kit (Zymo Research Corporation). For non-methylated control, human DNA was extracted and purified with Quick-gDNA Miniprep Kit (Zymo Research Corporation) from the HCT116 cell line (American Type Culture Collection, Manassas, VA, USA), which is double knock-out for both DNA methyltransferases DNMT1 (−/−) and DNMT3b (−/−), and thus contains a low level (<5%) of DNA methylation. For methylated control, human DNA was purified similarly from the HCT116 cell line and was enzymatically methylated at all cytosine positions comprising CG dinucleotides by CpG Methylase (Zymo Research Corporation).

Bisulfite-treated samples and 30 validated primer pairs were subjected to targeted amplification on the 48.48 Access Array System (Fluidigm), using the targeted sequencing service protocol of Zymo Research Corporation. Fluidigm’s protocols were used for sample loading, harvesting, and pooling, 1:100 dilution of amplicon pools for each sample, and for amplification using barcoded adapter-linkers (Fluidigm). Reactions were cleaned up using the DNA Clean and Concentrator-5 (Zymo Research Corporation), and products were normalized by concentration and pooled. The sequencing library was denatured, diluted, and sequenced with a 150-base paired-end run on the MiSeq Benchtop Sequencer (Illumina) according to Illumina’s protocols.

##### Sequence Alignment and Data Analysis

Sequence reads from bisulfite-treated libraries were identified using standard Illumina base-calling software, and then analyzed using a Zymo Research Corporation proprietary analysis pipeline. Residual cytosines (Cs) in each read were first converted to thymines (Ts), with each such conversion noted for subsequent analysis. Reads were aligned by Bismark, a Bowtie-based alignment tool for bisulfite converted reads.^6^ The number of mismatches in the induced alignment was then counted between the unconverted read and reference, ignoring cases in which a T in the unconverted read was matched to a C in the unconverted reference. For a given read, only the best scored alignment was kept. If there were more than one best read, then only one was kept arbitrarily. The methylation level of each sampled cytosine was estimated as the number of reads reporting a C, divided by the total number of reads reporting a C or T. CpGs with coverage of less than four reads were removed from the analysis. The developed sensitive and robust bisulfite sequencing assays yielded a median total sequencing read of 533 (range: 30–1,725) per CpG in the trophoblast-fetal blood cell comparison and a median total sequencing read of 136 (range: 4–2,609) per CpG in the clinical sample comparison.

Multiple sequencing counts (total and methylated) were summed for each sample at each CpG site, and samples with a total count <4 were dropped from the analysis. The mean methylation ratio in each group was computed for genomic visualization. In the comparison of methylation levels between trophoblasts and cord blood cells, a group sample size of two was considered as a minimum. In order to fit the count data, we used a generalized linear model of Poisson distribution with log link. When all samples in any of the two groups being compared had zero methylation counts, the maximum likelihood estimation of the Poisson model went to infinity. In such cases, the Student’s *t*-test was used alternatively. *P*-values and the group difference in methylation ratios were included above the bar plots in Figures S9 and S12 in the Supplementary Material. For comparisons of methylation levels between the clinical groups, only comparisons with a minimum group sample size of four were considered, and the Wilcoxon rank-sum test was used. *P*-values and the group difference in methylation ratios were included above the bar plots in Figure 11 as well as Figures S10 and S13 in the Supplementary Material. Differential methylation was considered to be mild, moderate, or strong when the *p*-value was <0.05 and the difference in methylation ratios was ≥0.125, ≥0.25, or ≥0.5, respectively. The correlation between methylation levels on each CpG in clinical samples and various demographical, clinical, or histopathological variables were evaluated by Kendall’s tau statistics. Correlation coefficients and *p*-values were plotted in the scatter plots. A Kendall’s tau *p* < 0.05 was considered significant.

### Maternal Study

#### “Virtual” Liquid Biopsy of the Placenta in Preterm Preeclampsia

##### Sample Collection

First trimester placentas and maternal blood samples were collected from healthy Caucasian women undergoing termination of pregnancy (*n* = 12), and processed at the Maternity Clinic and Semmelweis University in Budapest, Hungary. Villous tissues were dissected from the choriodecidua on dry ice and stored at −80^o^C. Aliquots of maternal sera and plasma were stored at −80^o^C.

Pregnancies were dated according to ultrasound scans between 5 and 13 weeks of gestation. Patients with multiple pregnancies were excluded. The collection and investigation of human clinical samples were approved by the Health Science Board of Hungary. Written informed consent was obtained from women prior to sample collection, and the experiments conformed to the principles set out in the World Medical Association Declaration of Helsinki and the Department of Health and Human Services Belmont Report. Specimens and data were stored anonymously.

##### Placental Total RNA Isolation and qRT-PCR

Total RNA was isolated from snap-frozen first trimester placental villous tissues (*n* = 12) with Direct-zol RNA MiniPrep Kit (Zymo Research Corporation) according to the manufacturer’s recommendations. The 28S/18S ratios and the RNA integrity numbers were assessed using an Agilent Bioanalyzer 2100 (Agilent Technologies), and RNA concentrations were measured with NanoDrop 1000. Total RNA (500 ng) was reverse transcribed with the qScript cDNA Synthesis Kit (Quanta Biosciences, Gaithersburg, MD, USA). TaqMan Assays (Applied Biosystems; Data S17 in Supplementary Material) were used for expression profiling on the StepOnePlus Real-Time PCR System (Applied Biosystems).

##### Enzyme-Linked Immunosorbent Assays

Concentrations of leptin and human placental lactogen (hPL) in first (*n* = 12) and third (*n* = 19) trimester maternal blood samples were measured with sensitive and specific immunoassays (Leptin ELISA Kit, Abnova, Taipei City, Taiwan; Human Placental Lactogen ELISA Kit, Alpco, Salem, NH, USA) according to the manufacturers’ instructions. Standard curves were generated, and sample assay values were extrapolated. The sensitivities of the assays were <4.4 ng/ml (leptin) and <550 ng/ml (hPL).

##### Correlation Analysis of Placental Gene Expressions and Maternal Plasma Protein Concentrations

Placental gene expression was measured with either microarray on third trimester samples or qRT-PCR on first trimester samples as described above. Maternal plasma protein concentrations were measured with the above-described immunoassays on respective blood samples taken from the same patients on the day of either delivery or termination of pregnancy. Correlations between placental gene expression and maternal plasma concentrations of gene product proteins were calculated with the Pearson method and visualized on scatter plots (Figure S4 in Supplementary Material).

##### Publication Search, Database Build, and “Virtual” Liquid Biopsy of the Placenta

To detect disease-associated protein signatures of placental dysfunction in maternal blood, similar to plasma DNA tissue mapping for noninvasive prenatal, cancer, and transplantation assessments (176), we performed “virtual” liquid biopsy of the placenta. Briefly, human microarray data on 79 human tissues and cells were downloaded from the BioGPS database, which was used for the generation of placenta enrichment scores (placental expression/mean expression in 78 other tissues and cells). Five genes (*CGB3, CSH1, ENG, FLT1*, and *LEP*) with enrichment scores between 1.4 and 1,490 were selected based on a literature search due to the extensive investigations of their gene products in maternal blood in preeclampsia (Figure S5 in Supplementary Material). Next, an extensive PubMed search was conducted for first trimester maternal blood protein measurements of these five gene products in patients who developed preeclampsia later in pregnancy. Altogether, 61 scientific reports were identified that met the inclusion criteria, which contained data for 80,170 measurements (35, 61, 82, 88, 126, 178–233). These reports were used to build a database for the “virtual” liquid biopsy. In this database, biomarker levels in preterm preeclampsia were expressed as the percentage of control levels as a function of gestational age. Then, the correlations of control percentage values with gestational age were evaluated using the Pearson method. Scatterplots were used to visualize data (Figure S5 in Supplementary Material; Figure 5).

#### Maternal Serum Proteomics Discovery Study

##### Study Groups, Clinical Definitions, and Sample Collection

Women were enrolled in a prospective, longitudinal, and multicenter study (196) in prenatal community clinics of the Maccabi Healthcare Services, Israel. Pregnancies were dated according to the last menstrual period and verified by first trimester ultrasound. Patients with multiple pregnancies or fetuses having congenital or chromosomal abnormalities were excluded. The collection and investigation of human clinical samples were approved by the Maccabi Institutional Review Board. Written informed consent was obtained from women prior to sample collection, and the experiments conformed to the principles set out in the World Medical Association Declaration of Helsinki and the Department of Health and Human Services Belmont Report. Specimens and data were stored anonymously.

Preeclampsia was defined according to the criteria set by the International Society for the Study of Hypertension in Pregnancy (338) and was subdivided into preterm (<37 weeks) or term (≥37 weeks) groups. Severe preeclampsia was defined by Sibai et al. (2). SGA was defined as neonatal BW below the 10th percentile for gestational age. Healthy controls had no medical or obstetric complications and delivered a neonate with a BW appropriate for gestational age.

Peripheral blood samples were obtained by venipuncture in the first trimester from women who subsequently developed preterm severe preeclampsia (*n* = 5) and term severe preeclampsia (*n* = 5) as well as healthy controls (*n* = 10) matched for gestational age at blood draw (Table 4). Blood samples were allowed to clot and were then centrifuged at 10,000 × *g* for 10 min to separate and collect sera. Serum samples were stored in aliquots at −20^o^C in the Maccabi Central Laboratory until shipped on dry ice to Hungary.

##### Sample Preparations, Immunodepletion of High-Abundance Serum Proteins

Sera were immunodepleted at Biosystems International Ltd., (Debrecen, Hungary) for 14 highly abundant serum proteins on an Agilent 1100 HPLC system using Multiple Affinity Removal LC Column-Human 14 (Agilent Technologies) according to the manufacturer’s protocol. To improve the resolution of 2D gels, immunodepleted serum samples were lyophilized, delipidated, and salt-depleted at the Proteomics Laboratory of the Eotvos Lorand University (Budapest, Hungary) (339). The delipidated and salt-depleted plasma protein samples were dissolved in lysis buffer, and their pH was adjusted to 8.0.

##### Fluorescent Labeling and 2D-DIGE

Protein concentrations of the immunodepleted, desalted, and delipidated serum samples were between 2 and 4 µg/µl as determined with PlusOne 2D Quant Kit (GE Healthcare, Little Chalfont, United Kingdom). Samples were equalized for protein content, and then 5 µg of each protein sample was labeled with a CyDye DIGE Fluor Labeling kit for Scarce Samples (saturation dye) (GE Healthcare) according to the manufacturer’s instructions. Individual samples from cases (*n* = 2 × 5) and controls (*n* = 2 × 5) were labeled with Cy5. An internal standard reference sample was pooled from equal amounts (2.5 µg) of all individual samples in this experimental set and was labeled with Cy3. Then, 5 µg of each Cy5-labeled individual sample was merged with 5 µg of the Cy3-labeled reference sample, and these 20 mixtures were run in 2 × 10 gels simultaneously. Briefly, labeled proteins were dissolved in isoelectric focusing (IEF) buffer and were rehydrated passively onto 24 cm immobilized non-linear pH gradient (IPG) strips (pH 3–10, GE Healthcare) for at least 14 h at room temperature. After rehydration, the IPG strips were subjected to the first dimension of IEF for 24 h to attain a total of 80 kVh. Focused proteins were reduced by equilibrating with a buffer containing 1% mercaptoethanol for 20 min. After reduction, IPG strips were loaded onto 10% polyacrylamide gels (24 × 20 cm) and SDS-PAGE was conducted at 12W/gel in the second dimension. Then, gels were scanned in a Typhoon TRIO + scanner (GE Healthcare) using appropriate lasers and filters with the photomultiplier tube biased at 600V. Images in different channels were overlaid using selected colors, and the differences were visualized using Image Quant software (GE Healthcare). Differential protein expression analysis was performed using the differential in-gel analysis and biological variance analysis (BVA) modules of the DeCyder 6.0 software package (GE Healthcare).

##### Identification of DE Protein Spots

The internal standard reference sample representative of every protein present in all experiments was loaded equally in all gels and thus provided an average image for the normalization of individual samples. The determination of the relative abundance of the fluorescent signal between internal standards across all gels provided standardization between the gels, removing experimental variations and reducing gel-to-gel variations. According to the standard proteomic protocol, the threshold for differential expression was set at 1.05-fold minimum fold-change (340). A *p*-value was determined for each protein spot using the Student’s *t*-test by the BVA module of the DeCyder software. A *p*-value of <0.05 was considered statistically significant.

##### Sample Preparation, Fluorescent Labeling, and Preparative 2D-DIGE

The density of spots in the case of Colloidal Coomassie Blue labeling depends only on the concentration of protein in the sample; however, the density of the spots in the case of saturation dye labeling also depends on the number of cysteines of the labeled proteins because the saturation dye labeling method labels all available cysteines on each protein. This results in the same pattern with different density among samples on the analytical and the preparative gels, rendering identification more difficult. To eliminate this problem for the exact identification of proteins in spots of interest, preparative 2D gel electrophoresis was performed using CyDye saturation fluorescent labeling and Colloidal Coomassie Blue labeling in the same gel. A total of 800 µg of proteins per each of the two gels was run. Following electrophoresis, gels were scanned in a Typhoon TRIO + scanner as described above, the significantly altered spots were matched among the “master” analytical and the fluorescent preparative gel images using the BVA module of the DeCyder 6.0 software package. The resolved protein spots were visualized by the Colloidal Coomassie Blue G-250 staining protocol. Individual spots of interest were excised from the gels based on the comparison of the matched images.

##### In-Gel Digestion, Liquid Chromatography–Tandem Mass Spectrometry (LC–MS/MS)

The excised protein spots were analyzed at the Proteomics Research Group of the Biological Research Center of the Hungarian Academy of Sciences (Szeged, Hungary); the detailed protocol is described in http://ms-facility.ucsf.edu/ingel.html. Briefly, salts, SDS, and Coomassie brilliant blue dye were washed out, disulfide bridges were reduced with dithiothreitol, and then free sulfhydryls were alkylated with iodoacetamide. Digestion with side-chain protected porcine trypsin (Promega, Madison, WI, USA) proceeded at 37°C for 4 h. The resulting peptides were extracted from the gel using 1% formic acid in 50% acetonitrile; then the samples were dried down and dissolved in 0.1% formic acid.

Samples were analyzed on an Eldex nanoHPLC system online coupled to a 3D ion trap tandem mass spectrometer (LCQ Fleet, Thermo Scientific) in “triple play” data-dependent acquisition mode, where MS acquisitions were followed by CID analyses on computer-selected multiply charged ions. HPLC conditions included in-line trapping onto a nanoACQUITY UPLC trapping column (Symmetry, C18 5 µm, 180 µm × 20 mm; and 15 µl/min with 3% solvent B) followed by a linear gradient of solvent B (5–60% in 35 min, flow rate: 300 nl/min, where solvent A was 0.1% formic acid in water and solvent B was 0.1% formic acid in acetonitrile) on a Waters Atlantis C18 Column (3 µm, 75 µm × 100 mm).

##### Database Search and Data Interpretation

Raw data files were converted into Mascot generic files (*.mgf) with the Mascot Distiller software v2.1.1.0 (Matrix Science Inc, London, UK). The resulting peak lists were searched against a human subdatabase of the non-redundant protein database of the NCBI (NCBInr, Bethesda, MD, USA) in MS/MS ion search mode on an in-house Mascot server v2.2.04 using Mascot Daemon software v2.2.2 (Matrix Science Inc). Monoisotopic masses with peptide mass tolerance of ±0.6 Da and fragment mass tolerance of ±1 Da were submitted. Trypsin with up to two missed cleavages was specified as an enzyme. Carbamidomethylation of cysteines was set as fixed modification, and acetylation of protein N-termini, methionine oxidation, and pyroglutamic acid formation from peptide N-terminal glutamine residues were permitted as variable modifications. Acceptance criterion was set to at least two significant (peptide score >40, *p* < 0.05) individual peptides per protein. Localization of identified peptides in the core protein sequences was visually analyzed in order to identify potential protein split products.

Biological functions of the altered serum proteins were retrieved from Pathway Studio 9.0 software (Ariadne Genomics Inc., Rockville, MD, USA), and from the open-access GO database^7^ (Figure 6; Figure S6 and Data S8 in Supplementary Material). To elucidate possible interactions between the altered serum proteins and placental genes in the microarray data, bioinformatics analysis was performed using the same software. Molecular networks between the changed serum proteins in preterm (*n* = 19) or term preeclampsia (*n* = 14) and DE placental genes annotated in the GO database (*n* = 1,142) were built separately with a non-linear literature processing search engine, and the resulting connections were manually validated by reading full-text publications (Figure 6; Figure S6 in Supplementary Material). The Fisher’s exact test was used to test for the enrichment of the connections between the altered serum proteins and (1) DE genes in individual modules, taking the connections between the proteins and DE genes in all modules as a background; and (2) DE placental genes, taking the connections between the proteins and all genes tested on the array as a background. To reveal the pathways enriched among the DE genes connected to angiotensinogen, the Ingenuity Pathway Analysis software (QIAGEN, Redwood City, Redwood City, CA, USA) was used, which utilizes Fisher’s exact test and Benjamini–Hochberg correction for multiple testing for the analyses. Statistical significance was set at *q* < 0.01 (Data S10 in Supplementary Material).

#### Maternal Serum Proteomics Validation Pilot Study

##### Study Groups, Clinical Definitions, and Sample Collection

Caucasian women were enrolled in a prospective study at the Department of Obstetrics and Gynaecology of the University of Debrecen and at the Andras Josa Teaching Hospital in Nyiregyhaza, Hungary. Pregnancies were dated according to ultrasound scans between 8 and 14 weeks of gestation. Patients with multiple pregnancies or fetuses having congenital or chromosomal abnormalities were excluded. The collection and investigation of human clinical samples were approved by the Regional Ethics Committee of the University of Debrecen. Written informed consent was obtained from women prior to sample collection, and the experiments conformed to the principles set out in the World Medical Association Declaration of Helsinki and the Department of Health and Human Services Belmont Report. Specimens and data were stored anonymously.

Women were included in the following groups: (1) preterm severe preeclampsia with SGA (*n* = 5) and (2) controls (*n* = 10). Women were matched for gestational age at blood draw (Table 5). Preeclampsia was defined according to the criteria set by the American College of Obstetricians and Gynecologists (1) and was subdivided into preterm (<37 weeks) or term (≥37 weeks) groups. Severe preeclampsia was defined according to Sibai et al. (2). SGA was defined as neonatal BW below the 10th percentile for gestational age. Healthy controls had no medical or obstetric complications and delivered a neonate with a BW appropriate for gestational age.

Peripheral blood samples were obtained by venipuncture in the first trimester. Plasma samples were separated by double centrifugation for 10 min. Samples were stored in aliquots at −80^o^C.

##### Sample Preparations

All solvents were HPLC-grade from Sigma-Aldrich (St. Louis, MO, USA) and all chemicals, where not stated otherwise, were obtained from Sigma-Aldrich. Frozen plasma samples were thawed and denatured with denaturing buffer (Biognosys AG; Schlieren, Switzerland). Samples were alkylated using alkylation solution (Biognosys). Subsequently, samples were digested overnight with sequencing grade modified trypsin (Promega; Madison, WI, USA) at a protein:protease ratio of 50:1. C18 cleanup for mass spectrometry was carried out according to the manufacturer’s instructions using C18 Micro Spin columns (Nest Group Inc.; Southborough, MA, USA). Peptides were dried down to complete dryness using a SpeedVac system. Dried peptides were redissolved with LC solvent A (1% acetonitrile in water with 0.1% formic acid). Final peptide concentrations were determined for all samples by 280 nm measurement (Spectrostar^NANO^, BMG Labtech, Offenburg, Germany). Samples were spiked with PlasmaDive™ (Biognosys) reference peptides mix at known concentrations.

##### LC–MRM Measurements and Data Analysis

Peptides (1 µg per sample, corresponding to injection of 0.0259 µl of initial plasma sample) were injected to a self-packed C18column [75 µm inner diameter and 10 cm column length, New Objective (Woburn, MA, USA); column material was Magic AQ, 3 µm particle size, and 200Å pore size from Michrom] on a ThermoScientific EASY-nLC1000 nano-liquid chromatography system. LC–MRM assays were measured on a ThermoScientific TSQ Vantage triple quadrupole mass spectrometer equipped with a standard nano-electrospray source. The LC gradient for LC–MRM was 6–40% solvent B (85% acetonitrile in water with 0.1% formic acid) for 30 min followed by 40–94% solvent B for 2 min and 94% solvent B for 8 min (total gradient length was 40 min). A subset of Biognosys’ PlasmaDive™ MRM Panel of 10 peptides representing 10 proteins (Data S9 in Supplementary Material) was used for the measurements of 10 altered proteins involved in immune responses. For the quantification of the peptides across samples, the TSQ Vantage was operated in scheduled MRM mode with an acquisition window length of 5 min. The LC eluent was electrosprayed at 1.9 kV and Q1/Q3 were operated at unit resolution (0.7 Da). Signal processing and data analysis were carried out using SpectroDive™ 8.0—Biognosys’ software for multiplexed MRM/PRM data analysis based on mProphet (341). A *q*-value filter of 1% was applied.

Because of the small sample size of this pilot study, statistical simulations were carried out to predict power and significance in the extended validation study by multiple permutation testing. The probability of significant differences between the two groups was estimated by a paired *t*-test and the Mann–Whitney test. For both, *p* < 0.05 was taken as criteria to count successful simulations. The number of successful simulations was calculated for different sample sizes (*n* = 10 or 100) and repeats of simulations (*n* = 10, 100, or 1,000). The simulated significance level at *p* < 0.05 was accepted if the number of successful simulations was >25% (Figure 6; Data S9 in Supplementary Material).

##### Publication Search

An extensive PubMed search was conducted for first trimester maternal blood protein measurements of the DE proteins found by 2D-DIGE. Altogether five scientific reports were identified that met the inclusion criteria (126, 143, 239–241). Figure 6 depicts biomarkers with the same direction differential abundance in preterm preeclampsia in published data as in 2D-DIGE assays.

### Trophoblast Study

#### *In Vitro* Modeling of Placental Disease Pathways

##### Database Search and Data Analysis

To build optimal *in vitro* cellular models of trophoblastic disease, an extensive PubMed search was first conducted for similar assays. Since no human trophoblastic stems cells were yet available that would enable a natural proliferative trophoblastic pool with differentiation potential into villous or extravillous lineages, choriocarcinoma-derived trophoblastic cell lines or immortalized EVTs were mostly used for such purposes. Our search revealed BeWo cells (342) and HTR8/SVneo cells (253) as the increasingly most accepted cell model systems based on 1,500 published articles.

##### Primary VT Differentiation

For *in vitro* trophoblast experiments, placentas (*n* = 6) were collected prospectively at the Perinatology Research Branch (NICHD, NIH, DHHS) from normal pregnant women at term who delivered an AGA neonate with Cesarean section. Cytotrophoblasts were isolated from these placentas by the modified method of Kliman et al. (343). Briefly, 100 g villous tissues were cut, rinsed in PBS, and sequentially digested with Trypsin (0.25%; Life Technologies, Grand Island, NY, USA) and DNAse I (60 U/ml; Sigma-Aldrich) for 90 min at 37^o^C. Dispersed cells were filtered through 100 µm Falcon nylon mesh cell strainers (BD Biosciences, San Jose, CA, USA), and then erythrocytes were lysed with 5 ml NH_4_Cl solution (Stemcell Technologies, Vancouver, BC, Canada). Washed and resuspended cells were layered over 20–50% Percoll gradients and centrifuged for 20 min at 1,200 × *g*. Trophoblast containing bands were collected and non-trophoblastic cells were excluded by negative selection using anti-CD9 (20 µg/ml) and anti-CD14 (20 µg/ml) mouse monoclonal antibodies (R&D Systems, Minneapolis, MN, USA), MACS anti-mouse IgG microbeads, and MS columns (Miltenyi Biotec, Auburn, CA, USA). Then, primary VTs were plated on a collagen-coated 12-well plate (BD Biosciences; 3 × 10^6^ cells/well) in Iscove’s modified Dulbecco’s medium (IMDM; Life Technologies) supplemented with 10% fetal bovine serum (FBS) and 1% penicillin/streptomycin (P/S). To test the effect of trophoblast differentiation on selected genes’ expression, primary trophoblasts were kept in IMDM containing 5% non-pregnant human serum (SeraCare, Milford, MA, USA) and 1% P/S. The medium was replenished every 24 h, and cells were harvested for total RNA every 24 h between days 1 and 7.

##### The Effect of Maternal Serum on Trophoblastic Gene Expression

Villous trophoblasts were isolated from normal term placentas and plated as described above. To test the effect of preeclampsia serum on VTs at two time points of differentiation, VTs were kept in IMDM containing 1% P/S and 10% first trimester maternal sera from control or preeclamptic women. Medium was replenished after 48 h, and cells were harvested for total RNA either 24 or 72 h after the start of the human serum treatment. All experiments were run in triplicate.

##### The Effect of Oxygen Levels, *BCL6*, and *ARNT2* Overexpression on Trophoblastic Gene Expression

BeWo cells (American Type Culture Collection) were incubated in a T-25 flask or 6-well plate with Ham’s F12-K medium (Life Technologies) supplemented with 10% FBS and 1% P/S in a humidified incubator (5% CO_2_, 20% O_2_) at 37^o^C until reaching 50–80% confluence. To test the effect of *ARNT2* or *BCL6* overexpression on gene expression, cells were transiently transfected with *ARNT2, BCL6*, or control (GFP) vectors. Briefly, 4 µg expression plasmid (OriGene Technologies, Inc., Rockville, MD, USA) and 12 µl FuGENE HD transfection reagent (Promega) were mixed with 180 µl Ham’s F12-K medium (10% FBS, 1% P/S), incubated at RT for 15 min and added to cell cultures with 1.8 ml medium in each well of 6-well plate. Twenty-four hours after transfection, cells were split into three treatment groups and kept under either normoxic (20% O_2_), hypoxic (2% O_2_), or ischemic (1% and 20% O_2_ alternating for 6 h) conditions in an Oxycycler C42 (BioSpherix, Lacona, NY, USA) for 48 h before cell harvest. This study setup followed the generally accepted view in reproductive sciences that ~2% O_2_ concentration represents physiologic hypoxia at the implantation site and that placental development occurs under physiologic hypoxia in the first trimester (242, 344).

##### Total RNA Isolation and qRT-PCR

Total RNA was isolated from primary VTs on days 0–7 of differentiation and from BeWo cell cultures with TRIzol reagent (Life Technologies) and RNeasy kit (QIAGEN) according to the manufacturers’ recommendations. The 28S/18S ratios and the RNA integrity numbers were assessed using an Agilent Bioanalyzer 2100; RNA concentrations were measured with NanoDrop 1000. Total RNA (500 ng) was reverse transcribed with High Capacity cDNA Reverse Transcription Kit using random hexamers (Applied Biosystems). TaqMan Assays (Applied Biosystems; Data S17 in Supplementary Material) were used for high-throughput gene expression profiling on the Biomark qRT-PCR system (Fluidigm) according to the manufacturers’ instructions.

##### Data Analysis

###### qRT-PCR

Data were analyzed using the ΔΔCt method. Data were first normalized to the reference gene (*RPLP0*), and log_2_ mRNA relative concentrations were obtained for each sample as −ΔCt_(gene)_ = Ct_(RPLPO) _− Ct_(gene)_.

###### Primary Trophoblast Differentiation

The overall changes in gene expression during the 7 days of differentiation were analyzed by comparing the mean expressions on a given day versus Day 0. The highest fold change for a given gene was defined as the maximum of the daily expression differences during the 7-day time-period. Significant differences were defined by a paired *t*-test (*p* < 0.05) (Figure 7).

###### Serum Treatment of Primary Trophoblast

Gene expression data were analyzed using the Student’s *t*-test to compare the effect of preeclampsia serum with the effect of control serum on gene expression at Days 1 and 3 of trophoblast differentiation. *P*-values of <0.05 were considered significant (Figure 6).

###### BeWo Cell Transfections

Gene expression data were analyzed to compare the effect of *ARNT2* or *BCL6* overexpression with the effect of control vector overexpression on gene expression in normoxic conditions using a one-way ANOVA model. The same model was used to assess the differential effect of *ARNT2, BCL6*, or *GFP* overexpression on gene expression in hypoxic or ischemic conditions vs. normoxia. *P*-values of <0.05 were considered significant (Figure 8).

A *permutation test* was used to measure the statistical significance of the matching between differential gene expression patterns in *in vitro* and *in vivo* conditions. Genes were discretized into three states, i.e. up-regulated (UP), down-regulated (DN), or unchanged (NS). For each gene in the two conditions, a score of 1 was assigned for a perfect match of UP/UP or DN/DN, 0 for a neutral match of NS/NS, −1 for a perfect mismatch of UP/DN or DN/UP, and −0.5 for all other patterns. The matching score for any pair of conditions was computed as the sum of all scores for each individual gene. The significance of the scores was assessed *via* a permutation on the class labels. Permutations were exhaustive when feasible, otherwise limited to a random sample of 5,000 (Data S11 in Supplementary Material).

#### Evaluation of Placental/Trophoblastic Expression and Function of ZNF554

##### Tissue qRT-PCR Array Expression Profiling

TaqMan assays for *ZNF554* and *RPLP0* (Data S17 in Supplementary Material) were run in triplicate for expression profiling of the Human Major Tissue qPCR Array (OriGene Technologies) that contains cDNAs from 48 different pooled tissues.

##### mRNA *In Situ* Hybridization

*In situ* hybridization on third trimester FFPE placental tissues (*n* = 6) retrieved from the Bank of Biological Specimens of the Perinatology Research Branch was carried out using the RNAscope 2.0 FFPE Assay–Brown (Advanced Cell Diagnostics, Hayward, CA, USA) on a HybEZ Hybridization System (Figure 9). Briefly, tissue sections were incubated with *ZNF554* target probe (Cat.No.: 423831, Advanced Cell Diagnostics) for 2 h at 40°C. After rinsing with 1× Wash Buffer, slides underwent a six-step amplification procedure at 40°C and were washed with 1× Wash Buffer between amplification steps. Chromogenic detection was performed using a 1:1 mixture of Brown-A and Brown-B solutions. Slides were counterstained with hematoxylin, dehydrated in graded ethanol, and mounted in xylene.

##### Immunohistochemistry

Third trimester placentas were retrieved from the Bank of Biological Specimens of the Perinatology Research Branch. First trimester placentas were collected from healthy Caucasian women undergoing termination of pregnancy and processed at the Maternity Clinic and Semmelweis University in Budapest, Hungary as described above. Five µm sections of first and third trimester FFPE placental tissues (*n* = 15) were placed on silanized slides and stained using anti-ZNF554 or anti-cytokeratin-7 antibodies as well as reagents listed in Data S18 (Supplementary Material) either on Ventana Discovery (Ventana Medical Systems, Inc, Tucson, AZ, USA) or Leica BOND-MAX (Leica Microsystems, Wetzlar, Germany) autostainers (Figures 9 and 10).

##### Primary VT Differentiation

The experimental procedures were embedded in the study on VT differentiation as described above (Figure 9).

##### BeWo Cell Cultures

BeWo cells were incubated in a T-25 flask or 6-well plate with Ham’s F12-K medium (Life Technologies) supplemented with 10% FBS and 1% P/S in a humidified incubator (5% CO_2_, 20% O_2_) at 37^o^C until reaching 50–80% confluence.

To test the effect of *ZNF554* knockdown on gene expression, cells were treated either with 100 nM *ZNF554* siRNA (Ambion-Life Technologies, Foster City, CA, USA) or 100 nM scrambled (control) siRNA (Ambion) using X-tremeGENE siRNA transfection reagent (Roche, Mississauga, ON, Canada), and incubated at 37^o^C in 2 ml serum-free Opti-MEM (Gibco-Life Technologies) medium. After 6 h, the medium was replaced with 2 ml Ham’s F12-K medium supplemented with 10% FBS. After 48 h, cells were collected for RNA isolation, microarray, and qRT-PCR as well as confocal microscopy (Figure 9).

To test the effect of DNA methylation on gene expression, BeWo cells were treated with 5 or 10 µM 5-azacitidine (Sigma-Aldrich), and control cells with DMSO. This experiment was also performed when both 5-azacitidine-treated and control cells received 25 µM forskolin (Sigma-Aldrich) to induce syncytialization. After 24 h incubation, cells were harvested for RNA isolation and qRT-PCR. The experiment was performed in six replicates (Figure 11; Figure S8 in Supplementary Material).

##### HTR8/SVneo Cell Cultures

HTR8/SVneo EVT cells (kindly provided by Dr. Charles H. Graham, Queen’s University, Kingston, Ontario, Canada) were incubated in a 6-well plate with RPMI-1640 medium (Gibco-Life Technologies) supplemented with 10% FBS and 1% P/S in a humidified incubator (5% CO_2_, 20% O_2_) at 37^o^C until reaching 50% confluence.

To test the effect of *ZNF554* knockdown on gene expression and functions, cells were treated either with 100 nM *ZNF554* siRNA or 100 nM scrambled (control) siRNA as described for BeWo cells. After 6 h, the medium was replaced with 2 ml RPMI-1640 medium (Gibco-Life Technologies) supplemented with 10% FBS. On the following day, cells were kept in various O_2_ concentrations (2, 8, or 20%) in an Oxycycler C42. Cells were collected for functional assays after 24 h, while cells were collected for RNA isolation, microarray, qRT-PCR, or confocal microscopy and their supernatants for ELISA after 48 h. Cell cultures were used for cell proliferation assays after 0, 24, and 48 h.

##### Total RNA Isolation, Microarray, and qRT-PCR

Total RNA was isolated from BeWo and HTR8/SVneo cell cultures with TRIzol reagent (Life Technologies) and RNeasy kit (QIAGEN, Valencia, CA, USA) according to the manufacturers’ recommendations. The 28S/18S ratios and the RNA integrity numbers were assessed using an Agilent Bioanalyzer 2100; RNA concentrations were measured with NanoDrop 1000. DNase-treated RNA from BeWo and HTR8/SVneo cells (500 ng) was amplified and biotin-labeled with the Illumina TotalPrep RNA Amplification Kit (Ambion-Life Technologies). Labeled cRNAs were hybridized to a HumanHT-12v4 Expression BeadChip (Illumina, Inc., San Diego, CA). BeadChips were imaged using a BeadArray Reader (Illumina, Inc.), and raw data were obtained with BeadStudio Software V.3.4.0 (Illumina, Inc.). Total RNA (500 ng) was also reverse transcribed with High Capacity cDNA Reverse Transcription Kit using random hexamers (Applied Biosystems, Foster City, CA, USA). TaqMan Assays (Applied Biosystems; Data S17 in Supplementary Material) were used for high-throughput gene expression profiling on the Biomark qRT-PCR system (Fluidigm, San Francisco, CA, USA) according to the manufacturers’ instructions.

##### Confocal Microscopy

*ZNF554* knock-down and control BeWo and HTR8/SVneo cells cultured in 6-well plate were detached with 0.05% Trypsin-EDTA (Life Technologies), washed, and resuspended in PBS, and then 4 × 10^4^ cells were cytospined to Superfrost Plus slides (Fisher Scientific). Then, cells were fixed with 4% paraformaldehyde (Electron Microscopy Sciences, Hatfield, PA, USA), blocked with Protein Block (Dako North America, Inc., Carpinteria, CA, USA), and immunostained with anti-ZNF554 mouse polyclonal antibody (1:100 dilution, overnight; Abnova, Taipei City, Taiwan) and an AlexaFluor-488 goat anti-mouse antibody (1:1,000 dilution; Life Technologies). Cells were mounted with ProLong Gold antifade reagent with 4’,6-diamidino-2-phenylindole (DAPI; Life Technologies), followed by confocal microscopy using a Leica TCS SP5 MP spectral confocal system (Leica Microsystems) (Figures 9 and 10).

##### Enzyme-Linked Immunosorbent Assays

Concentrations of human plasminogen activator inhibitor-1 (PAI-1) and TIMP-3 in HTR8/SVneo cell culture supernatants were measured with sensitive and specific immunoassays (Human PAI-1 ELISA Kit, Invitrogen; TIMP-3 Human ELISA Kit, Abcam Inc., Cambridge, MA, USA) according to the manufacturers’ instructions. Standard curves were generated, and sample assay values were extrapolated. The sensitivities of the assays were <30 pg/mL (PAI-1) and <2 pg/ml (TIMP-3) (Figure 10).

##### Cell Proliferation Assays

Cell cultures of control and *ZNF554* siRNA treated HTR8/SVneo cells were assayed using the CellTiter 96 Aqueous Non-radioactive Cell Proliferation Assay (Promega) according to the manufacturer’s instructions (Figure S11 in Supplementary Material).

##### Migration Assay

The migratory capacity of HTR8/SVneo cells was examined with 8μm-pore transwell inserts (Corning, NY, USA) inserted in a 12-well plate described previously (345). After transfection with *ZNF554* or scrambled siRNAs for 24 h, 5 × 10^5^ HTR8/SVneo cells were plated in the upper chambers in a serum-free RPMI-1640 medium, whereas the lower chambers contained an RPMI-1640 medium supplemented with 10% FBS. After incubation for 36 h in 2, 8, or 20% O_2_ concentrations, cells on the upper side of the membranes were removed by cotton swab, and the inserts were fixed in methanol for 10 min at RT and washed once with PBS. Then, the membranes were cut out and mounted on Superfrost Plus slides (Fisher Scientific) with ProLong Gold antifade reagent with DAPI. Comprehensive images of each membrane were taken using a Leica TCS SP5 MP spectral confocal system. The number of invaded cells was quantified using Image-Pro Premier v9.0.2 (Media Cybernetics, Inc., Rockville, MD, USA). The experiment was performed in six replicates (Figure 10).

##### Matrigel Invasion Assay

The invasiveness of HTR8/SVneo cells was examined with a Matrigel invasion assay using 8 μm-pore cell culture inserts (BD Biosciences) pre-coated with Matrigel (125 ng/ml; BD Biosciences) and inserted in a 24-well plate described previously (345). After transfection with *ZNF554* or scrambled siRNAs for 24 h, 2 × 10^5^ cells were plated in the upper chambers in a serum-free RPMI-1640 medium, whereas an RPMI-1640 medium supplemented with 10% FBS was added to the lower chambers. After incubation for 48 h in 2, 8, or 20% O_2_ concentrations, cells on the Matrigel side of the membranes were removed by cotton swab, and the membranes were processed as in the migration assay. Comprehensive images taken using the Leica TC5 SP5 spectral confocal system were quantified using Image-Pro Plus 6.2 (Media Cybernetics, Inc.). The experiment was performed in triplicate (Figure 10).

##### Data Analysis

###### Tissue qRT-PCR Array

The expression of *ZNF554* relative to *RPLP0* in the placenta was compared to 47 other human tissues using the Student’s *t*-test. *P*-values of <0.05 were considered significant (Figure 9).

The Student’s *t*-test was used to evaluate *ZNF554* knock-down efficiency and the effect of *ZNF554* knock-down on gene expression in BeWo and HTR8/SVneo cells (Figures 9 and 10).

###### BeWo and HTR8/SVneo Cell Microarray

Data were analyzed using the Bioconductor packages in R (346) following the MIAME guidelines and methodologies described previously (318). Raw microarray gene expression data were normalized by a quantile normalization approach. A moderated *t*-test was used to select DE genes using a cutoff of >1.5 fold-change and *q* < 0.1. GO analysis and pathway analysis using the Kyoto Encyclopedia of Genes and Genomes (KEGG) pathway database were also performed.

###### HTR8/SVneo Cell qRT-PCR, Immunoassay, Cell Proliferation, Migration, and Invasion

qRT-PCR data were analyzed using the ΔΔCt method relative to *RPLP0* expression. The Student’s *t*-test was used to evaluate *ZNF554* knock-down efficiency in HTR8/SVneo cells and the effect of *ZNF554* knock-down on gene expression and cell proliferation. A linear model was built to quantify the effects of *ZNF554* knock-down and various O_2_ concentrations on the gene expression and protein secretion of HTR8/SVneo cells as well as on their migratory and invasive capacity. O_2_ concentration was treated as a continuous variable, and the interaction between *ZNF554* knock-down and O_2_ concentrations was retained in the model when the coefficient was significant. *P*-values of <0.05 were considered significant (Figure 10).

## Data Availability

All relevant data are within the paper and its Supplementary Material files. MIAME compliant microarray data are available from the Gene Expression Omnibus (GEO) under accession numbers GSE65866, GSE65940, and GSE66273.

## Ethics Statement

The collection and use of human biological specimens and clinical data for research purposes were approved by the Health Science Board of Hungary, the Institutional Review Board of the *Eunice Kennedy Shriver* National Institute of Child Health and Human Development (NICHD, NIH, DHHS), the Wayne State University Human Investigation Committee, the Maccabi Institutional Review Board, and the Regional Ethics Committee of the University of Debrecen. Written informed consent was obtained from women prior to sample collection, and the experiments conformed to the principles set out in the World Medical Association Declaration of Helsinki and the Department of Health and Human Services Belmont Report. Specimens and data were stored anonymously.

## Author Contributions

NGT conceptualized study and designed research. NGT, KAK, YX, KJ, RJL, EH-G, ZsD, AS, KE, SzSz, VT, HE-A, CL, AB, GSz, SL, and ZD performed research. NGT, RR, ALT, ZX, LO, OT, HM, SD, SSH, THC, CJK, and ZP contributed new reagents/analytic tools/clinical specimens. NGT, RR, ALT, KAK, YX, ZX, KJ, GB, ZsG, JP, THC, BAGy, AD, ASz, ZsD, GSz, IK, AF, MKr, MKn, OE, GJB, CJK, GJ, and ZP analyzed and interpreted data. All authors contributed to manuscript writing and approved the paper.

## Conflict of Interest Statement

Part of the data was submitted by NGT, RR, ALT, KAK, RJL, THC, HM, MK, GJ, and ZP as patent applications in 2012 and 2014 to describe biomarkers for preeclampsia. Other authors declare no conflict of interest.

## Footnotes

^1^www.r-project.org.

^2^www.genecards.org.

^3^www.bioconductor.org.

^4^http://genome.ucsc.edu/.

^5^www.epigenomebrowser.org.

^6^http://www.bioinformatics.babraham.ac.uk/projects/download.html#bismark.

^7^http://www.geneontology.org/.

## Supplementary Material

Figure S1 Hub transcription regulatory genes in the M1 and M2 modules and their interaction network. **(A)** Co-expression matrix of transcription regulatory genes and predominantly placenta-expressed genes in the M1 and M2 modules. Within the M1 (green) module, *ESRRG, POU5F1*, and *ZNF554* transcription regulatory genes correlated most strongly with predominantly placenta-expressed genes. *ESRRG* and *ZNF554* had the most correlation with *CSH1* and *HSD11B2*, genes strongly implicated in fetal growth. Within the M2 (red) module, *BCL6, BHLHE40*, and *ARNT2* transcription regulatory genes were correlated most strongly with predominantly placenta-expressed genes. *BCL6* and *ARNT2* had the most correlation with *FLT1*. Heatmap represents Pearson coefficients. **(B,C)** The networks of biological processes enriched among genes dysregulated in preeclampsia and co-expressed with hub factors in M1 (*ZNF554*) and M2 (*BCL6*) modules were visualized with the BINGO module of Cytoscape. Sizes of the circles relate to the number of genes involved in the biological processes and colors refer to *p*-values. The groups of most enriched biological processes were manually circled and labeled. The color code depicts *p*-values.

Figure S2 Clinical characteristics in the transcriptomic validation study groups. Blood pressure, birth weight, and gestational age data from the 100 pregnant women included in the validation study show that preeclampsia phenotypes are heterogeneous, underlining the complex pathways of disease in preeclampsia.

Figure S3 Placental gene expression changes in various phenotypes of preeclampsia detected by qRT-PCR. Data represent gene expressions relative to *RPLP0* measured across 100 placentas. In each bar plot (mean ± SE), the left and right panels show significant differences (“*”) in preterm and term preeclampsia associated with or without SGA samples compared to gestational age-matched controls, respectively. Changes in preterm preeclampsia samples significantly different to changes in term preeclampsia samples are indicated by “+”.

Figure S4 The correlation between placental gene expression and maternal plasma protein concentration. Placental *LEP* and *CSH1* gene expression was either measured with microarrays in the third trimester or with qRT-PCR in the first trimester. Maternal plasma leptin and serum human placental lactogen protein concentrations were measured with ELISA. Third trimester placental microarray data were correlated with ELISA data from maternal blood samples collected at the time of delivery from the same patients. qRT-PCR data from placentas taken from first trimester terminations were correlated with ELISA data from blood samples collected at the time of the procedure from the same patients. Correlations were investigated with the Pearson method and visualized on scatter plots. The two investigated genes’ expression and their protein products’ concentrations correlated both in the first and third trimesters.

Figure S5 The timing of gene module dysregulation in preterm preeclampsia. **(A)** Human microarray data on 79 human tissues and cells downloaded from the BioGPS database was used for the generation of placenta enrichment scores (placental expression/mean expression in 78 other tissues and cells). Five genes with scores between 1.4 and 1,490 were selected based on literature search due to the extensive investigations of their gene products in maternal blood in preeclampsia. Colors depict gene module involvement. **(B–F)** The 80,170 measurements for five gene products published in 61 scientific reports (35, 61, 82, 88, 126, 178–233) were used for the virtual liquid biopsy of the placenta in preterm preeclampsia. Biomarker levels in preterm preeclampsia were expressed as the percentage of control levels (dotted lines) throughout pregnancy. Percentage values were represented in the scatter plots by different colors reflecting gene module classification. Based on qRT-PCR data, sEng belongs to M2 (red) module. The number of measurements, the Pearson correlation values for biomarker levels, and gestational age as well as corresponding *p*-values are depicted for each biomarker.

Figure S6 Maternal blood proteomic changes in term preeclampsia and their effect on differentially expressed (DE) genes in the placenta. **(A)** The 14 DE maternal serum proteins in term preeclampsia belong to six functional groups. **(B)** These 14 proteins have connections with 116 DE placental genes, among which 46 belong to the M2 (red) module. Angiotensinogen has more connections than other proteins (OR = 2.5, *p* = 1.6 × 10^−8^) and the most with M2 (red) module genes (*n* = 35). Seventy seven of 86 connections of angiotensinogen have a directional effect toward the gene.

Figure S7 Summary of functional experiments on module M2. Epigenetic changes to the trophoblast and abnormal trophoblast differentiation lead to a general down-regulation of gene expression and the up-regulation of hub factors in module M2 (e.g. *BCL6*). After placental circulation has been established and placental ischemic stress occurs, the up-regulation of *BCL6* sensitizes the trophoblast to ischemia by inducing *ARNT2* up-regulation and downstream increase of expression of *FLT1, ENG, LEP*, leading to the placental release of pro-inflammatory and anti-angiogenic gene products. This pathway is only observed in preterm preeclampsia, suggesting that the dysregulation of this placental pathway promotes the early development of preeclampsia. The alterations in maternal blood proteome can induce trophoblastic functional changes leading to the up-regulation of module M2 genes, the overproduction of sFlt-1 and an anti-angiogenic state through a trajectory that does not necessarily affect fetal growth.

Figure S8 DNA methylation regulates *BCL6* expression in the trophoblast. **(A)** Decreased *BCL6* expression was observed in BeWo cells upon treatment with 5-azacitidine (5-AZA) irrespective of Forskolin (FRSK) co-treatment. **(B)** Upper three lanes: whole genome bisulfite sequencing data of *BCL6* first intron from the Human Reference Epigenome Mapping Project. H1 ESC; H1 embryonic stem cell; HBDT, H1 BMP4-derived trophoblast; and HDNP, H1-derived neuronal progenitor. Lower three lanes: bisulfite sequencing data in this study. Abbreviations: CB, cord blood cell; CT, cytotrophoblast; ST, syncytiotrophoblast. Red box: differentially methylated region; red arrow: CpG Chr3:187458163.

Figure S9 DNA methylation levels at individual CpGs in *BCL6* in the trophoblast and umbilical cord blood cells. DNA methylation levels (0–100%) at individual CpGs in *BCL6* in umbilical cord blood cells (CB), cytotrophoblasts (CT), and differentiated syncytiotrophoblasts (ST) are depicted in the bar plots that represent means and SEs. Umbilical cord blood cells and cytotrophoblasts were obtained from the same fetuses. The genomic coordinates of the CpGs, the group differences (CB vs. CT; CT vs. ST) in mean DNA methylation levels and the *p*-values are shown above the bar plots. The number of samples analyzed with methylation reads above the threshold are shown below the bar plots (only comparisons with a group sample size of minimum two were considered). Differential methylation was claimed to be mild, moderate, or strong when the *p*-value was <0.05 and the difference in methylation level was ≥0.125, ≥0.25, or ≥0.5, respectively.

Figure S10 DNA methylation levels at individual CpGs in *BCL6* in the trophoblast in controls and in cases of preeclampsia. DNA methylation levels (0–100%) at individual CpGs in *BCL6* in laser captured trophoblasts are depicted in the bar plots that represent means and SEs. The genomic coordinates of the CpGs, the group differences (compared preterm or term controls) in DNA methylation levels and the *p*-values are shown above the bar plots. The number of samples analyzed with methylation reads above the threshold are shown below the bar plots (only comparisons with a group sample size of minimum four were considered). Differential methylation was claimed to be mild, moderate, or strong when the *p*-value was <0.05 and the difference in methylation level was ≥0.125, ≥0.25, or ≥0.5, respectively. Preterm (left) and term (right) groups of patients were analyzed separately. Abbreviations: PE, preeclampsia; PE + SGA, preeclampsia associated with small-for-gestational age.

Figure S11 The effect of *ZNF554* knock-down on cell proliferation in HTR8/SVneo extravillous trophoblastic cells. **(A)** Cell proliferation assays showed that *ZNF554* knock-down slightly but significantly decreased (−14%, *p* = 0.02) cell proliferation rate in HTR8/SVneo extravillous trophoblastic cells after 48 h. *Y*-axis depicts viable cell number, *X*-axis shows incubation time. **(B)** The differential expression of *CDKN1A* (cyclin-dependent kinase inhibitor 1A) and *STK40* (serine/threonine kinase 40), genes involved in the regulation of cell cycle, upon *ZNF554* knock-down was confirmed by qRT-PCR.

Figure S12 DNA methylation levels at individual CpGs in *ZNF554* in the trophoblast and umbilical cord blood cells. DNA methylation levels (0–100%) at individual CpGs in *ZNF554* in umbilical cord blood cells (CB), cytotrophoblasts (CT), and differentiated syncytiotrophoblasts (ST) are depicted in the bar plots that represent means and SEs. Umbilical cord blood cells and CT were obtained from the same fetuses. The genomic coordinates of the CpGs, the group differences (CB vs. CT; CT vs. ST) in mean methylation levels and the *p*-values are shown above the bar plots. The number of samples analyzed with methylation reads above the threshold are shown below the bar plots (only comparisons with a group sample size of minimum two were considered). Differential methylation was claimed to be mild, moderate, or strong when the *p*-value was <0.05 and the difference in methylation level was ≥0.125, ≥0.25, or ≥0.5, respectively.

Figure S13 DNA methylation levels at individual CpGs in *ZNF554* in the trophoblast in controls and in cases of preeclampsia. DNA methylation levels (0–100%) at individual CpGs in *ZNF554* in laser captured trophoblasts are depicted in the bar plots that represent means and SEs. The genomic coordinates of the CpGs, the group differences (compared preterm or term controls) in methylation levels, and the *p*-values are shown above the bar plots. The number of samples analyzed with methylation reads above the threshold are shown below the bar plots (only comparisons with a group sample size of minimum four were considered). Differential methylation was claimed to be mild, moderate, or strong when the *p*-value was <0.05 and the difference in methylation level was ≥0.125, ≥0.25, or ≥0.5, respectively. Preterm (left) and term (right) groups of patients were analyzed separately. Abbreviations: PE, preeclampsia; PE + SGA, preeclampsia associated with SGA.

Data S1 Genes differentially expressed in the placenta in preterm preeclampsia.

Data S2 Predominantly placenta-expressed genes.

Data S3 The enrichment of differentially expressed genes on chromosomes.

Data S4 The enrichment of differentially expressed transcription regulatory genes on chromosomes.

Data S5 The enrichment of predominantly placenta-expressed genes on chromosomes.

Data S6 Genes associated with blood pressure.

Data S7 The association of gene expression with placental pathology.

Data S8 Maternal blood proteomic changes in preeclampsia—two-dimensional differential in-gel electrophoresis.

Data S9 Maternal blood proteomic changes in preeclampsia—multiple reaction monitoring.

Data S10 Placental pathways enriched among the differentially expressed genes connected to angiotensinogen.

Data S11 Permutation test of functional experiments.

Data S12 Enrichment of transposable elements in genes within the M1 and M2 gene modules.

Data S13 Genes differentially expressed in ZNF554-silenced BeWo cells.

Data S14 Enrichment analysis of ZNF554-silenced BeWo cells.

Data S15 Genes differentially expressed in ZNF554-silenced HTR8/SVneo cells.

Data S16 Enrichment analysis of ZNF554-silenced HTR8/SVneo cells.

Data S17 TaqMan assays.

Data S18 Immunostaining conditions and antibodies.
