# Supplementary figures and images for: Integrated Systems Biology Approach Identifies Novel Maternal and Placental Pathways of Preeclampsia
Source: Front Immunol. 2018 Aug 8;9:1661. doi: 10.3389/fimmu.2018.01661 (PMC6092567; doi:10.3389/fimmu.2018.01661)

**A** M1 - green module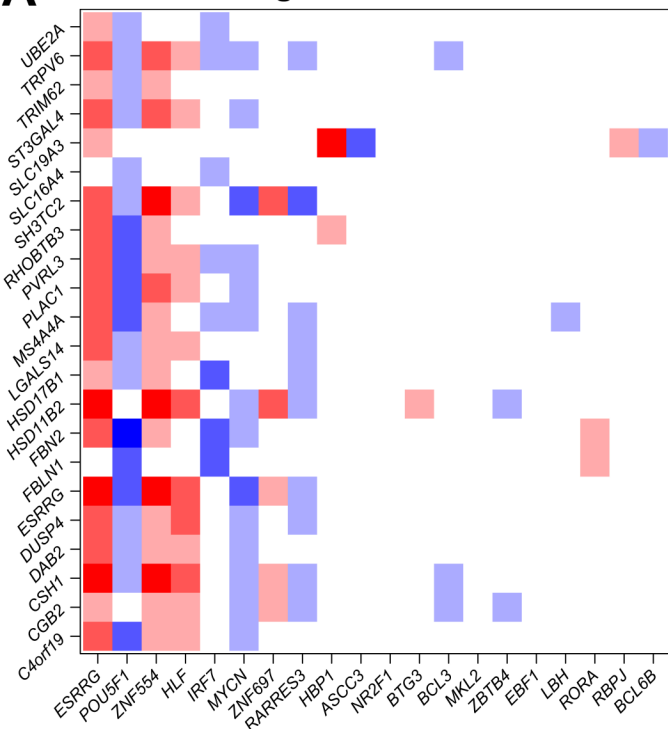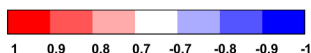**M2 - red module**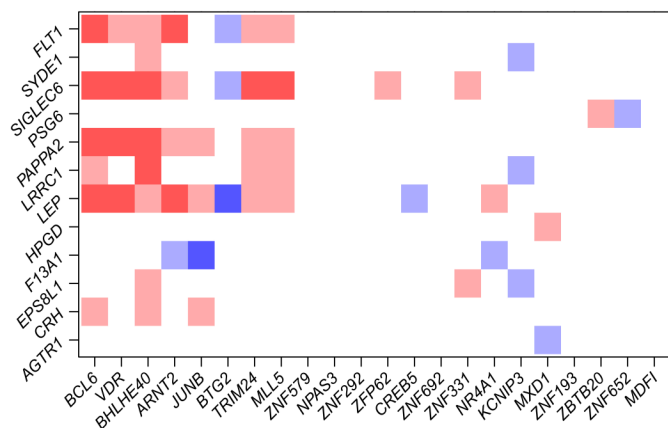**B** ZNF554 co-expression network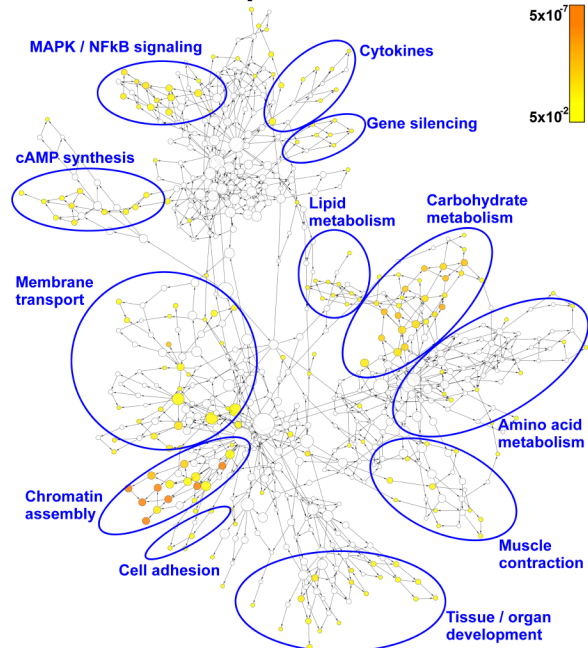**C** BCL6 co-expression network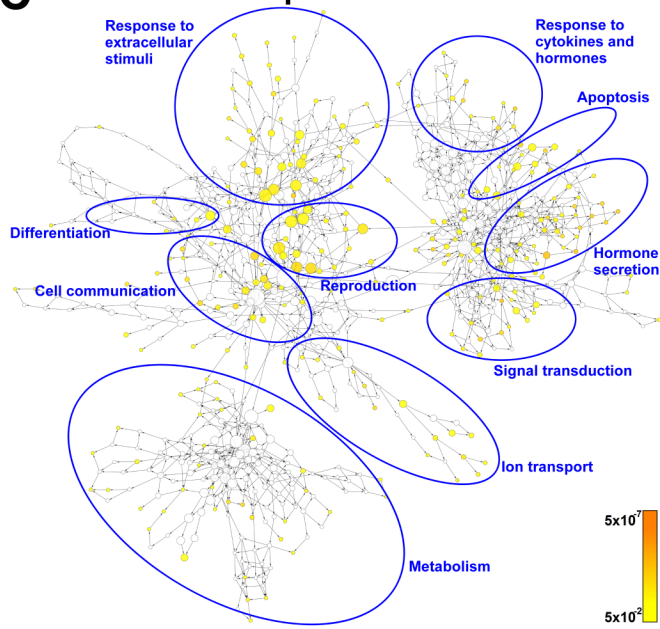

Supplement: Figure S1 — Hub transcription regulatory genes in the M1 and M2 modules and their interaction network. (A) Co-expression matrix of transcription regulatory genes and predominantly placenta-expressed genes in the M1 and M2 modules. Within the M1 (green) module, ESRRG, POU5F1, and ZNF554 transcription regulatory genes correlated most strongly with predominantly placenta-expressed genes. ESRRG and ZNF554 had the most correlation with CSH1 and HSD11B2, genes strongly implicated in fetal growth. Within the M2 (red) module, BCL6, BHLHE40, and ARNT2 transcription regulatory genes were correlated most strongly with predominantly placenta-expressed genes. BCL6 and ARNT2 had the most correlation with FLT1. Heatmap represents Pearson coefficients. (B,C) The networks of biological processes enriched among genes dysregulated in preeclampsia and co-expressed with hub factors in M1 (ZNF554) and M2 (BCL6) modules were visualized with the BINGO module of Cytoscape. Sizes of the circles relate to the number of genes involved in the biological processes and colors refer to p-values. The groups of most enriched biological processes were manually circled and labeled. The color code depicts p-values. [file Image_1.pdf]

● Control    ● Preeclampsia    ● Preeclampsia + SGA

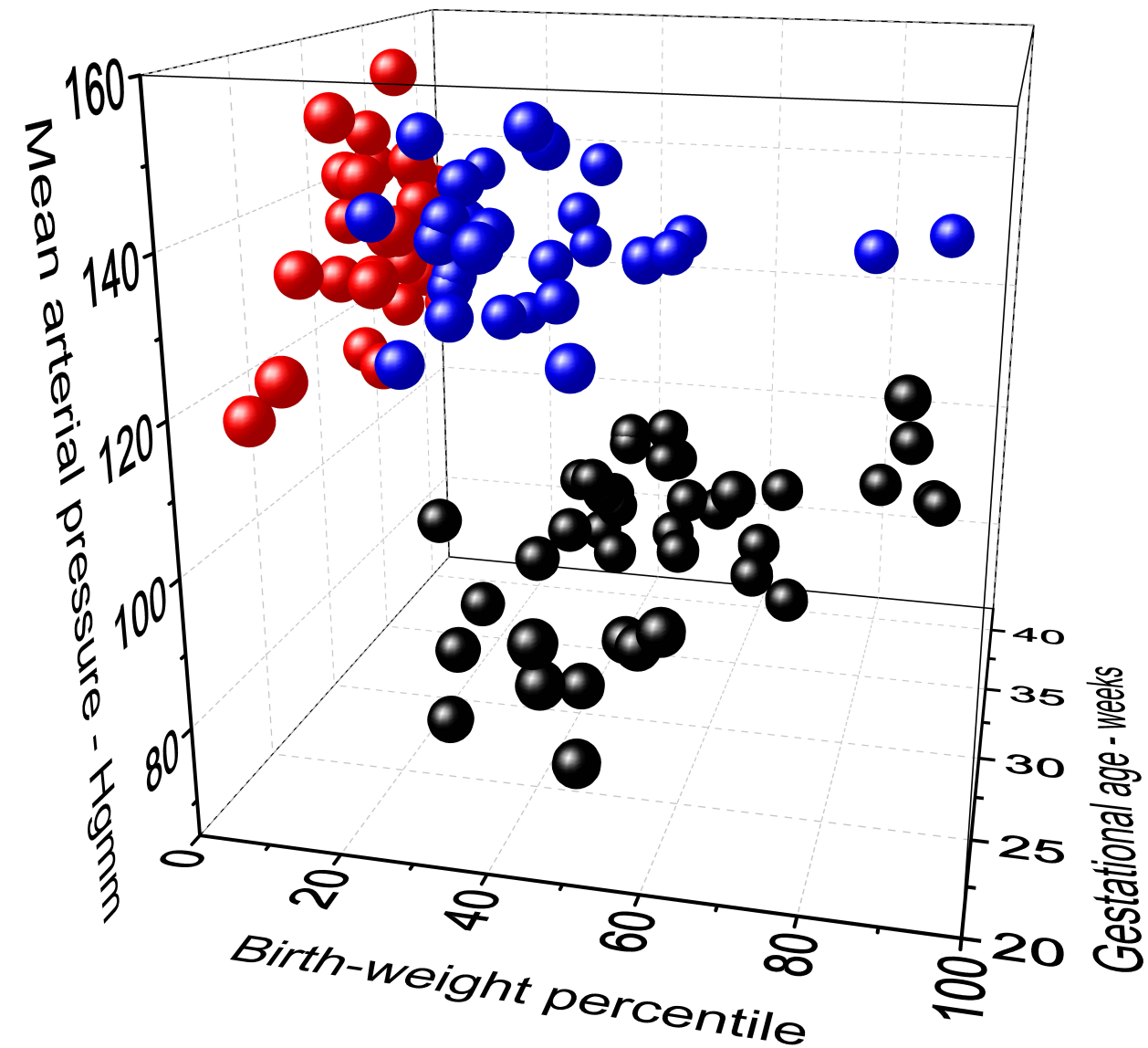

Supplement: Figure S2 — Clinical characteristics in the transcriptomic validation study groups. Blood pressure, birth weight, and gestational age data from the 100 pregnant women included in the validation study show that preeclampsia phenotypes are heterogeneous, underlining the complex pathways of disease in preeclampsia. [file Image_2.pdf]

**LEP (1st trimester)**

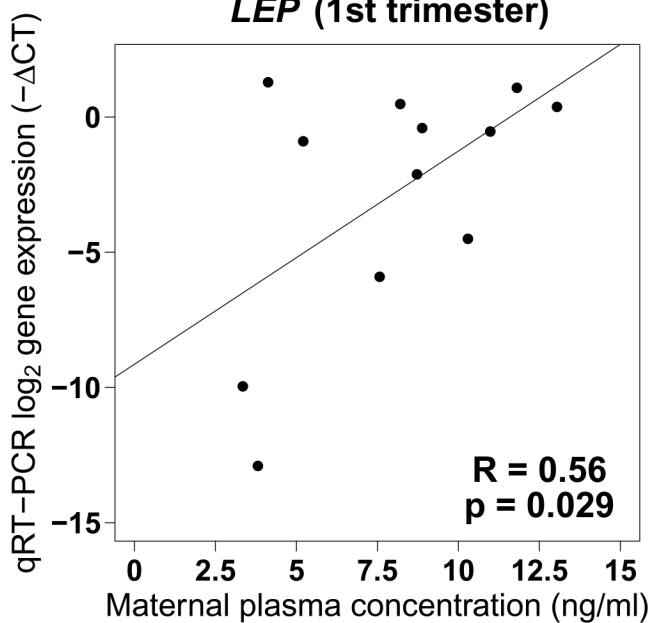

**CSH1 (1st trimester)**

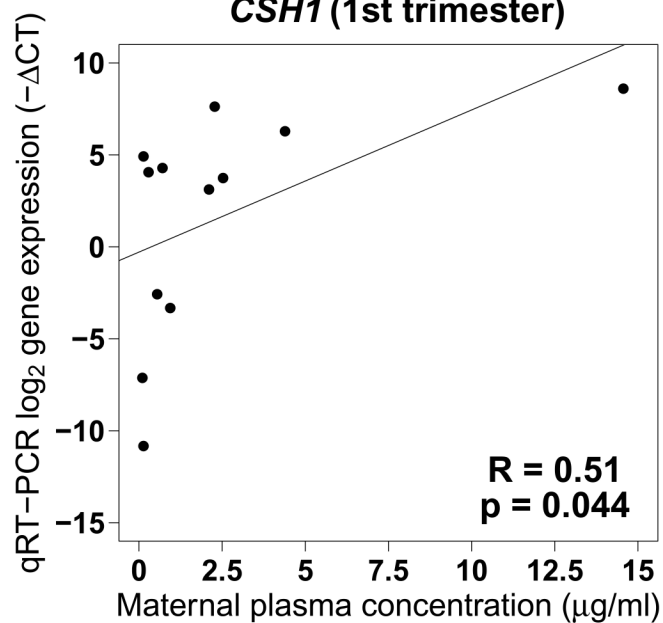

**Leptin (3rd trimester)**

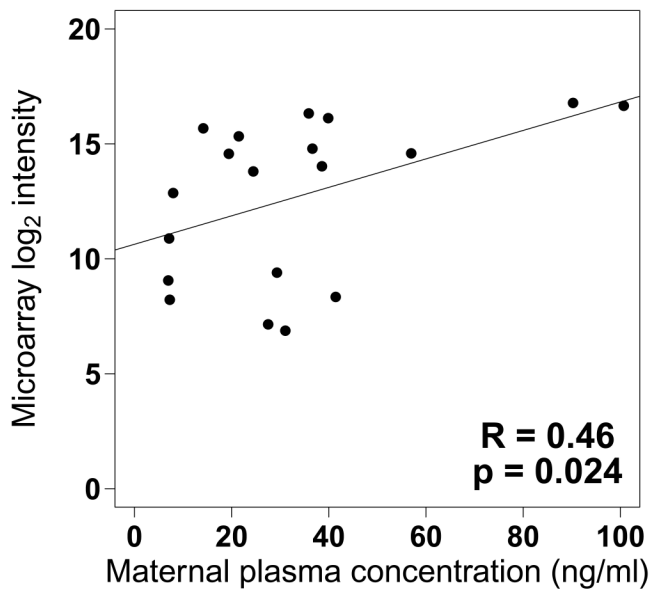

**hPL (3rd trimester)**

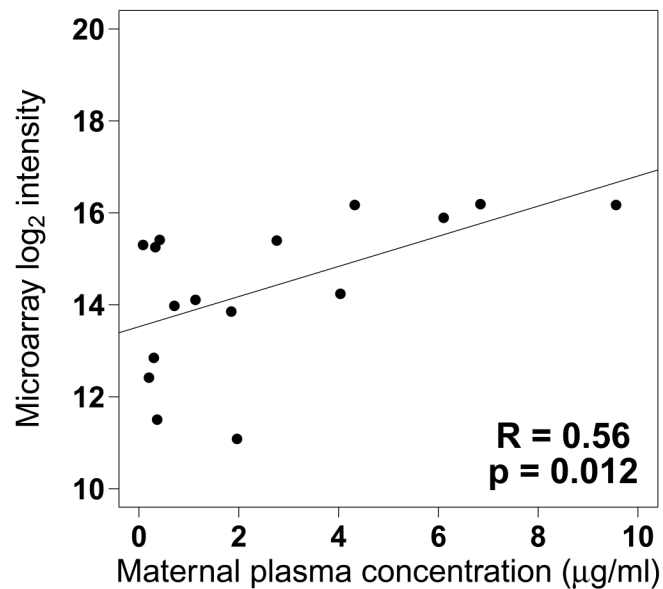

Supplement: Figure S4 — The correlation between placental gene expression and maternal plasma protein concentration. Placental LEP and CSH1 gene expression was either measured with microarrays in the third trimester or with qRT-PCR in the first trimester. Maternal plasma leptin and serum human placental lactogen protein concentrations were measured with ELISA. Third trimester placental microarray data were correlated with ELISA data from maternal blood samples collected at the time of delivery from the same patients. qRT-PCR data from placentas taken from first trimester terminations were correlated with ELISA data from blood samples collected at the time of the procedure from the same patients. Correlations were investigated with the Pearson method and visualized on scatter plots. The two investigated genes’ expression and their protein products’ concentrations correlated both in the first and third trimesters. [file Image_4.pdf]

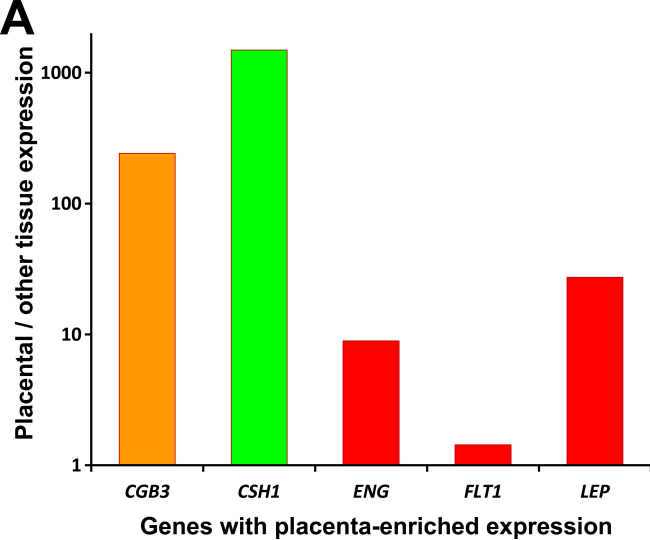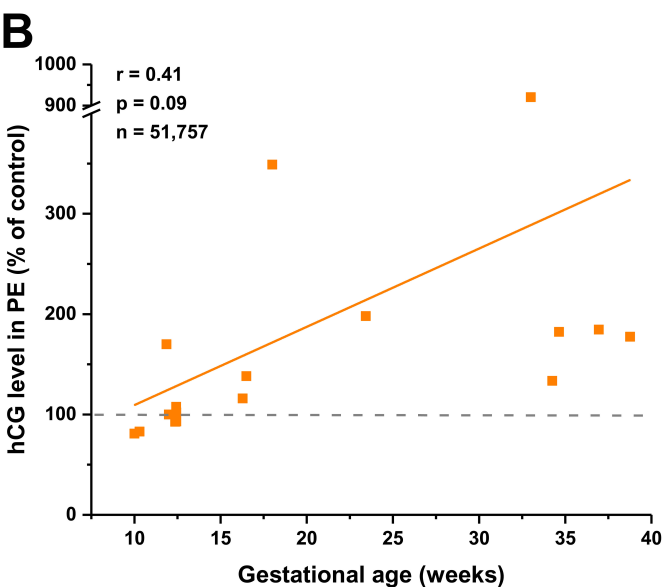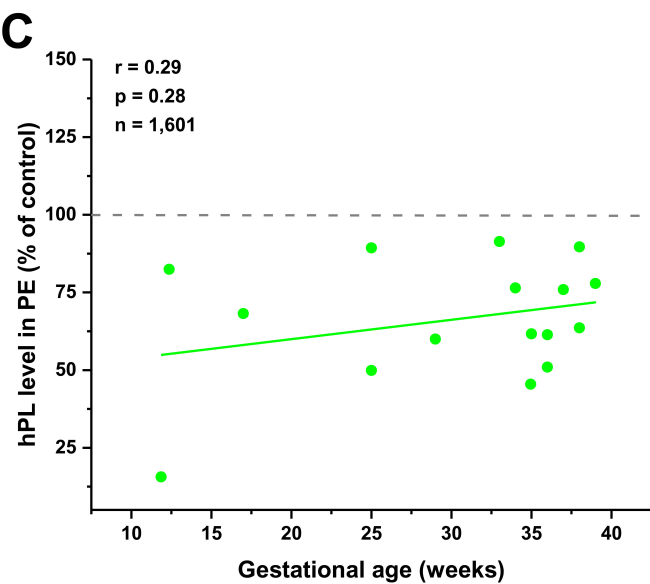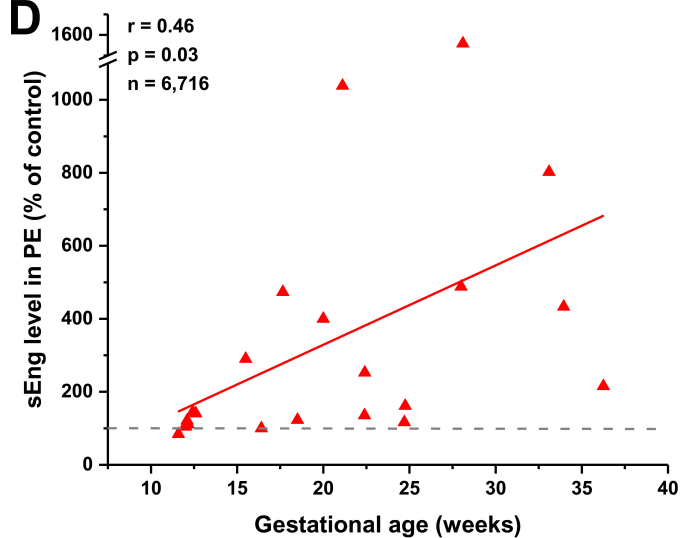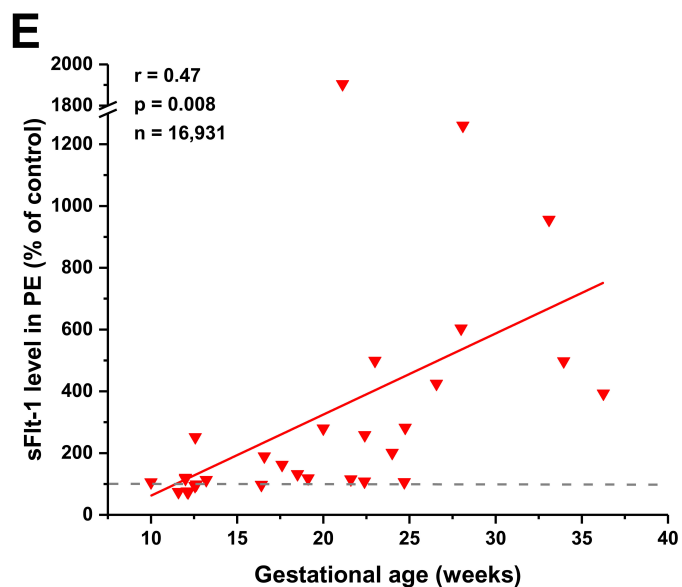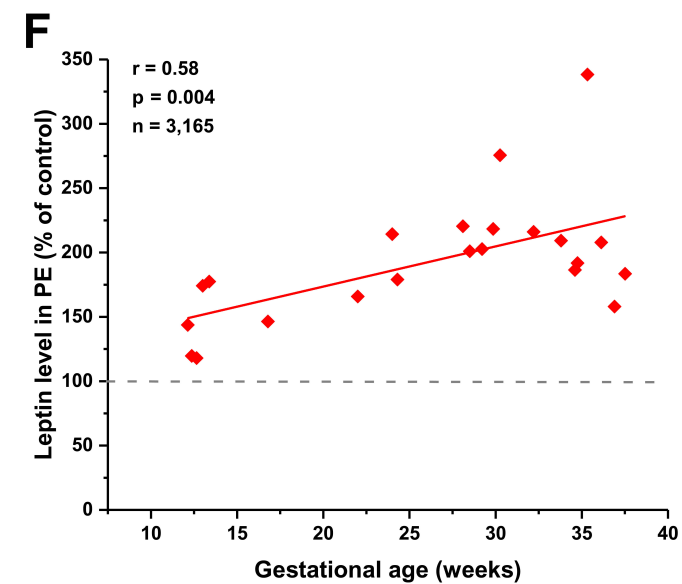

Supplement: Figure S5 — The timing of gene module dysregulation in preterm preeclampsia. (A) Human microarray data on 79 human tissues and cells downloaded from the BioGPS database was used for the generation of placenta enrichment scores (placental expression/mean expression in 78 other tissues and cells). Five genes with scores between 1.4 and 1,490 were selected based on literature search due to the extensive investigations of their gene products in maternal blood in preeclampsia. Colors depict gene module involvement. (B–F) The 80,170 measurements for five gene products published in 61 scientific reports (35, 61, 82, 88, 126, 178–233) were used for the virtual liquid biopsy of the placenta in preterm preeclampsia. Biomarker levels in preterm preeclampsia were expressed as the percentage of control levels (dotted lines) throughout pregnancy. Percentage values were represented in the scatter plots by different colors reflecting gene module classification. Based on qRT-PCR data, sEng belongs to M2 (red) module. The number of measurements, the Pearson correlation values for biomarker levels, and gestational age as well as corresponding p-values are depicted for each biomarker. [file Image_5.pdf]

**A****Relative gene  
expression*****BCL6***

5-AZA ( $\mu$ M) 0 5 10 0 5 10  
FRSK ( $\mu$ M) 0 0 0 25 25 25

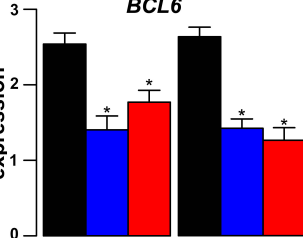**B*****BCL6***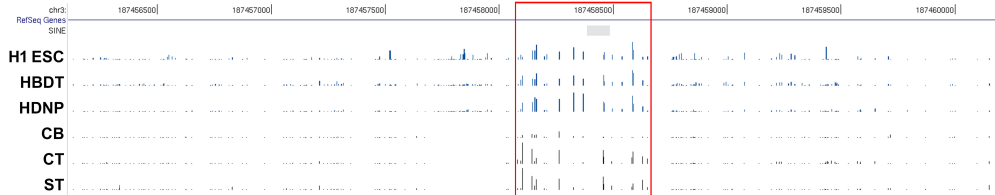

Supplement: Figure S8 — DNA methylation regulates BCL6 expression in the trophoblast. (A) Decreased BCL6 expression was observed in BeWo cells upon treatment with 5-azacitidine (5-AZA) irrespective of Forskolin (FRSK) co-treatment. (B) Upper three lanes: whole genome bisulfite sequencing data of BCL6 first intron from the Human Reference Epigenome Mapping Project. H1 ESC; H1 embryonic stem cell; HBDT, H1 BMP4-derived trophoblast; and HDNP, H1-derived neuronal progenitor. Lower three lanes: bisulfite sequencing data in this study. Abbreviations: CB, cord blood cell; CT, cytotrophoblast; ST, syncytiotrophoblast. Red box: differentially methylated region; red arrow: CpG Chr3:187458163. [file Image_8.pdf]

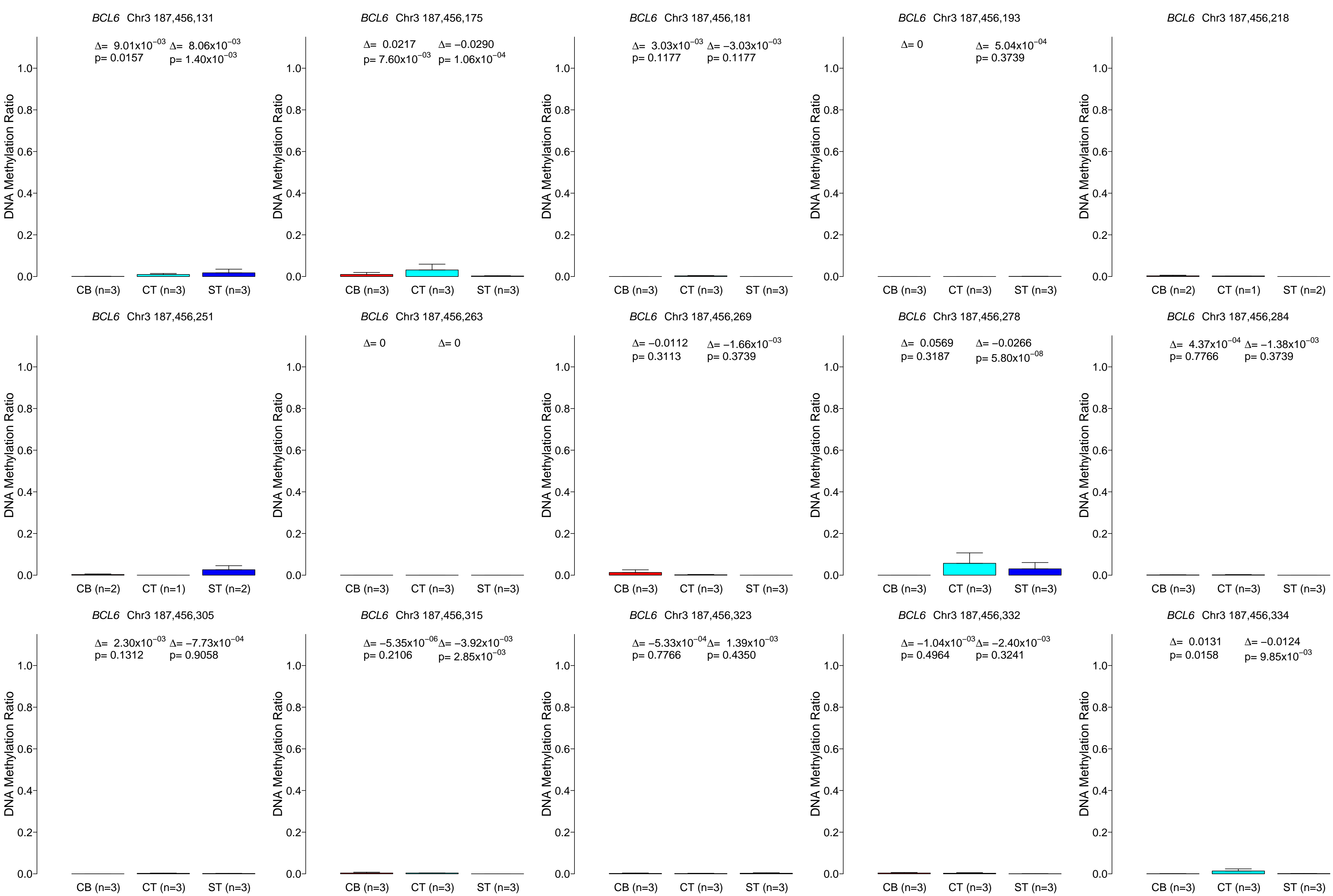

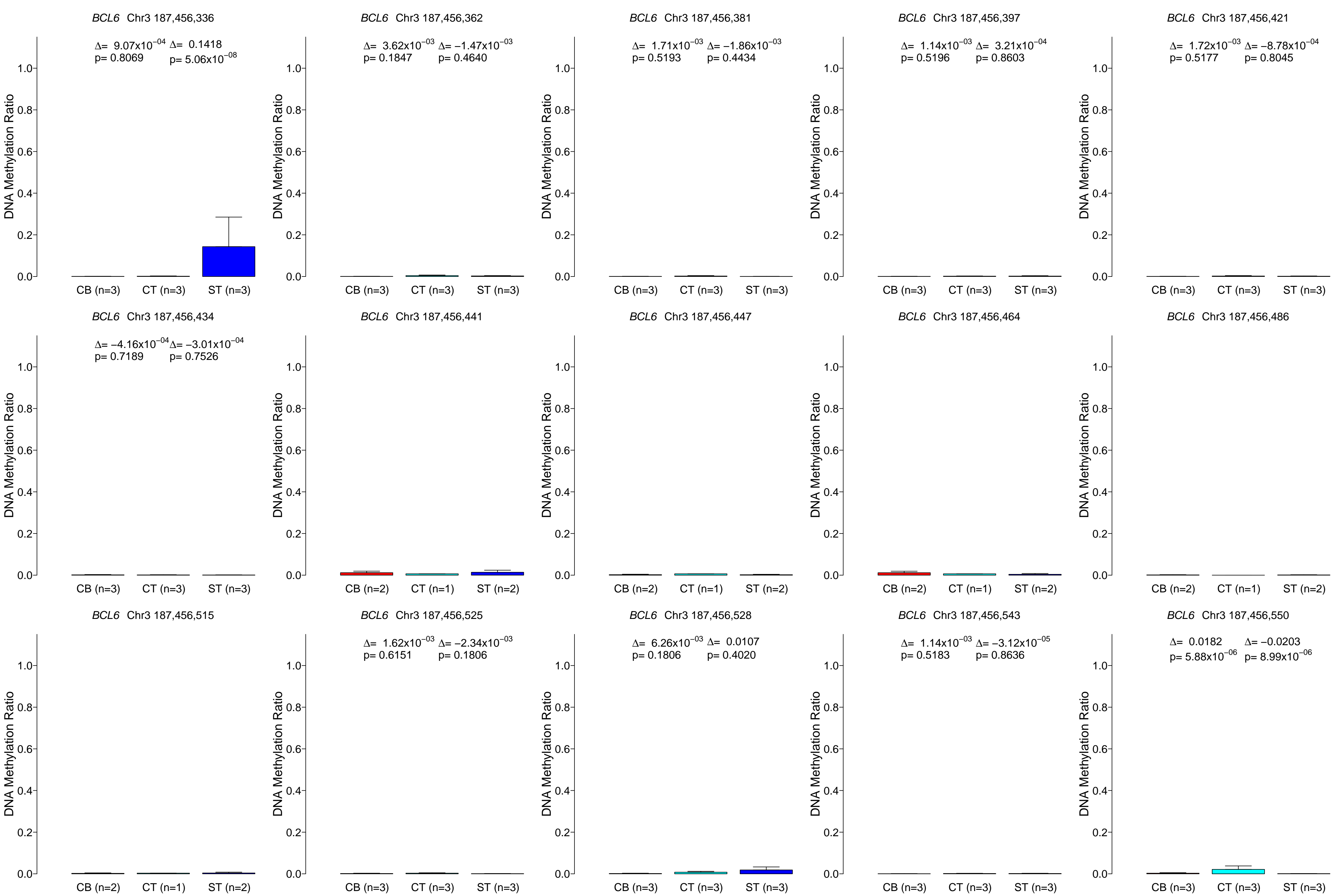

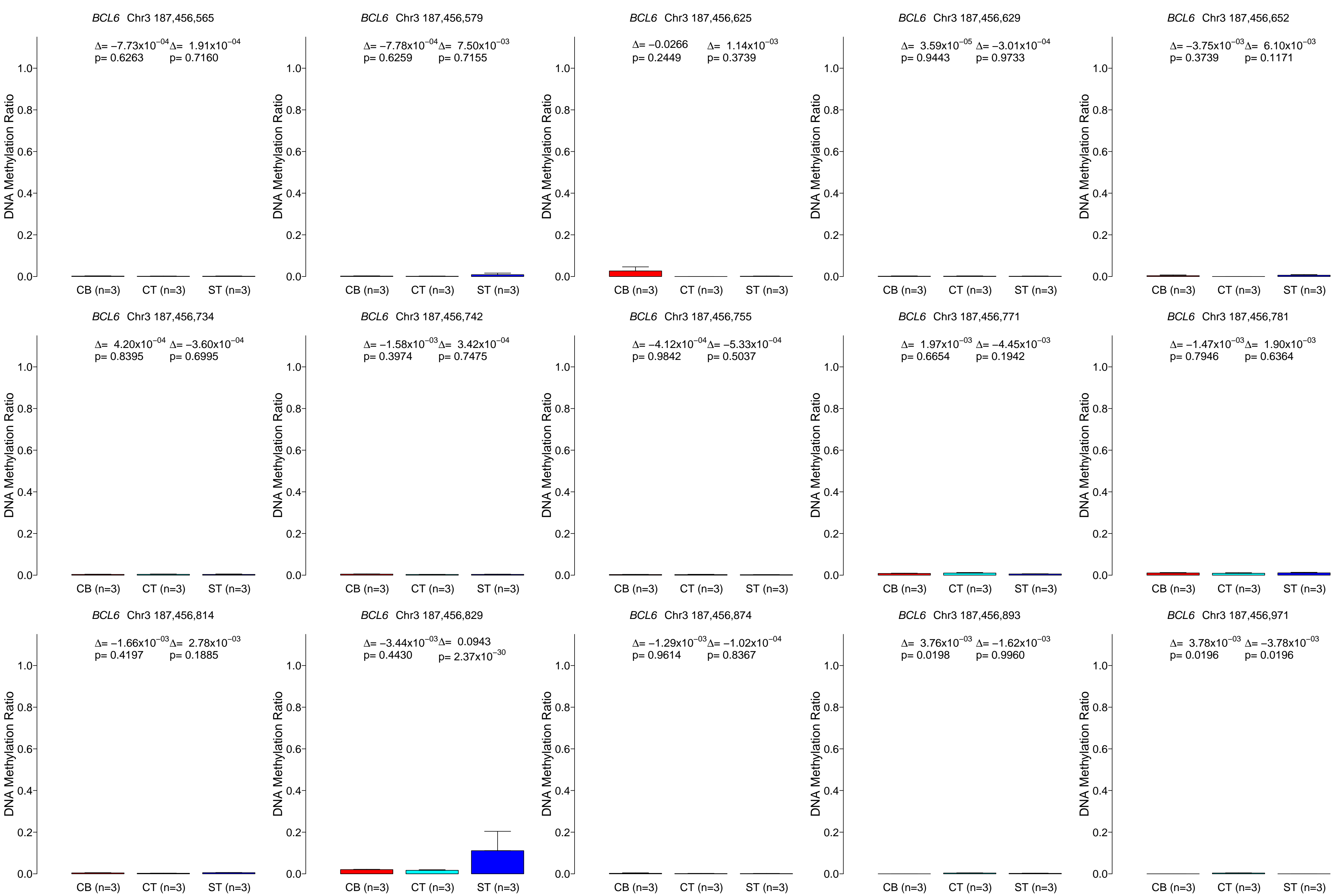

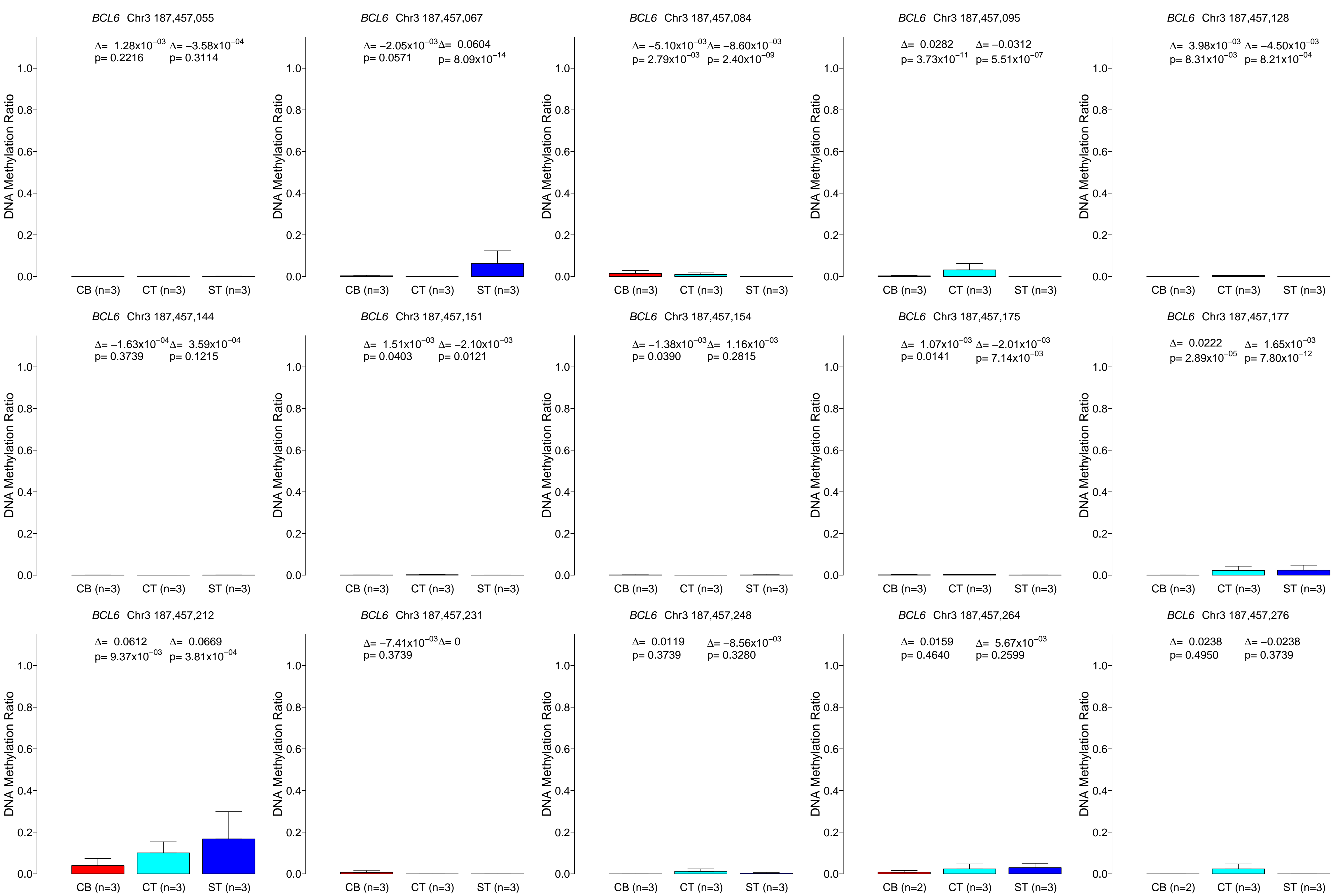

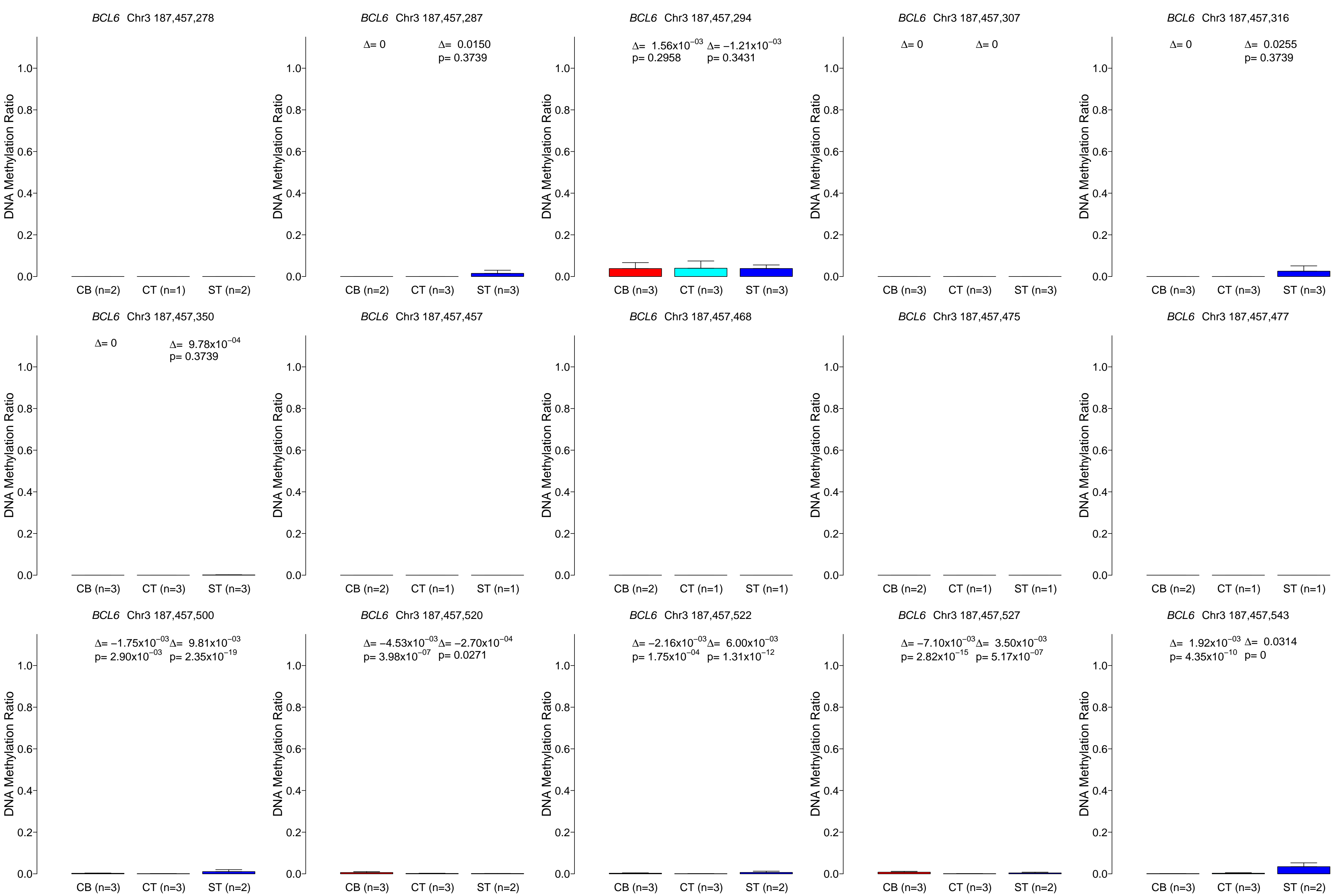

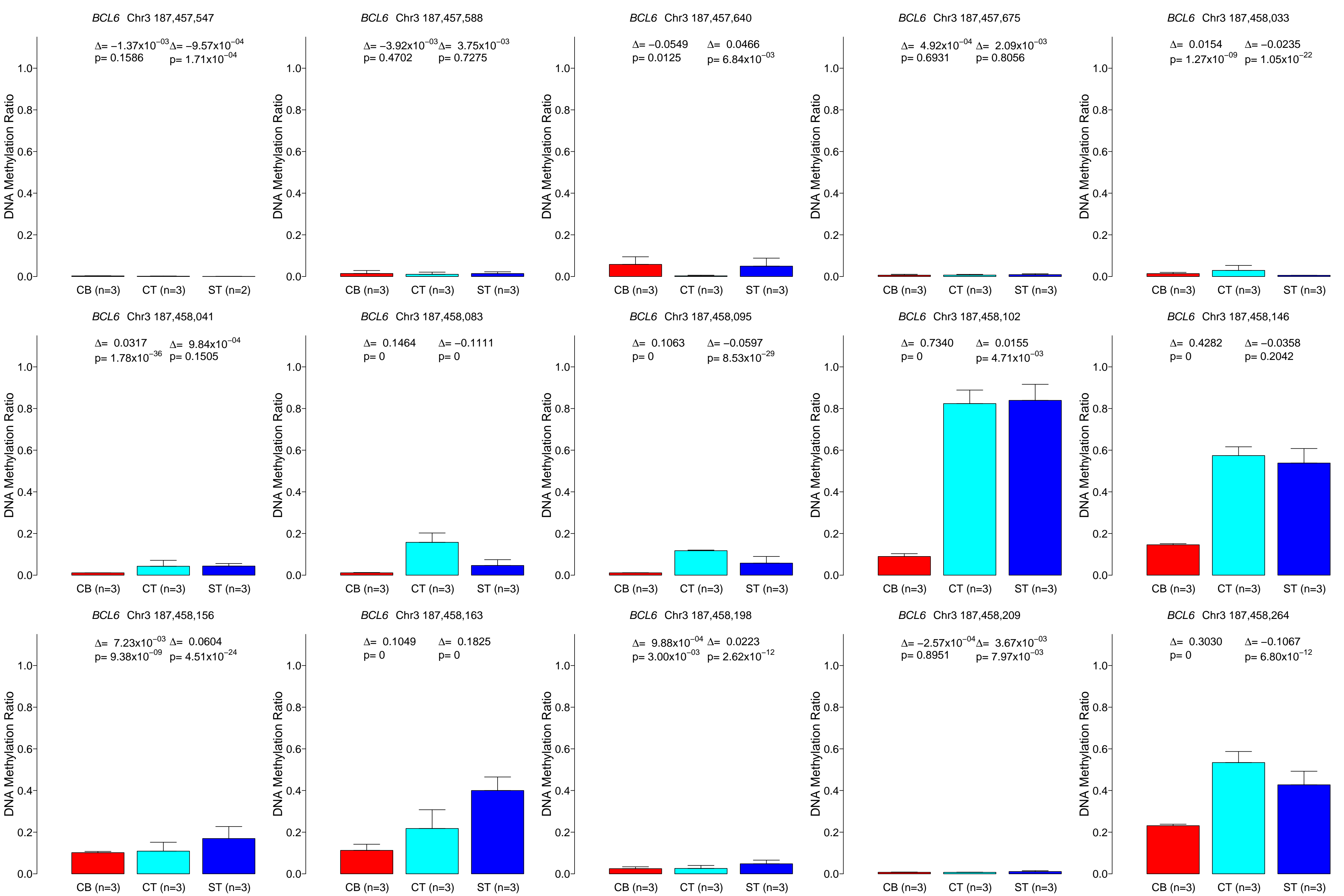

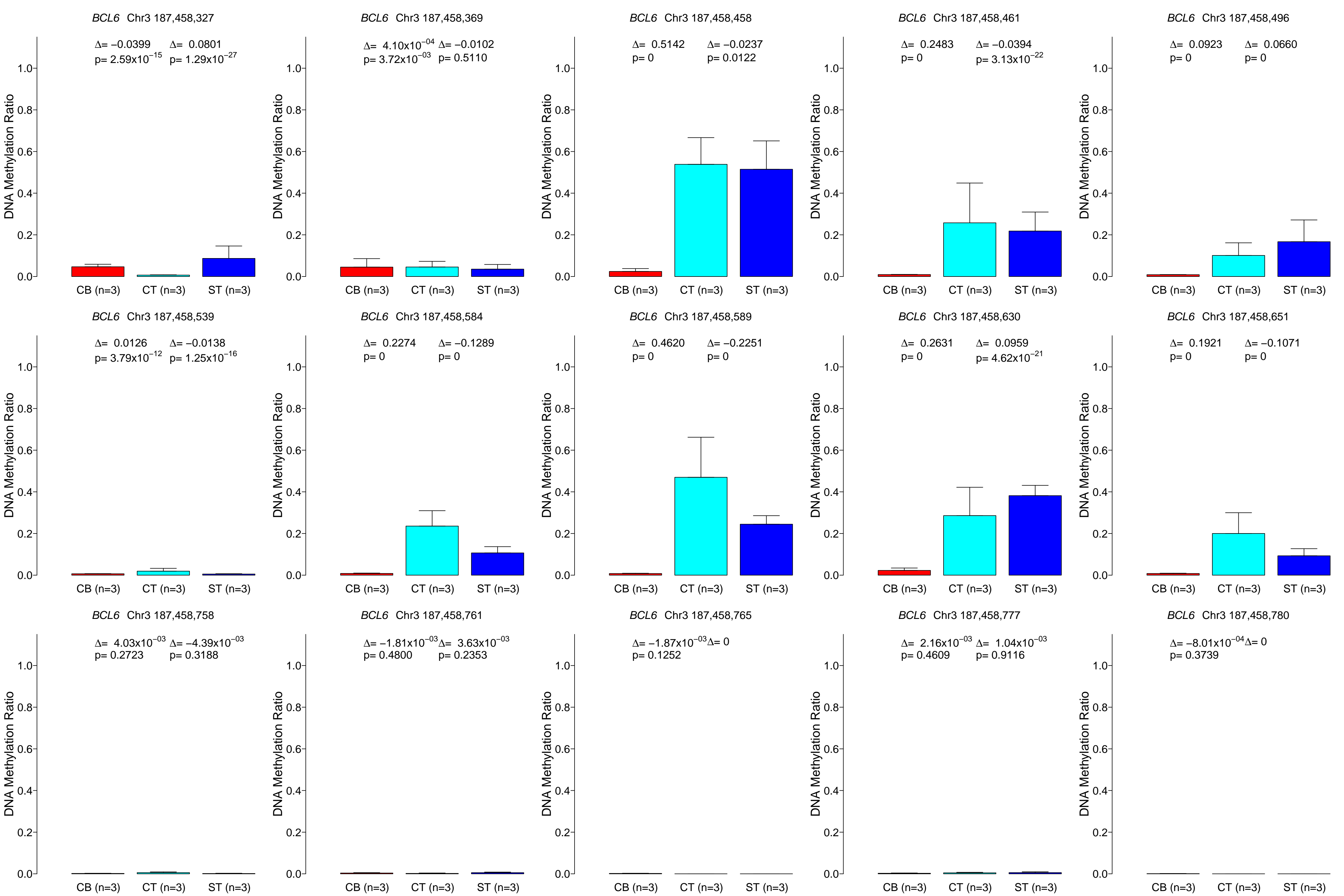

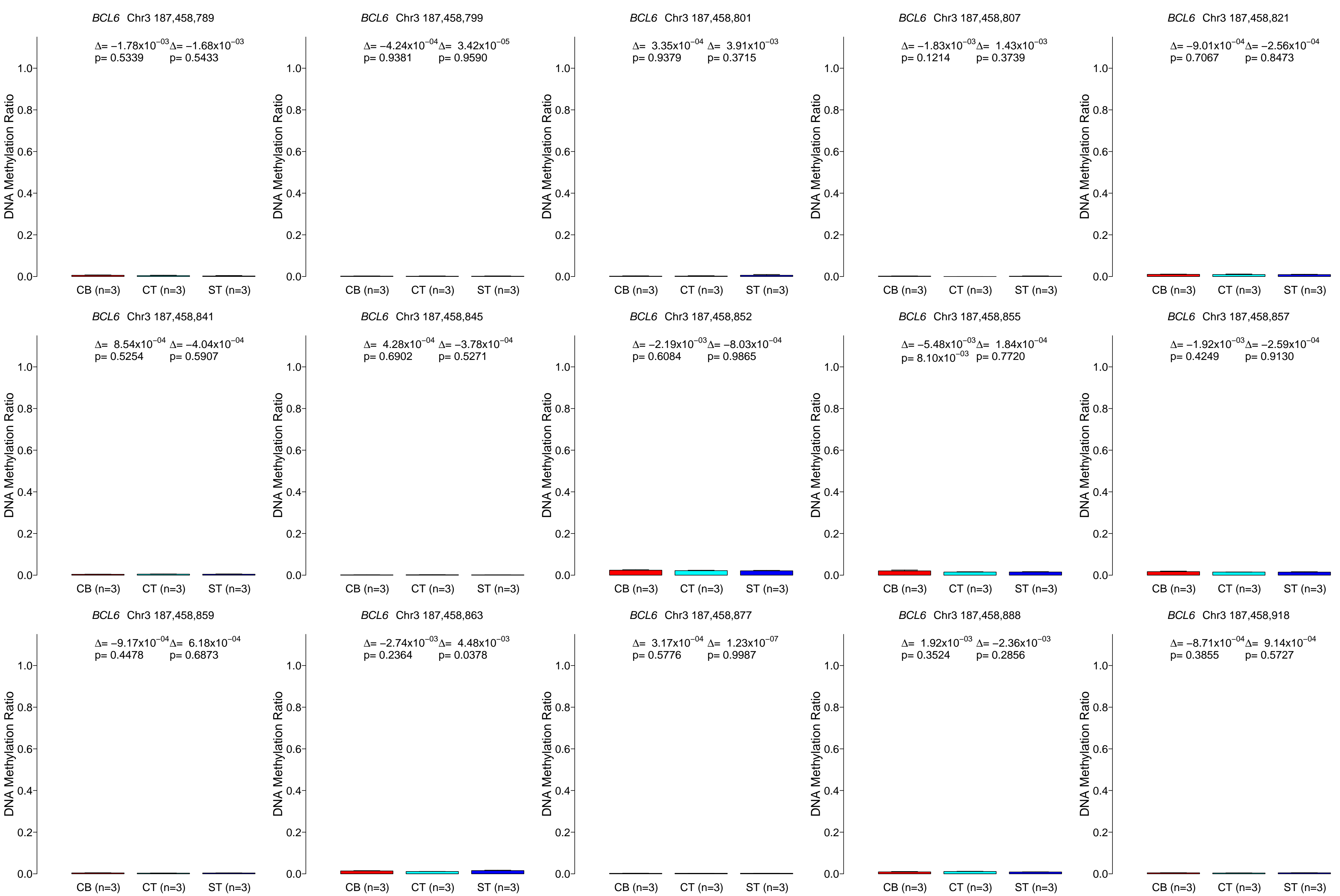

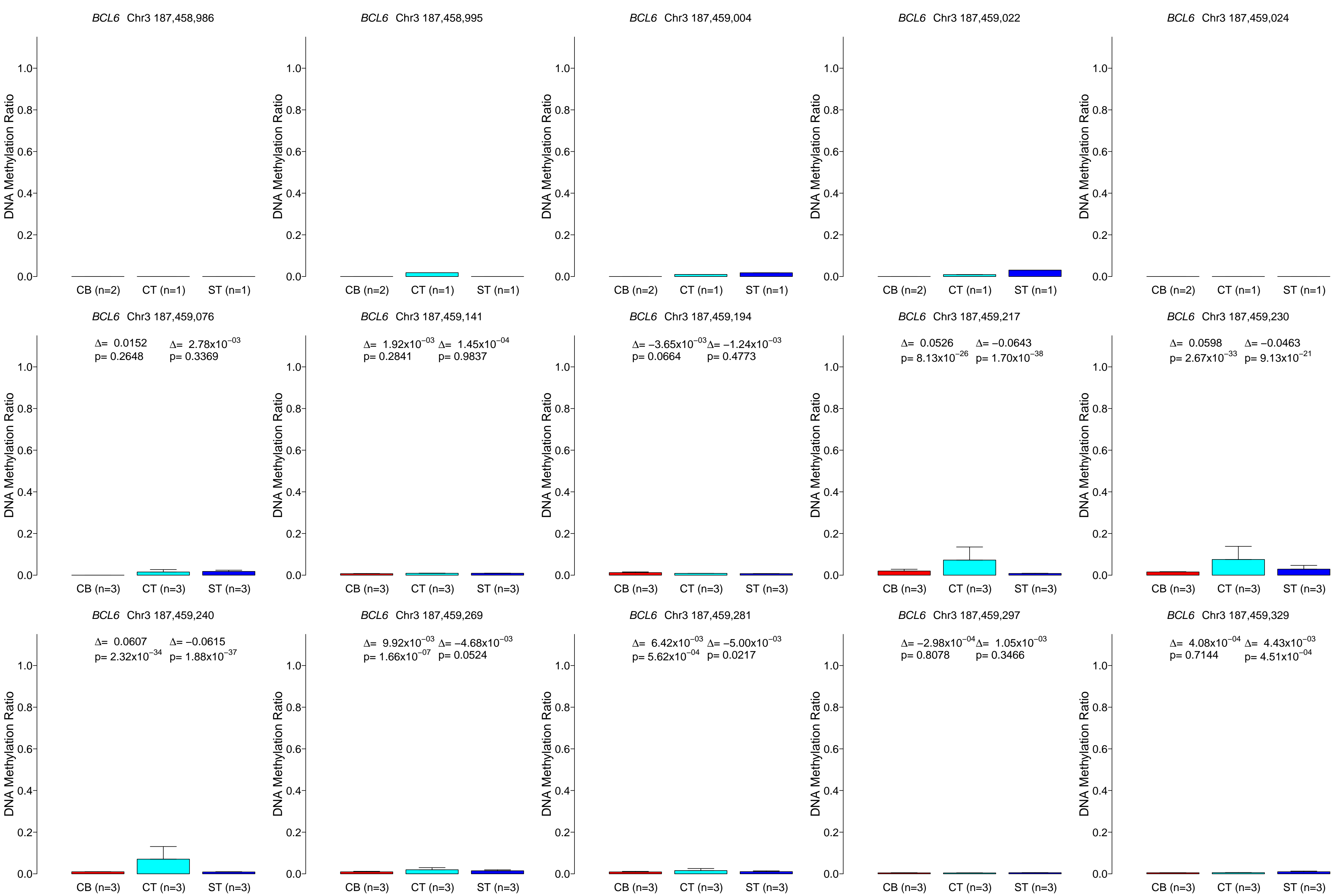

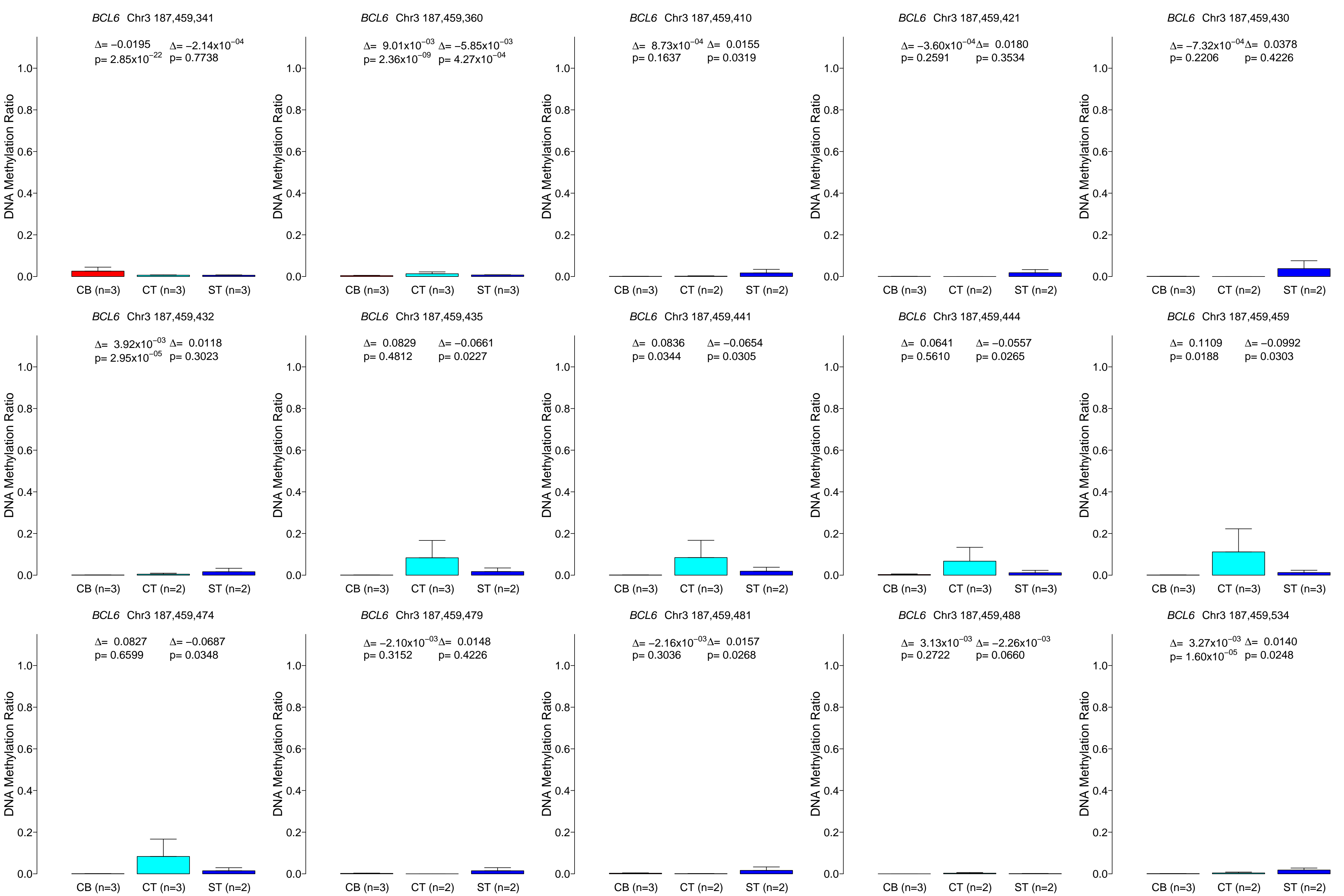

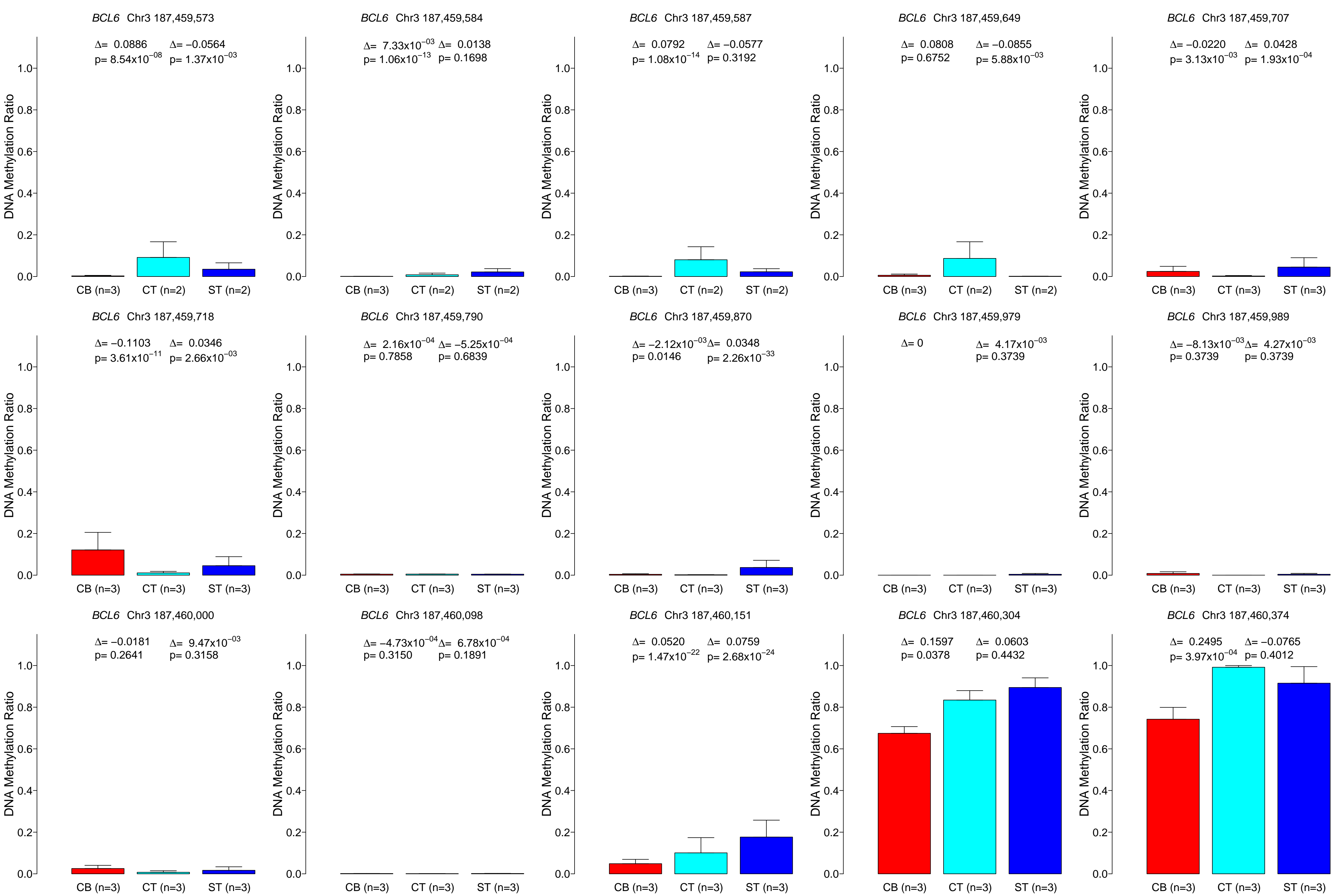

Supplement: Figure S9 — DNA methylation levels at individual CpGs in BCL6 in the trophoblast and umbilical cord blood cells. DNA methylation levels (0–100%) at individual CpGs in BCL6 in umbilical cord blood cells (CB), cytotrophoblasts (CT), and differentiated syncytiotrophoblasts (ST) are depicted in the bar plots that represent means and SEs. Umbilical cord blood cells and cytotrophoblasts were obtained from the same fetuses. The genomic coordinates of the CpGs, the group differences (CB vs. CT; CT vs. ST) in mean DNA methylation levels and the p-values are shown above the bar plots. The number of samples analyzed with methylation reads above the threshold are shown below the bar plots (only comparisons with a group sample size of minimum two were considered). Differential methylation was claimed to be mild, moderate, or strong when the p-value was <0.05 and the difference in methylation level was ≥0.125, ≥0.25, or ≥0.5, respectively. [file Image_9.pdf]

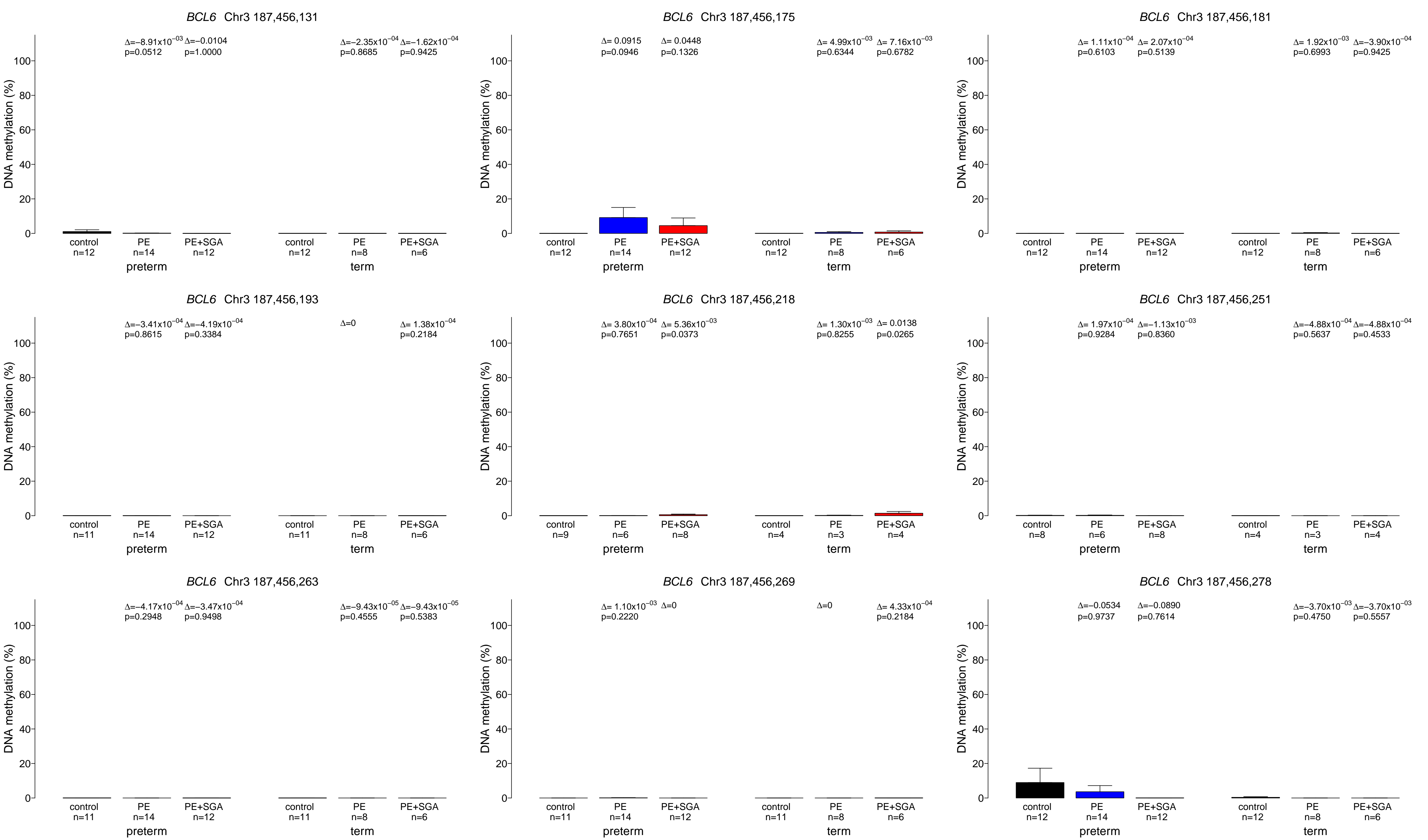

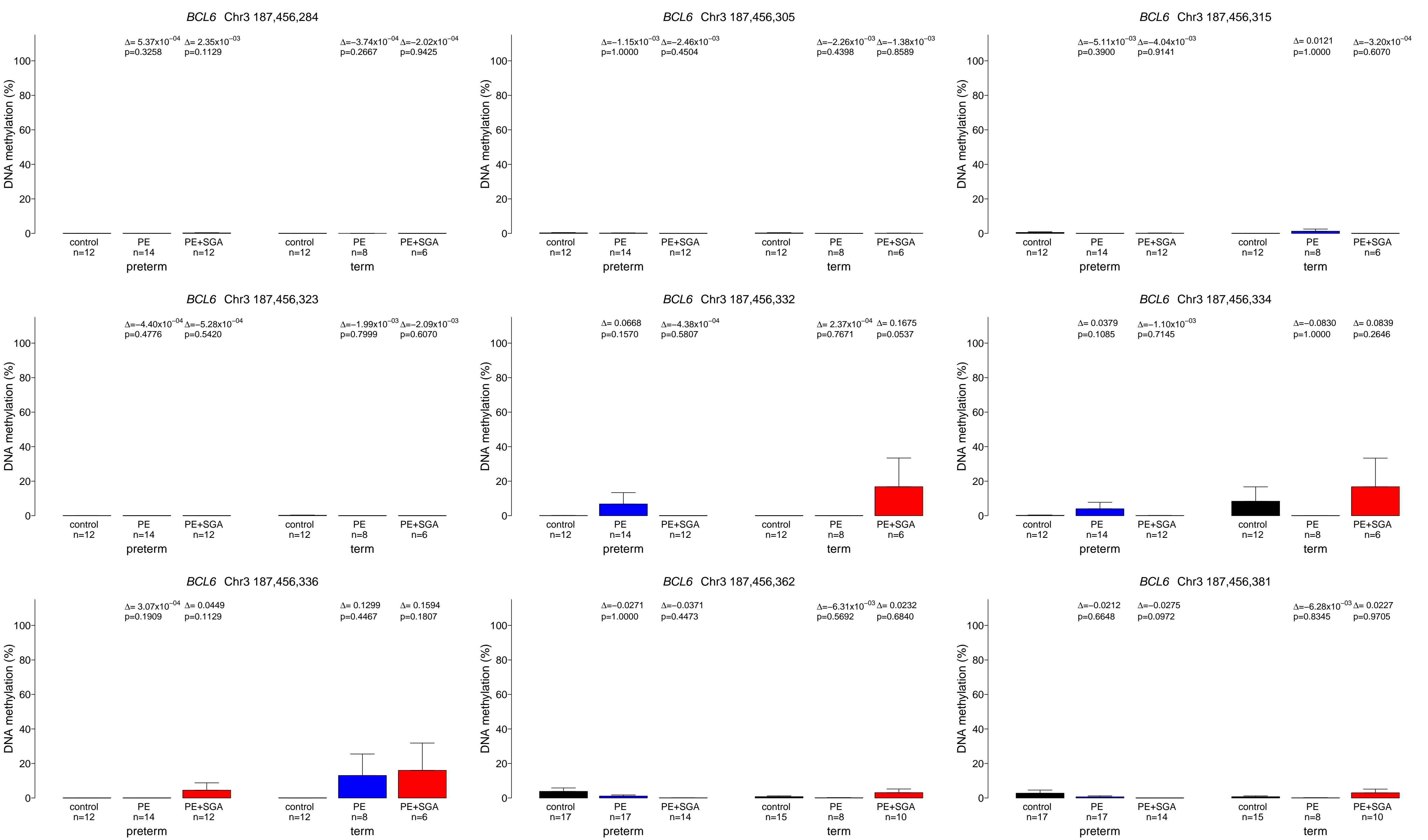

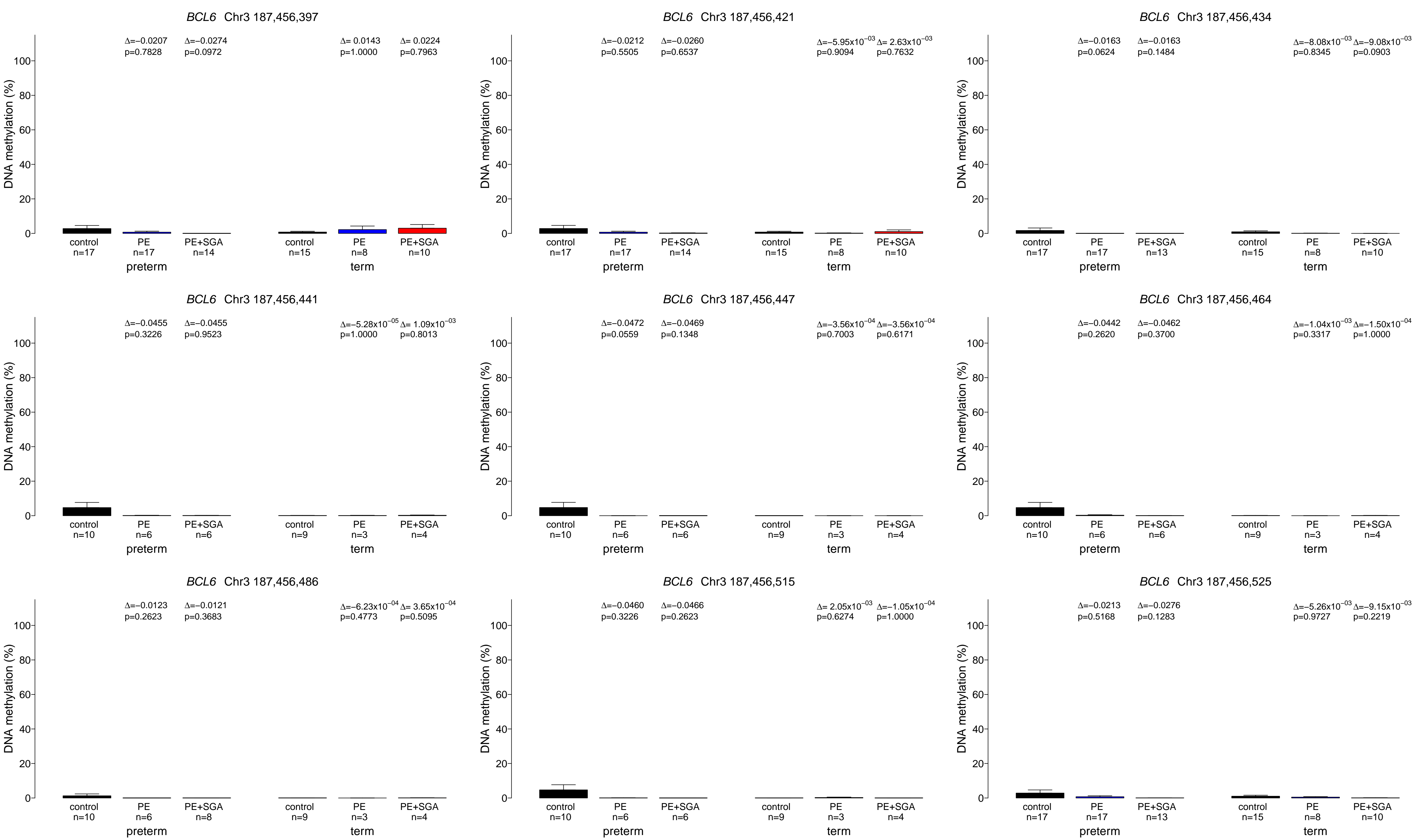

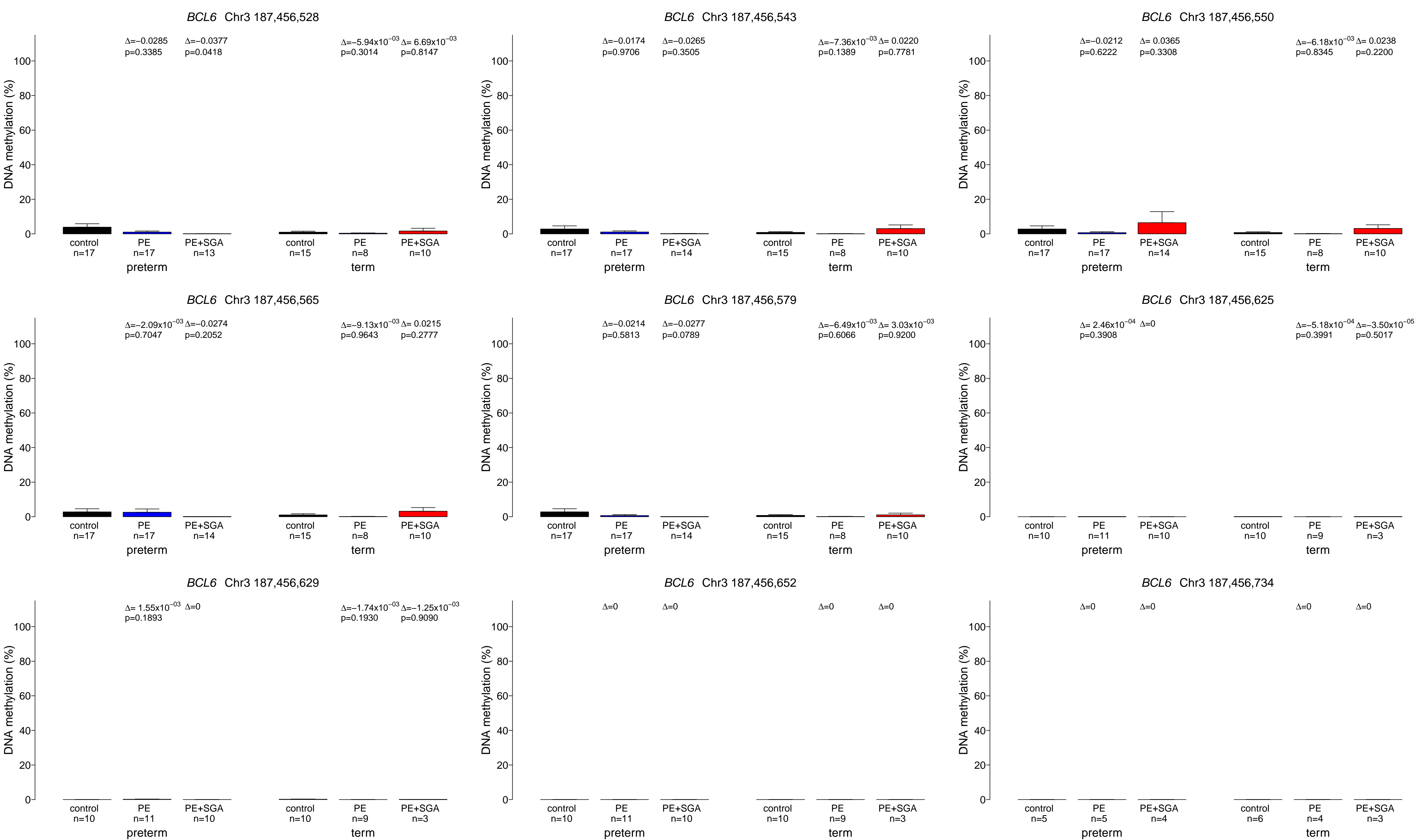

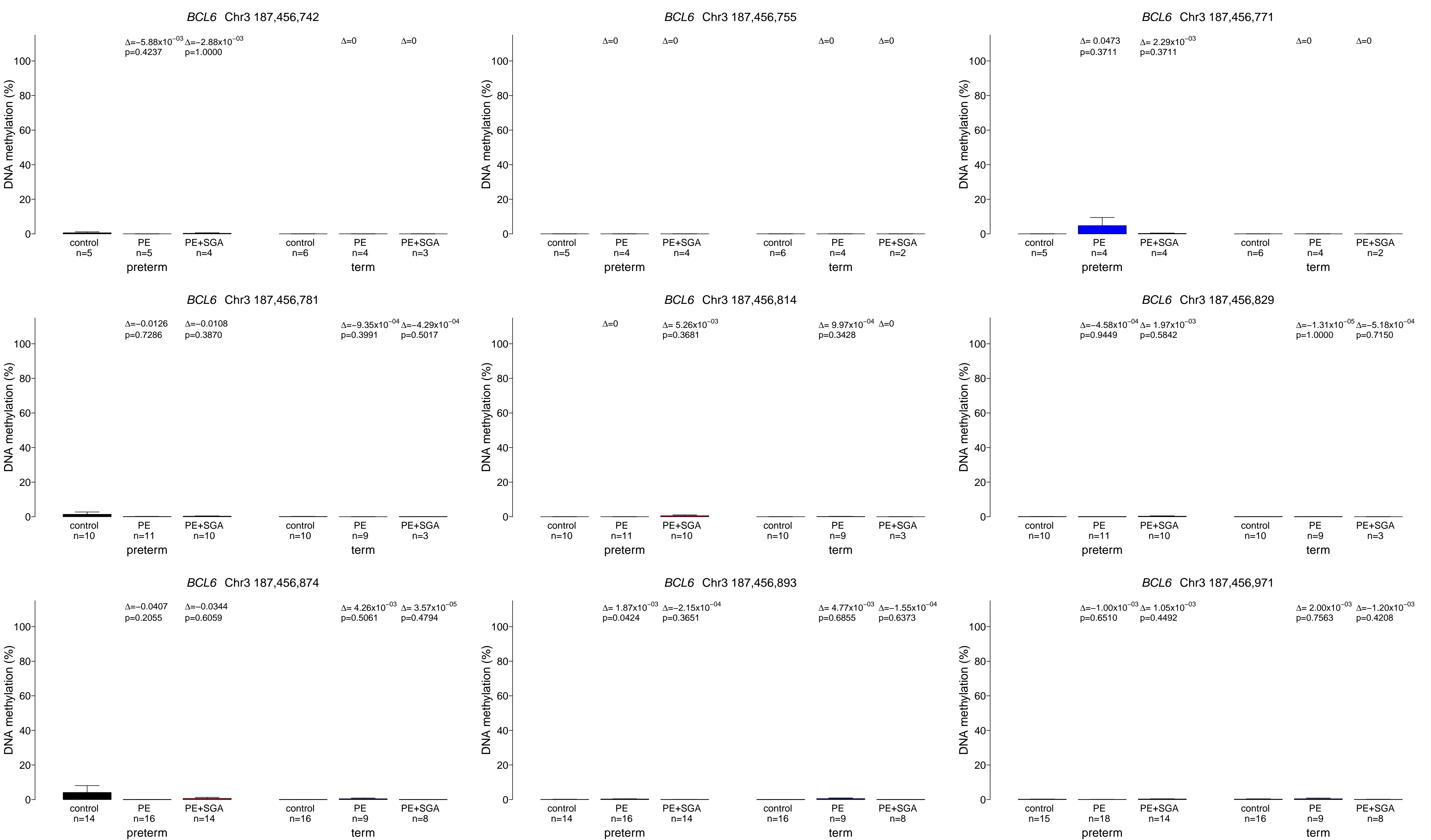

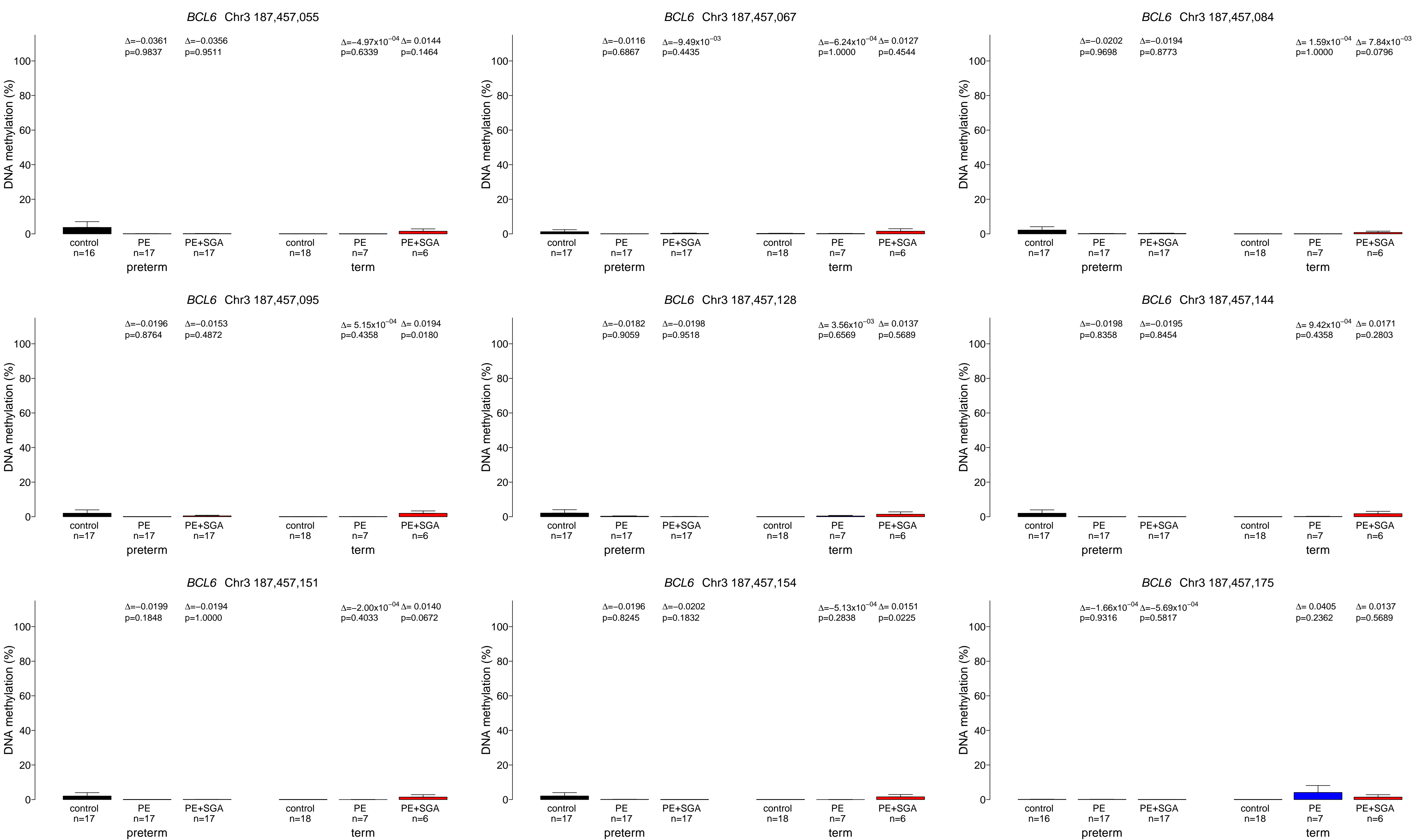

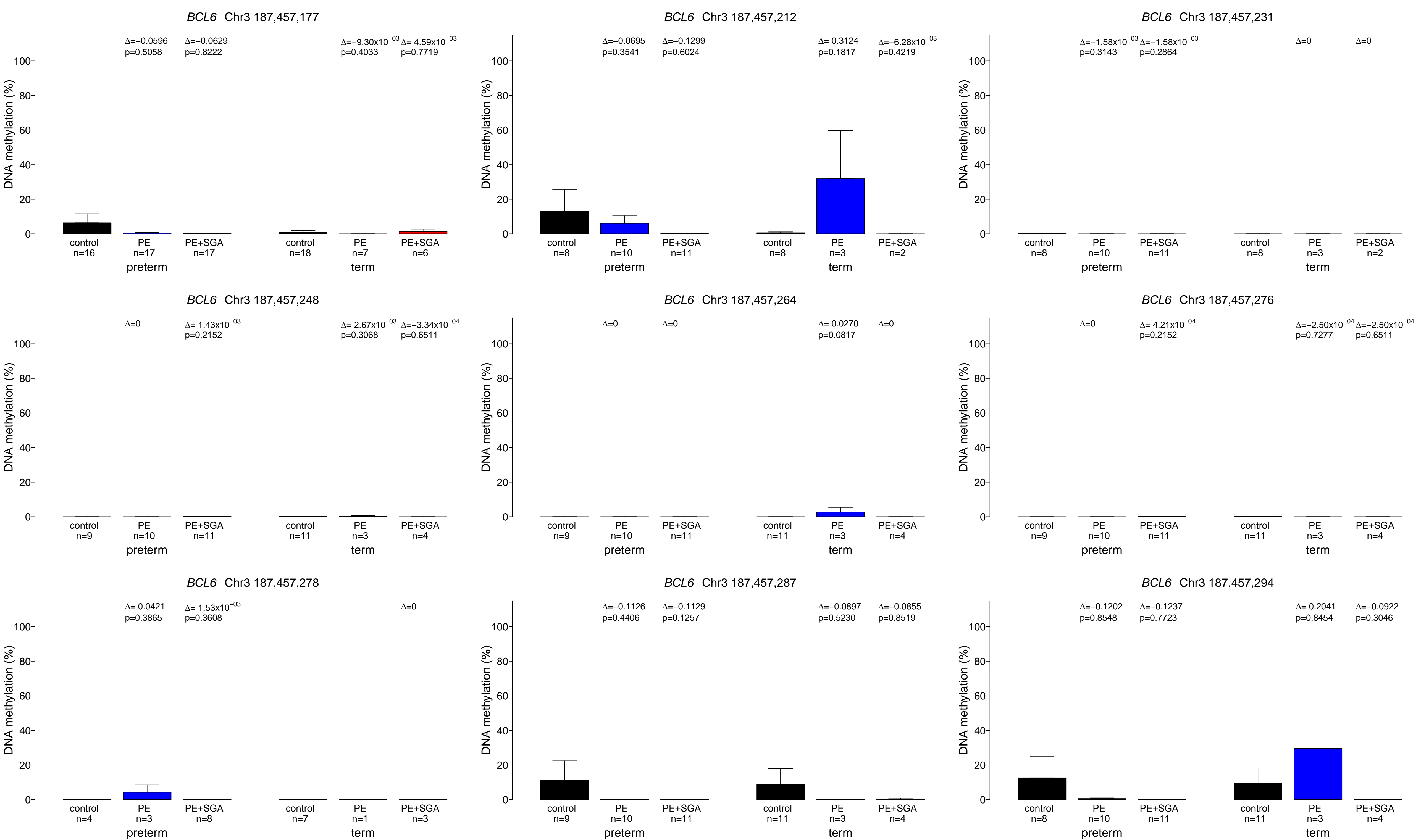

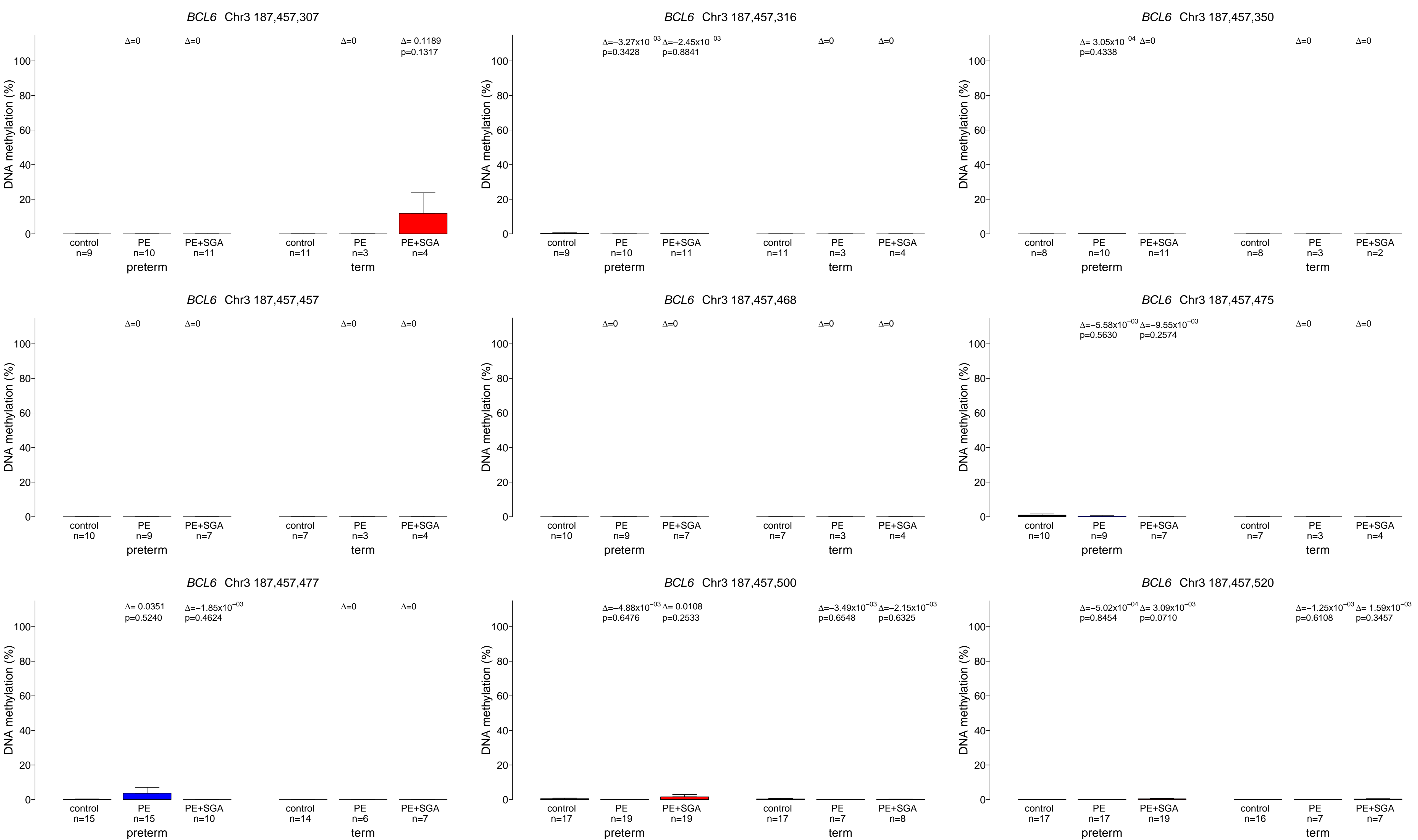

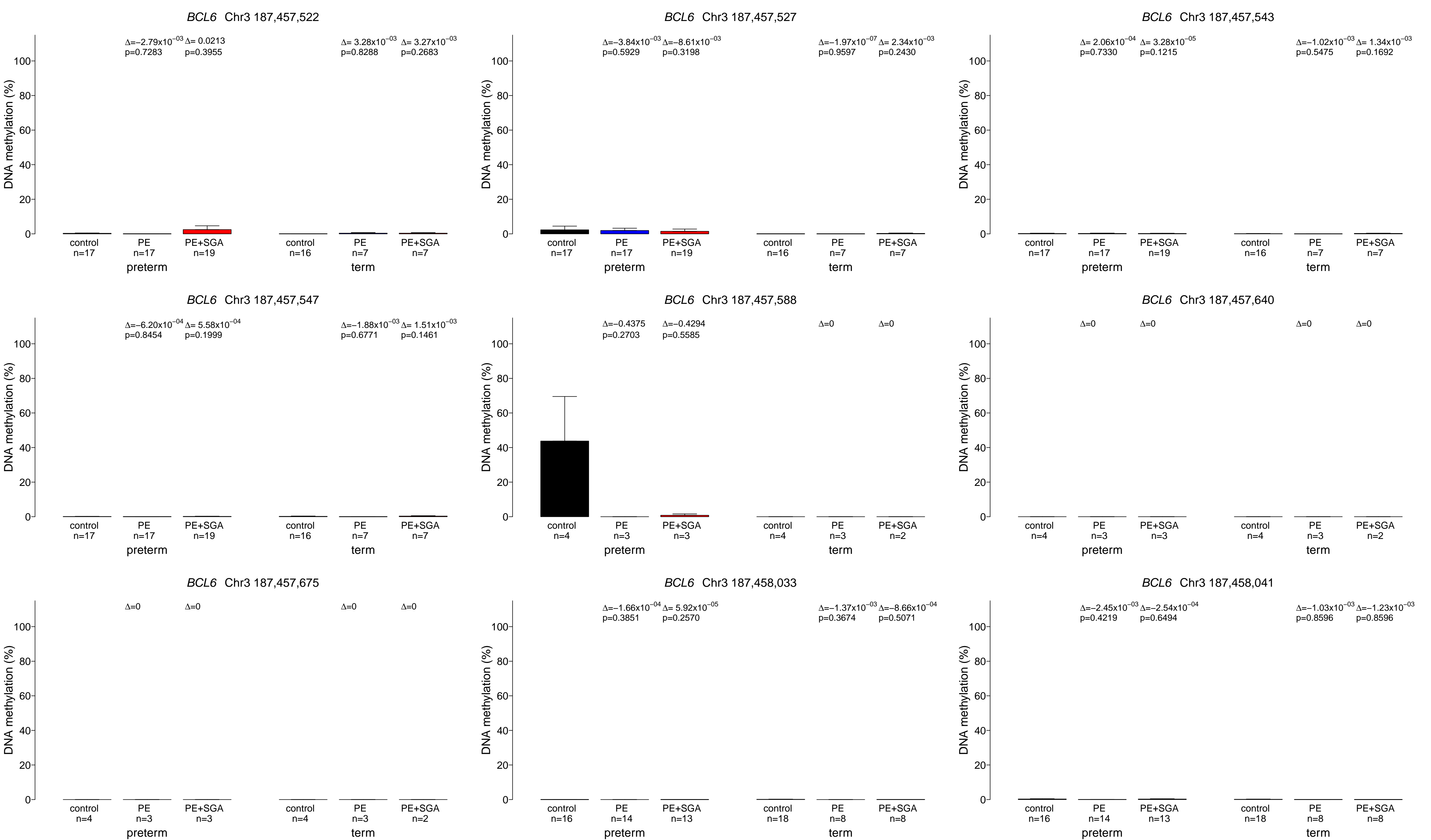

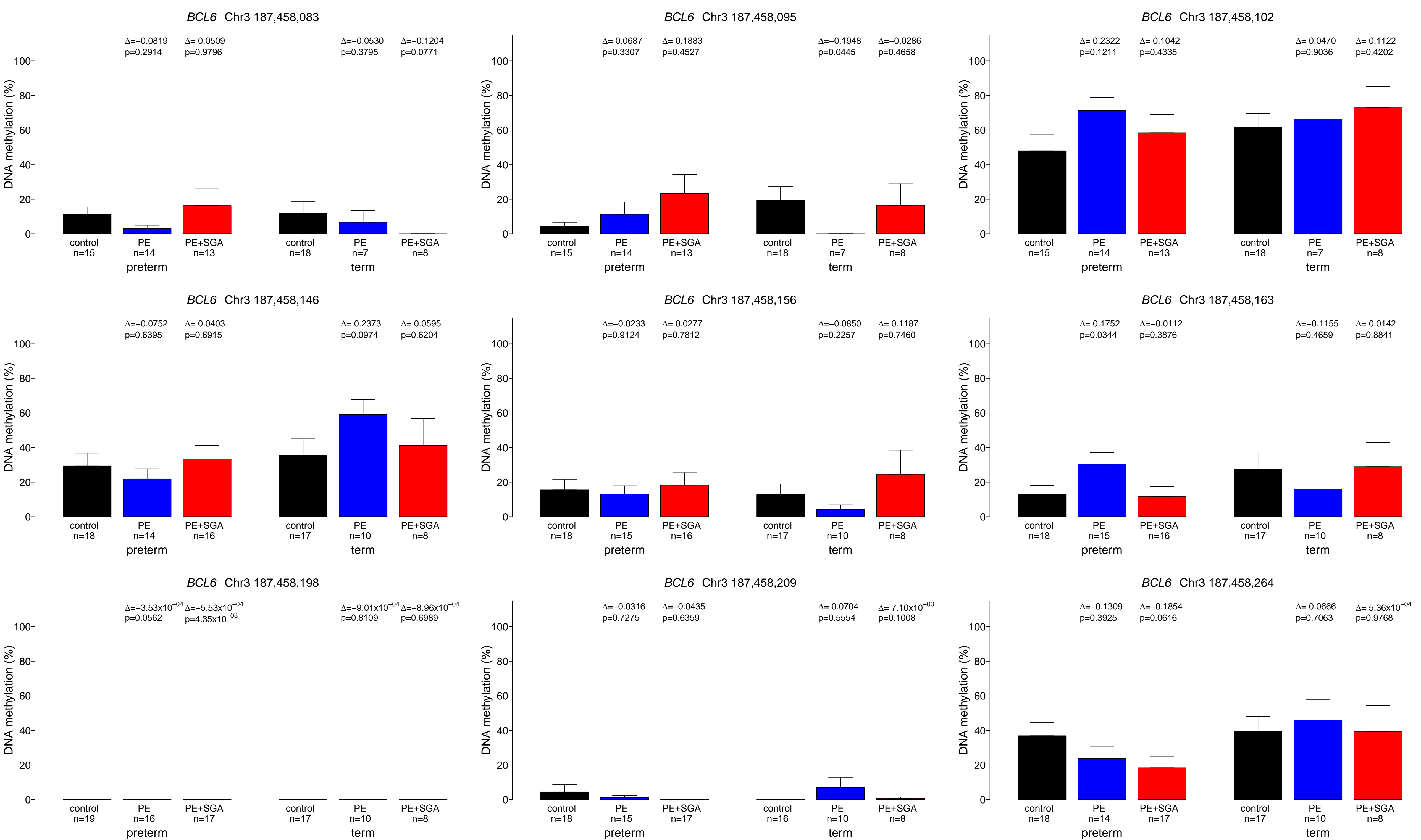

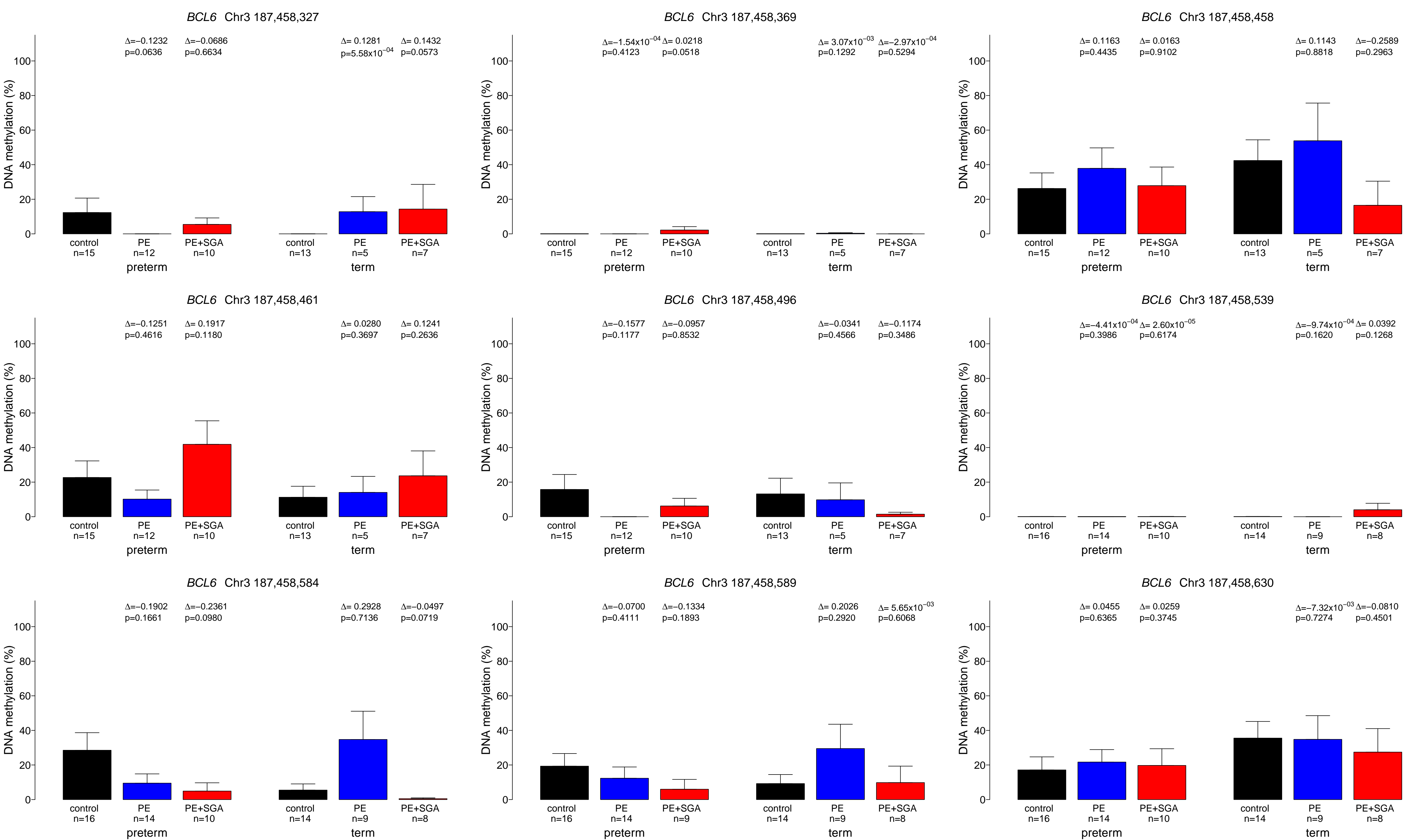

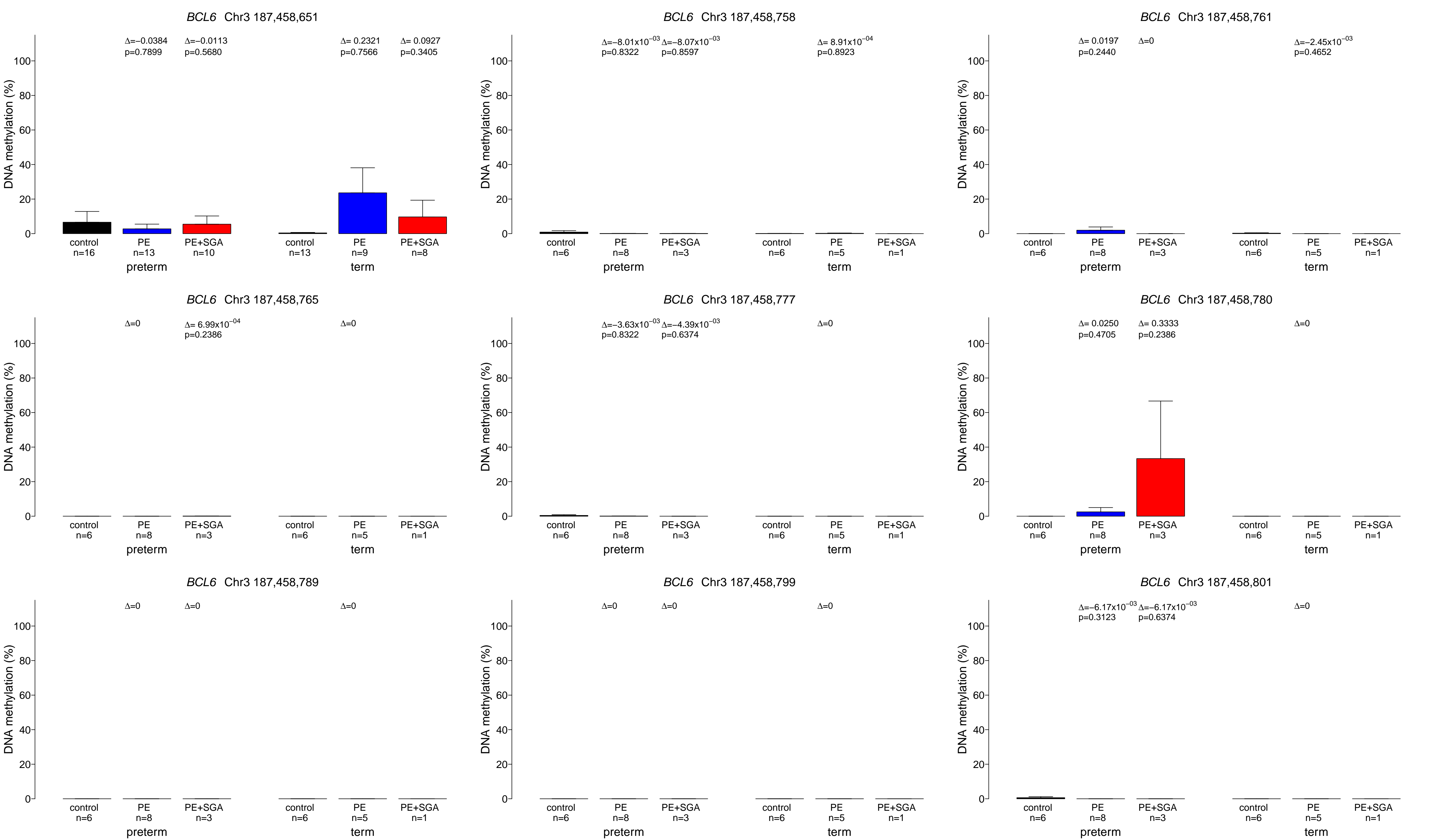

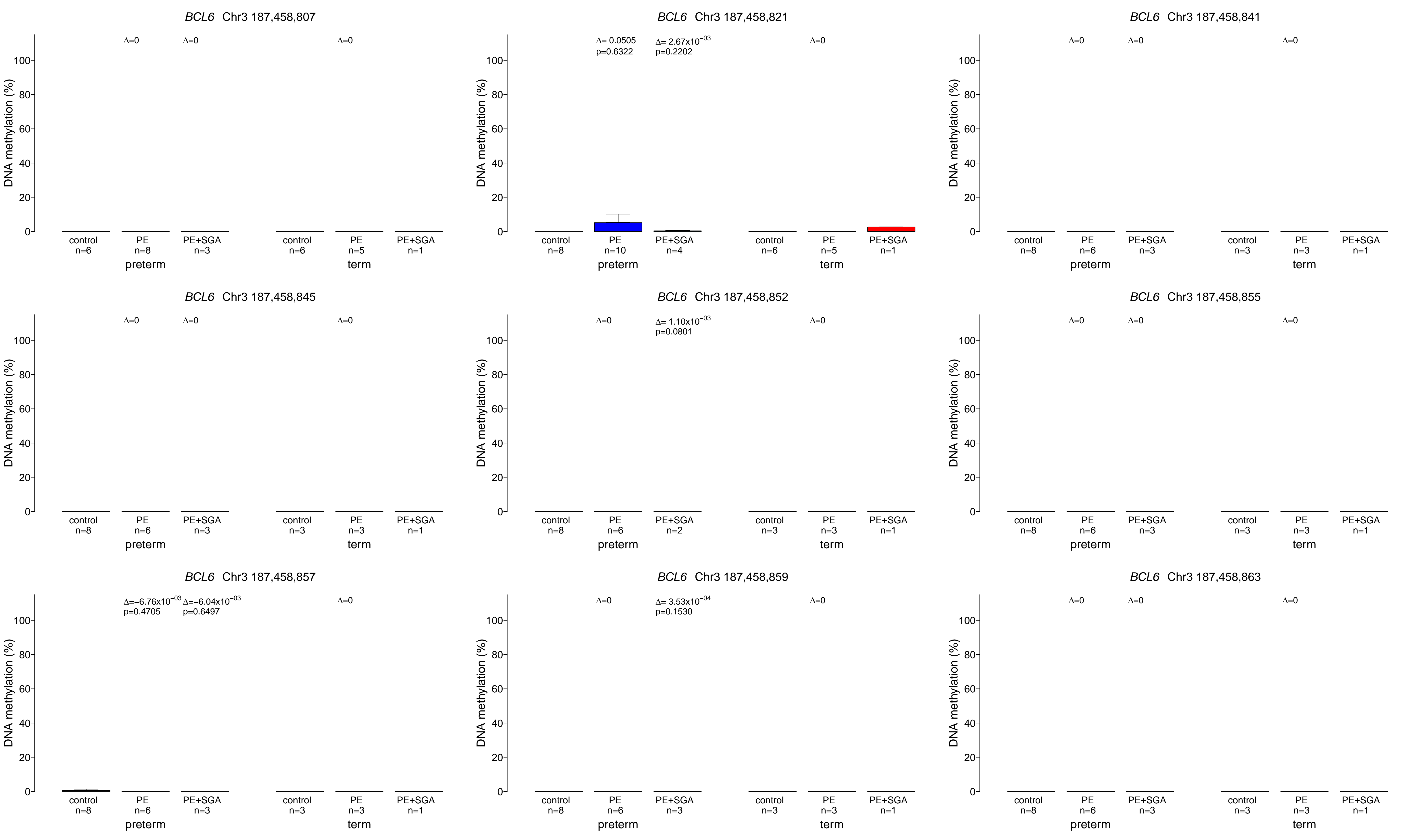

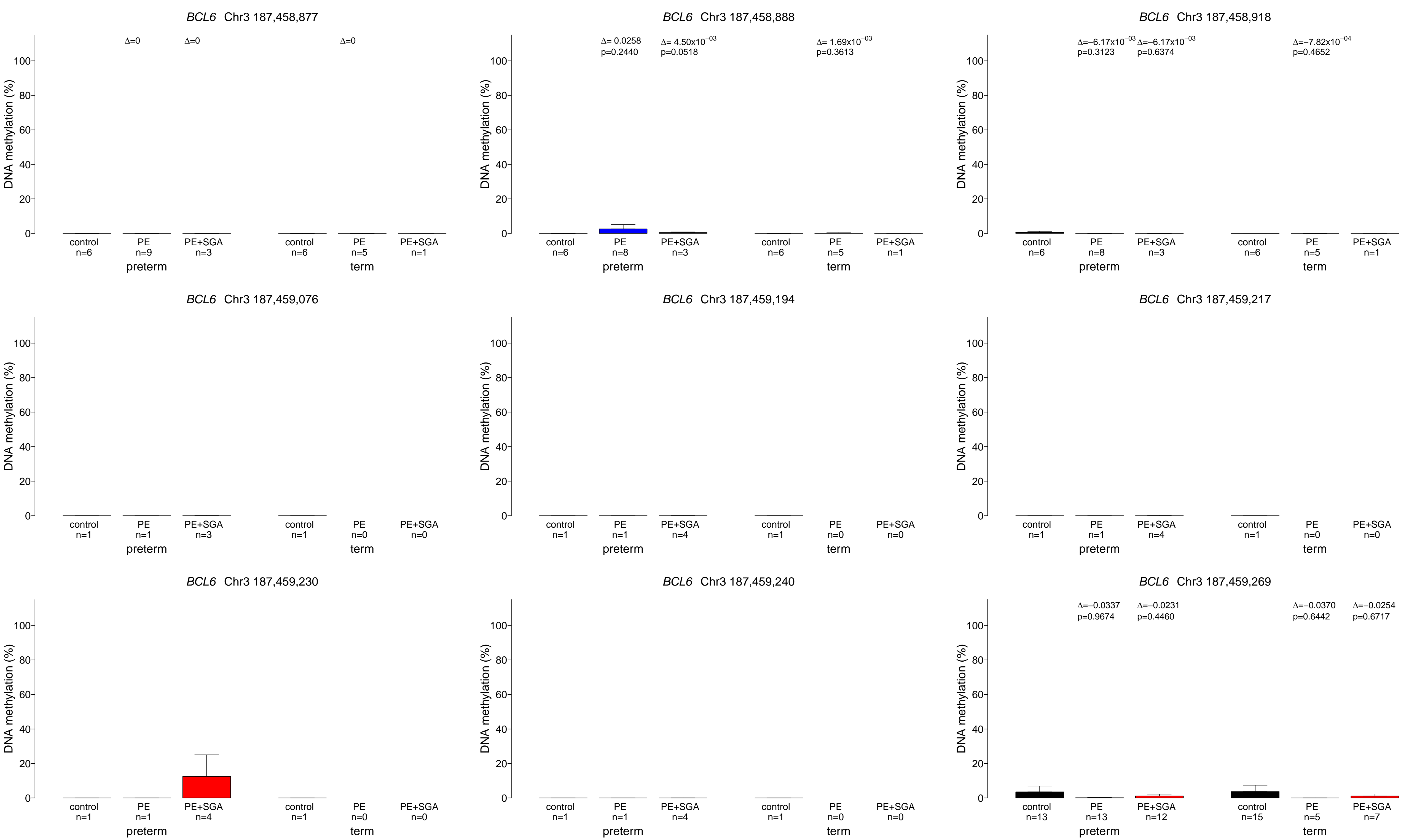

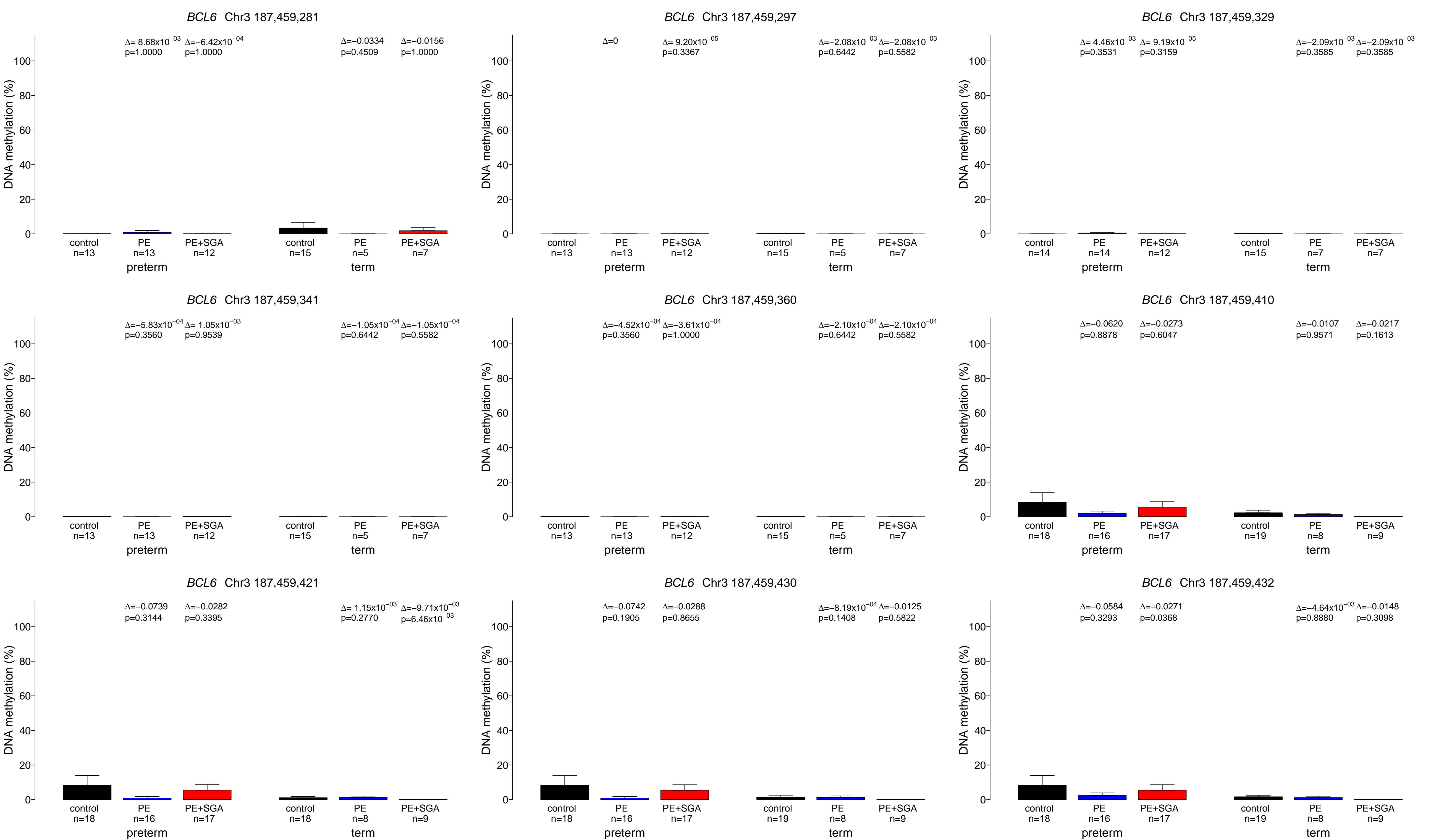

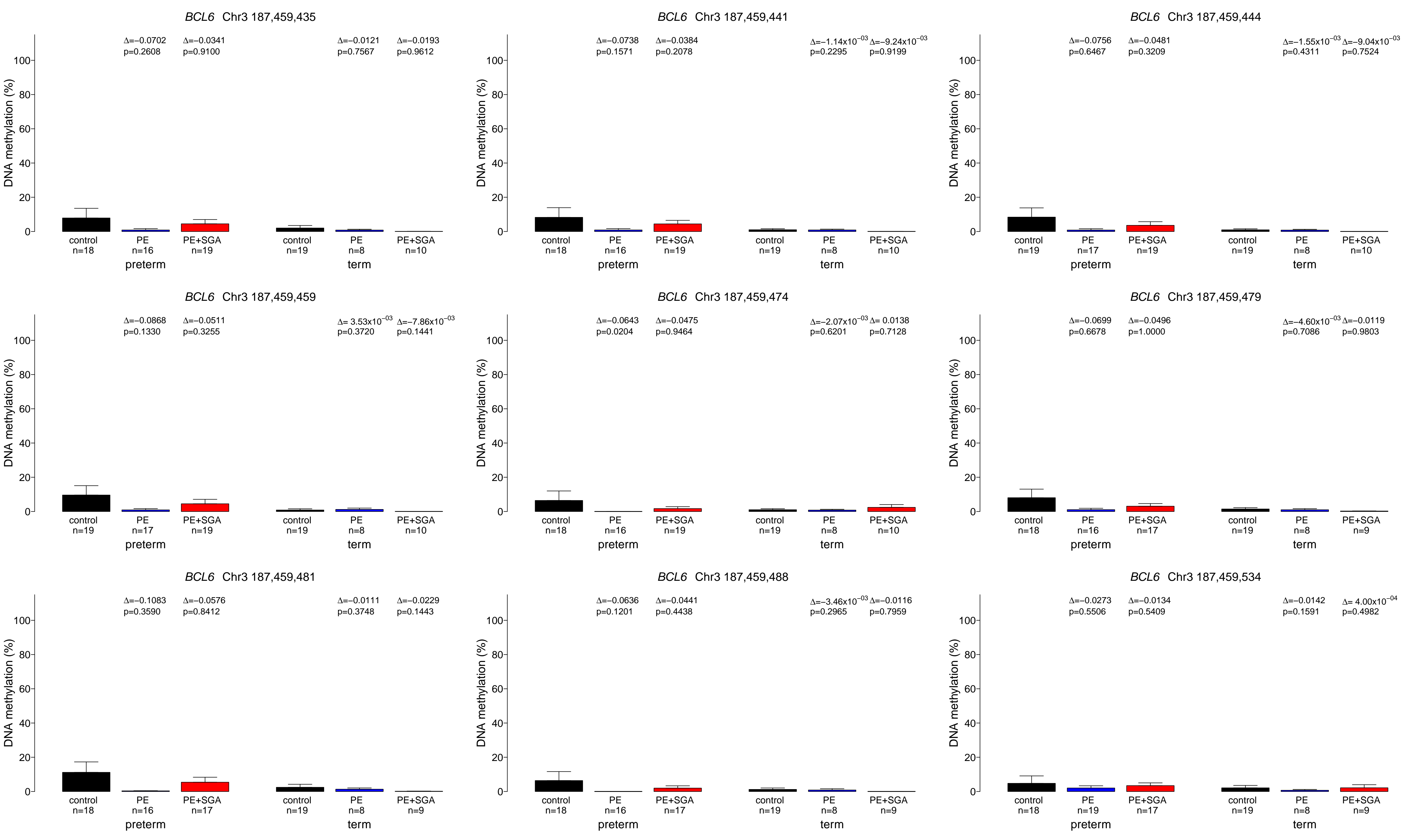

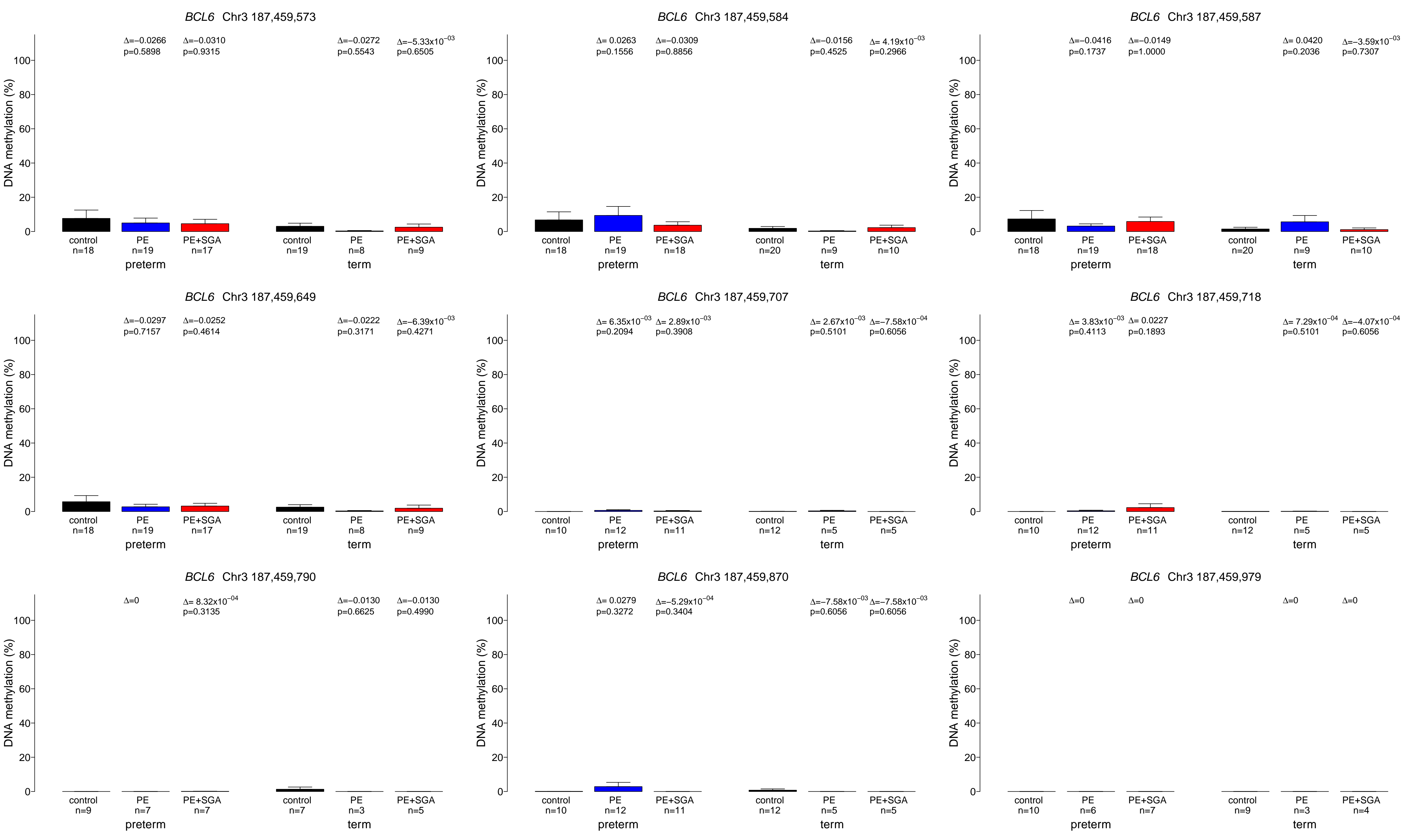

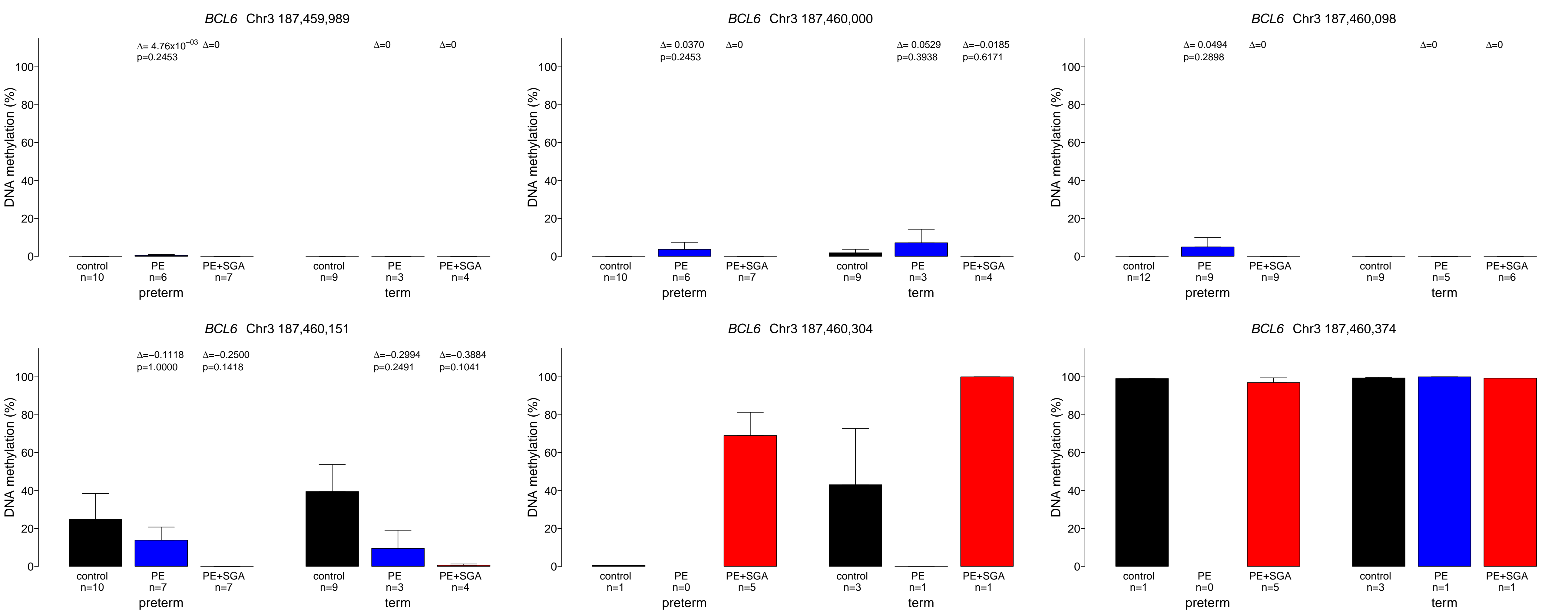

Supplement: Figure S10 — DNA methylation levels at individual CpGs in BCL6 in the trophoblast in controls and in cases of preeclampsia. DNA methylation levels (0–100%) at individual CpGs in BCL6 in laser captured trophoblasts are depicted in the bar plots that represent means and SEs. The genomic coordinates of the CpGs, the group differences (compared preterm or term controls) in DNA methylation levels and the p-values are shown above the bar plots. The number of samples analyzed with methylation reads above the threshold are shown below the bar plots (only comparisons with a group sample size of minimum four were considered). Differential methylation was claimed to be mild, moderate, or strong when the p-value was <0.05 and the difference in methylation level was ≥0.125, ≥0.25, or ≥0.5, respectively. Preterm (left) and term (right) groups of patients were analyzed separately. Abbreviations: PE, preeclampsia; PE + SGA, preeclampsia associated with small-for-gestational age. [file Image_10.pdf]

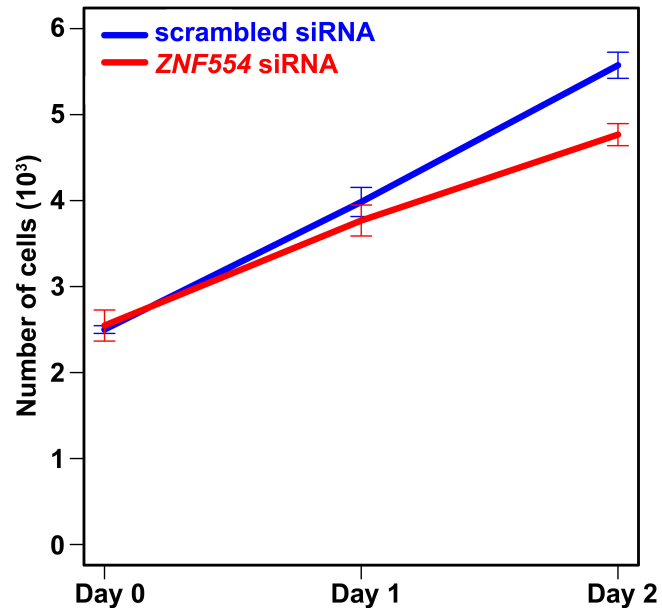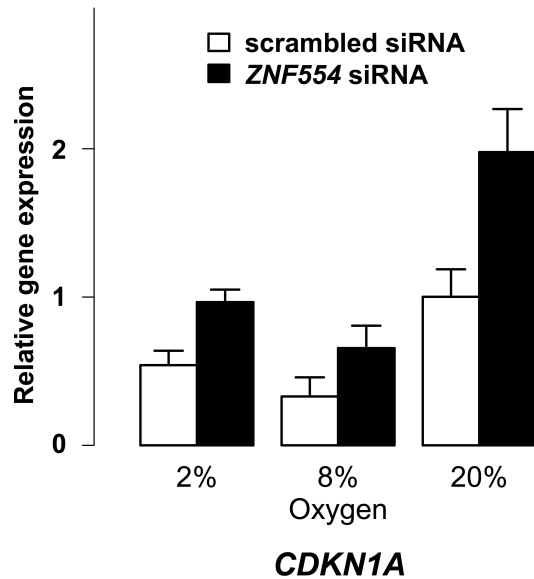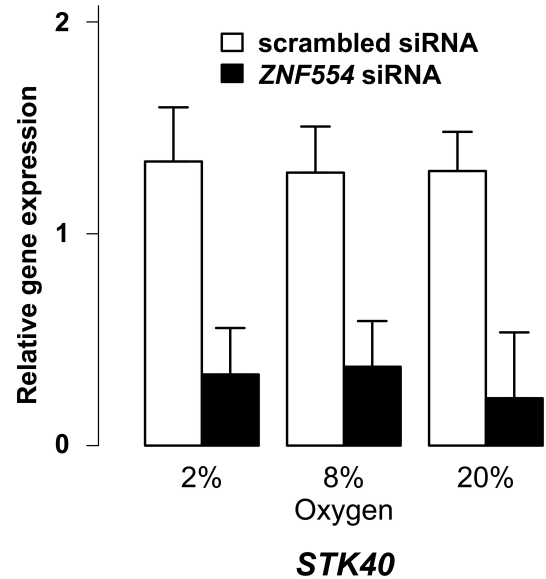

Supplement: Figure S11 — The effect of ZNF554 knock-down on cell proliferation in HTR8/SVneo extravillous trophoblastic cells. (A) Cell proliferation assays showed that ZNF554 knock-down slightly but significantly decreased (−14%, p = 0.02) cell proliferation rate in HTR8/SVneo extravillous trophoblastic cells after 48 h. Y-axis depicts viable cell number, X-axis shows incubation time. (B) The differential expression of CDKN1A (cyclin-dependent kinase inhibitor 1A) and STK40 (serine/threonine kinase 40), genes involved in the regulation of cell cycle, upon ZNF554 knock-down was confirmed by qRT-PCR. [file Image_11.pdf]

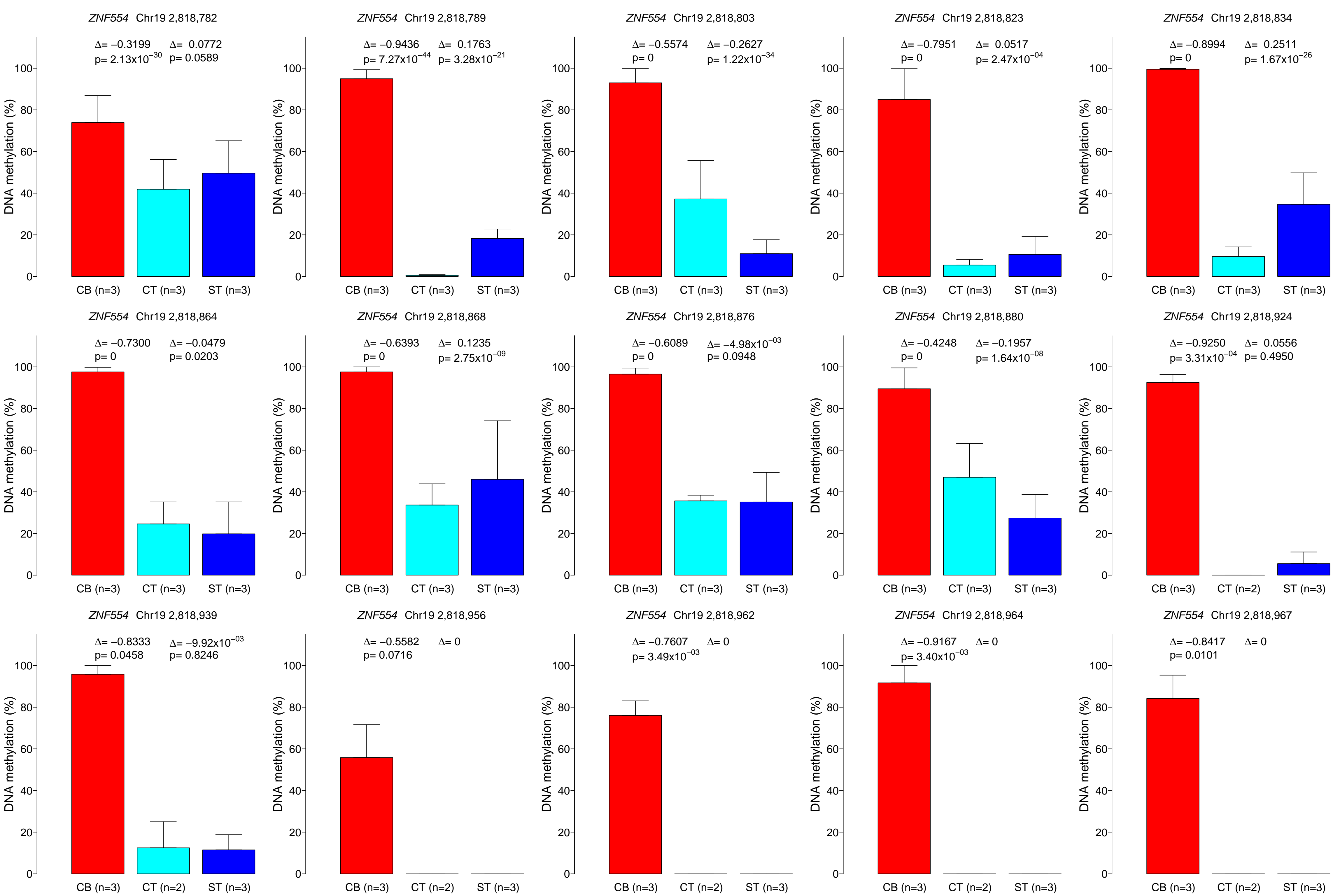

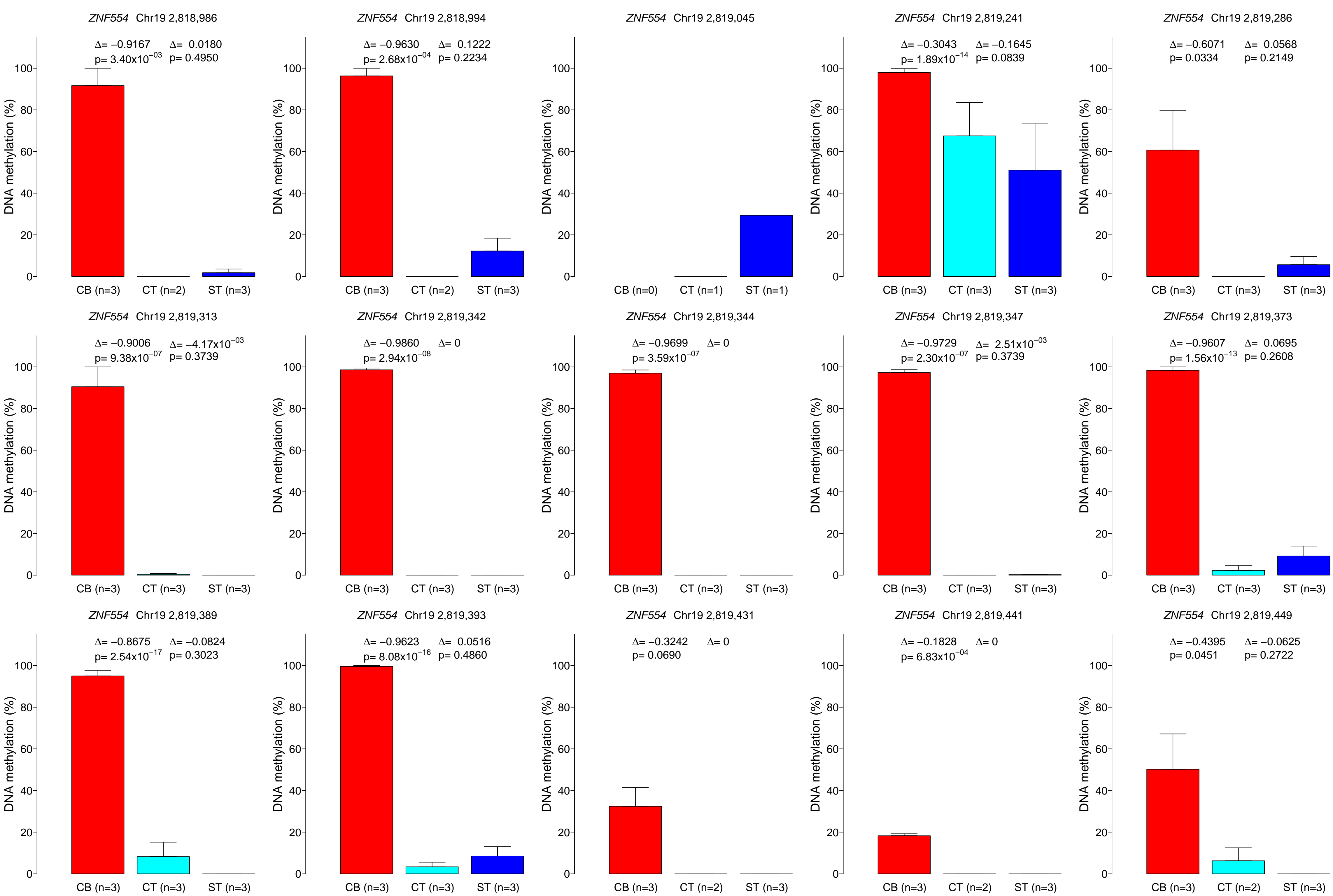

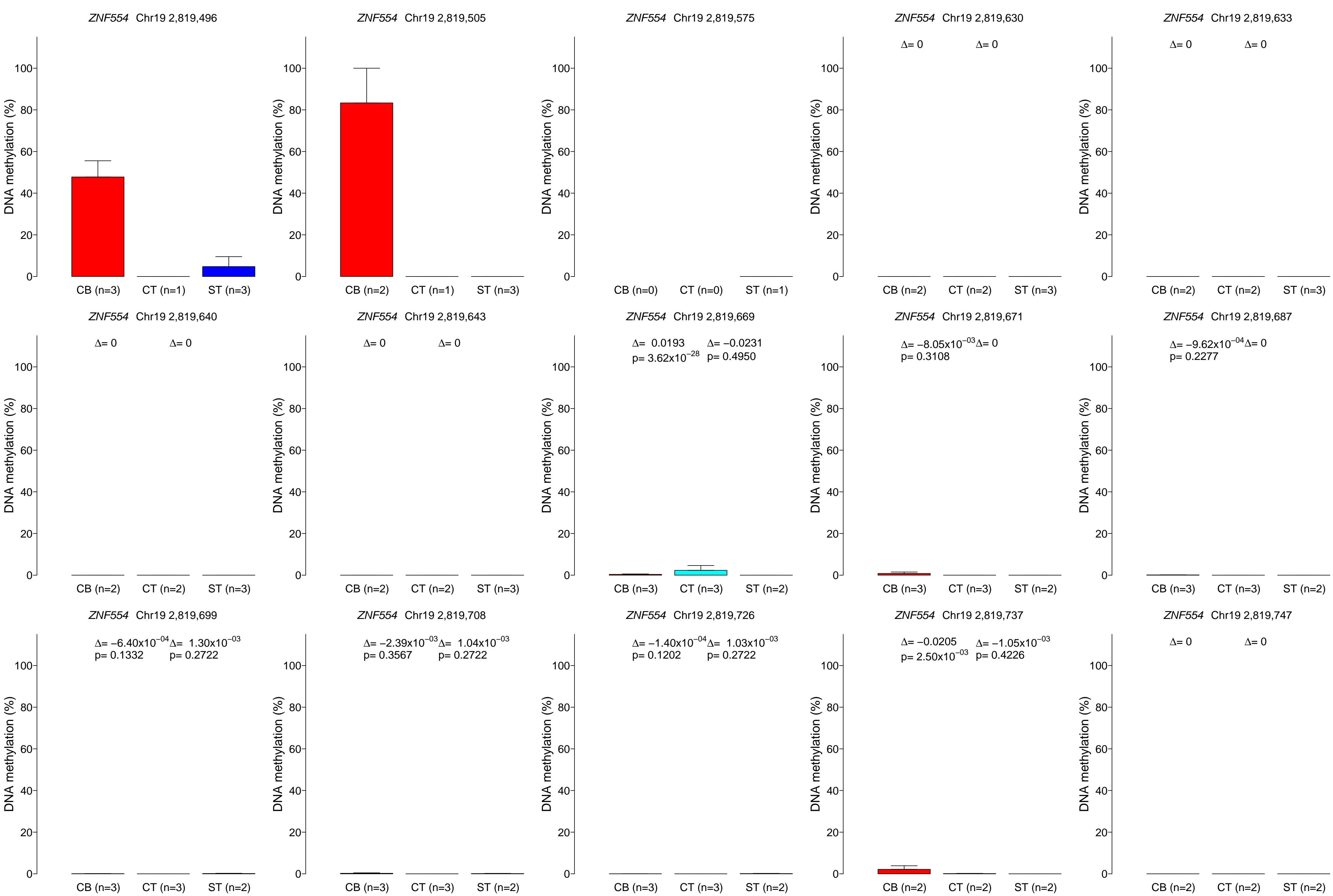

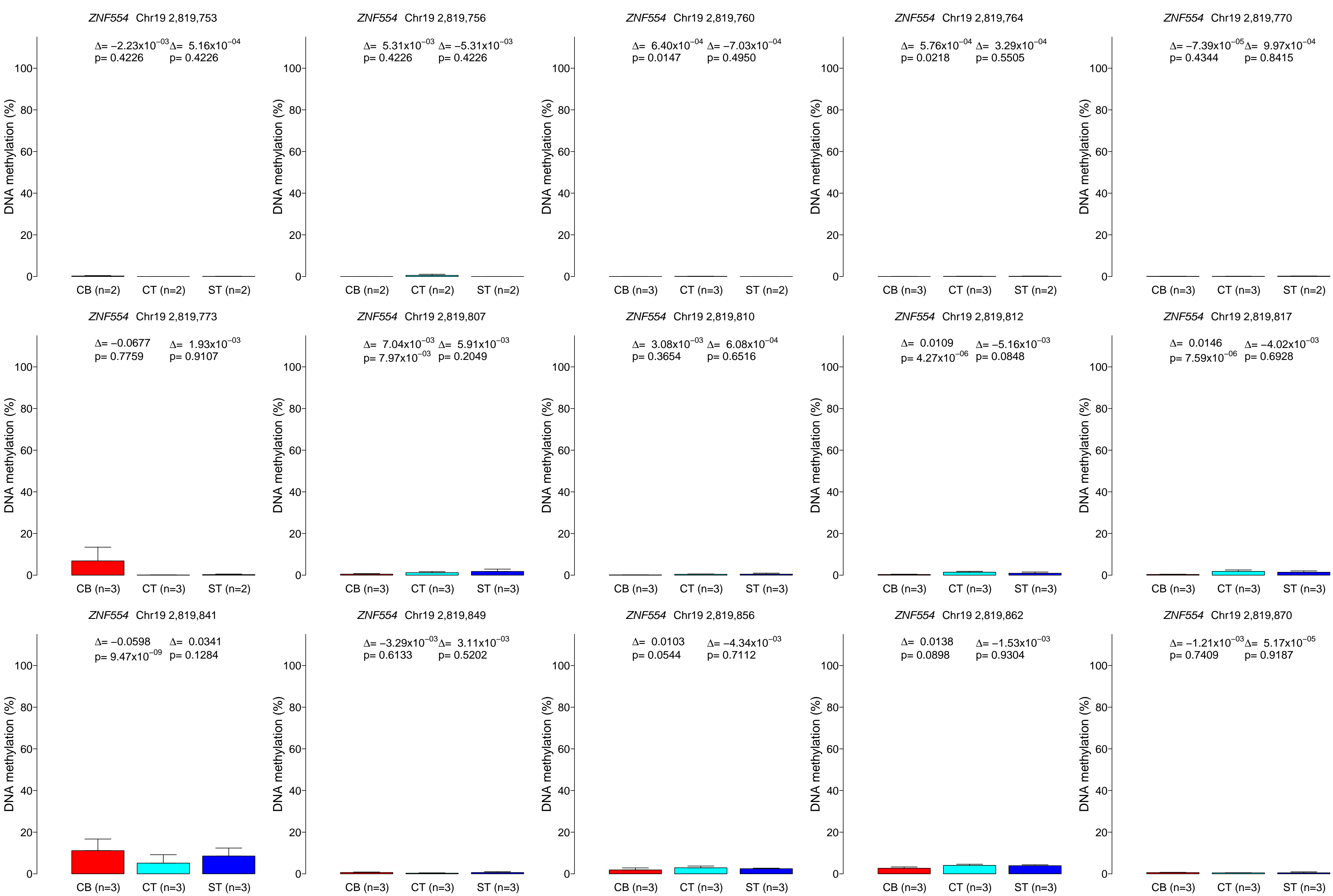

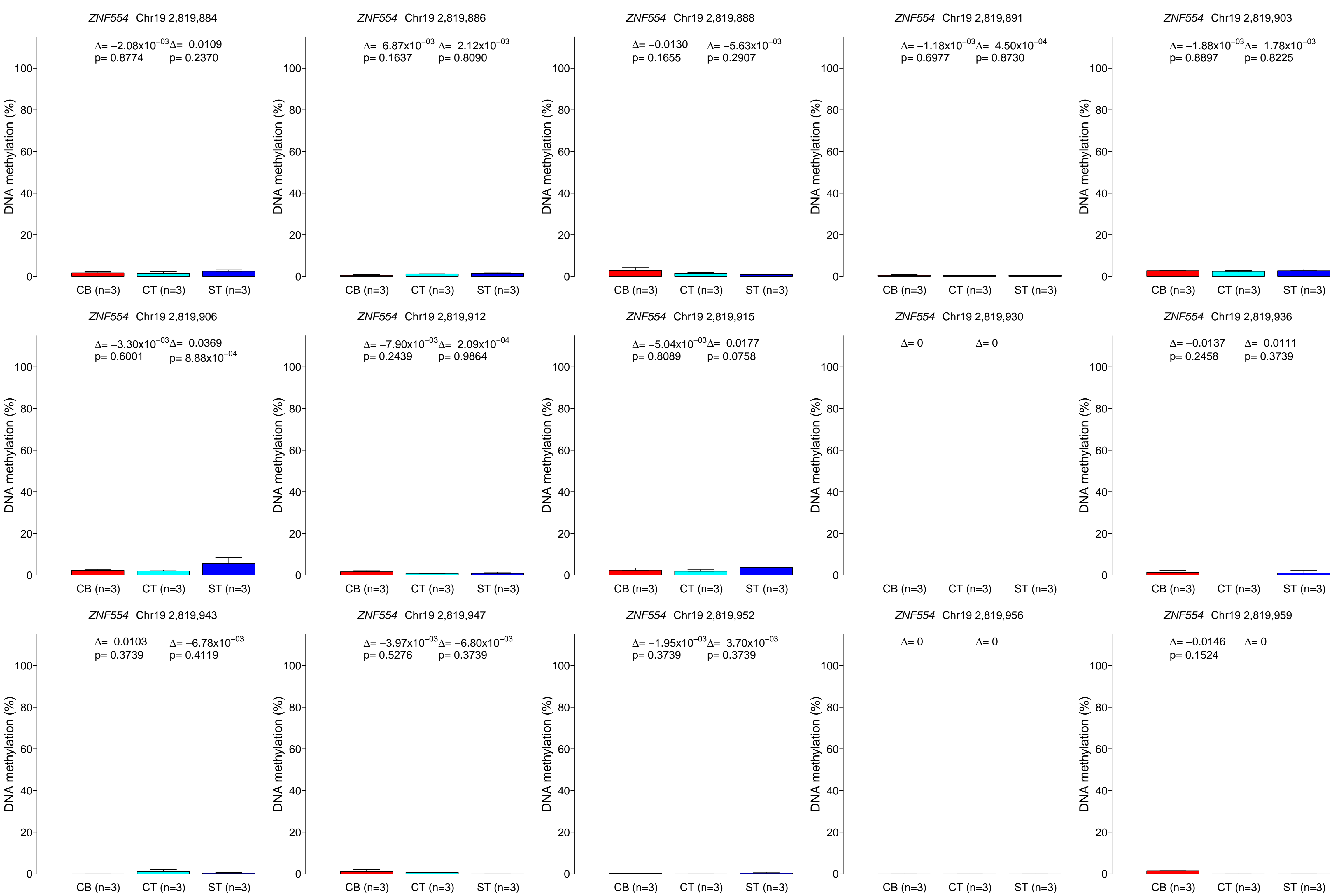

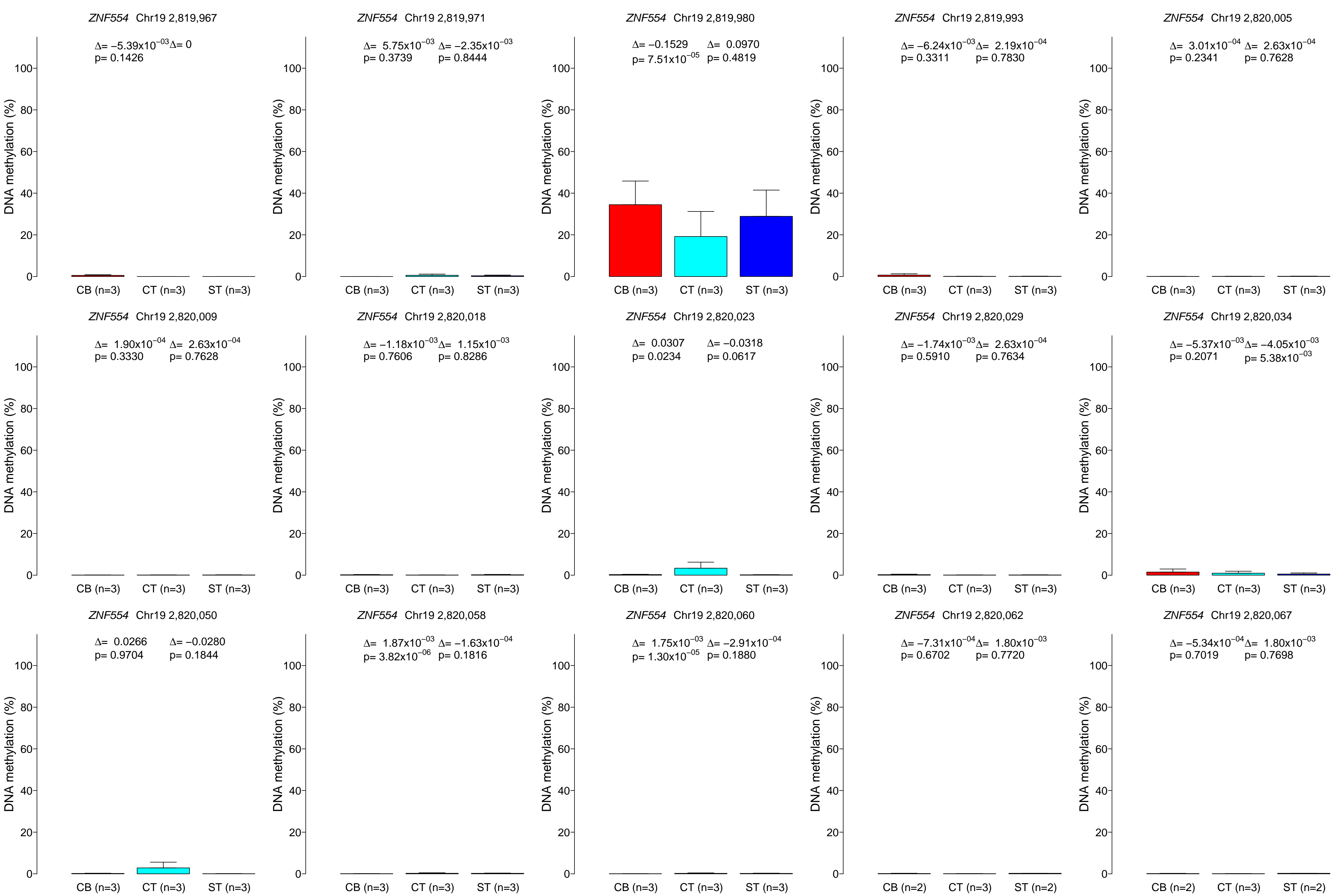

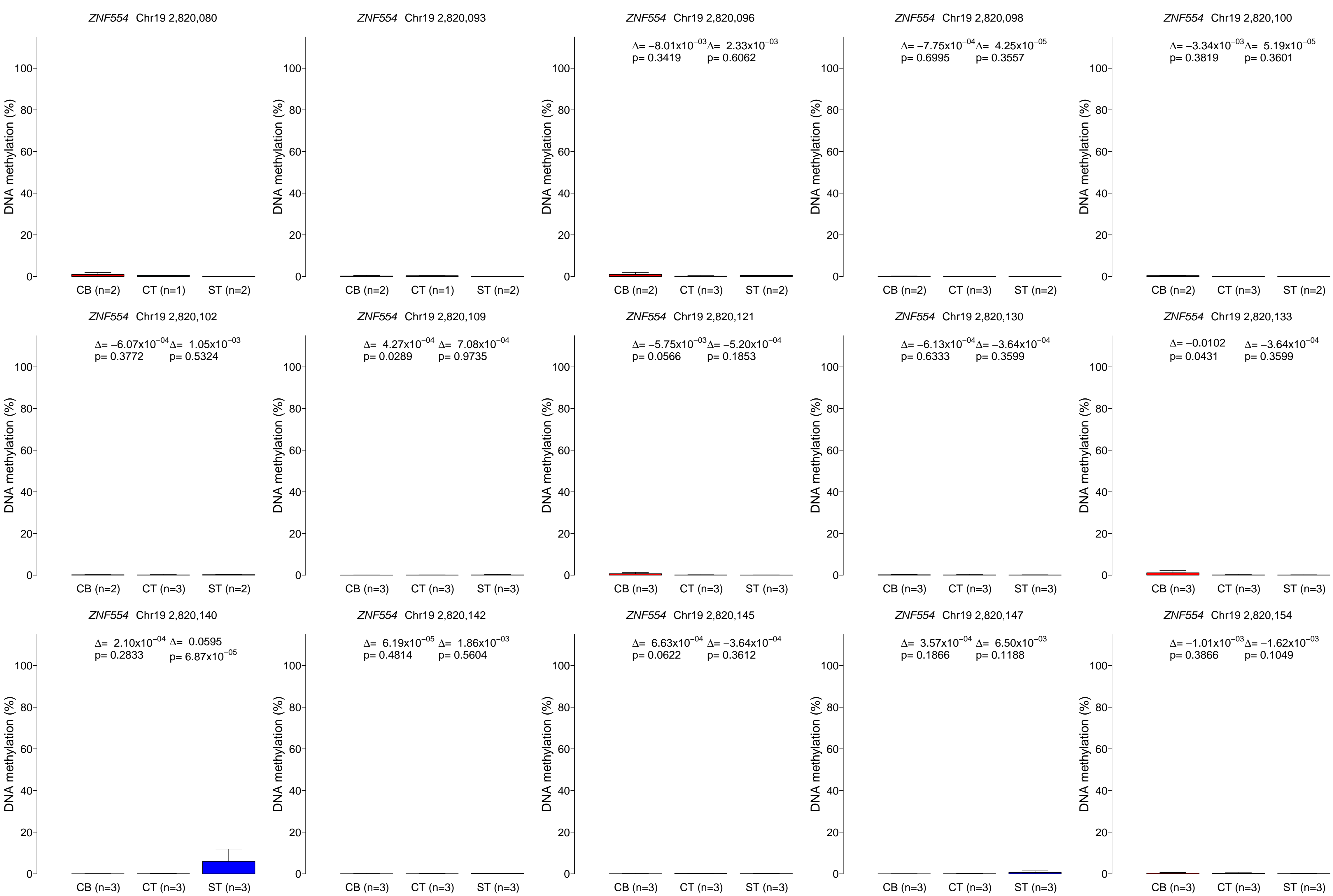

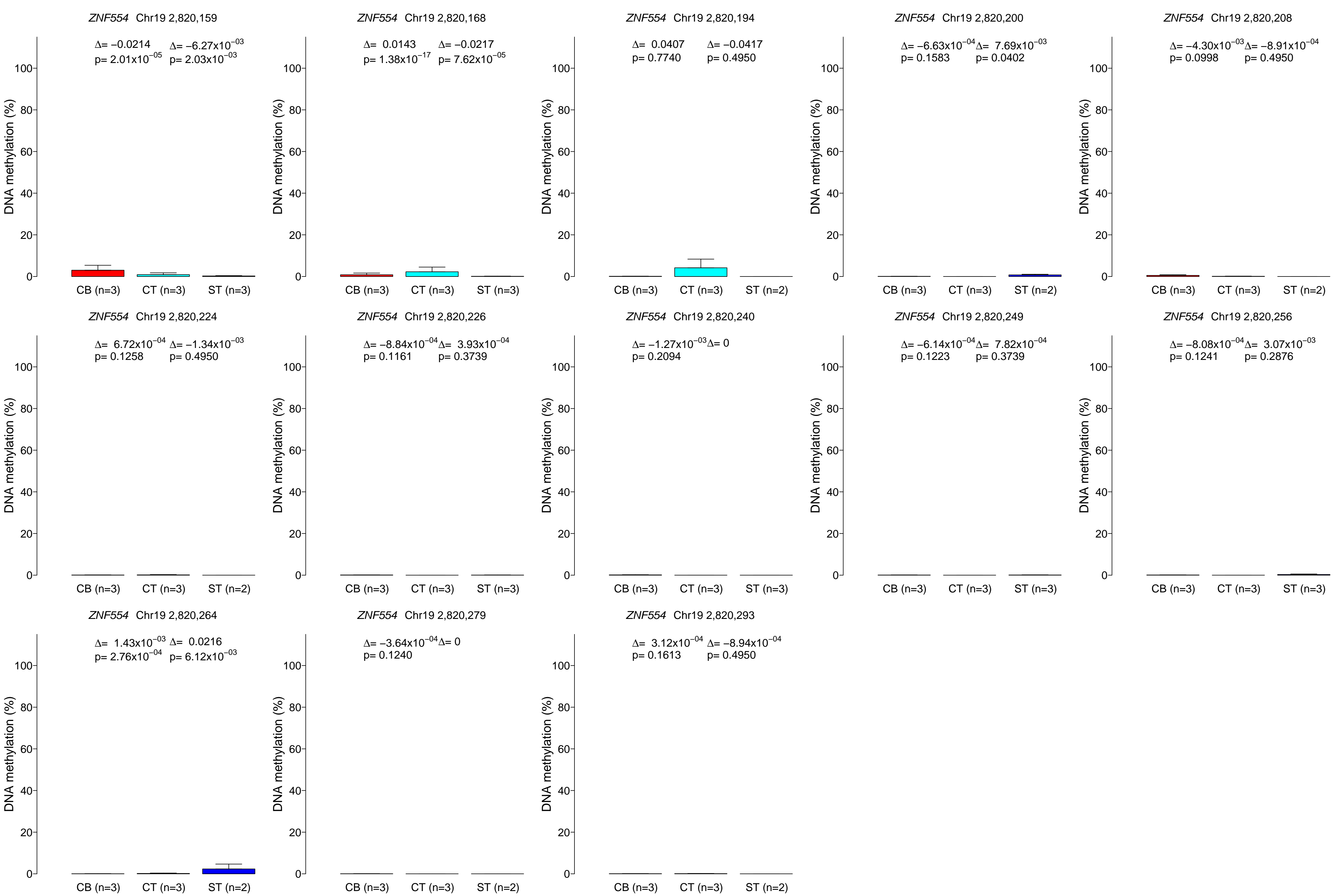

Supplement: Figure S12 — DNA methylation levels at individual CpGs in ZNF554 in the trophoblast and umbilical cord blood cells. DNA methylation levels (0–100%) at individual CpGs in ZNF554 in umbilical cord blood cells (CB), cytotrophoblasts (CT), and differentiated syncytiotrophoblasts (ST) are depicted in the bar plots that represent means and SEs. Umbilical cord blood cells and CT were obtained from the same fetuses. The genomic coordinates of the CpGs, the group differences (CB vs. CT; CT vs. ST) in mean methylation levels and the p-values are shown above the bar plots. The number of samples analyzed with methylation reads above the threshold are shown below the bar plots (only comparisons with a group sample size of minimum two were considered). Differential methylation was claimed to be mild, moderate, or strong when the p-value was <0.05 and the difference in methylation level was ≥0.125, ≥0.25, or ≥0.5, respectively. [file Image_12.pdf]

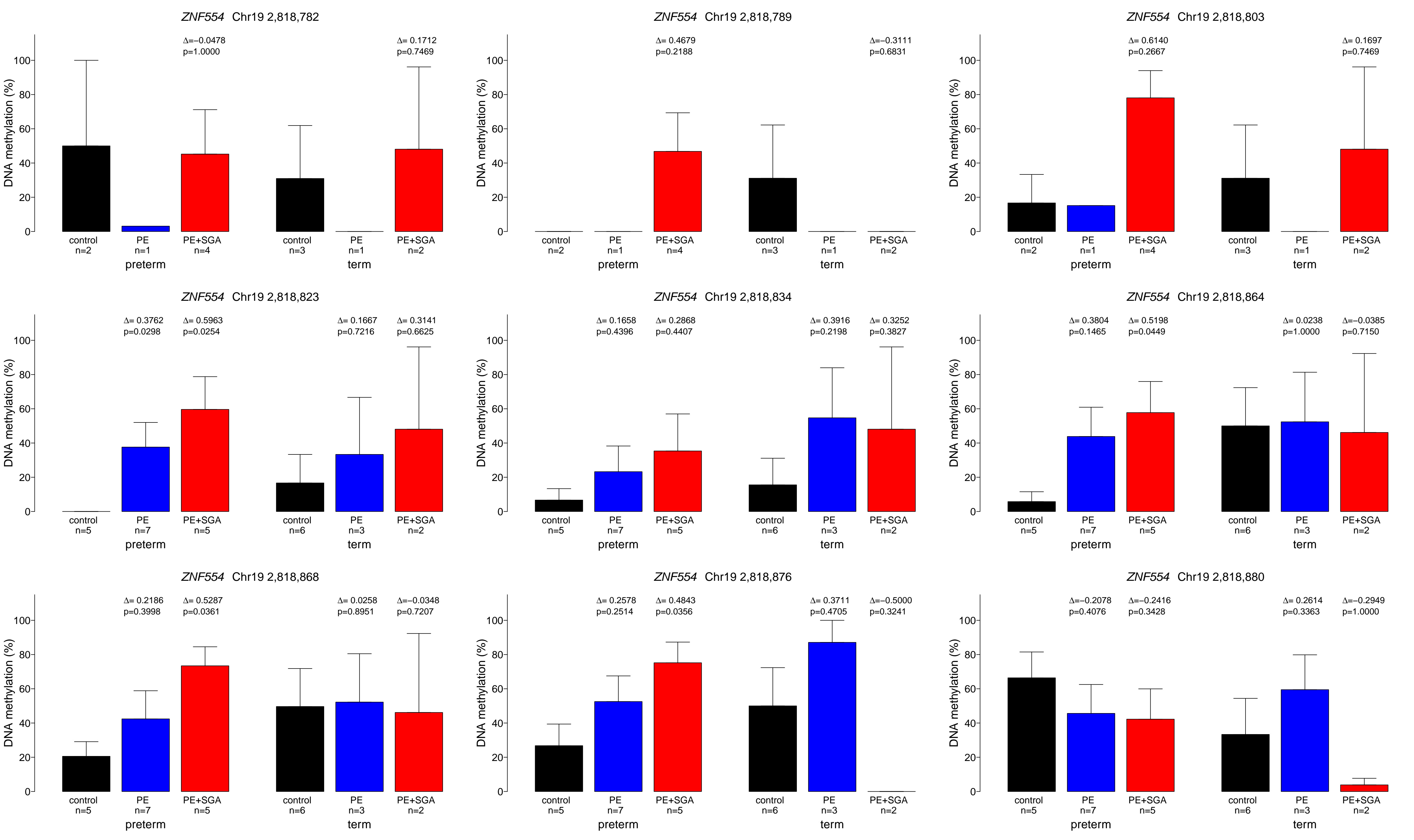

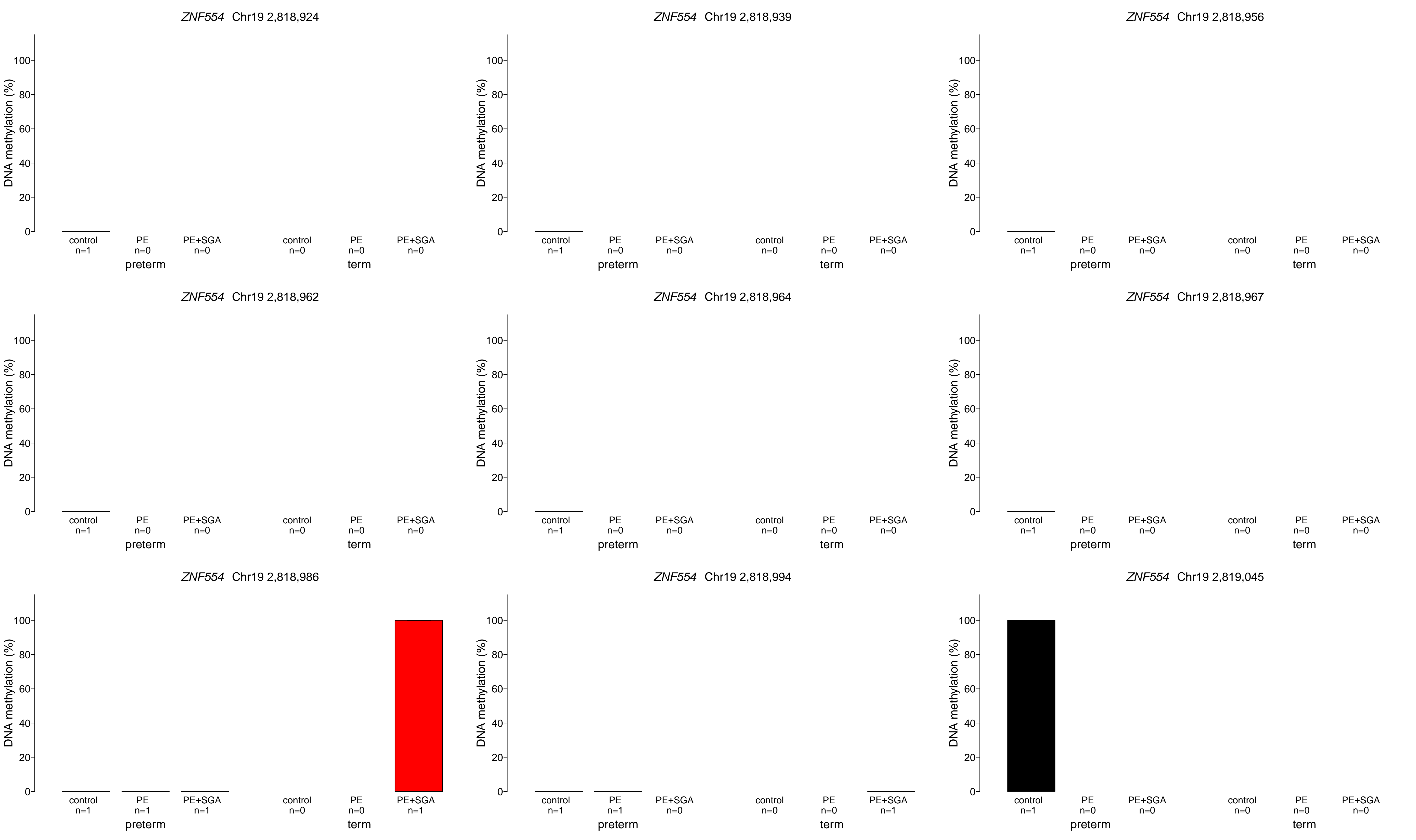

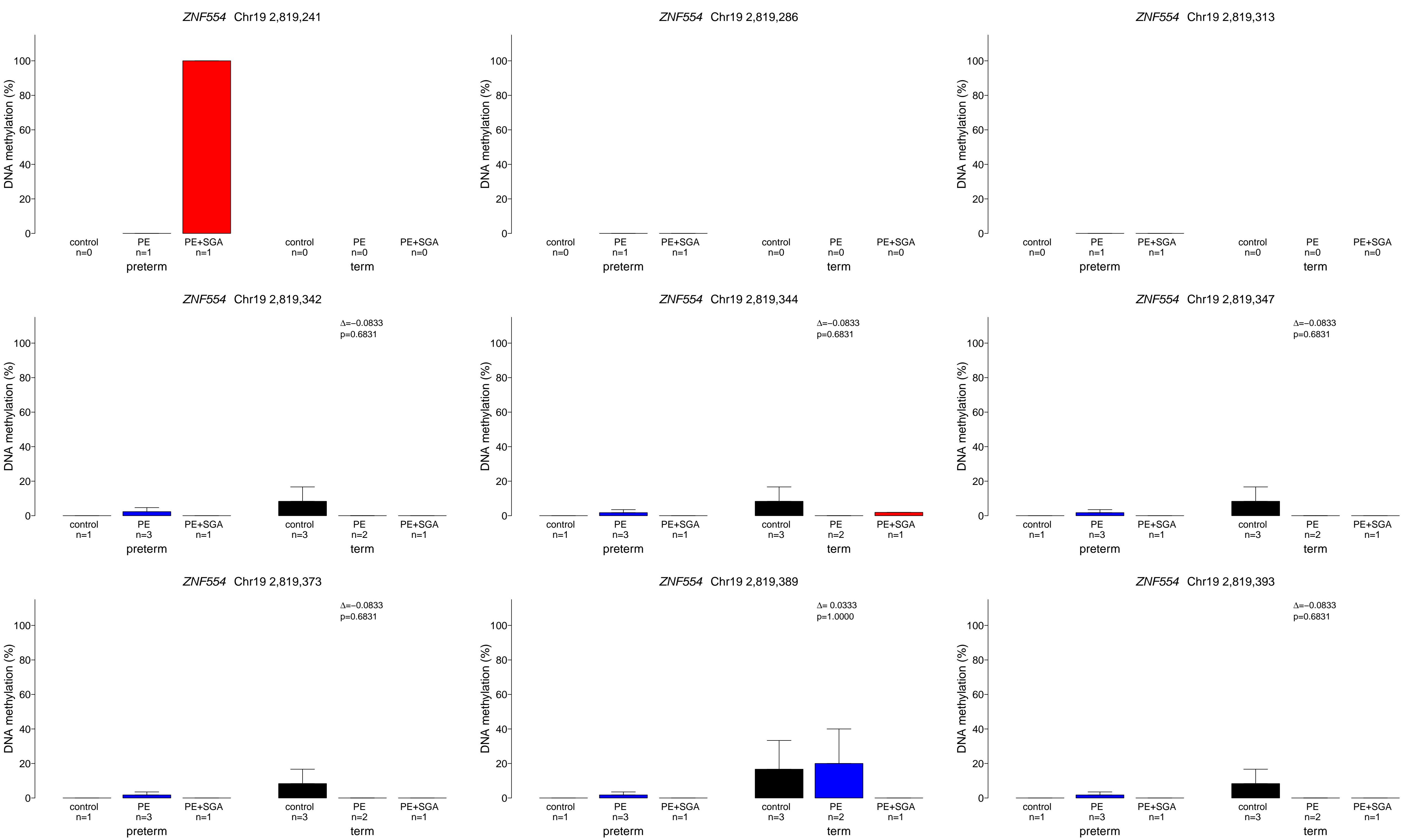

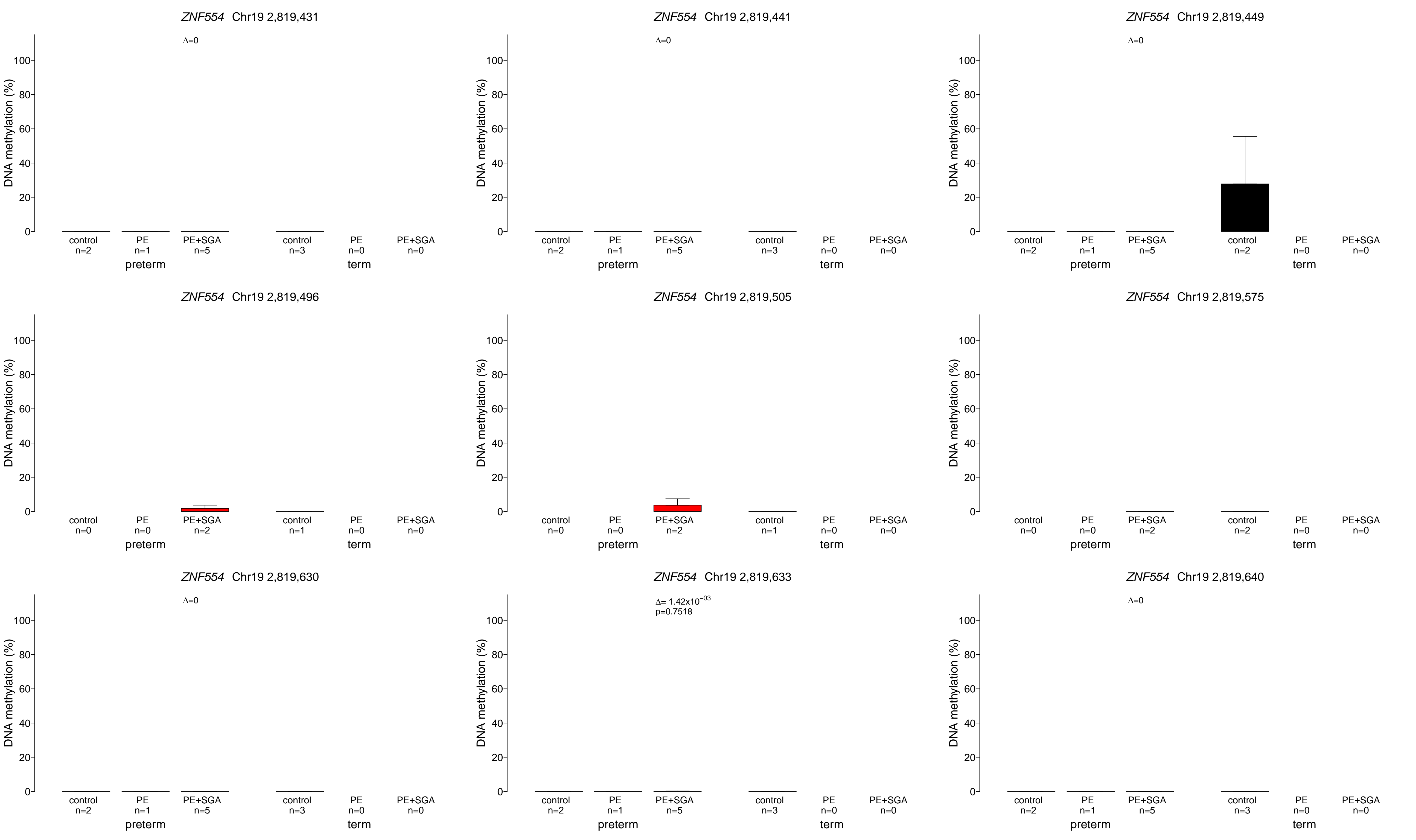

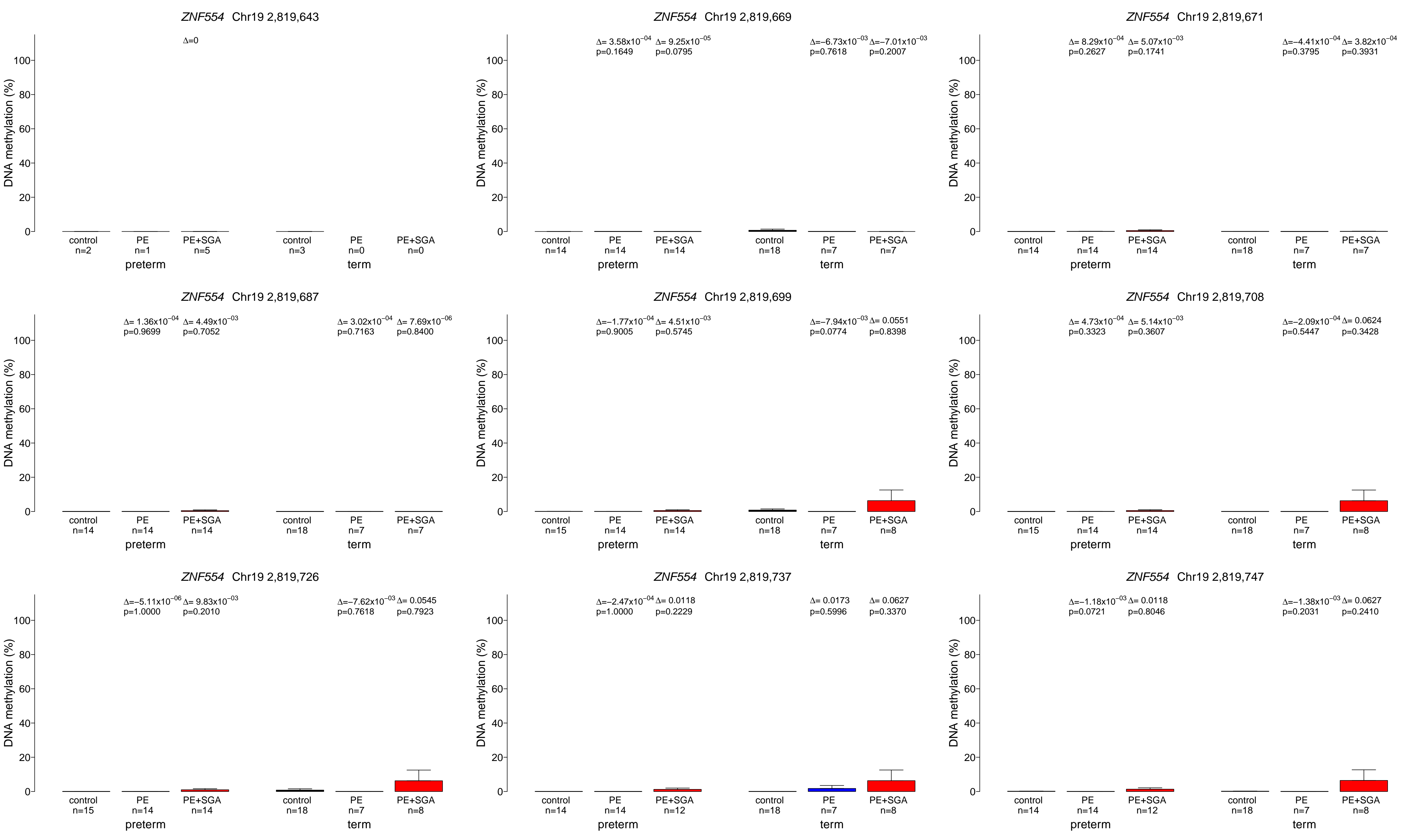

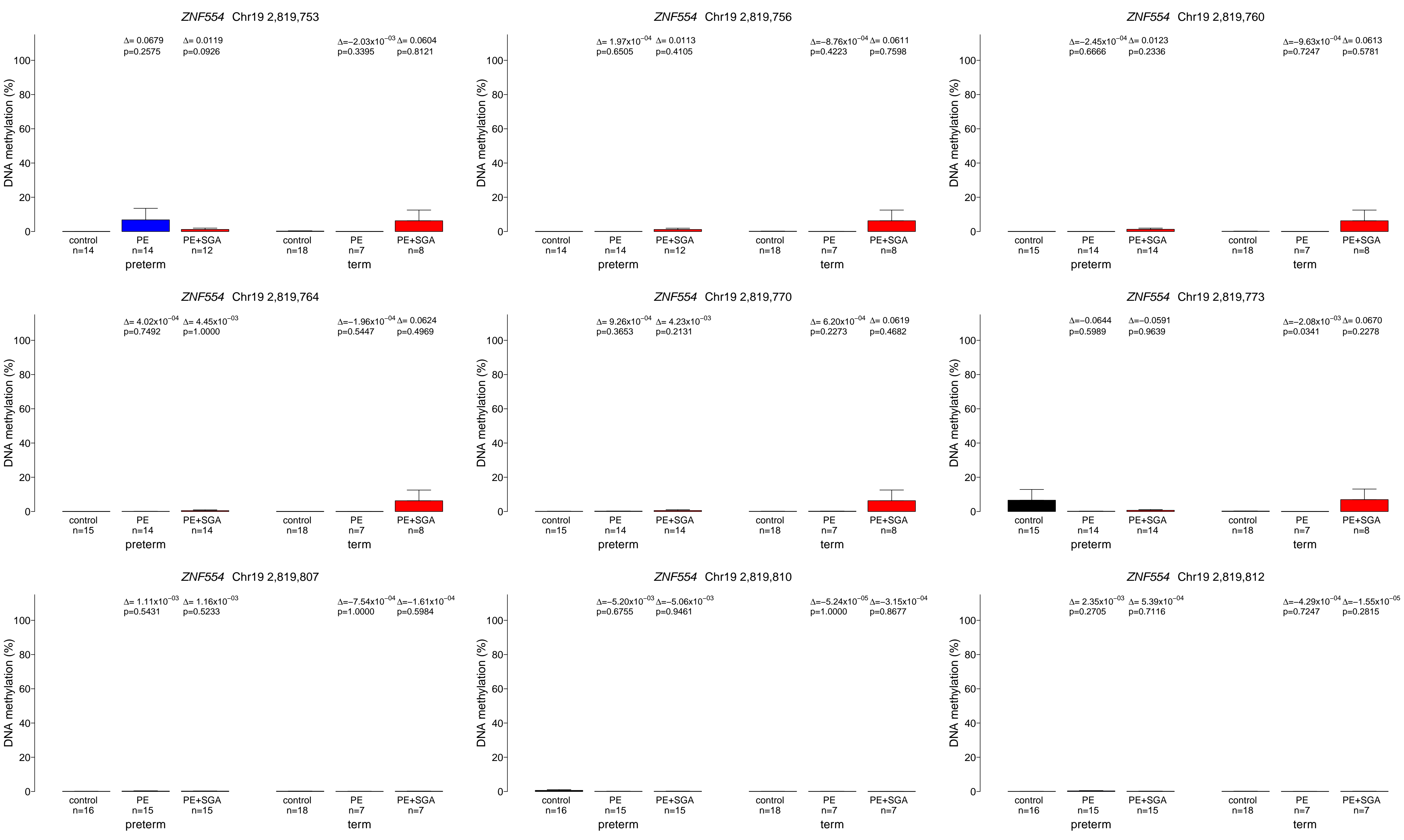

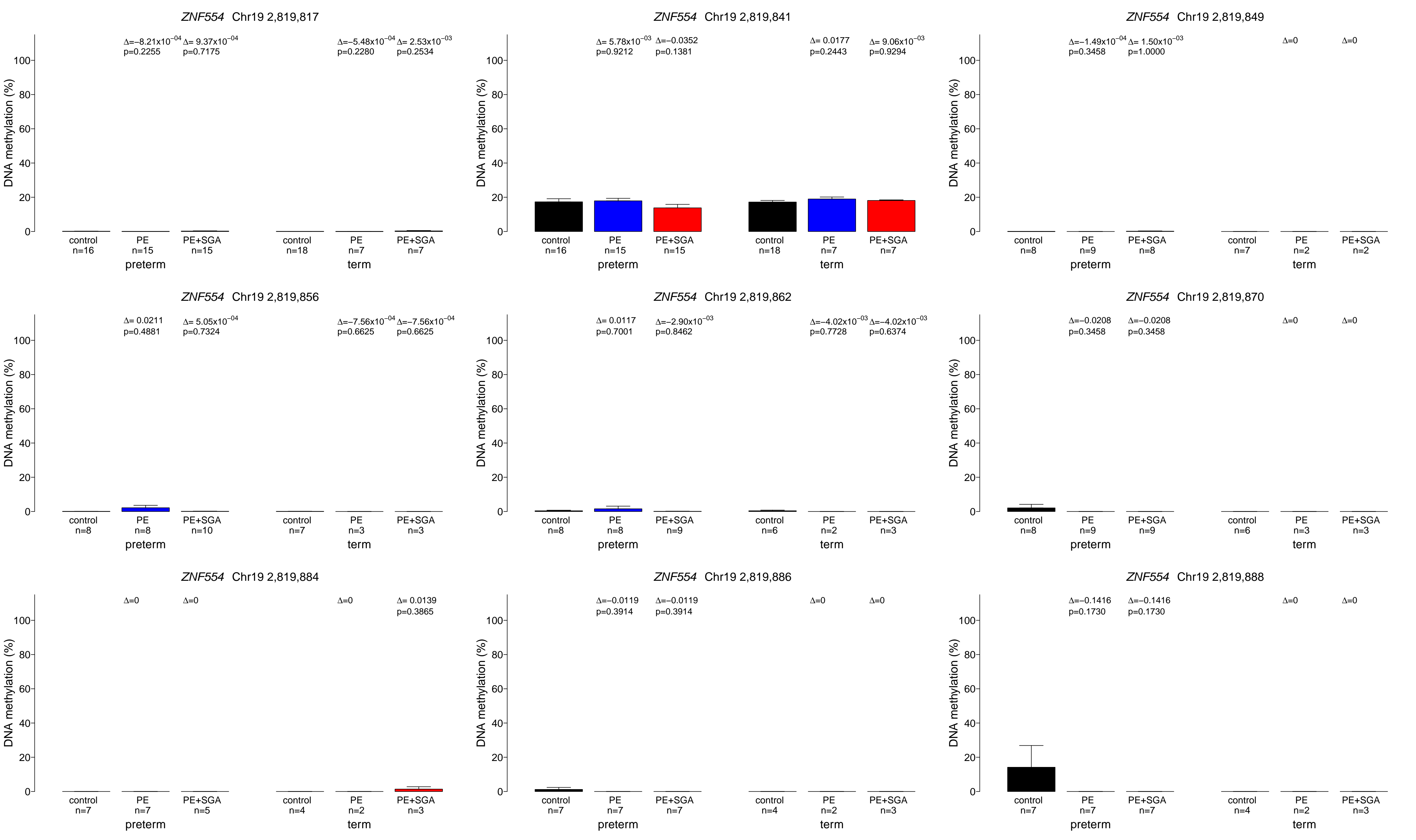

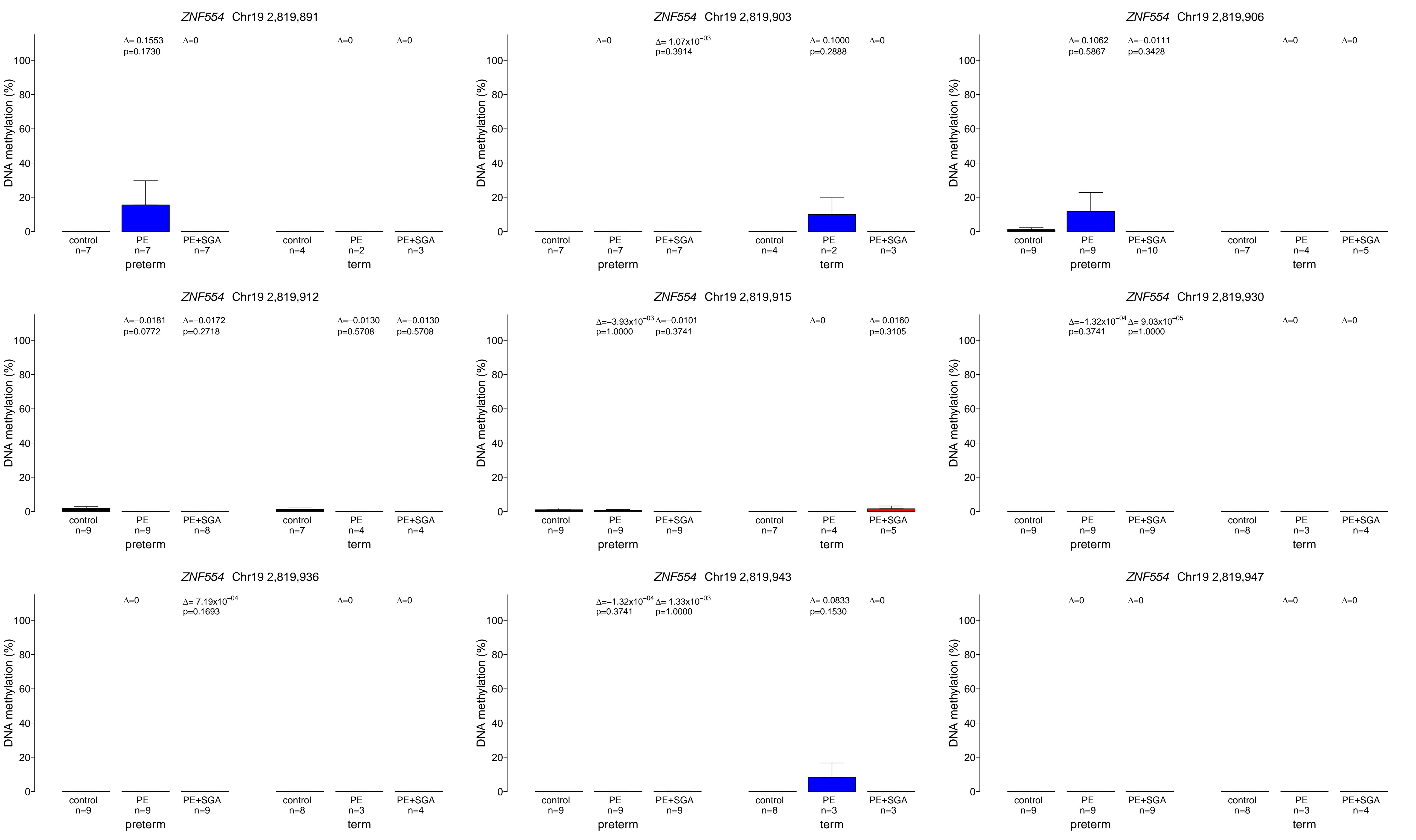

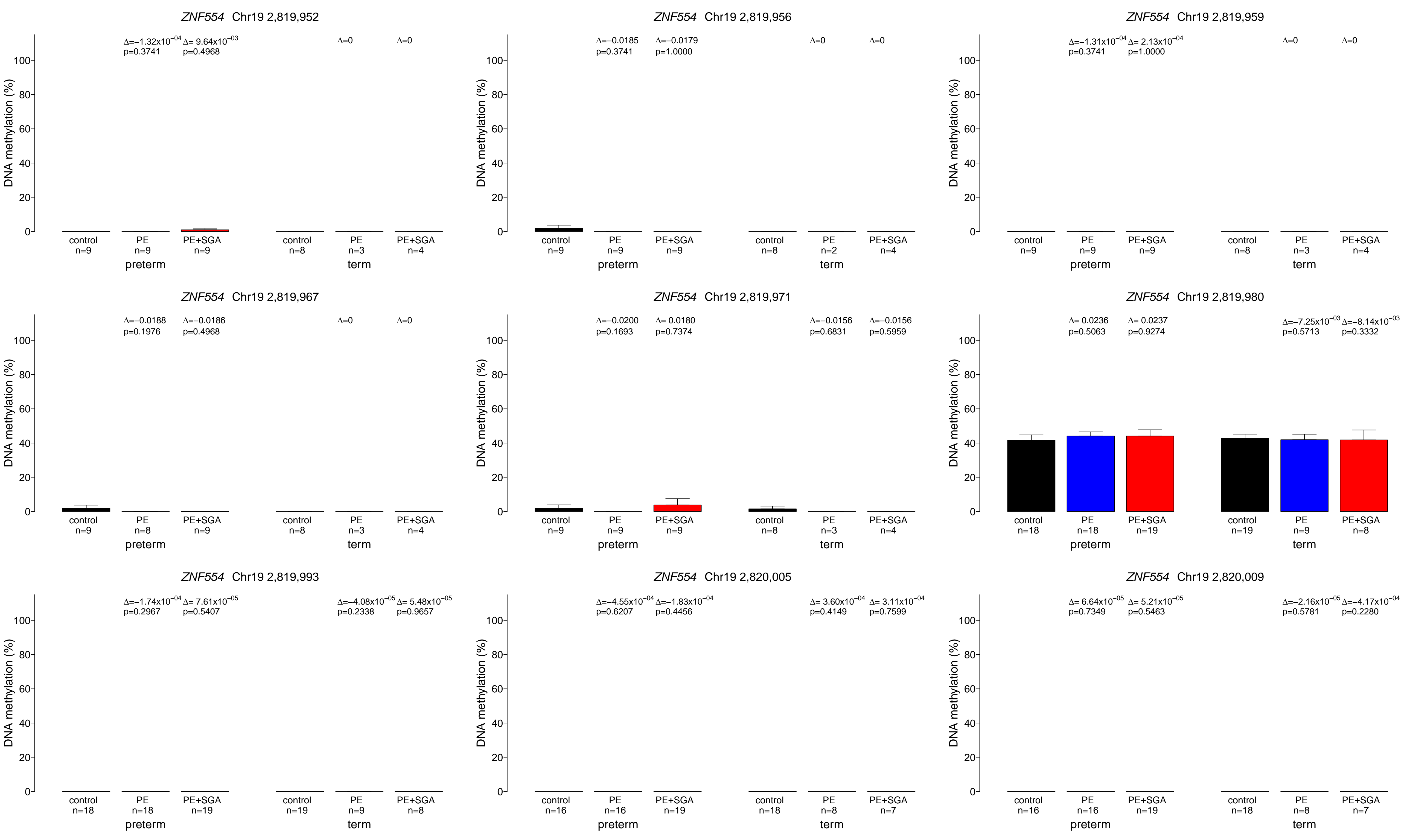

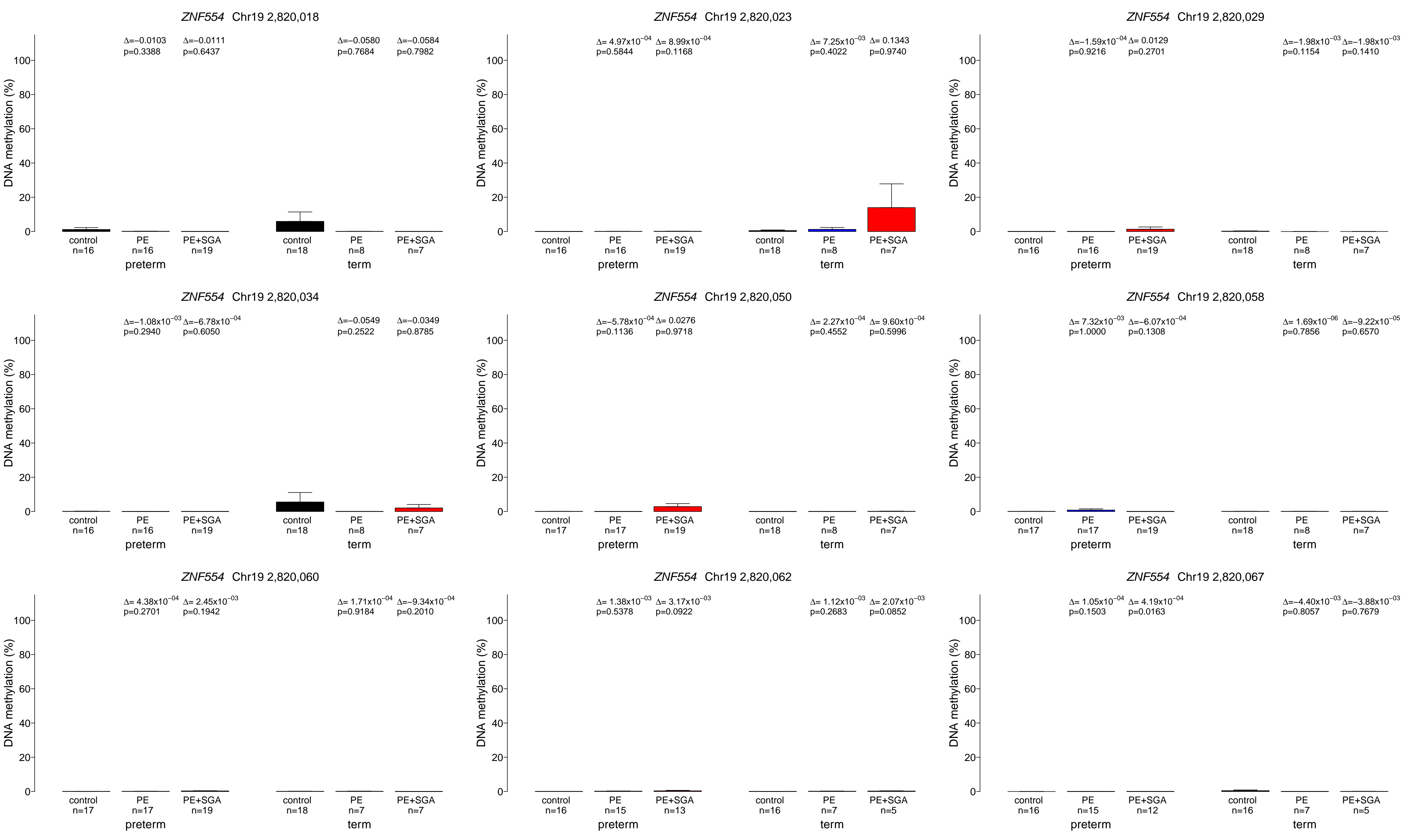

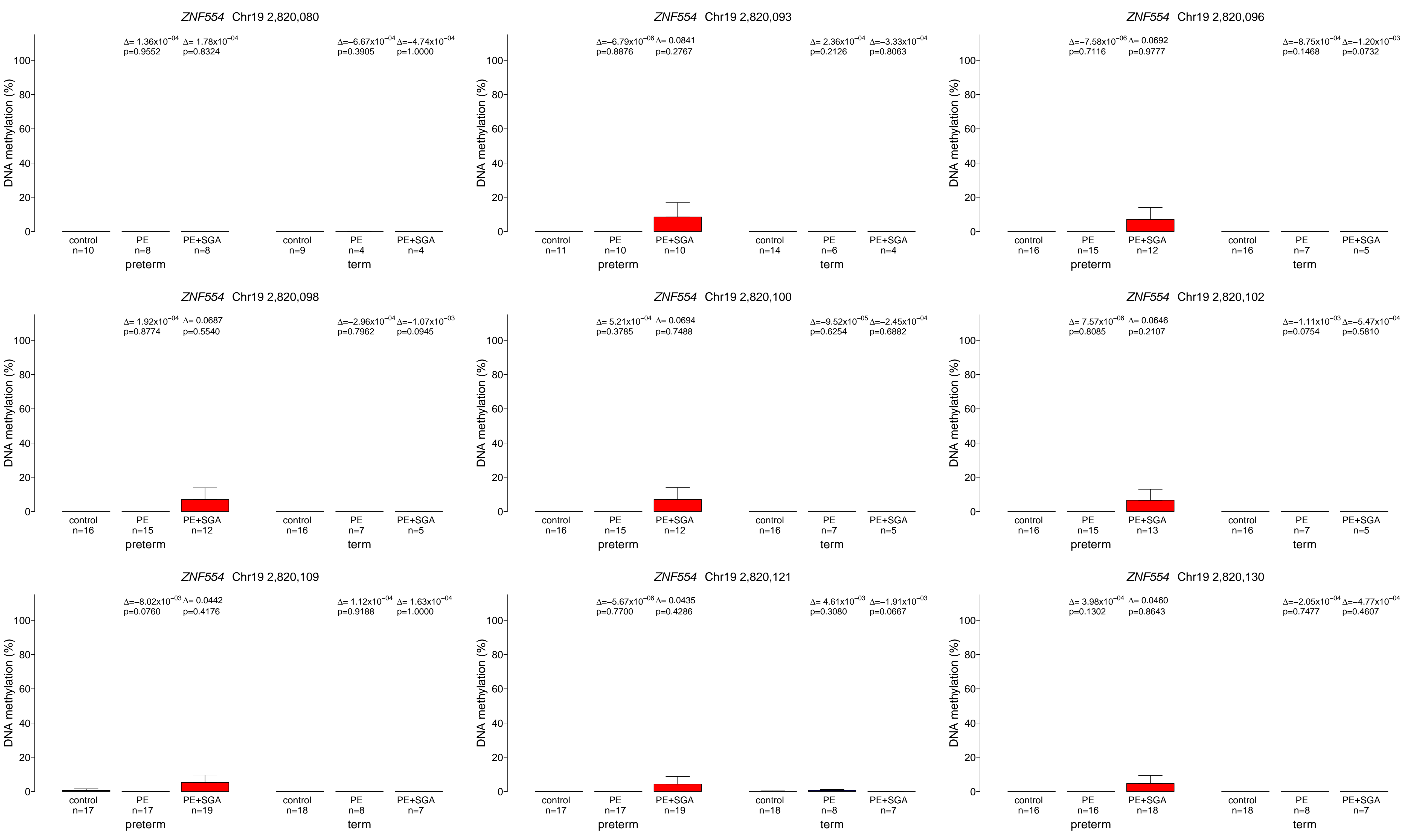

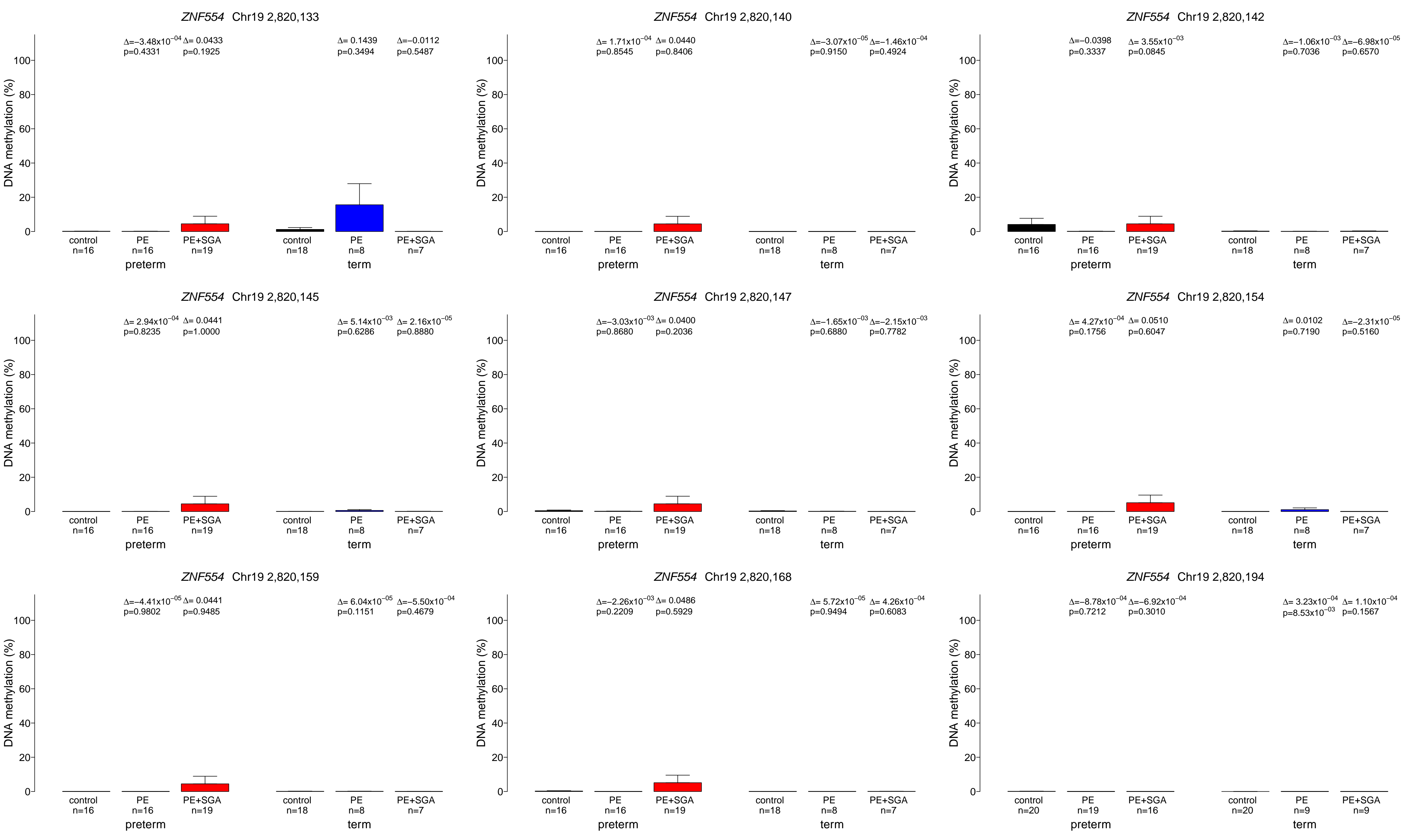

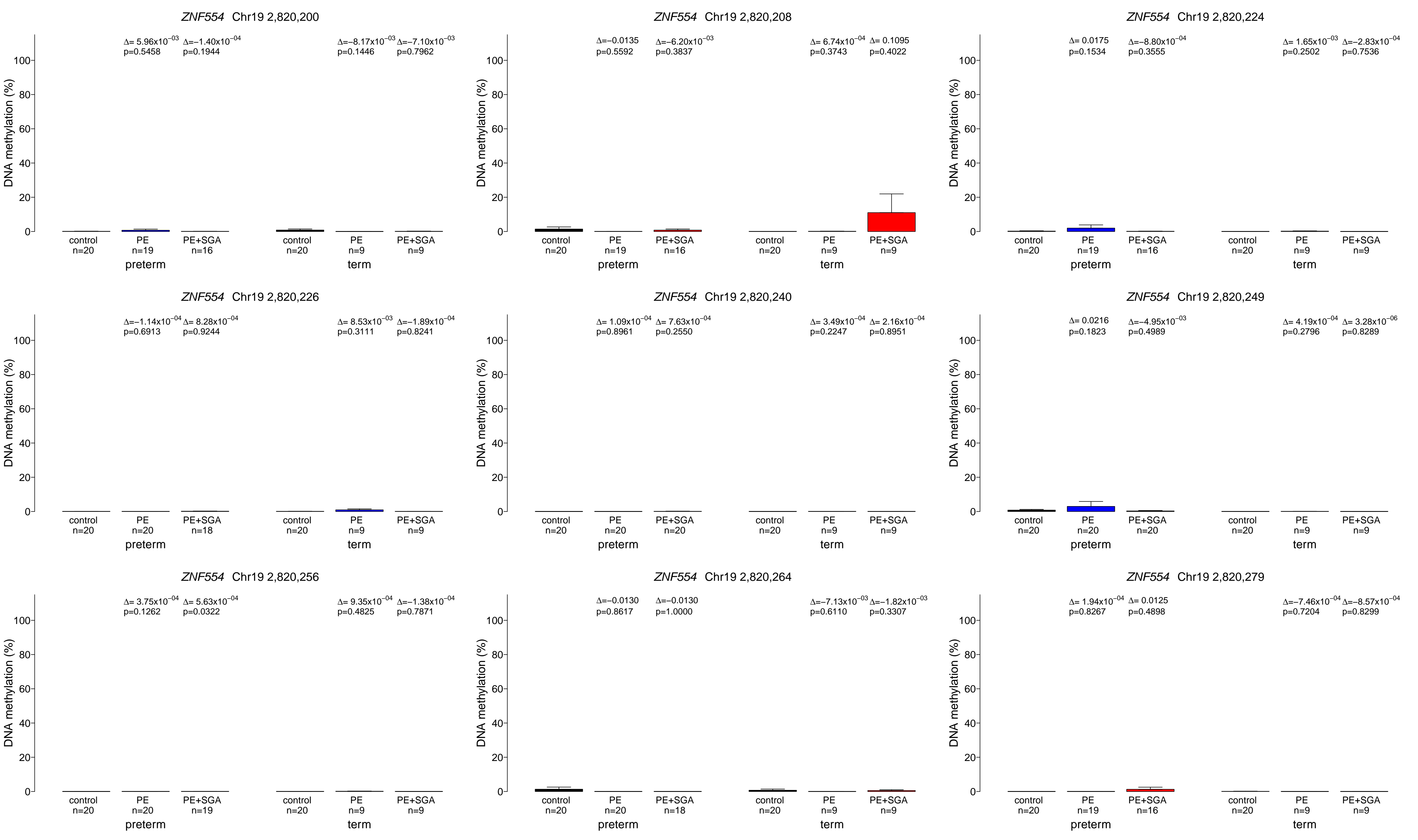

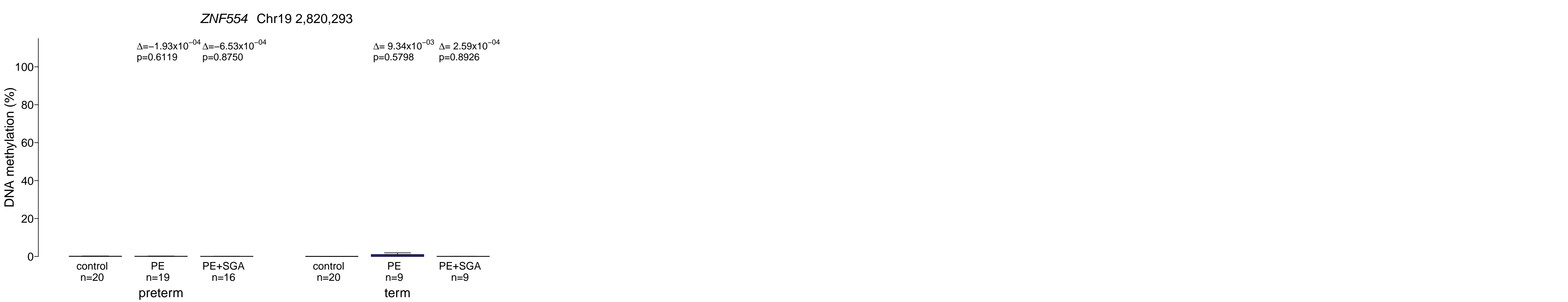

Supplement: Figure S13 — DNA methylation levels at individual CpGs in ZNF554 in the trophoblast in controls and in cases of preeclampsia. DNA methylation levels (0–100%) at individual CpGs in ZNF554 in laser captured trophoblasts are depicted in the bar plots that represent means and SEs. The genomic coordinates of the CpGs, the group differences (compared preterm or term controls) in methylation levels, and the p-values are shown above the bar plots. The number of samples analyzed with methylation reads above the threshold are shown below the bar plots (only comparisons with a group sample size of minimum four were considered). Differential methylation was claimed to be mild, moderate, or strong when the p-value was <0.05 and the difference in methylation level was ≥0.125, ≥0.25, or ≥0.5, respectively. Preterm (left) and term (right) groups of patients were analyzed separately. Abbreviations: PE, preeclampsia; PE + SGA, preeclampsia associated with SGA. [file Image_13.pdf]
