# Supplementary material for: Integrated Systems Biology Approach Identifies Novel Maternal and Placental Pathways of Preeclampsia
Source: Front Immunol. 2018 Aug 8;9:1661. doi: 10.3389/fimmu.2018.01661 (PMC6092567; doi:10.3389/fimmu.2018.01661)
Supplement: Figure S3 — Placental gene expression changes in various phenotypes of preeclampsia detected by qRT-PCR. Data represent gene expressions relative to RPLP0 measured across 100 placentas. In each bar plot (mean ± SE), the left and right panels show significant differences (“*”) in preterm and term preeclampsia associated with or without SGA samples compared to gestational age-matched controls, respectively. Changes in preterm preeclampsia samples significantly different to changes in term preeclampsia samples are indicated by “+”. [file Image_3.pdf]

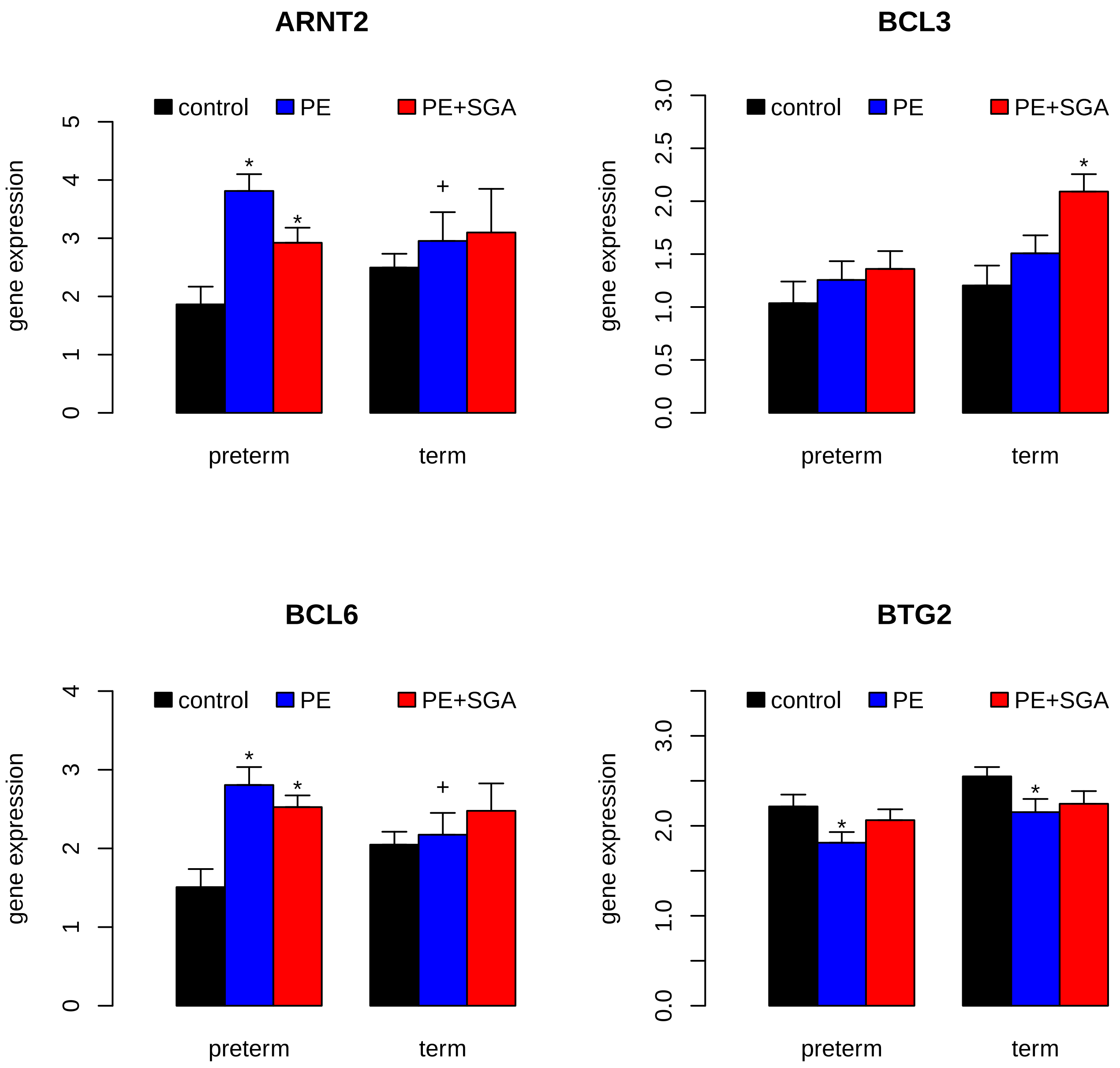

ARNT2
control
PE
PE+SGA
gene expression
0
1
2
3
4
5
*
*
+
preterm
term
BCL3
control
PE
PE+SGA
gene expression
0.0
0.5
1.0
1.5
2.0
2.5
3.0
*
preterm
term
BCL6
control
PE
PE+SGA
gene expression
0
1
2
3
4
*
*
+
preterm
term
BTG2
control
PE
PE+SGA
gene expression
0.0
1.0
2.0
3.0
*
*
preterm
term

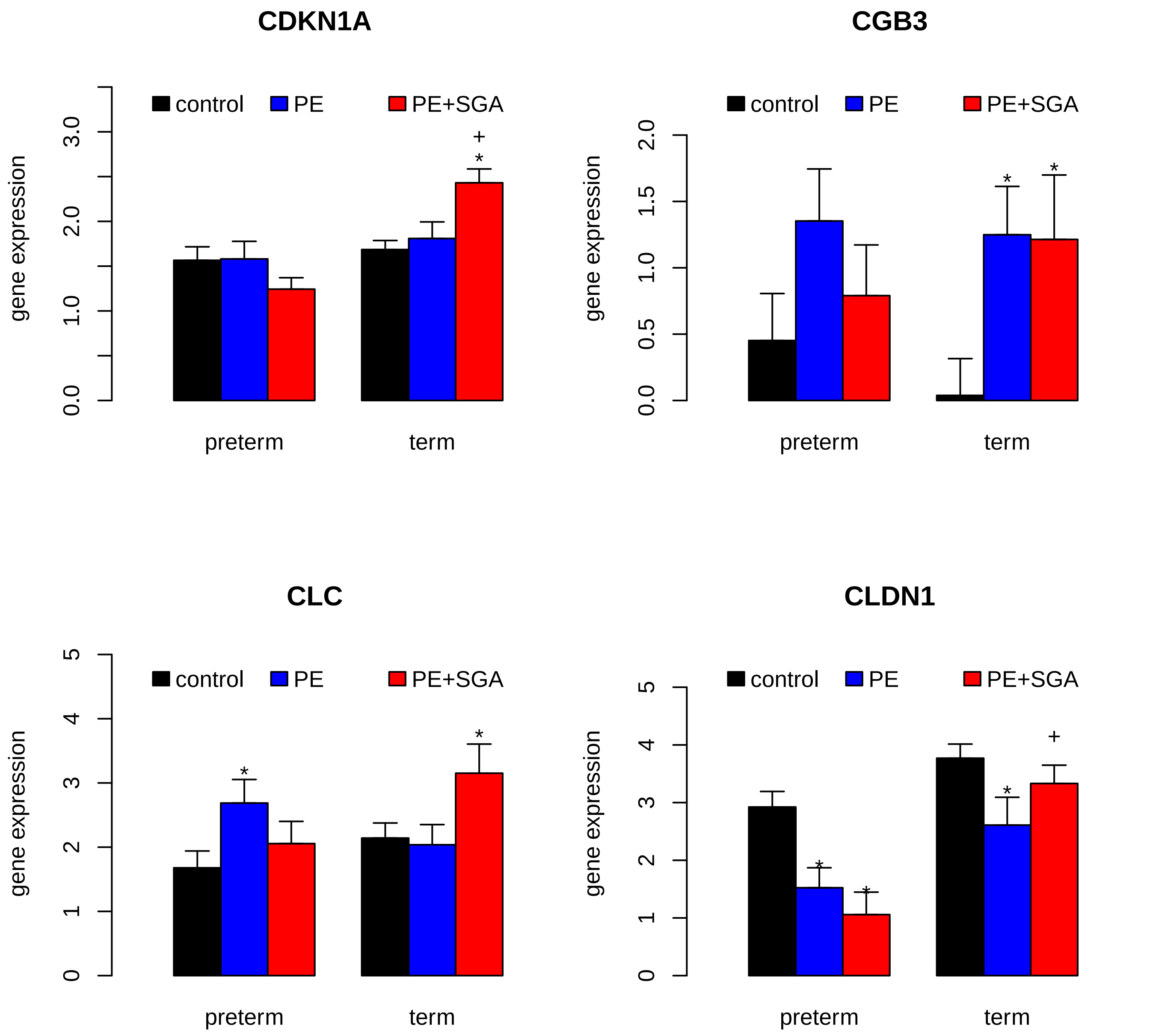

CDKN1A
CGB3
CLC
CLDN1
control
PE
PE+SGA
gene expression
preterm
term
0.0
1.0
2.0
3.0
0.5
1.5
0
1
2
3
4
5
*
+

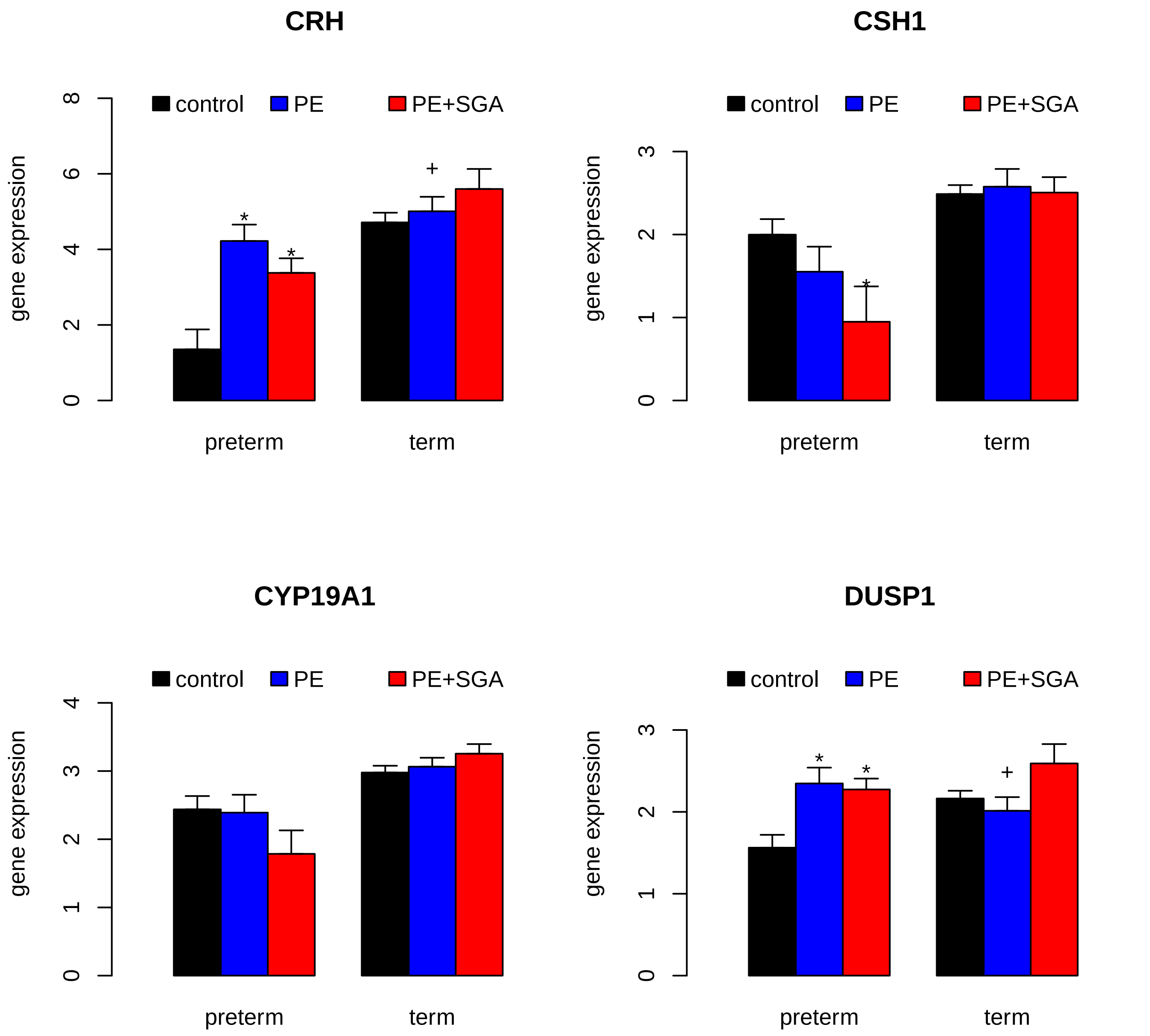

CRH
CSH1
CYP19A1
DUSP1
control
PE
PE+SGA
gene expression
preterm
term
0
2
4
6
8
1
3
*
+

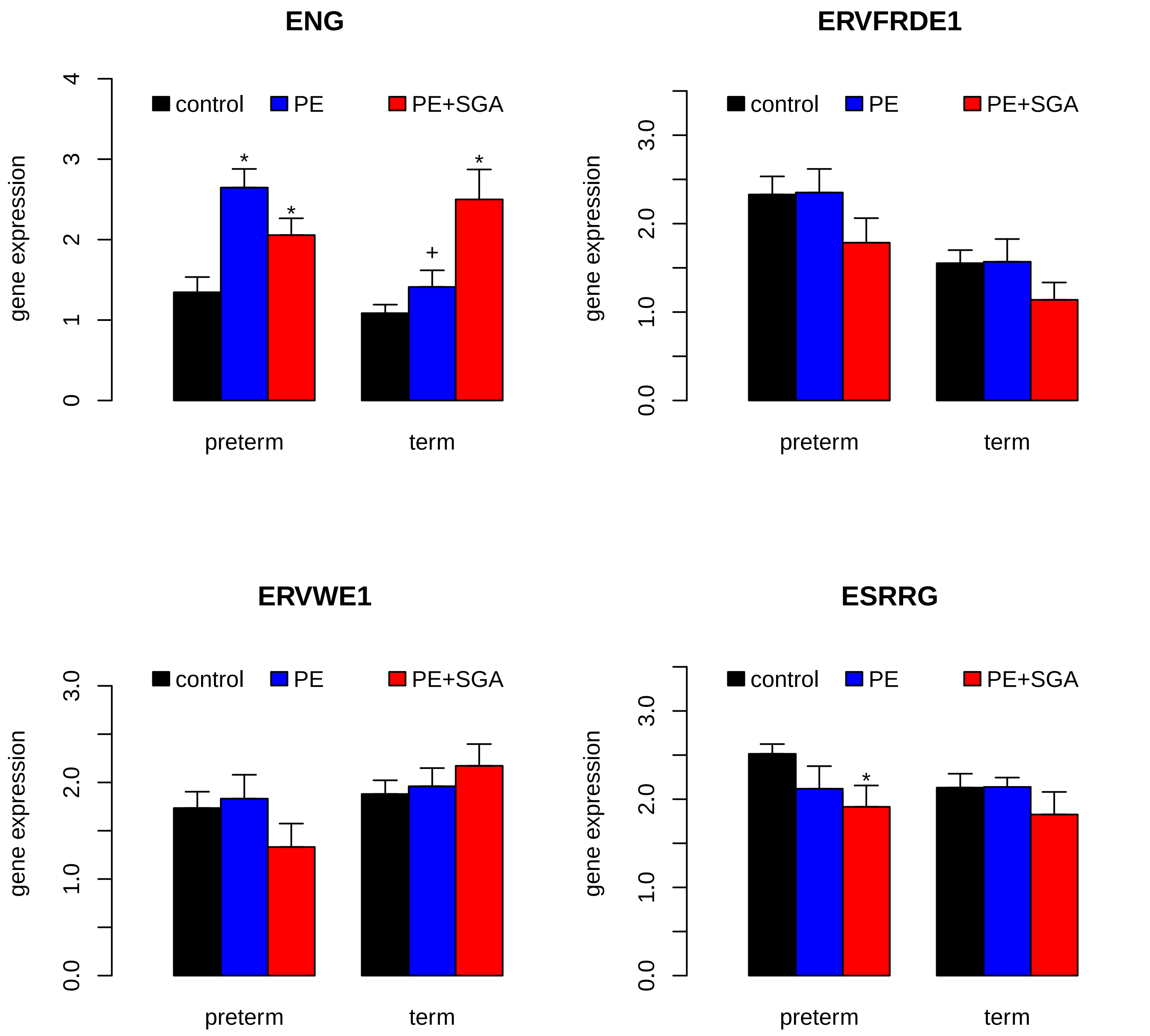

ENG
ERVFRDE1
ERVWE1
ESRRG
control
PE
PE+SGA
gene expression
preterm
term
*
+
0
1
2
3
4
0.0
1.0
2.0
3.0

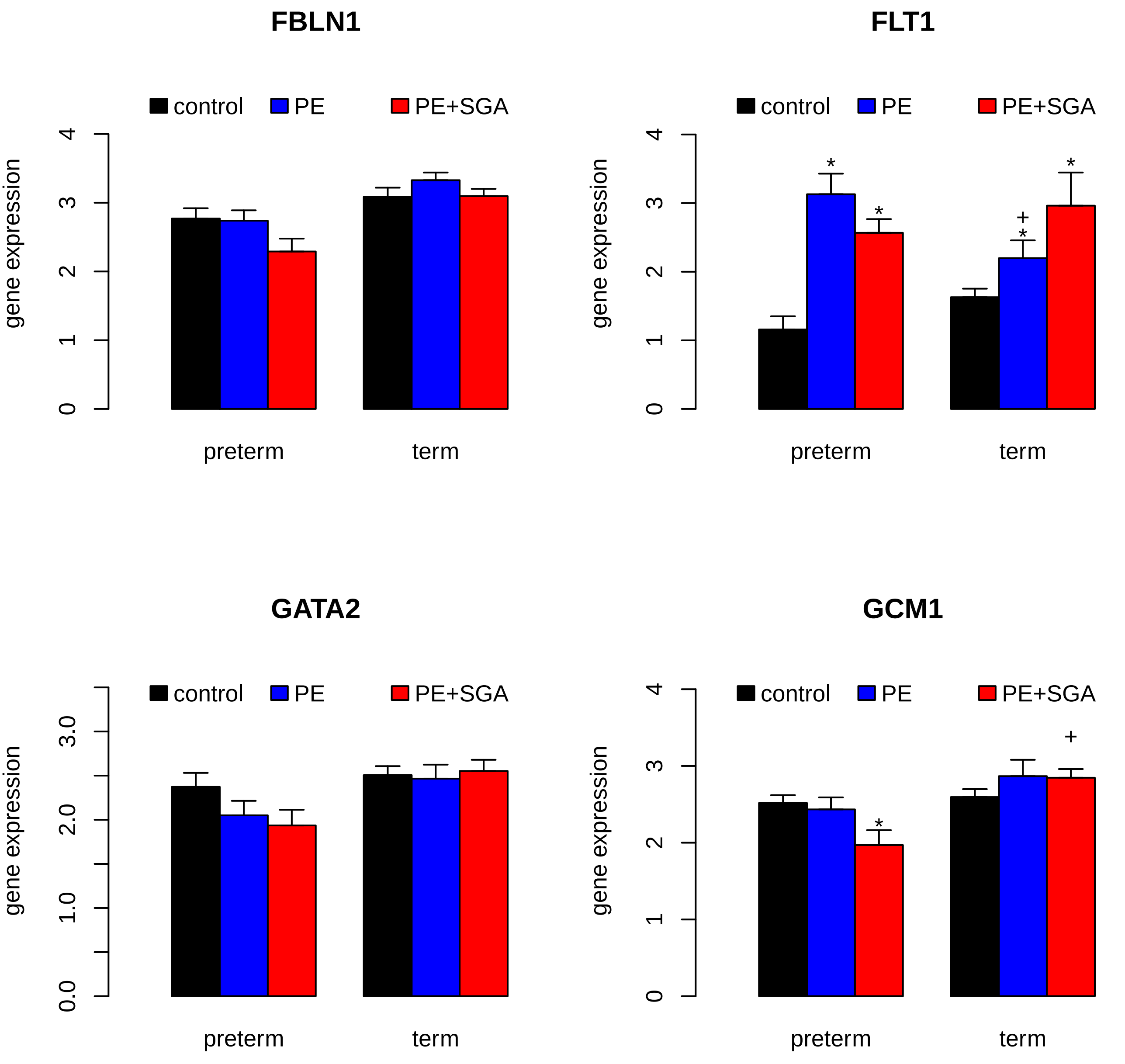
FBLN1
FLT1
GATA2
GCM1
control
PE
PE+SGA
gene expression
preterm
term
0
1
2
3
4
0.0
1.0
2.0
3.0
*
+

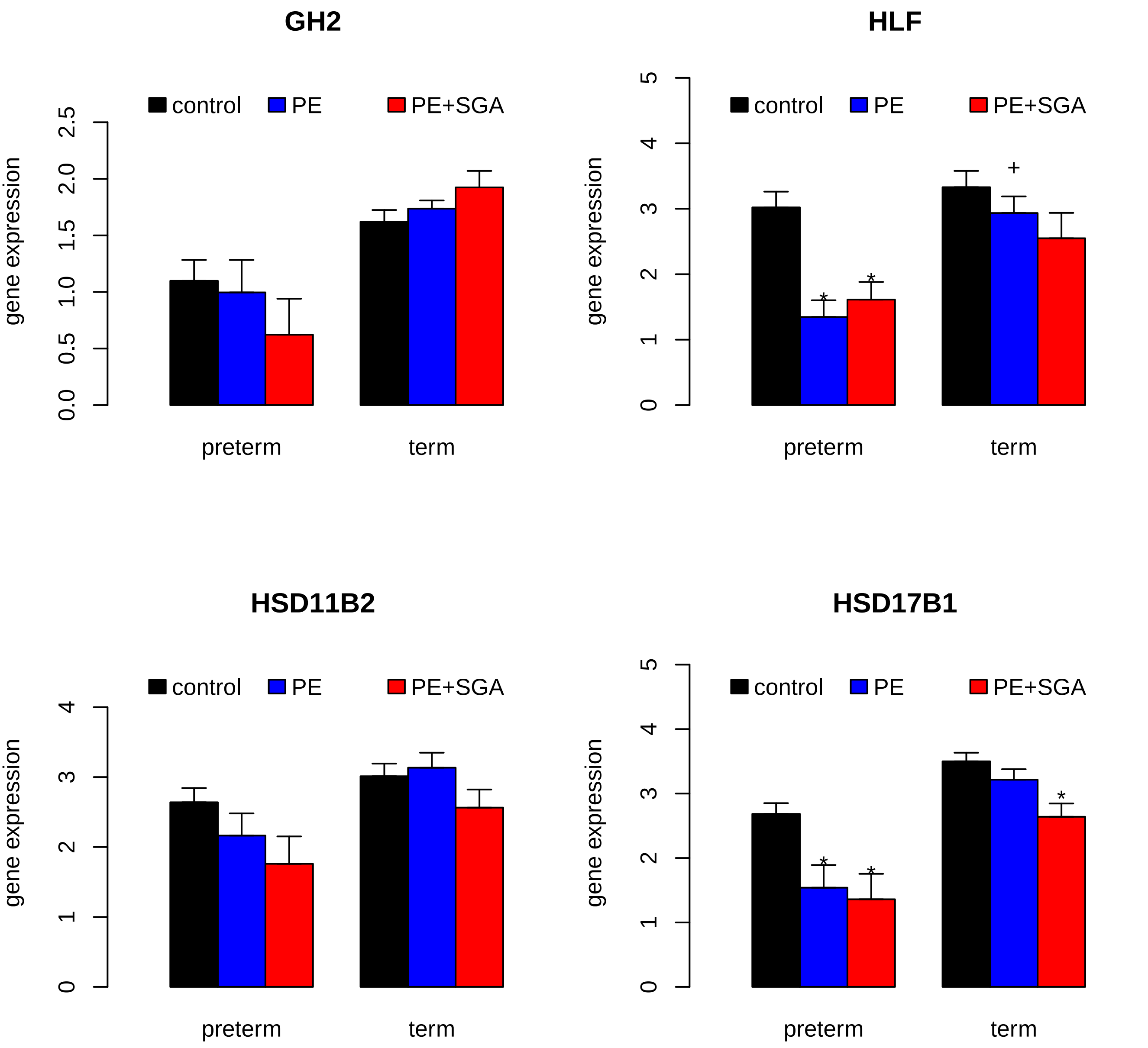

GH2
HLF
HSD11B2
HSD17B1
control
PE
PE+SGA
gene expression
preterm
term
0.0
0.5
1.0
1.5
2.0
2.5
0
1
2
3
4
5
*
+

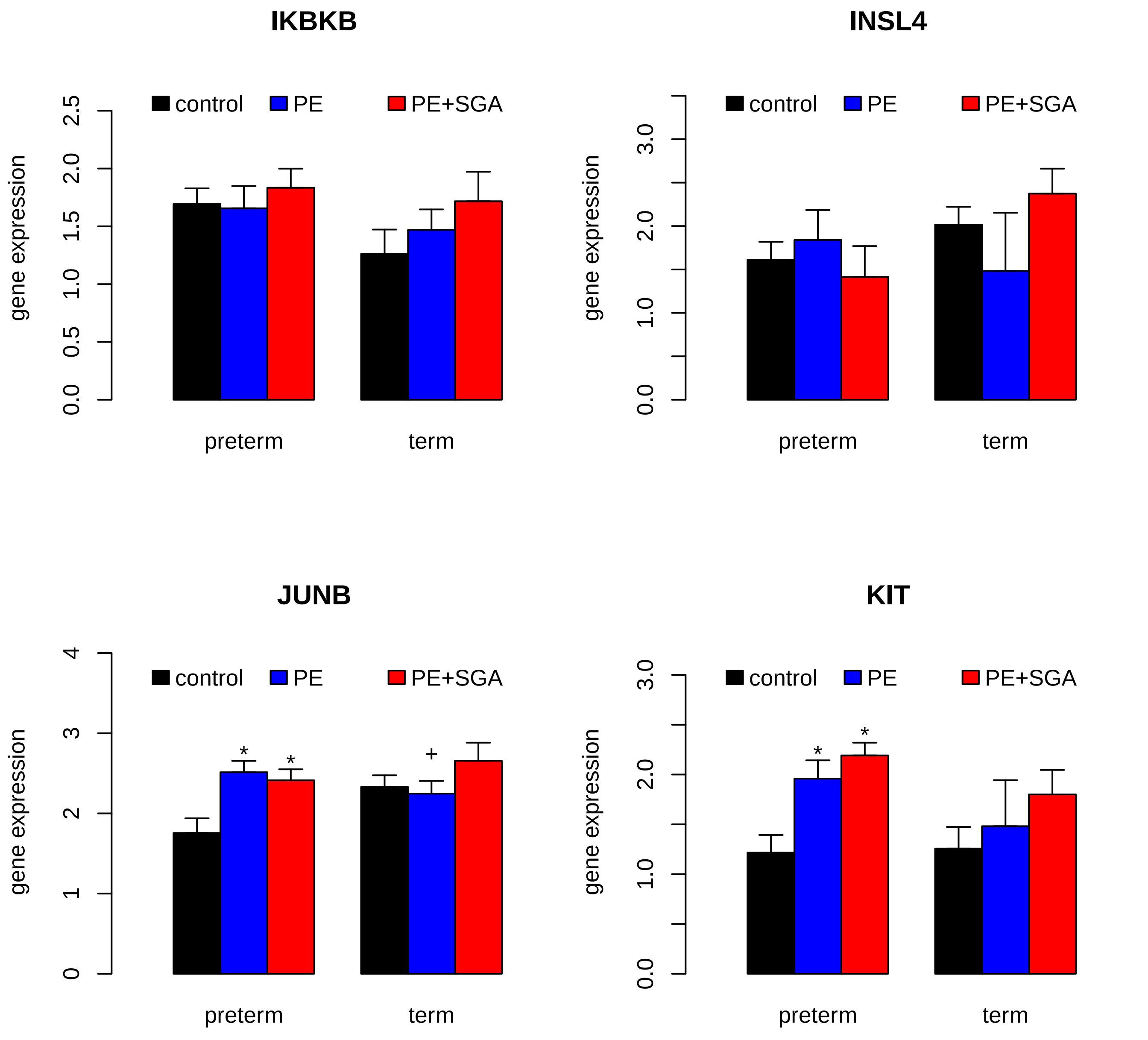
IKBKB
control
PE
PE+SGA
gene expression
0.0
0.5
1.0
1.5
2.0
2.5
preterm
term
INSL4
control
PE
PE+SGA
gene expression
0.0
1.0
2.0
3.0
preterm
term
JUNB
control
PE
PE+SGA
gene expression
0
1
2
3
4
*
*
+
preterm
term
KIT
control
PE
PE+SGA
gene expression
0.0
1.0
2.0
3.0
*
*
preterm
term

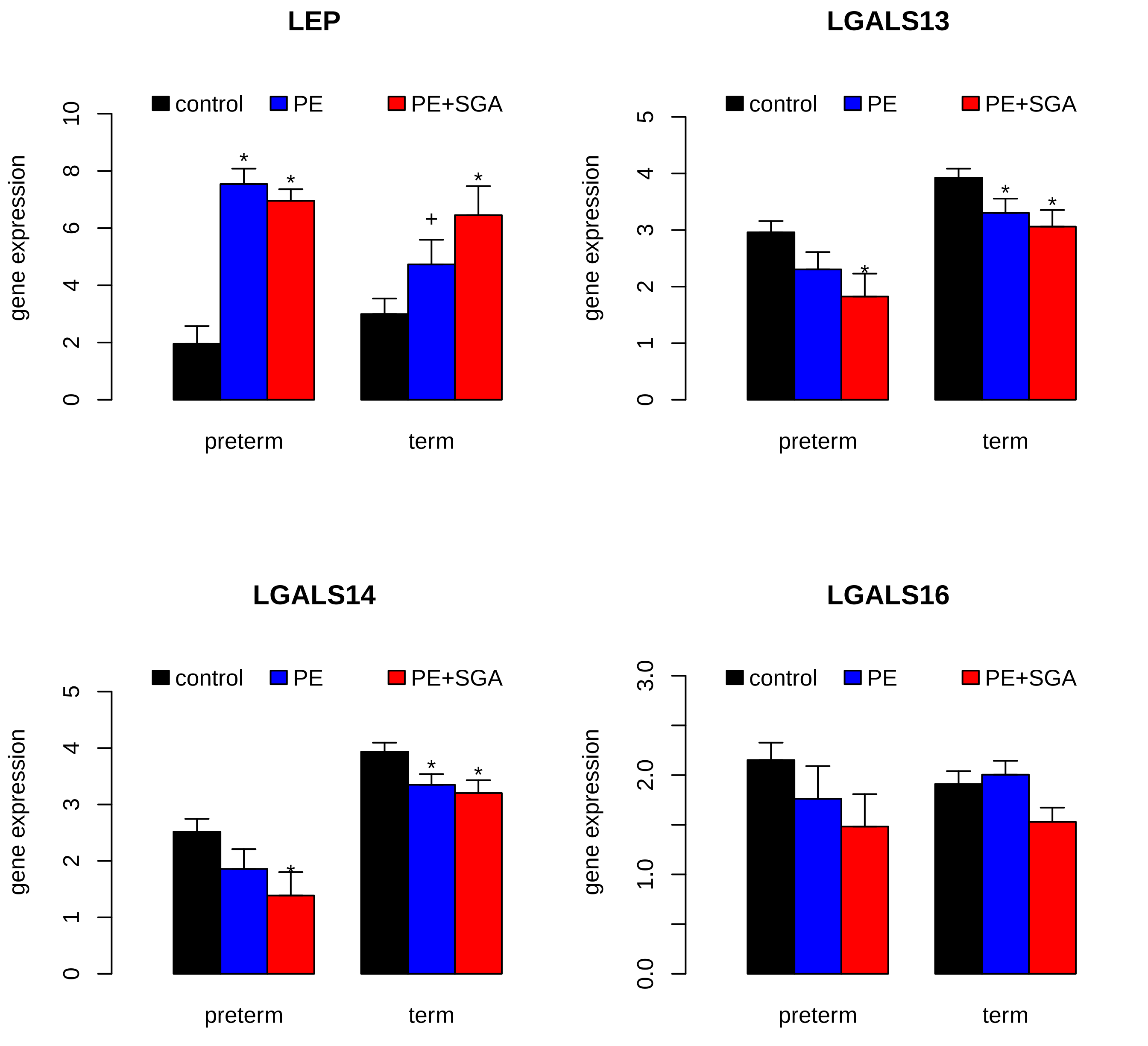
LEP
control
PE
PE+SGA
gene expression
0
2
4
6
8
10
preterm
term
LGALS13
0
1
2
3
4
5
LGALS14
LGALS16
0.0
1.0
2.0
3.0

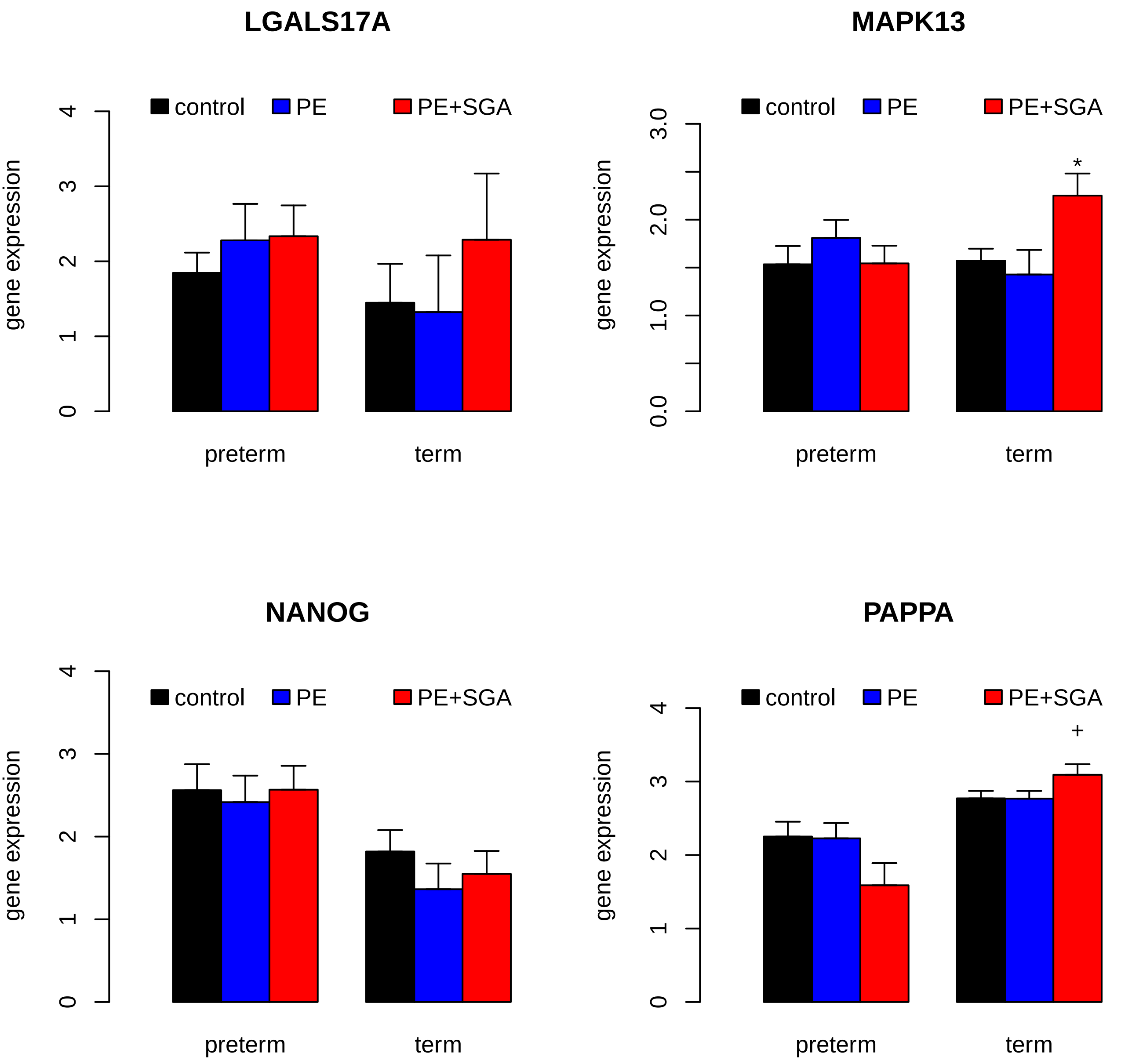
LGALS17A
MAPK13
NANOG
PAPPA
control
PE
PE+SGA
gene expression
preterm
term
0
1
2
3
4
0.0
1.0
2.0
3.0
*
+

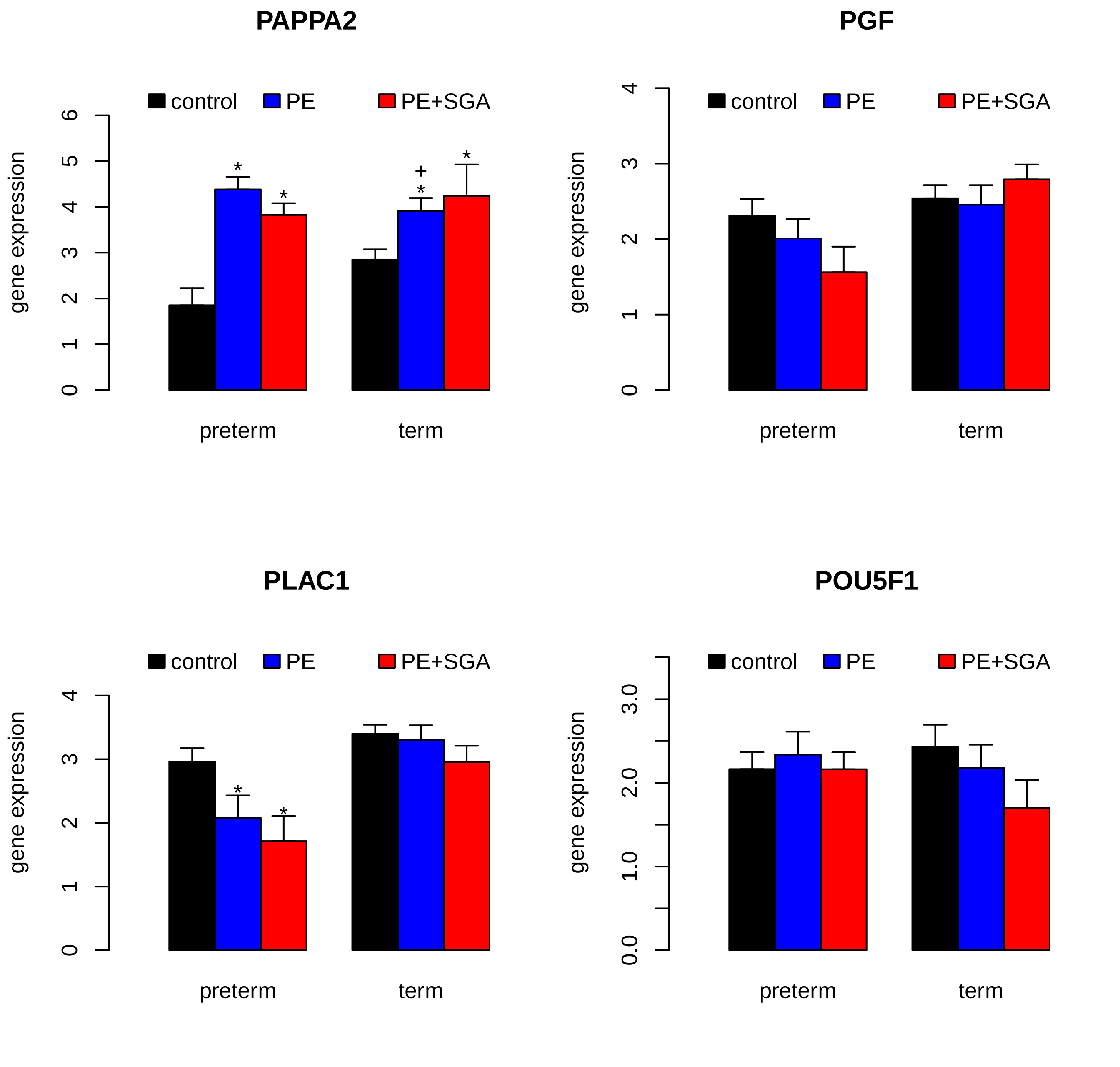
PAPPA2
PGF
PLAC1
POU5F1
control
PE
PE+SGA
gene expression
preterm
term

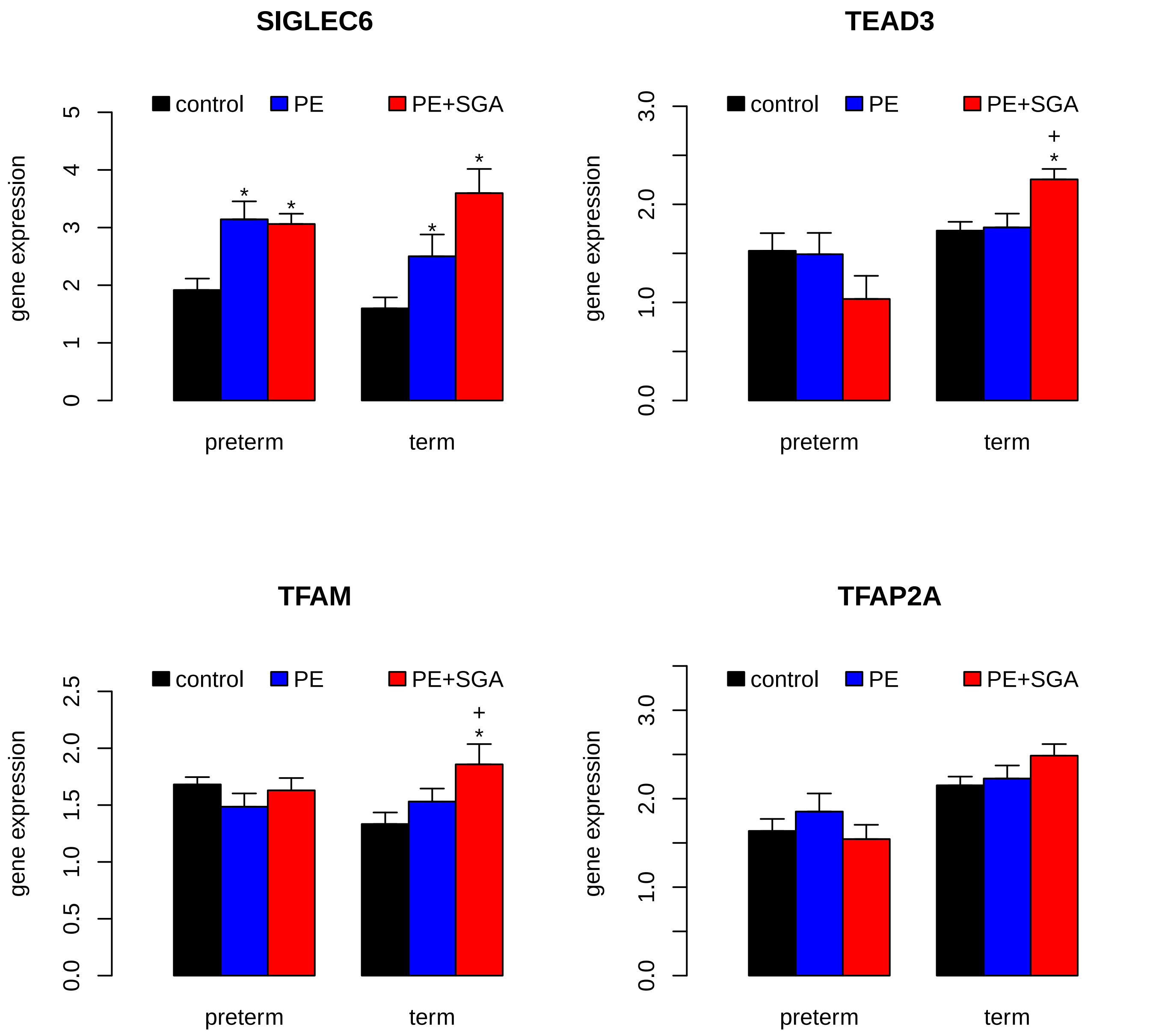
SIGLEC6
TEAD3
TFAM
TFAP2A
control
PE
PE+SGA
gene expression
preterm
term
*
+

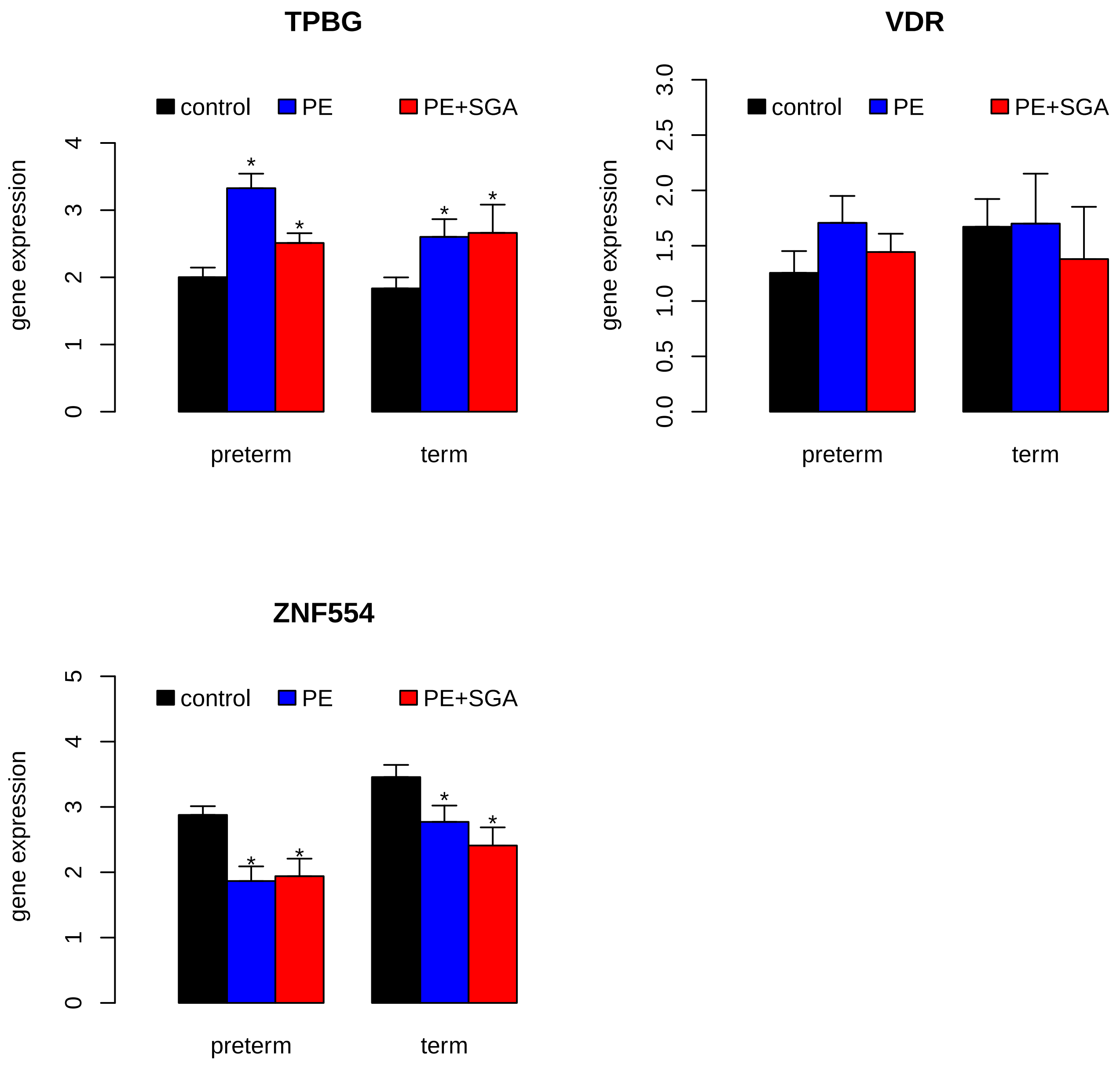
TPBG
control
PE
PE+SGA
gene expression
0
1
2
3
4
*
preterm
term
VDR
control
PE
PE+SGA
gene expression
0.0
0.5
1.0
1.5
2.0
2.5
3.0
preterm
term
ZNF554
control
PE
PE+SGA
gene expression
0
1
2
3
4
5
*
preterm
term
