# Supplementary material for: Integrated Systems Biology Approach Identifies Novel Maternal and Placental Pathways of Preeclampsia
Source: Front Immunol. 2018 Aug 8;9:1661. doi: 10.3389/fimmu.2018.01661 (PMC6092567; doi:10.3389/fimmu.2018.01661)
Supplement: Figure S6 — Maternal blood proteomic changes in term preeclampsia and their effect on differentially expressed (DE) genes in the placenta. (A) The 14 DE maternal serum proteins in term preeclampsia belong to six functional groups. (B) These 14 proteins have connections with 116 DE placental genes, among which 46 belong to the M2 (red) module. Angiotensinogen has more connections than other proteins (OR = 2.5, p = 1.6 × 10^−8^) and the most with M2 (red) module genes (n = 35). Seventy seven of 86 connections of angiotensinogen have a directional effect toward the gene. [file Image_6.pdf]

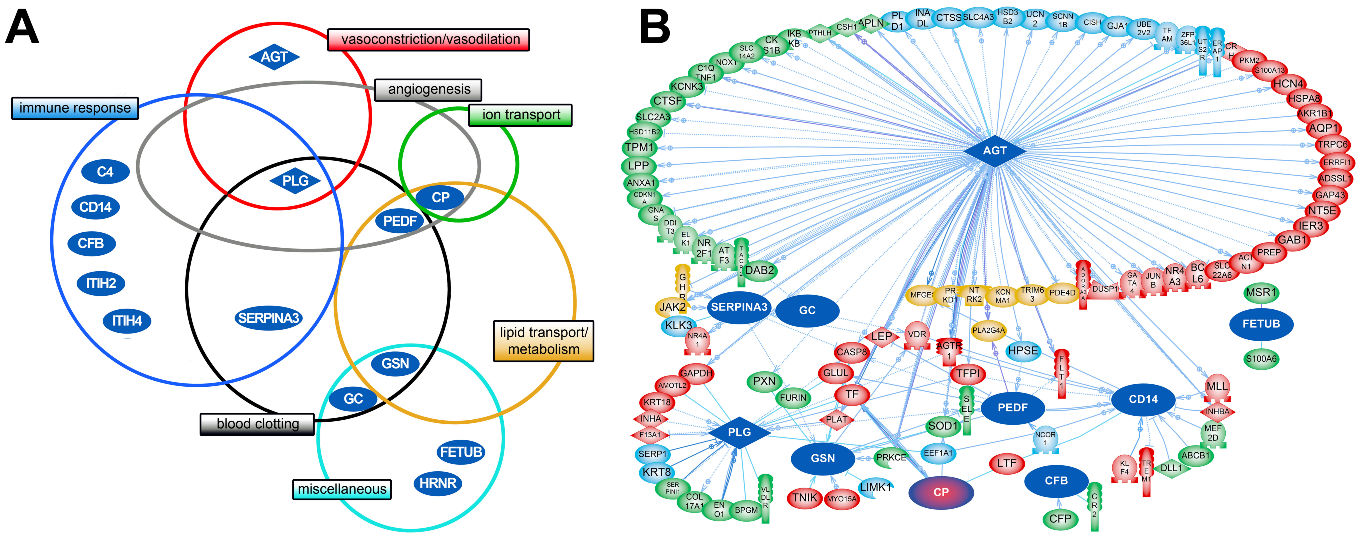
A
vasoconstriction/vasodilation
AGT
angiogenesis
immune response
ion transport
C4
PLG
CP
CD14
PEDF
CFB
ITIH2
SERPINA3
ITIH4
lipid transport/ metabolism
GSN
GC
blood clotting
FETUB
HRNR
miscellaneous
B
AGT
PTHLH
CSH1
APLN
PLD1
INADL
CTSS
SLC4A3
HSD3B2
UCN2
SCNN1B
CISH
GJA1
UBE2V2
TFAM
ZFP36L1
UTS2R
ERAP1
CRH
IKBKB
CKS1B
SLC14A2
NOX1
C1QTNF1
KCNK3
CTSF
SLC2A3
HSD11B2
TPM1
LPP
ANXA1
CDKN1A
GNAS
DDIT3
ELK1
NR2F1
ATF3
TACR2
DAB2
PKM2
S100A13
HCN4
HSPA8
AKR1B1
AQP1
TRPC6
ERRFI1
ADSSL1
GAP43
NT5E
IER3
GAB1
PREP
ACTN1
SLC22A6
BCL6
NR4A3
JUNB
GATA4
DUSP1
ADORA2A
PDE4D
TRIM63
KCNMA1
NTRK2
PRKD1
MFGE8
GHR
JAK2
SERPINA3
GC
KLK3
NR4A1
LEP
VDR
AGTR1
CASP8
GLUL
TFPI
PLA2G4A
HPSE
FLT1
MSR1
FETUB
S100A6
GAPDH
AMOTL2
KRT18
INHA
F13A1
SERP1
KRT8
SERPINI1
COL17A1
ENO1
BPGM
VLDLR
PLG
PXN
FURIN
TF
PLAT
GSN
TNIK
MYO15A
LIMK1
PRKCE
CP
SELE
SOD1
EEF1A1
PEDF
NCOR1
LTF
CFB
CR2
CFP
CD14
MLL
INHBA
MEF2D
ABCB1
DLL1
KLF4
TREM1
