# Supplementary material for: Integrated Systems Biology Approach Identifies Novel Maternal and Placental Pathways of Preeclampsia
Source: Front Immunol. 2018 Aug 8;9:1661. doi: 10.3389/fimmu.2018.01661 (PMC6092567; doi:10.3389/fimmu.2018.01661)
Supplement: Figure S7 — Summary of functional experiments on module M2. Epigenetic changes to the trophoblast and abnormal trophoblast differentiation lead to a general down-regulation of gene expression and the up-regulation of hub factors in module M2 (e.g. BCL6). After placental circulation has been established and placental ischemic stress occurs, the up-regulation of BCL6 sensitizes the trophoblast to ischemia by inducing ARNT2 up-regulation and downstream increase of expression of FLT1, ENG, LEP, leading to the placental release of pro-inflammatory and anti-angiogenic gene products. This pathway is only observed in preterm preeclampsia, suggesting that the dysregulation of this placental pathway promotes the early development of preeclampsia. The alterations in maternal blood proteome can induce trophoblastic functional changes leading to the up-regulation of module M2 genes, the overproduction of sFlt-1 and an anti-angiogenic state through a trajectory that does not necessarily affect fetal growth. [file Image_7.pdf]

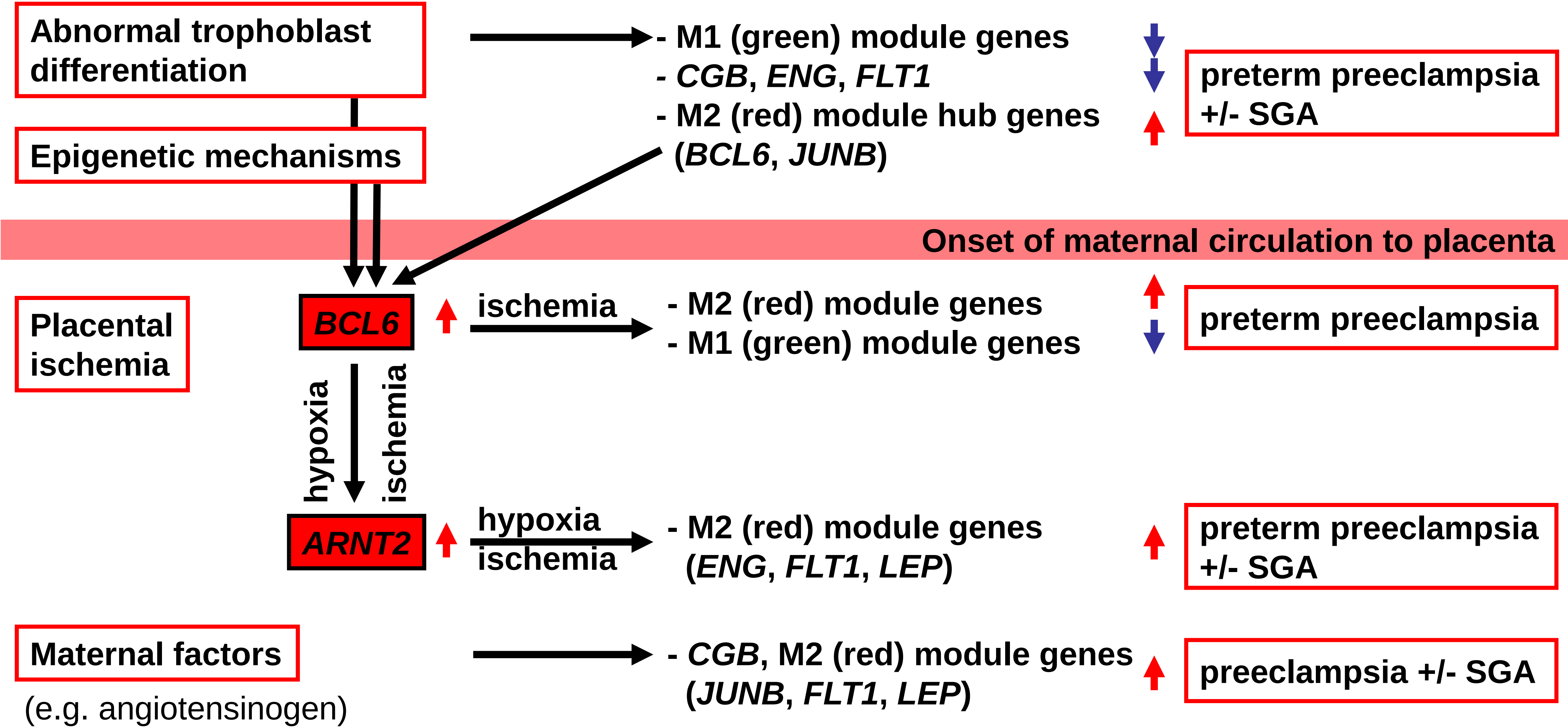
Abnormal trophoblast differentiation
Epigenetic mechanisms
- M1 (green) module genes
- *CGB*, *ENG*, *FLT1*
- M2 (red) module hub genes (*BCL6*, *JUNB*)
preterm preeclampsia +/- SGA
Onset of maternal circulation to placenta
Placental ischemia
BCL6
ischemia
- M2 (red) module genes
- M1 (green) module genes
preterm preeclampsia
hypoxia
ischemia
ARNT2
hypoxia
ischemia
- M2 (red) module genes (*ENG*, *FLT1*, *LEP*)
preterm preeclampsia +/- SGA
Maternal factors
(e.g. angiotensinogen)
- *CGB*, M2 (red) module genes (*JUNB*, *FLT1*, *LEP*)
preeclampsia +/- SGA
